# Machine learning-based prediction of diagnostic markers for Graves’ orbitopathy

**DOI:** 10.1007/s12020-023-03349-z

**Published:** 2023-04-15

**Authors:** Yunying Cai, Heng Su, Yongting Si, Ninghua Ni

**Affiliations:** 1 Department of Endocrinology, The First People’s Hospital of Yunnan Province. The Affiliated Hospital of Kunming University of Science and Technology, Kunming City, Yunnan Provence China; 2 Department of Ophthalmology, The First People’s Hospital of Yunnan Province. The Affiliated Hospital of Kunming University of Science and Technology, Kunming City, Yunnan Provence China

**Keywords:** Graves’ orbitopathy, Methylation, Immune cell infiltration, Machine learning, Bioinformatics

## Abstract

**Purpose:**

The pathogenesis of Graves’ orbitopathy/thyroid-associated orbitopathy (TAO) is still unclear, and abnormal DNA methylation in TAO has been reported. Thus, selecting and exploring TAO biomarkers associated with DNA methylation may provide a reference for new therapeutic targets.

**Methods:**

The TAO-associated expression data and methylation data were downloaded from The Gene Expression Omnibus database. Firstly, weighted gene co-expression network analysis was used to obtain the TAO-related genes, which were intersected with differentially methylated genes (DMGs), and differentially expressed genes between TAO samples and normal samples to obtain TAO-associated DMGs (TA-DMGs). Thereafter, the functions of the TA-DMGs were analyzed, and diagnostic markers were screened by least absolute shrinkage and selection operator (Lasso) regression analysis and support vector machine (SVM) analysis. The expression levels and diagnostic values of the diagnostic markers were also analyzed. Furthermore, single gene pathway enrichment analysis was performed for each diagnostic marker separately using gene set enrichment analysis (GSEA) software. Next, we also performed immune infiltration analysis for each sample in the GSE58331 dataset using the single-sample GSEA algorithm, and the correlation between diagnostic markers and differential immune cells was explored. Lastly, the expressions of diagnostic markers were explored by quantitative real-time polymerase chain reaction (qRT-PCR).

**Results:**

A total of 125 TA-DMGs were obtained. The enrichment analysis results indicated that these TA-DMGs were mainly involved in immune-related pathways, such as Th1 and Th2 cell differentiation and the regulation of innate immune response. Moreover, two diagnostic markers, including *S100A11* and *NKD2*, were obtained by Lasso regression analysis and SVM analysis. Single gene pathway enrichment analysis showed that *S100A11* was involved in protein polyufmylation, pancreatic-mediated proteolysis, and *NKD2* was involved in innate immune response in mucosa, Wnt signaling pathway, etc. Meanwhile, immune cell infiltration analysis screened 12 immune cells, including CD56 dim natural killer cells and Neutrophil cells that significantly differed between TAO and normal samples, with the strongest positive correlation between *NKD2* and CD56 dim natural killer cells. Finally, the qRT-PCR illustrated the expressions of *NKD2* and *S100A11* between normal and TAO.

**Conclusion:**

*NKD2* and *S100A11* were screened as biomarkers of TAO and might be regulated by DNA methylation in TAO, providing a new reference for the diagnosis and treatment of TAO patients.

## Introduction

Graves’ orbitopathy, also called thyroid eye disease or thyroid-associated orbitopathy (TAO), is a disfiguring inflammatory disease affecting the orbit and ocular adnexa. The disease has a variety of clinical presentations, including upper eyelid retraction, restrictive strabismus, proptosis, exposure keratopathy, and optic neuropathy [1]. Previous studies have found that TAO existed in about 50% of Graves’ disease (GD) patients, with female predominance [2, 3]. According to the 2021 European Group on Graves Orbitopathy (EUGOGO), the annual incidence of TAO was 0.54–0.9 cases/100,000 in men and 2.67–3.3 cases/100,000 in women [4, 5]. The current guidelines of the EUGOGO recommend local treatment and selenium for mild TAO and high-dose intravenous glucocorticoid pulses as standard treatment for active and moderate-to-severe disease [4, 6]. However, a substantial number (20–30%) of active moderate-to-severe TAO patients may not respond to current guideline-recommended approaches. It may still require additional surgical corrections to improve vision and appearance [7]. Therefore, more extensive studies are needed to explore the pathogenesis of TAO and novel therapeutic strategies for TAO.

DNA methylation is a process that regulates gene expression without changing the DNA sequence. Studies have shown that alteration in DNA methylation was involved in autoimmune diseases, cancers, fragile X syndrome, and other disorders [8], and the abnormal DNA methylation played a vital role in the occurrence and development of TAO by being involved in regulating oxidative stress, inflammation, adipogenesis, and glycosaminoglycan production [9, 10]. In addition, DNA methylation of T lymphocytes was developed as a future therapeutic and diagnostic target in rheumatoid arthritis (RA) [11]. Thus, understanding the DNA methylation regulation of immune cell infiltration can provide a framework for developing novel, individualized TAO therapeutics.

This study obtained two diagnostic markers, S100A11 and NKD2, by least absolute shrinkage and selection operator (Lasso) regression analysis and support vector machine (SVM) analysis for TAO-related DMGs. Enrichment analysis showed that these diagnostic markers were mainly concentrated in pathways such as innate immune response and Wnt signaling pathway calcium modulating pathway. At the same time, CD56 dim natural killer cells and Neutrophil cells were also screened as differential immune cells. These findings provided targets for the diagnosis and treatment of TAO and had great significance for the further study of TAO.

## Material and methods

### Data source

The gene ontology (GO) expression profiles of the orbit tissue from the GSE58331 and GSE105149 datasets, besides the methylation data of orbit adipose/connective tissue from the GSE175399 dataset, were downloaded from the Gene Expression Omnibus database (https://www.ncbi.nlm.nih.gov/). The GSE58331 dataset contained 35 TAO samples and 29 normal samples, and in the GSE105149 dataset, there were 7 normal and 4 TAO samples. Meanwhile, the GSE175399 dataset included methylation data of 4 normal and 4 TAO samples.

### Determination of differentially expressed genes between TAO and normal samples

The differentially expressed genes (DEGs) between TAO and normal samples in the GSE58331 dataset were selected with *p* < 0.05 and |log_2_fold change (FC)| > 0.5 using the “limma” R package [12]. The distribution of DEGs was shown in the volcano map plotted by “ggplot” R package.

### Determination of TAO-associated genes by weighted gene co-expression network analysis

The “weighted gene co-expression network analysis” (WGCNA) R package was adopted to detect modules of TAO-associated genes (TAGs) in the GSE58331 dataset [13]. Firstly, all genes’ expression data in the GSE58331 dataset were ranked in descending order of variance, and genes with variance greater than 25% were selected for WGCNA analysis. In this study, the disease statuses were grouped into TAO and normal. Next, all samples in the GSE58331 dataset were clustered to see the overall correlation of all samples, and outliers were excluded to ensure the accuracy of the analysis. In addition, the disease statuses of the samples were collated and added to the clustering plot to construct a sample clustering and clinical trait heatmap. Next, the soft threshold was determined to ensure that inter-gene interactions maximally conformed to the scale-free distribution. Naturally, the neighborliness between genes was calculated, and the similarity between genes was calculated based on the neighborliness, and the coefficient of dissimilarity between genes was introduced, on which the systematic clustering tree between genes was obtained. After that, we set the minimum number of genes per gene module to 30 and set MEDissThres = 0.2 to merge the similar modules analyzed by dynamic tree cutting algorithm. The modules were then correlated with the traits, and the key modules associated with the disease were screened according to the threshold values *p* < 0.05 and |*r*| > 0.3, in which genes were TAGs.

### Determination of TAO-associated differentially methylated genes

The methylation data in the GSE175399 dataset was quality-controlled, filtered, and normalized using the champ.filter function and champ.norm function in the “ChAMP” R package [14]. Moreover, the differentially methylated CpG sites (DMCs) between TAO and normal samples were screened using the champ.DMP function and corrected for multiple testing using the Benjamini and Hochberg method. Methylation sites with the threshold of adj.*p* < 0.05 were regarded as DMCs, and the DMCs were annotated to the corresponding genes DMG based on the annotation information. Furthermore, TAO-associated differentially methylated genes (TA-DMGs) were obtained by overlapping the TAGs obtained above, DEGs between TAO and normal samples, and DMGs.

### Functional enrichment analysis of TA-DMGs and construction of protein–protein interaction network

GO and Kyoto Encyclopedia of Genes and Genomes (KEGG) enrichment analyses of TA-DMGs were performed using the “cluster profile” R package [15] with a significance threshold of *p* < 0.05. Following this, the top 10 GO and KEGG terms were selected for presentation using the “ggplot2” R package, ranked according to their *p* value. To further examine the interactions between the above TA-DMGs, the proteins encoded by TA-DMGs were predicted and analyzed for interactions using the STRING (https://version-11-0.string-db.org/) database, and a protein–protein interaction (PPI) network was constructed using Cytoscape software.

### Diagnostic marker detection by machine learning

Based on the above-obtained expression values of TA-DMGs for each sample in the GSE58331 dataset, we combined the grouping information of the samples to construct lasso regressions to predict sample classification. To reduce the feature dimension, we used the “glmnet” (version 4.0-2) R package [16] to perform 10-fold cross-validation, calculate the error rate for different features, select strongly correlated features, and filter out the signature genes. Meanwhile, we used the “e1071” (version1.7-9) R package to rank the TA-DMGs by SVM algorithm, obtained the importance of each gene using the recursive feature elimination (RFE) method and ranked them, and obtained the error rate and accuracy rate of each combination iteration. The lowest error rate was selected as the best combination, and the corresponding gene was taken out as the signature gene. The intersecting genes of the signature genes screened by Lasso analysis and SVM analysis, respectively, were the diagnostic marker.

### Diagnostic value analysis

To evaluate the diagnostic value of each diagnostic marker, we performed receiver operating characteristic (ROC) curve analysis for each biomarker in the GSE58331 dataset and GSE105149 dataset using the “pROC” R package, respectively. The expression of each diagnostic marker was extracted separately, and the expression of each diagnostic marker was plotted in the above two datasets using the “ggplot2” R package together with the sample grouping information of the respective datasets. In addition, the methylation levels of each diagnostic marker in TAO and normal samples were compared by *t*-test in the GSE175399 dataset.

### Gene set enrichment analysis enrichment analysis

The above gene set was used as background genes for enrichment analysis using gene set enrichment analysis (GSEA) software (V4.0.3). The significant enrichment threshold was set at NOM *p* < 0.05, and we selected TOP 5 enrichment results for each GO and KEGG pathway for each diagnostic marker separately for presentation.

### Immune infiltration analysis

Immune cell infiltrating tumors are most likely to be used as drug targets to improve patient survival [17]. In this study, the single-sample GSEA algorithm was used to calculate the infiltration abundance of 28 immune cells in all samples in the GSE58331 dataset, and immune cells in disease and normal samples were compared by the Wilcox test method. To further understand the relationships between diagnostic markers and differential immune cells, the correlations between diagnostic markers and differential immune cells were calculated using Spearman correlation analysis. The correlation heatmap between each diagnostic marker and differential immune cells was plotted using the “ggplot2” R package.

### Quantitative real-time polymerase chain reaction (qRT-PCR)

Blood samples of five TAO patients and five normal samples were recruited from the First People’s Hospital of Yunnan Province. All samples endorsed informed consent forms and the study passed the ethical review of First People’s Hospital of Yunnan Province (No. KHLL2016-KY038). First, total RNA was extracted by TRIzol Reagent from Ambion Inc. Then, reverse transcription reaction was performed by SureScript First strand cDNA synthesis kit provided by Servicebio. PCR was conducted using the 2xUniversal Blue SYBR Green qPCR Master Mix kit provided by Servicebio. The PCR conditions were 95 °C pre-denaturation for 1 min, then 40 cycles. Each cycle included 95 °C denaturation for 20 s, 55 °C annealing for 20 s, and 72 °C extension for 30 s. GAPDH was used as an internal reference for gene detection. Primer sequences were shown in Supplementary Table S1. The expressions of *NKD2* and *S100A11* in TAO and normal samples were compared by *t*-test.

## Results

### Determination of DEGs between TAO and normal samples

**Fig. 1:**
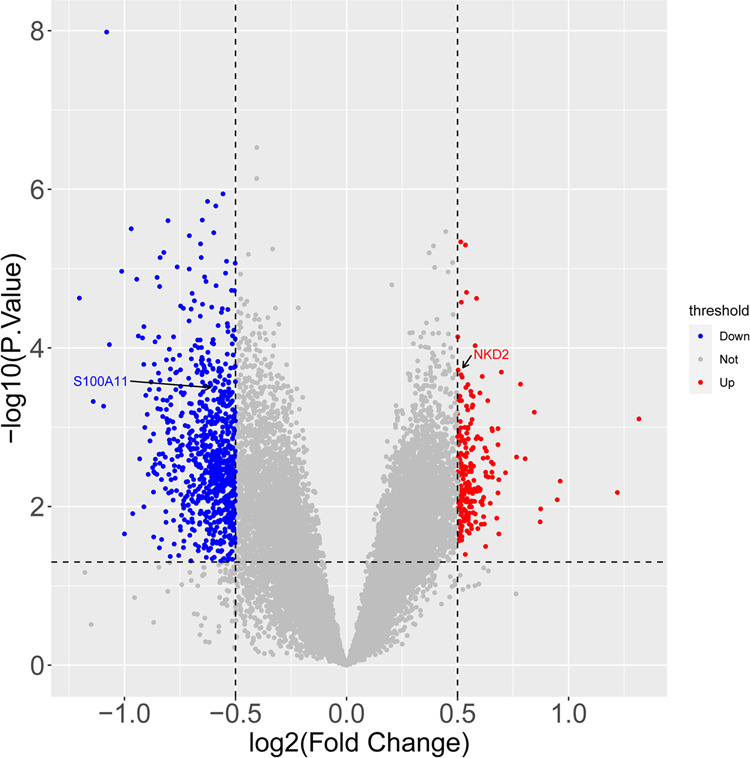
Determination of DEGs between TAO and normal samples. The volcano plot showed all differentially expressed genes (DEGs) between TAO and normal samples. Red indicates that the expression of genes is relatively upregulated, and blue means that the expression of genes is relatively downregulated. The number of genes in each module is listed in Supplementary Table S1

The volcano plot of DEGs was shown in Fig. 1. There were 994 significant DEGs between TAO and normal samples, containing 218 upregulated DEGs and 776 downregulated DEGs (Supplementary Table S2).

### Determination of TAGs by WGCNA

**Fig. 2:**
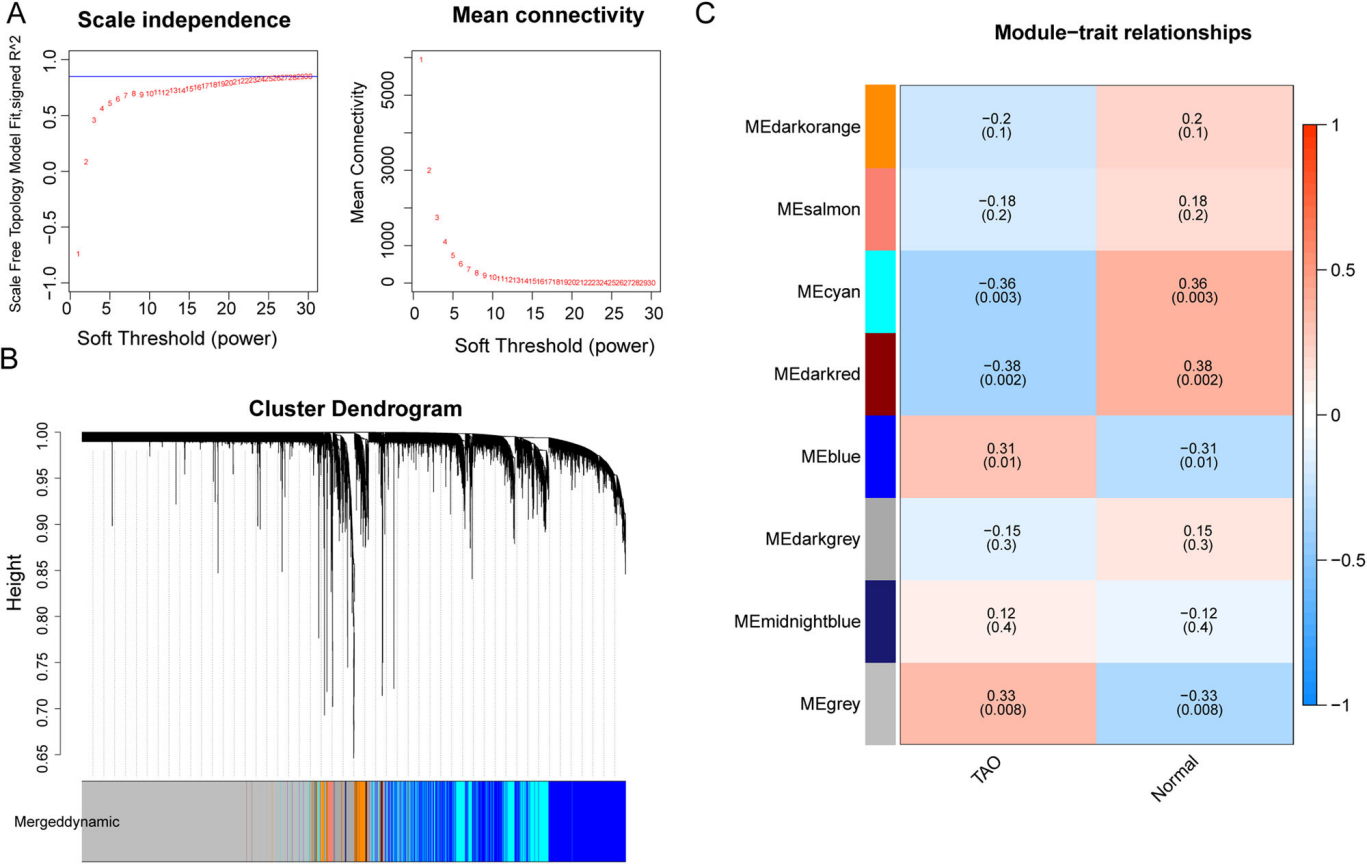
Determination of TAO-associated genes (TAGs) by weighted gene co-expression network analysis (WGCNA). **A** Analysis of network topology for various soft-thresholding powers. **B** Clustering dendrogram of genes, with dissimilarity based on the topological overlap and assigned module colors. **C** Module-trait associations. Each row corresponds to a module, and each column corresponds to a trait. Each cell contains the corresponding correlation and *p* value. The table is color-coded by correlation according to the color legend. Heatmap of the correlation between module eigengenes and the clinical modules

A total of 16,230 genes with variance greater than 25% were obtained in the GSE58331 database for WGCA analysis. The sample clustering plot showed that the samples clustered well, so no sample rejection was required (Supplementary Fig. 1A). Supplementary Fig. 1B showed the sample clustering and clinical trait heatmap. The power threshold was chosen to be 30 when the interaction between genes maximally conformed to the scale-free distribution (Fig. 2A). The hybrid dynamic shear tree algorithm obtained 28 modules, and a total of 8 modules were obtained after merging similar modules (Fig. 2B). The genes contained in each module were as Supplementary Table S3. Three key modules associated with GO were obtained by module-trait association analysis (Fig. 2C), namely cyan module (2643 genes), dark red module (81 genes), and blue module (4603 genes), and a total of 7327 TAGs were obtained by combining genes in the three modules.

### Determination of TA-DMGs

**Fig. 3:**
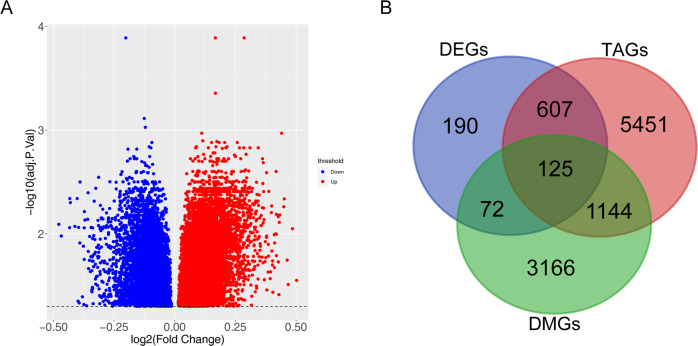
Determination of TA-DMGs between TAO and normal samples. **A** Volcano plot of DMGs. **B** Venn plot of TAGs, DEGs, and DMGs between TAO and normal samples

In total, 12,404 DMCs were obtained by differential analysis, and 4507 DMGs were obtained after annotation (Fig. 3A). A sum of 125 TA-DMGs (Supplementary Table S3) was obtained after taking the intersection of 7327 TAGs, 994 DEGs between TAO and normal samples, and 4507 DMGs (Fig. 3B).

### Functional enrichment analysis of TA-DMGs and construction of PPI network

**Fig. 4:**
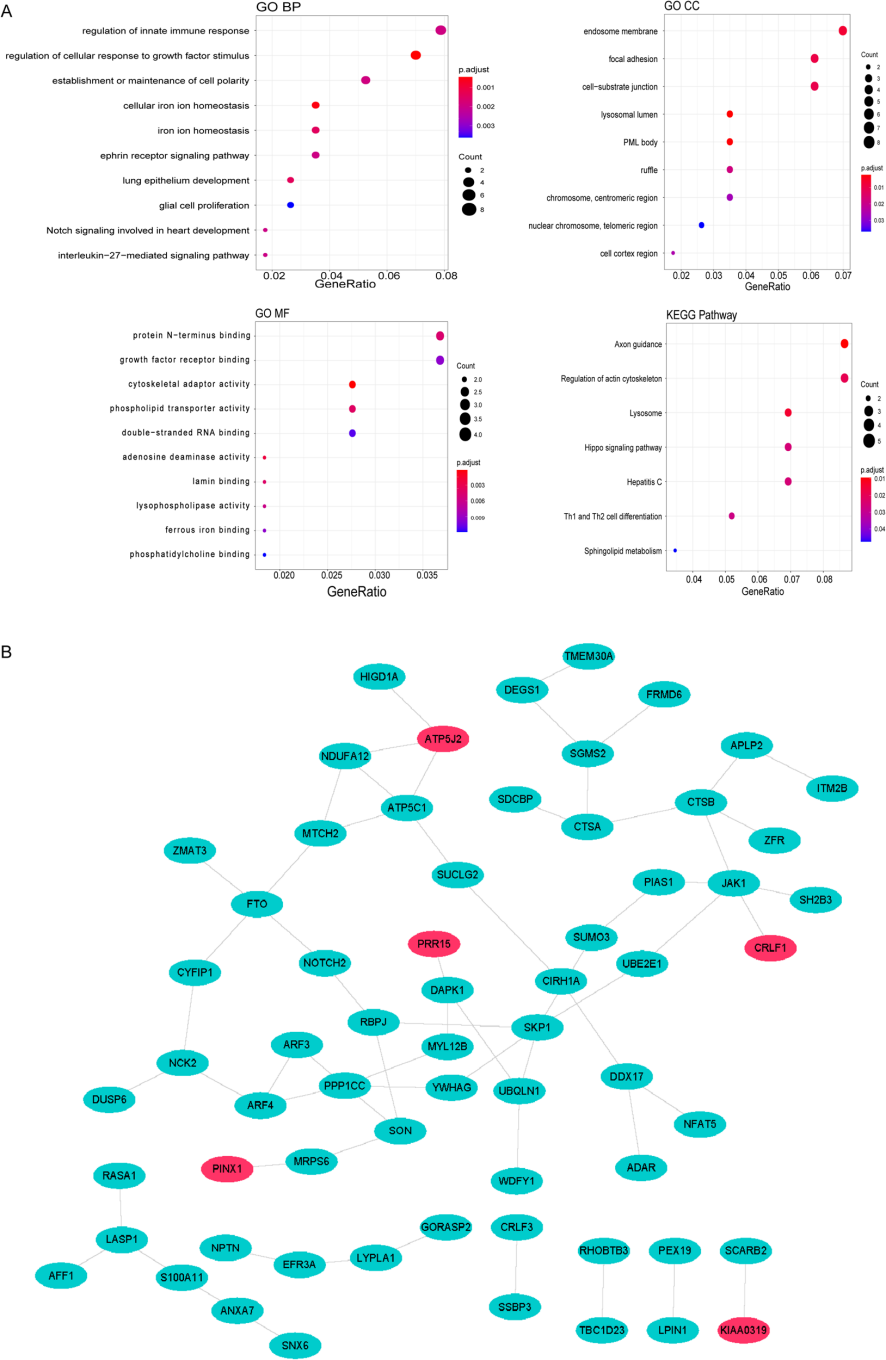
Functional enrichment analysis of TA-DMGs and PPI network. **A** Gene Ontology (GO) functional and KEGG pathway enrichment analysis for TA-DMGs. **B** Protein–protein interaction network of the common differentially methylated genes (DMGs) set. Blue indicates downregulation while red represents upregulation

Functional enrichment analysis of 125 TA-DMGs revealed about 80 GO biological processes (BP), 9 GO cellular components, 21 GO molecular functions, and 7 KEGG pathways (Supplementary Tables S4–S7), which were enriched for Th1 and Th2 cell differentiation, regulation of innate immune response, neutrophil degranulation, neutrophil activation involved in immune response, neutrophil-mediated immunity, negative regulation of immune response, neutrophil activation, and other immune-related pathways. The GO and KEGG top 10 terms were presented in Fig. 4A. The PPI network constructed by TA-DMGs predicted proteins was shown in Fig. 4B, and the results obtained 64 protein-interaction pairs containing 64 protein nodes, including four upregulated differential proteins and 60 downregulated differential proteins (see Supplementary Table S8).

### Diagnostic marker detection by machine learning

**Fig. 5:**
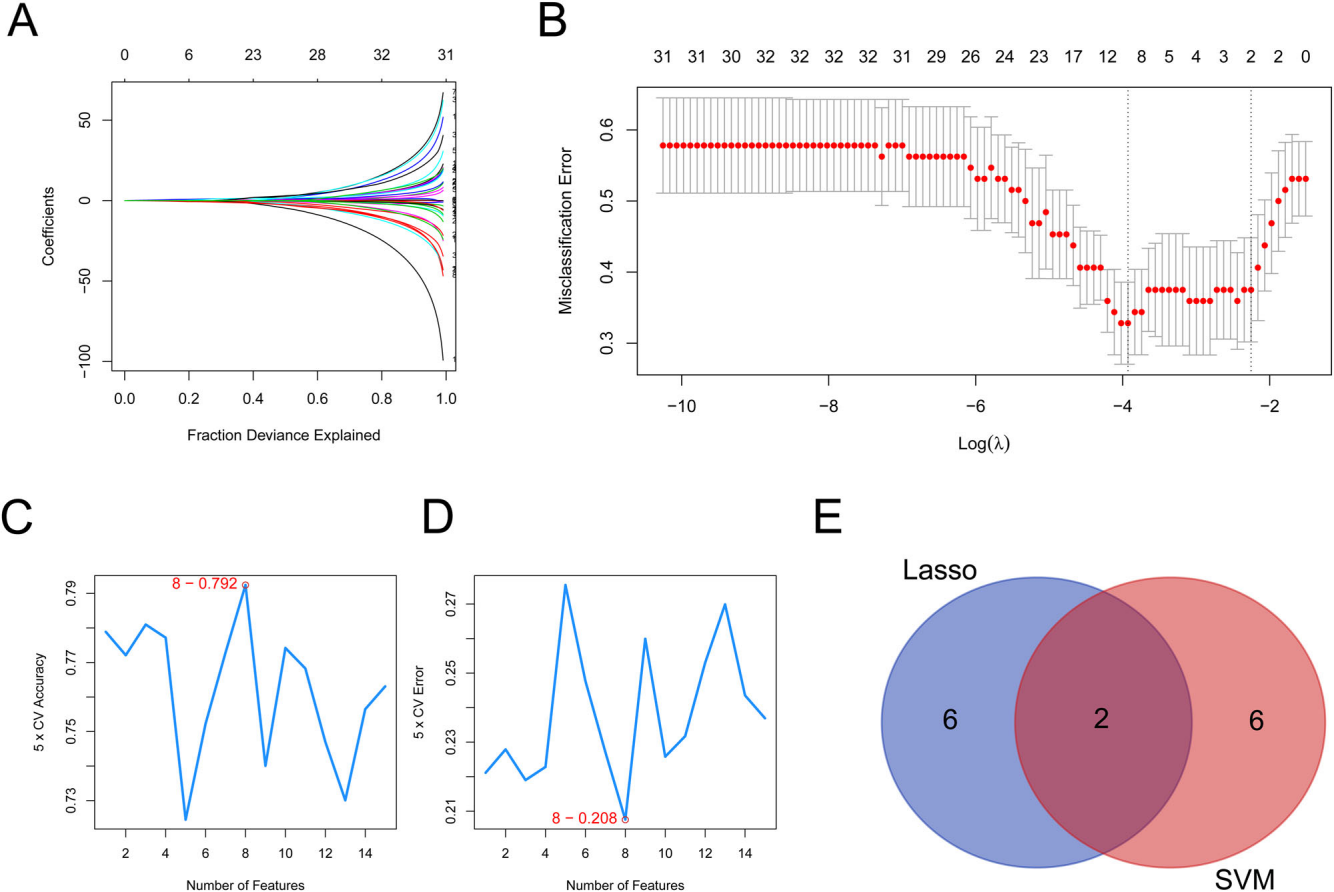
Diagnostic marker detection by machine learning. **A**, **B** The gene coefficient plot and cross-validation error plot were obtained from the lasso regression. Eight signature genes (*ADAD2, ADAMTSL2, EHBP1, GPR144, MRPS6, NKD2, NRIP2*, and *S100A11*) were screened. **C**, **D** The results of the SVM analysis. Eight signature genes were selected (*GDE1, NKD2, PTTG1IP, ATP5J2, SGCE, S100A11, SKP1*, and *BNIP3L*). **E** Venn plot of signature genes by lasso regression and SVM analysis

The gene coefficient plot and cross-validation error plot obtained from the lasso regression were shown in Fig. 5A, B. The lowest error rate was reached at a lambda. min of 0.0197 and eight signature genes were screened out, namely *ADAD2*, *ADAMTSL2*, *EHBP1*, *GPR144*, *MRPS6*, *NKD2*, *NRIP2*, and *S100A11*. Figure 5C, D showed the accuracy of the model under different features. In the SVM analysis, we calculated the accuracy under different features using a 5-fold cross-validation, and the model reached the highest accuracy after selecting the first eight features, at which point 8 signature genes were selected, namely *GDE1*, *NKD2*, *PTTG1IP*, *ATP5J2*, *SGCE*, *S100A11*, *SKP1*, and *BNIP3L*. The SVM-RFE model feature rankings were shown in Table 1. The signature genes screened by Lasso analysis and SVM analysis were intersected to obtain an overall total of *S100A11* and *NKD2* diagnostic markers for subsequent analysis (Fig. 5E).

**Table 1:** The SVM-RFE model feature rankings

No.	FeatureName	FeatureID	AvgRank
1	GDE1	41	16
2	NKD2	72	16.6
3	PTTG1IP	89	17.6
4	ATP5J2	15	18
5	SGCE	101	18.2
6	S100A11	79	18.4
7	SKP1	106	21.4
8	BNIP3L	18	24.2

### Diagnostic value analysis

**Fig. 6:**
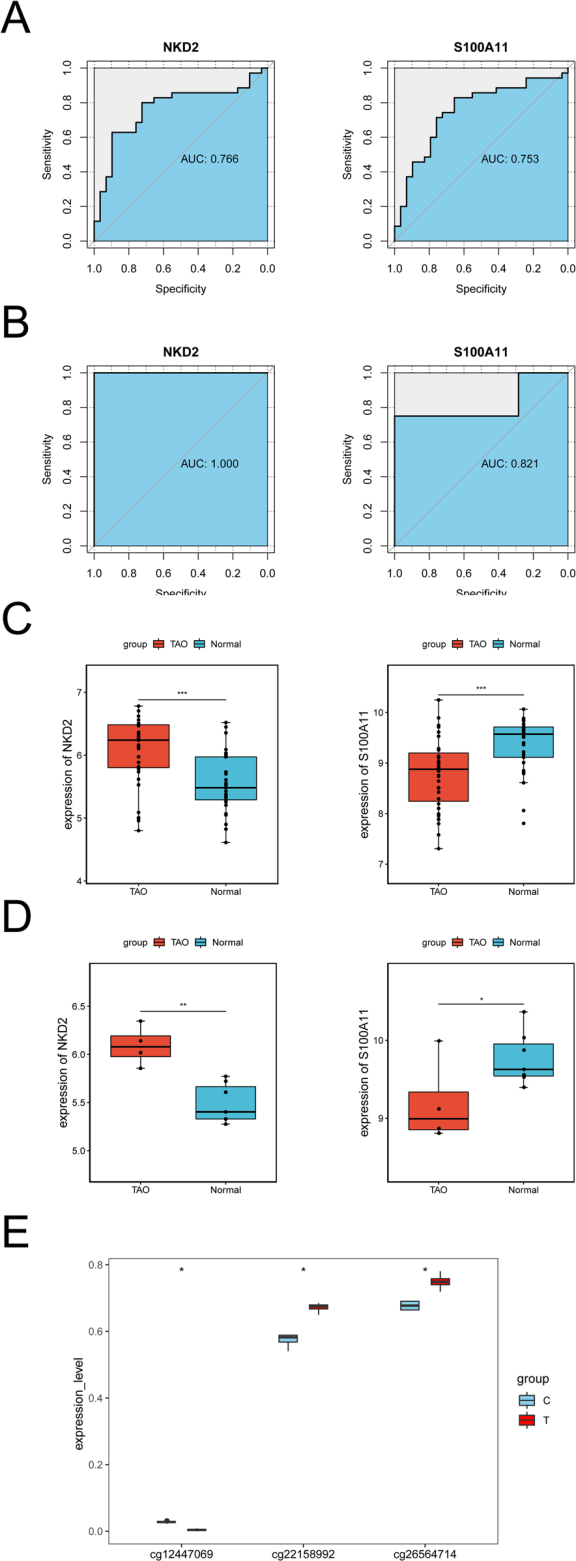
The diagnostic performance of machine learning for the prediction of biomarkers. **A** The receiver operating characteristic curve (ROC) of *NKD2* and *S100A11* in the training group. **B** The ROC curve of *NKD2* and *S100A11* in the validation set. **C**, **D** The boxplots of *NKD2* and *S100A11* in the GSE58331 and GSE105149 datasets. **E** Boxplot of the methylation levels of *NKD2* and *S100A11* in TAO samples and normal samples

The ROC results showed that the area under curve (AUC) value of each diagnostic marker in the GSE58331 dataset reached above 0.75, and the AUC value of each diagnostic marker in the GSE105149 dataset reached above 0.8, indicating that each diagnostic marker had the diagnostic ability to distinguish between disease and normal samples (Fig. 6A, B). The expression trends of the diagnostic markers in the GSE58331 and GSE105149 datasets were consistent, with *NKD2* belonging to differentially upregulated genes and *S100A11* belonging to differentially downregulated genes in the TAO samples (Fig. 6C, D). It can be seen from the boxplot of differential methylation levels of diagnostic markers that the cg26564714 methylation level and the cg22158992 methylation level of *NKD2* gene were upregulated in TAO samples, and the cg12447069 methylation level of *S100A11* gene was downregulated in TAO samples (Fig. 6E).

### GSEA enrichment analysis

**Fig. 7:**
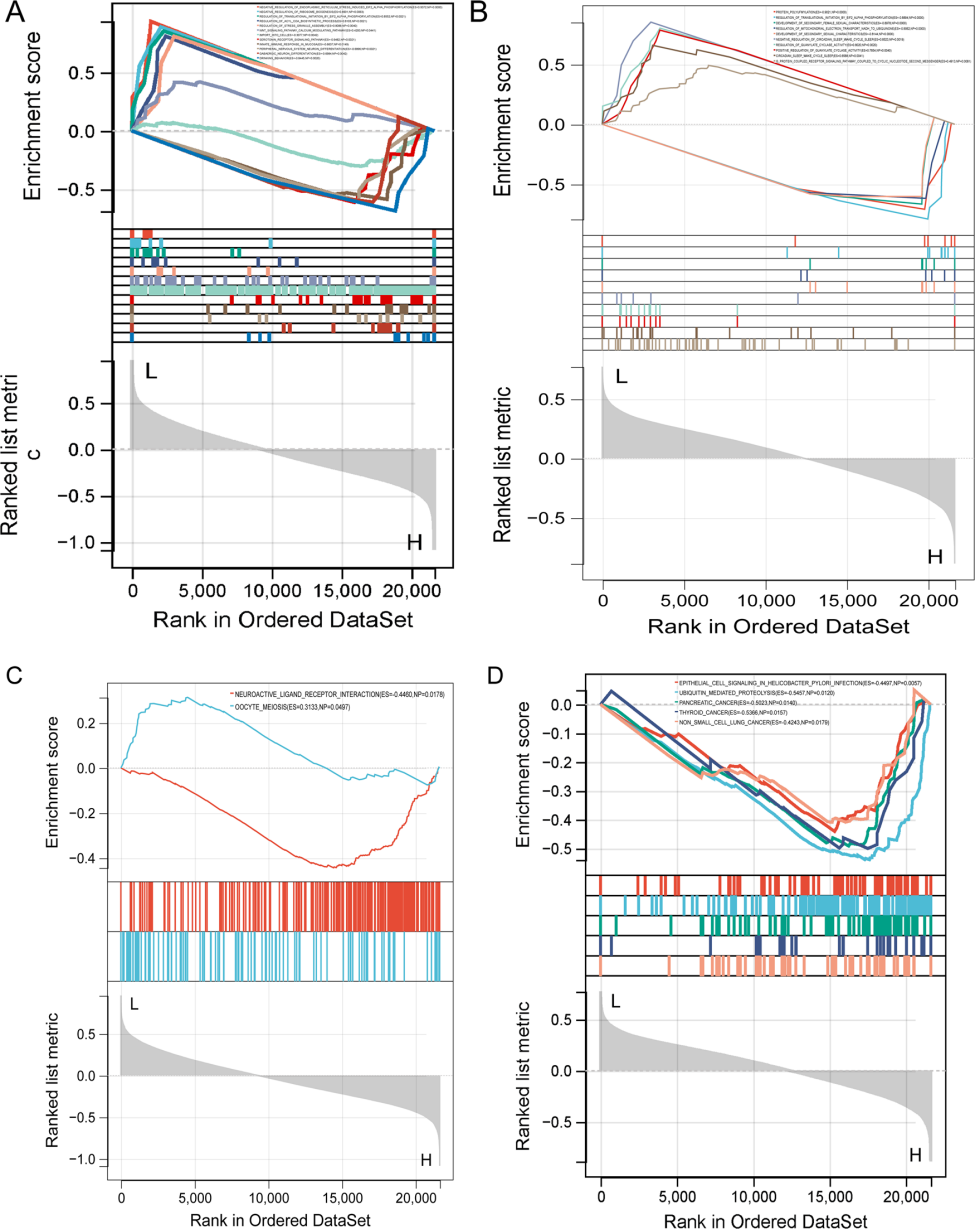
GSEA for *NKD2* and *S100A11*. **A** The GO result of NKD2. **B** The KEGG result of *NKD2*. **C** The GO result of *S100A11*. **D** The KEGG result of *S100A11*. Each line represents one particular gene set with unique color. Upregulated genes are located on the left, approaching the origin of the coordinates; by contrast, the downregulated lay on the right of the *x*-axis. Only gene sets with NOM *p* < 0.05 were considered significant, and only several leading gene sets were displayed in the plot

Gene enrichment analysis showed that *NKD2* was enriched with 176 GO BPs and 2 KEGG pathways, and *S100A11* was enriched with 451 GO BPs and 18 KEGG pathways. The top 10 GO and KEGG enrichment results of *NKD2* diagnostic markers were shown in Fig. 7A, B. The figure showed that *NKD2* regulated endoplasmic reticulum stress-induced EIF2 alpha phosphorylation, ribosome biogenesis regulation, translational initiation by EIF2 alpha phosphorylation, and WNT signaling pathway calcium modulating pathway, and other GO functions. It is involved in Neuroactive Ligand receptor interaction and Oocyte meiosis KEGG pathways. *S100A11* participated in protein polyufmylation, regulation of translational initiation by EIF2 alpha phosphorylation, development of secondary female sexual characteristics, regulation of mitochondrial electron transport NADH to ubiquinone, development of secondary sexual characteristics, and other GO functions and KEGG pathways such as epithelial cell signaling in *Helicobacter pylori* infection, ubiquitin-mediated proteolysis, pancreatic cancer, thyroid cancer, non-small cell lung cancer (Fig. 7C, D).

### Immune infiltration analysis

**Fig. 8:**
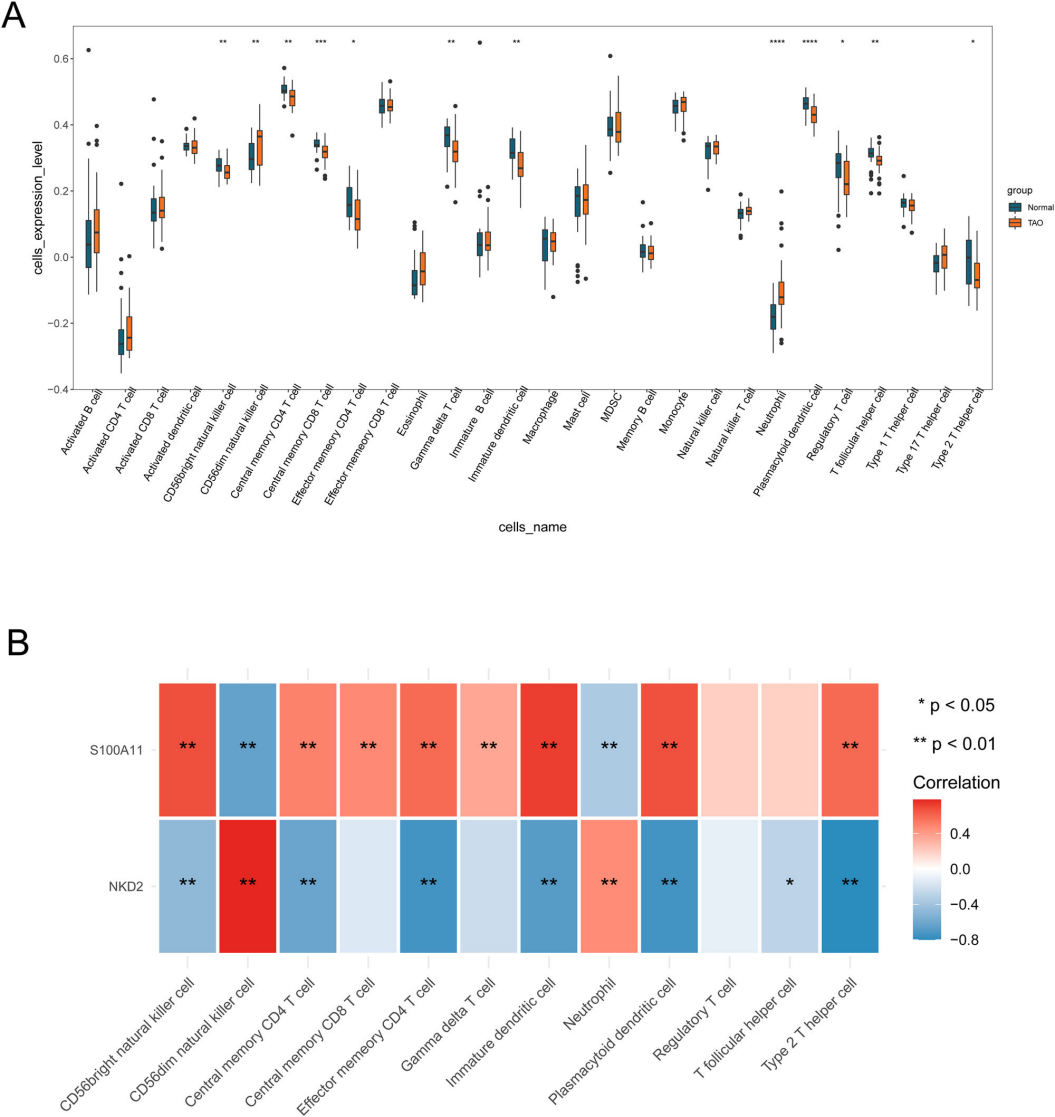
Immune cell infiltration analysis in TAO. **A** Twelve differential immune cells were found between TAO and normal samples. **B** Heatmap of the correlation between NKD2 and s100A11 with immune cells in TAO. *p < 0.05, **p < 0.01

There were 12 differential immune cells between TAO and normal samples, with CD56 dim natural killer cells and Neutrophil cells being upregulated in TAO samples, CD56 bright natural killer cells, central memory CD4 T cells, central memory CD8 T cells, effector memory CD4 T cells, gamma delta T cells, immature dendritic cells, plasmacytoid dendritic cells, regulatory T cells, T follicular helper cell, and Type 2 T helper cell ten cells were downregulated in the TAO samples (Fig. 8A and Supplementary Table S9). A heatmap of the correlation between each diagnostic marker and the differential immune cells was shown in Fig. 8B. Among them, *S100A11* was significantly positively correlated with CD56 bright natural killer cell, central memory CD4 T cell, central memory CD8 T cell, effector memory CD4 T cell, gamma delta T cell, immature dendritic cell, plasmacytoid dendritic cell, and type 2 T helper cell, and negatively correlated with CD56 dim natural killer cell and neutrophils cell. *NKD2* was significantly positively associated with CD56 dim natural killer cell and neutrophil and significantly negatively correlated with CD56 bright natural killer cell, central memory CD4 T cell, effector memory CD4 T cell, immature dendritic cell, plasmacytoid dendritic cell, T follicular helper cell, and Type 2 T helper cell (Supplementary Table S10).

### The results of qRT-PCR

**Fig. 9:**
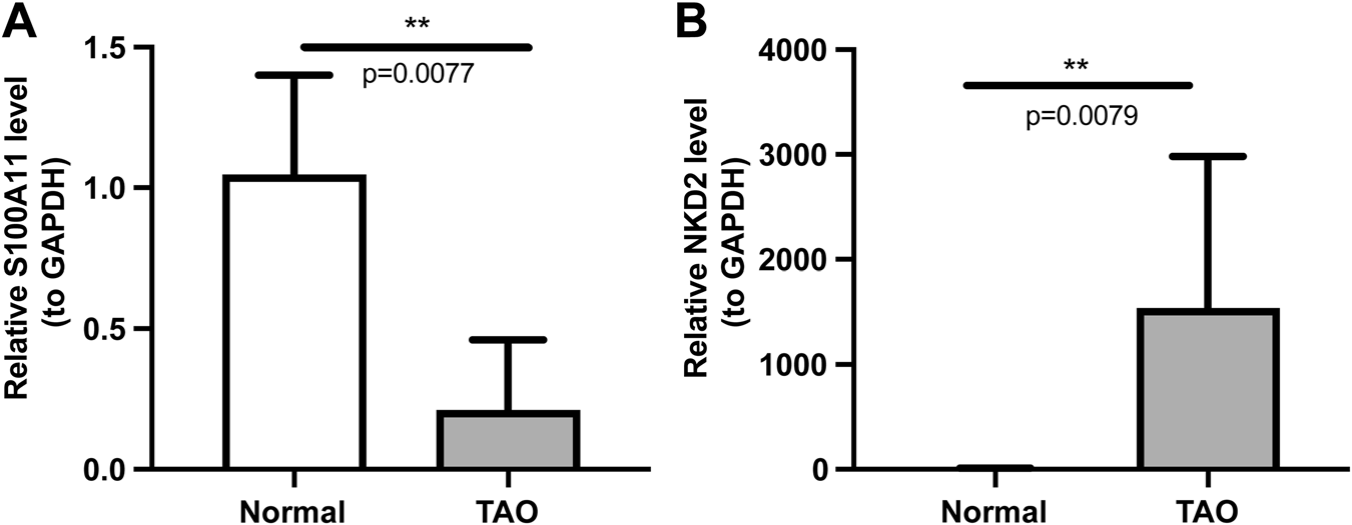
qRT-PCR for *NKD2* and *S100A11*. **A** S100A11 was significantly downregulated in TAO. **B** NKD2 in TAO was significantly upregulated. *p < 0.05, **p < 0.01

Compared with normal samples, the expression of *S100A11* in TAO was significantly downregulated, and the expressions of *NKD2* and in TAO were significantly upregulated (Fig. 9), which was consistent with our analysis.

## Discussion

TAO is a vexing autoimmune condition that causes early orbital inflammation and late tissue remodeling, with cellular and humoral immunity forming a complex regulatory network. Previous studies have revealed the relationship between DNA methylation and TAO, and several genomic loci were identified [9, 10, 18–20], such as interleukin 17 receptor E and cyclin-dependent kinase 5. However, the methylation-related pathogenesis of TAO has not been fully elucidated.

In this study, we identified 125 DEGs associated with the development of TAO, which was mainly involved in immunity-related pathways, including Th1 and Th2 cell differentiation, regulation of innate immune response, neutrophil activation involved in immune response, and other immune-related pathways.

Th1 and Th2 cells of the adaptive immune system play a vital role in the pathogenesis of immune-mediated inflammatory diseases [21]. According to previous studies, Type 1, Type 2, and Type 17 helper T cells and regulatory T cells may participate in the pathogenesis of TAO through multiple mechanisms, including activating B cells, promoting adhesion molecule expression, and producing inflammatory cytokines [22, 23]. In the orbital immune microenvironment, T cells are the primary immune cells that activate orbital fibroblasts (OFs). They activate OFs via CD40-CD40L costimulatory molecules, upregulating the phosphorylation of p38, ERK 1/2, JNK, and NF-kB p65 and inducing the high production of IL-6, IL-8, and hyaluronan [24]. In different TAO stages, there are different T cell subset biases, and the skewed pattern of cytokine production in orbit, such as Th1 immune response predominated early active TAO, and Th2 immune response prevailed in late stable TAO [25]. Our study also found that T cell infiltration in TAO orbital tissues, Th1 and Th2 cell differentiation pathways, are involved in TAO pathogenesis. This aligns with the widely accepted concept that T-cell-mediated immunity contributes to TAO development. In addition, our study also found that innate immune system members, especially neutrophil cells and natural killer cells, are differentially expressed in the TAO orbital immune microenvironment. Due to many studies on T cells and adaptive immunity in TAO, this paper focuses on the relationship between TAO and innate immunity. We believe that both adaptive and innate immunity is involved in the pathophysiology of TAO, although the study of innate immunity is still in the exploratory stage.

Neutrophils are primary effector cells of innate immunity and fight infection by phagocytosis and degranulation. Therefore, in autoimmune diseases, activation and release of neutrophil extracellular traps (NETs) can be considered a source of autoantigens and may induce the formation of autoantibodies [26]. Recently, NETs have been proposed to play an essential role in several autoimmune diseases, such as RA and SLE, by externalizing intracellular neoepitopes, e.g., dsDNA and nuclear proteins in SLE and citrullinated peptides in RA [27]. In addition, DNA demethylation was validated to enhance spontaneous NETosis through increasing PAD4 expression and histone citrullination [28].

Previous studies reported that the active inflammatory phase of TAO was mediated by the innate immune system [29]. For example, the polymorphism of interleukin-17A, an important cytokine involved in innate immune responses, was strongly associated with GD susceptibility [30]. Similarly, increased PTX3, a component of the innate immune system, in orbital tissue and serum has been found in TAO [31]. Furthermore, it has been revealed that differential methylation status HLA -DPB1 and PDCD1LG2 genes played a role in developing autoimmune thyroiditis [32]. Thus, neutrophils and innate immune responses may participate in TAO development by converting methylation status in genes related to those processes.

We obtained two diagnostic markers by the machine learning algorithm, including *S100A11* and *NKD2*. qRT-PCR illustrated that compared with normal samples, the expression of S100A11 in TAO was significantly downregulated, and the expressions of NKD2 in TAO were significantly upregulated. GSEA analysis showed that *NKD2* participated in innate immune response in the mucosa and Wnt signaling pathways. Previous studies reported *NKD2* negatively regulated canonical Wnt signaling by binding Dishevelled [33, 34]. In addition, the Wnt signaling pathway was validated to control adipogenesis and myofibroblast formation in TAO [35]. Besides, *NKD2* has multiple biological functions: modulating cell homeostasis, preventing tumorigenesis, and promoting kidney fibrosis by inducing col1a1 expression [36]. Furthermore, recent studies have found a strong correlation between *NKD2* expression and proinflammatory cytokine production in effector T cells, which mediated stimulation-dependent ORAI1 trafficking to augment Ca2+ entry in T cells, has been found in recent studies [37, 38]. Therefore, methylation alteration of *NKD2* may influence adipogenesis and pathogenic effector T cell responses, modulating TAO development.

Enrichment analysis found that *S100A11* involved protein polyufmylation and pancreatic-mediated proteolysis. Previous studies demonstrated that *S100A11* played a crucial role in tumor and low-grade inflammation [39]. Increased *S100A11* expression was recently shown in RA and associated with disease activity, inflammation, and autoantibodies against citrullinated proteins [40]. Moreover, extracellular *S100A11* was shown to act via RAGE-dependent signaling to activate the p38 mitogen-activated protein kinase pathway and accelerate chondrocyte hypertrophy and matrix catabolism [41]. In addition, in vitro study revealed that extracellular *S100A11* augmented the inflammatory response by inducing proinflammatory cytokines in neutrophils [40]. The function of *S100A11* in TAO has yet to be thoroughly evaluated. Combining the present and previous results, we speculated that *S100A11* demethylation was a crucial immune regulator in TAO.

Meanwhile, our study has proven that innate immune system members, such as natural killer cells, dendritic cells, and macrophages, were essential constituents of the TAO orbital microenvironment [42]. Besides, both NKD2 and S100A11 expression strongly positively correlated with natural killer cell infiltration in TAO. Studies indicated NK-derived cytokines and their cytotoxic functions through induction of apoptosis took part in regulating the immune responses. They could contribute to the pathogenesis of many immune-mediated diseases, including Behcet’s disease and multiple sclerosis, RA, SLE, and type 1 diabetes [43]. But the precise mechanisms of NK cells in TAO progression are still largely unknown. Our study showed that the expression of NKD2 and S100A11 was closely related to immune infiltration, especially by innate immune cell infiltration in TAO.

However, several limitations to our study should be noted: firstly, our results were mainly based on the prediction of bioinformatics data and a small number of clinical samples; only seldom clinical stages, such as clinical activity score and thyroid antibody status of the patients whose samples were contained in the gene dataset, can be retrieved (Supplementary Table S11). In addition, TAO is a very heterogeneous disease; therefore, the expression of related genes might differ in patients based on disease severity, activity, and duration. So more detailed biologic mechanisms of the final selected markers remain to be investigated in laboratory and future clinical experiments. Second, this study was mainly based on DNA and mRNA expression on orbital tissues, and integrated characterization and analysis using transcriptomic, proteomic, and metabolomic molecular profiles could be more accurate.

## Conclusion

In short, this study identified *NKD2* and *S100A11* as two potential biomarkers for TAO. This study was the first clinical investigation of the association of *NKD2*, *S100A11*, and immune cell infiltration with TAO. Further studies are needed to elucidate their precise roles in this disease.

## Supplementary Information

Supplementary_Material revised

Supplementary material figure 1

Supplementary Figure 1 legend
