# Supplementary material for: Machine learning-based prediction of diagnostic markers for Graves’ orbitopathy
Source: Endocrine. 2023 Apr 15;81(2):277–89. doi: 10.1007/s12020-023-03349-z (PMC10293385; doi:10.1007/s12020-023-03349-z)
Supplement: Supplementary file 1 — Supplementary_Material revised [file 12020_2023_3349_MOESM1_ESM.docx]

Supplementary Material

## Supplementary Tables

**Supplementary Table S1.** Primer sequences for qRT-PCR.

| Primer | Sequence |
| --- | --- |
| S100A11 F | TCGAGTCCCTGATTGCTGTC |
| S100A11 R | CAAGCCATAGCTAGGCCACC |
| NKD2 F | GACAAGGCTAGAGGAGTCGG |
| NKD2 R | ACGGCATGTGTATCTGGCTC |
| GAPDH F | CGAAGGTGGAGTCAACGGATTT |
| GAPDH R | ATGGGTGGAATCATATTGGAAC |

**Supplementary Table S2.** 994 significant DEGs between TAO and normal samples.

| **Gene** | **logFC** | **AveExpr** | **t** | **P.Value** | **adj.P.Val** | **B** | **type** | **Gene** | **logFC** | **AveExpr** | **t** | **P.Value** | **adj.P.Val** | **B** | **type** |
| --- | --- | --- | --- | --- | --- | --- | --- | --- | --- | --- | --- | --- | --- | --- | --- |
| FOSB | 1.317 | 5.455 | 3.520 | 0.001 | 0.029 | -0.610 | up | SPOCK1 | -0.567 | 4.719 | -2.007 | 0.049 | 0.136 | -4.140 | down |
| FOS | 1.220 | 5.840 | 2.802 | 0.007 | 0.054 | -2.474 | up | DDOST | -0.567 | 7.239 | -3.138 | 0.003 | 0.040 | -1.640 | down |
| EGR1 | 0.962 | 6.099 | 2.920 | 0.005 | 0.048 | -2.190 | up | PRKACB | -0.568 | 4.722 | -2.222 | 0.030 | 0.103 | -3.735 | down |
| CHAD | 0.949 | 4.773 | 2.725 | 0.008 | 0.057 | -2.654 | up | RPL30 | -0.568 | 10.516 | -3.768 | 0.000 | 0.023 | 0.100 | down |
| CYR61 | 0.874 | 6.411 | 2.627 | 0.011 | 0.063 | -2.880 | up | SRPR | -0.568 | 7.774 | -2.501 | 0.015 | 0.073 | -3.158 | down |
| APOLD1 | 0.872 | 4.474 | 2.482 | 0.016 | 0.075 | -3.199 | up | SRSF6 | -0.568 | 6.695 | -2.710 | 0.009 | 0.058 | -2.690 | down |
| S100A12 | 0.845 | 2.818 | 3.581 | 0.001 | 0.027 | -0.439 | up | ARPP19 | -0.568 | 5.514 | -2.992 | 0.004 | 0.045 | -2.012 | down |
| ANGPTL7 | 0.804 | 4.454 | 3.144 | 0.002 | 0.040 | -1.625 | up | MARCKS | -0.569 | 6.107 | -2.878 | 0.005 | 0.050 | -2.291 | down |
| COL11A2 | 0.783 | 4.726 | 3.830 | 0.000 | 0.022 | 0.283 | up | SECISBP2L | -0.569 | 5.969 | -2.357 | 0.021 | 0.087 | -3.462 | down |
| IL6 | 0.765 | 4.451 | 3.160 | 0.002 | 0.039 | -1.582 | up | WIPI1 | -0.570 | 6.165 | -3.115 | 0.003 | 0.041 | -1.700 | down |
| S100A8 | 0.716 | 3.844 | 3.005 | 0.004 | 0.045 | -1.978 | up | LAP3 | -0.570 | 5.725 | -3.231 | 0.002 | 0.038 | -1.397 | down |
| CSRNP1 | 0.697 | 5.285 | 3.934 | 0.000 | 0.020 | 0.594 | up | FAM210B | -0.570 | 6.266 | -3.937 | 0.000 | 0.020 | 0.600 | down |
| LOC102724965 | 0.686 | 6.668 | 2.344 | 0.022 | 0.088 | -3.490 | up | H2BFS | -0.571 | 8.913 | -3.898 | 0.000 | 0.020 | 0.483 | down |
| IQCC | 0.686 | 5.260 | 2.934 | 0.005 | 0.047 | -2.154 | up | MYD88 | -0.571 | 6.037 | -3.132 | 0.003 | 0.040 | -1.654 | down |
| SCUBE1 | 0.682 | 7.160 | 2.798 | 0.007 | 0.054 | -2.483 | up | RCN1 | -0.571 | 7.759 | -3.588 | 0.001 | 0.027 | -0.420 | down |
| B3GNT8 | 0.682 | 4.999 | 3.279 | 0.002 | 0.036 | -1.269 | up | GNPNAT1 | -0.571 | 5.017 | -3.270 | 0.002 | 0.036 | -1.293 | down |
| FCGR3B | 0.682 | 1.896 | 3.430 | 0.001 | 0.031 | -0.859 | up | CLK1 | -0.571 | 5.657 | -2.484 | 0.015 | 0.075 | -3.194 | down |
| GPHA2 | 0.676 | 3.591 | 2.524 | 0.014 | 0.071 | -3.109 | up | SLC35A4 | -0.571 | 6.159 | -3.693 | 0.000 | 0.025 | -0.117 | down |
| DAPK3 | 0.660 | 4.158 | 3.092 | 0.003 | 0.042 | -1.759 | up | CEBPZOS | -0.572 | 4.444 | -3.667 | 0.000 | 0.025 | -0.192 | down |
| RP11-357G3.1 | 0.657 | 4.573 | 3.411 | 0.001 | 0.032 | -0.912 | up | CRNDE | -0.572 | 5.197 | -2.943 | 0.004 | 0.047 | -2.131 | down |
| ZNF793 | 0.657 | 4.779 | 2.960 | 0.004 | 0.046 | -2.090 | up | ZIC2 | -0.572 | 2.439 | -3.007 | 0.004 | 0.045 | -1.973 | down |
| S100A9 | 0.654 | 6.468 | 3.431 | 0.001 | 0.031 | -0.858 | up | ACLY | -0.573 | 7.166 | -3.954 | 0.000 | 0.020 | 0.653 | down |
| IRX1 | 0.654 | 5.921 | 3.010 | 0.004 | 0.044 | -1.965 | up | AMZ2 | -0.573 | 5.902 | -3.074 | 0.003 | 0.042 | -1.804 | down |
| CSF3R | 0.652 | 5.209 | 3.110 | 0.003 | 0.041 | -1.711 | up | JTB | -0.574 | 7.137 | -2.625 | 0.011 | 0.064 | -2.885 | down |
| C9orf172 | 0.647 | 6.228 | 2.688 | 0.009 | 0.059 | -2.741 | up | SRP14 | -0.574 | 9.098 | -2.934 | 0.005 | 0.047 | -2.154 | down |
| PRAM1 | 0.638 | 6.314 | 2.962 | 0.004 | 0.046 | -2.087 | up | CTSA | -0.574 | 7.619 | -3.967 | 0.000 | 0.020 | 0.690 | down |
| TREM1 | 0.636 | 3.488 | 3.685 | 0.000 | 0.025 | -0.142 | up | TTC37 | -0.575 | 4.265 | -3.161 | 0.002 | 0.039 | -1.580 | down |
| NAPSA | 0.635 | 4.890 | 3.154 | 0.002 | 0.039 | -1.598 | up | ARL8B | -0.575 | 6.192 | -2.881 | 0.005 | 0.050 | -2.284 | down |
| MAB21L2 | 0.631 | 2.488 | 2.969 | 0.004 | 0.046 | -2.069 | up | SNRPD2 | -0.576 | 8.691 | -3.943 | 0.000 | 0.020 | 0.618 | down |
| NEBL-AS1 | 0.630 | 5.641 | 3.218 | 0.002 | 0.038 | -1.431 | up | GPS2 | -0.576 | 7.460 | -3.698 | 0.000 | 0.025 | -0.104 | down |
| MGC10814 | 0.628 | 5.572 | 2.972 | 0.004 | 0.046 | -2.061 | up | CRLF3 | -0.577 | 4.020 | -2.478 | 0.016 | 0.075 | -3.207 | down |
| NR4A1 | 0.626 | 6.754 | 3.010 | 0.004 | 0.044 | -1.966 | up | MAGT1 | -0.578 | 4.775 | -3.197 | 0.002 | 0.039 | -1.485 | down |
| CASC14 | 0.626 | 6.458 | 2.193 | 0.032 | 0.106 | -3.792 | up | MS4A7 | -0.578 | 5.289 | -2.490 | 0.015 | 0.074 | -3.181 | down |
| TRIM17 | 0.623 | 5.419 | 3.248 | 0.002 | 0.037 | -1.351 | up | CAP1 | -0.578 | 7.224 | -3.511 | 0.001 | 0.029 | -0.634 | down |
| ZBTB32 | 0.621 | 5.498 | 2.457 | 0.017 | 0.077 | -3.252 | up | MGLL | -0.578 | 8.291 | -4.325 | 0.000 | 0.015 | 1.801 | down |
| SOX21 | 0.617 | 5.442 | 2.751 | 0.008 | 0.056 | -2.593 | up | ADAMTS5 | -0.579 | 3.978 | -2.138 | 0.036 | 0.114 | -3.897 | down |
| PLA2G4F | 0.617 | 5.740 | 2.541 | 0.013 | 0.070 | -3.072 | up | DAD1 | -0.579 | 7.349 | -3.104 | 0.003 | 0.041 | -1.727 | down |
| RP11-400N9.1 | 0.615 | 3.980 | 3.149 | 0.002 | 0.040 | -1.612 | up | MYCBP2 | -0.579 | 7.042 | -3.036 | 0.003 | 0.043 | -1.901 | down |
| CDRT15L2 | 0.612 | 3.871 | 2.673 | 0.009 | 0.060 | -2.774 | up | GGTA1P | -0.579 | 6.530 | -2.813 | 0.006 | 0.053 | -2.447 | down |
| TCAP | 0.611 | 6.403 | 2.717 | 0.008 | 0.058 | -2.674 | up | TRIM21 | -0.579 | 5.898 | -3.697 | 0.000 | 0.025 | -0.106 | down |
| COMP | 0.611 | 4.856 | 3.897 | 0.000 | 0.020 | 0.482 | up | PAPSS1 | -0.579 | 5.894 | -2.689 | 0.009 | 0.059 | -2.739 | down |
| THBS1 | 0.607 | 4.557 | 2.455 | 0.017 | 0.077 | -3.257 | up | TMEM30A | -0.580 | 5.699 | -3.210 | 0.002 | 0.039 | -1.453 | down |
| LOC286058 | 0.605 | 2.674 | 3.334 | 0.001 | 0.034 | -1.123 | up | SLC40A1 | -0.580 | 4.380 | -4.026 | 0.000 | 0.019 | 0.870 | down |
| FAM109A | 0.605 | 6.633 | 2.702 | 0.009 | 0.059 | -2.707 | up | MAP3K1 | -0.580 | 5.278 | -3.531 | 0.001 | 0.028 | -0.578 | down |
| PTPRH | 0.602 | 4.952 | 2.839 | 0.006 | 0.052 | -2.386 | up | TOMM5 | -0.580 | 6.653 | -3.611 | 0.001 | 0.026 | -0.354 | down |
| RP11-401P9.4 | 0.601 | 4.784 | 3.753 | 0.000 | 0.023 | 0.057 | up | UHMK1 | -0.580 | 5.531 | -2.963 | 0.004 | 0.046 | -2.083 | down |
| AC008753.4 | 0.600 | 5.923 | 2.821 | 0.006 | 0.053 | -2.429 | up | DEGS1 | -0.580 | 5.353 | -3.538 | 0.001 | 0.028 | -0.561 | down |
| LOC100128644 | 0.593 | 6.187 | 2.855 | 0.006 | 0.051 | -2.347 | up | NGFRAP1 | -0.580 | 7.801 | -3.540 | 0.001 | 0.028 | -0.553 | down |
| RP11-272D12.1 | 0.591 | 6.610 | 2.405 | 0.019 | 0.082 | -3.363 | up | ECHDC1 | -0.580 | 6.709 | -3.184 | 0.002 | 0.039 | -1.521 | down |
| SIK1 | 0.589 | 5.960 | 3.356 | 0.001 | 0.034 | -1.063 | up | PSMA1 | -0.581 | 6.042 | -3.305 | 0.002 | 0.035 | -1.199 | down |
| PNMT | 0.586 | 5.469 | 2.814 | 0.006 | 0.053 | -2.446 | up | BZW2 | -0.581 | 5.469 | -3.847 | 0.000 | 0.021 | 0.334 | down |
| GADD45G | 0.585 | 5.218 | 4.546 | 0.000 | 0.011 | 2.510 | up | CD47 | -0.582 | 5.520 | -3.079 | 0.003 | 0.042 | -1.791 | down |
| FAM110D | 0.584 | 4.891 | 3.336 | 0.001 | 0.034 | -1.118 | up | MRPS6 | -0.582 | 6.464 | -3.225 | 0.002 | 0.038 | -1.413 | down |
| PITX3 | 0.581 | 5.325 | 2.481 | 0.016 | 0.075 | -3.201 | up | NCK2 | -0.582 | 7.064 | -2.589 | 0.012 | 0.066 | -2.964 | down |
| TMEM262 | 0.580 | 5.682 | 2.571 | 0.012 | 0.067 | -3.003 | up | SKP1 | -0.582 | 6.613 | -2.998 | 0.004 | 0.045 | -1.996 | down |
| GRASPOS | 0.579 | 4.133 | 4.159 | 0.000 | 0.016 | 1.279 | up | CCDC86 | -0.583 | 5.953 | -3.498 | 0.001 | 0.029 | -0.672 | down |
| CXCR2 | 0.579 | 3.951 | 2.839 | 0.006 | 0.052 | -2.385 | up | CREBL2 | -0.583 | 5.973 | -3.529 | 0.001 | 0.028 | -0.586 | down |
| STAC3 | 0.577 | 6.614 | 2.709 | 0.009 | 0.058 | -2.693 | up | ZNF275 | -0.583 | 5.725 | -3.186 | 0.002 | 0.039 | -1.515 | down |
| CPNE9 | 0.573 | 4.829 | 2.928 | 0.005 | 0.048 | -2.169 | up | C3 | -0.583 | 9.473 | -3.079 | 0.003 | 0.042 | -1.792 | down |
| F2 | 0.573 | 4.614 | 2.837 | 0.006 | 0.052 | -2.391 | up | CTSS | -0.584 | 5.273 | -3.157 | 0.002 | 0.039 | -1.592 | down |
| CRLF1 | 0.572 | 5.340 | 3.071 | 0.003 | 0.042 | -1.811 | up | ASAP2 | -0.584 | 3.636 | -2.423 | 0.018 | 0.080 | -3.326 | down |
| GKN1 | 0.570 | 2.997 | 3.723 | 0.000 | 0.024 | -0.032 | up | MFSD1 | -0.584 | 5.058 | -2.786 | 0.007 | 0.054 | -2.512 | down |
| S100P | 0.570 | 4.710 | 3.252 | 0.002 | 0.037 | -1.340 | up | PABPC3 | -0.584 | 8.874 | -2.234 | 0.029 | 0.101 | -3.711 | down |
| LIM2 | 0.570 | 4.503 | 2.585 | 0.012 | 0.066 | -2.972 | up | EIF2S3 | -0.584 | 7.154 | -3.174 | 0.002 | 0.039 | -1.546 | down |
| FAM53A | 0.569 | 5.052 | 3.243 | 0.002 | 0.037 | -1.366 | up | BC022047 | -0.584 | 5.722 | -2.249 | 0.028 | 0.099 | -3.682 | down |
| PNPLA5 | 0.568 | 4.256 | 3.646 | 0.001 | 0.026 | -0.252 | up | LMAN1 | -0.585 | 5.512 | -3.184 | 0.002 | 0.039 | -1.520 | down |
| AC006538.1 | 0.568 | 3.213 | 2.793 | 0.007 | 0.054 | -2.496 | up | NCOA1 | -0.586 | 6.654 | -3.560 | 0.001 | 0.028 | -0.499 | down |
| GCK | 0.565 | 3.733 | 3.625 | 0.001 | 0.026 | -0.314 | up | MOB1A | -0.586 | 4.838 | -2.894 | 0.005 | 0.049 | -2.252 | down |
| LOC101060004 | 0.565 | 4.560 | 3.515 | 0.001 | 0.029 | -0.625 | up | RRAGA | -0.586 | 7.021 | -2.942 | 0.004 | 0.047 | -2.136 | down |
| MYOG | 0.564 | 4.130 | 2.712 | 0.008 | 0.058 | -2.684 | up | VAMP3 | -0.587 | 7.601 | -2.773 | 0.007 | 0.055 | -2.543 | down |
| LYG2 | 0.563 | 3.418 | 2.404 | 0.019 | 0.082 | -3.365 | up | EBLN3 | -0.587 | 5.090 | -2.844 | 0.006 | 0.051 | -2.372 | down |
| TNNI1 | 0.563 | 4.522 | 2.649 | 0.010 | 0.062 | -2.830 | up | LDOC1 | -0.587 | 7.487 | -2.764 | 0.007 | 0.055 | -2.563 | down |
| PGLYRP2 | 0.563 | 4.557 | 3.231 | 0.002 | 0.038 | -1.396 | up | PTGES3 | -0.587 | 6.691 | -2.579 | 0.012 | 0.067 | -2.987 | down |
| SOCS3 | 0.562 | 5.675 | 3.016 | 0.004 | 0.044 | -1.950 | up | NINJ1 | -0.588 | 6.419 | -4.647 | 0.000 | 0.009 | 2.840 | down |
| FCN1 | 0.562 | 5.748 | 3.734 | 0.000 | 0.024 | 0.001 | up | ACVR1 | -0.588 | 4.348 | -2.708 | 0.009 | 0.058 | -2.694 | down |
| PERM1 | 0.561 | 7.279 | 2.440 | 0.017 | 0.079 | -3.289 | up | MFF | -0.588 | 5.879 | -3.088 | 0.003 | 0.042 | -1.770 | down |
| SFRP5 | 0.561 | 5.329 | 3.039 | 0.003 | 0.043 | -1.892 | up | SH2B3 | -0.589 | 6.118 | -2.815 | 0.006 | 0.053 | -2.443 | down |
| PPFIA3 | 0.561 | 5.555 | 2.702 | 0.009 | 0.059 | -2.709 | up | SUSD1 | -0.589 | 3.914 | -5.264 | 0.000 | 0.006 | 4.922 | down |
| LOC100996246 | 0.561 | 4.798 | 2.817 | 0.006 | 0.053 | -2.439 | up | APOL6 | -0.589 | 5.879 | -3.335 | 0.001 | 0.034 | -1.119 | down |
| LOC728061 | 0.560 | 5.128 | 3.767 | 0.000 | 0.023 | 0.098 | up | BZW1 | -0.590 | 6.541 | -2.782 | 0.007 | 0.054 | -2.522 | down |
| LOC151174 | 0.559 | 4.486 | 3.097 | 0.003 | 0.042 | -1.745 | up | OST4 | -0.590 | 8.973 | -3.344 | 0.001 | 0.034 | -1.095 | down |
| ATF3 | 0.559 | 4.849 | 3.477 | 0.001 | 0.030 | -0.731 | up | PTP4A2 | -0.591 | 7.477 | -3.149 | 0.002 | 0.040 | -1.611 | down |
| FOSL1 | 0.557 | 5.018 | 3.070 | 0.003 | 0.042 | -1.815 | up | S100A11 | -0.592 | 9.041 | -3.797 | 0.000 | 0.022 | 0.185 | down |
| LOC403323 | 0.556 | 5.110 | 2.707 | 0.009 | 0.058 | -2.696 | up | DHCR24 | -0.592 | 6.105 | -3.391 | 0.001 | 0.032 | -0.966 | down |
| RP5-968J1.1 | 0.554 | 5.001 | 2.649 | 0.010 | 0.062 | -2.829 | up | GNPAT | -0.592 | 4.595 | -2.380 | 0.020 | 0.085 | -3.416 | down |
| KMT2E-AS1 | 0.554 | 6.091 | 2.580 | 0.012 | 0.067 | -2.984 | up | AZIN1 | -0.592 | 4.429 | -2.998 | 0.004 | 0.045 | -1.996 | down |
| WTIP | 0.553 | 5.947 | 2.886 | 0.005 | 0.049 | -2.271 | up | RAB8A | -0.592 | 6.775 | -3.478 | 0.001 | 0.029 | -0.727 | down |
| PDIA2 | 0.553 | 4.952 | 2.856 | 0.006 | 0.051 | -2.344 | up | PDCD6IP | -0.592 | 6.112 | -3.128 | 0.003 | 0.040 | -1.667 | down |
| FAM132A | 0.553 | 4.356 | 2.701 | 0.009 | 0.059 | -2.711 | up | GNPDA2 | -0.592 | 3.797 | -3.860 | 0.000 | 0.021 | 0.371 | down |
| ATP4A | 0.553 | 4.751 | 3.016 | 0.004 | 0.044 | -1.951 | up | HIGD1A | -0.592 | 5.144 | -3.182 | 0.002 | 0.039 | -1.527 | down |
| SEMA3B | 0.553 | 4.277 | 2.822 | 0.006 | 0.053 | -2.426 | up | HIAT1 | -0.593 | 4.975 | -2.896 | 0.005 | 0.049 | -2.247 | down |
| ADCY8 | 0.553 | 4.576 | 2.352 | 0.022 | 0.088 | -3.473 | up | KIAA1033 | -0.593 | 5.907 | -3.045 | 0.003 | 0.043 | -1.877 | down |
| LOC401913 | 0.552 | 4.041 | 2.457 | 0.017 | 0.077 | -3.253 | up | UBE2E1 | -0.594 | 5.414 | -3.001 | 0.004 | 0.045 | -1.989 | down |
| GRASP | 0.552 | 7.099 | 2.704 | 0.009 | 0.059 | -2.704 | up | SARAF | -0.595 | 6.681 | -2.477 | 0.016 | 0.075 | -3.210 | down |
| NR4A3 | 0.552 | 3.954 | 2.697 | 0.009 | 0.059 | -2.719 | up | DYNLL1 | -0.595 | 7.539 | -2.915 | 0.005 | 0.048 | -2.202 | down |
| CEP83-AS1 | 0.551 | 4.806 | 2.805 | 0.007 | 0.053 | -2.466 | up | NPTN | -0.595 | 5.067 | -2.369 | 0.021 | 0.086 | -3.438 | down |
| SPRED3 | 0.548 | 7.199 | 2.538 | 0.013 | 0.070 | -3.077 | up | MXI1 | -0.595 | 6.294 | -2.387 | 0.020 | 0.084 | -3.401 | down |
| LOC283335 | 0.548 | 5.846 | 2.509 | 0.015 | 0.073 | -3.142 | up | SLC35A1 | -0.595 | 4.289 | -2.594 | 0.012 | 0.066 | -2.953 | down |
| GUCA2A | 0.548 | 4.920 | 2.909 | 0.005 | 0.048 | -2.216 | up | LDOC1L | -0.595 | 5.987 | -2.826 | 0.006 | 0.052 | -2.416 | down |
| LDHC | 0.548 | 4.163 | 3.335 | 0.001 | 0.034 | -1.120 | up | FAM127A | -0.596 | 8.263 | -3.006 | 0.004 | 0.045 | -1.977 | down |
| JSRP1 | 0.547 | 6.962 | 2.823 | 0.006 | 0.053 | -2.425 | up | IRX3 | -0.596 | 5.309 | -2.057 | 0.044 | 0.128 | -4.048 | down |
| KRT85 | 0.547 | 3.584 | 3.441 | 0.001 | 0.031 | -0.829 | up | CLCN3 | -0.596 | 5.490 | -3.025 | 0.004 | 0.044 | -1.928 | down |
| DUSP21 | 0.546 | 3.366 | 3.826 | 0.000 | 0.022 | 0.271 | up | TNFRSF21 | -0.597 | 4.928 | -3.690 | 0.000 | 0.025 | -0.128 | down |
| CXCL8 | 0.546 | 3.333 | 3.429 | 0.001 | 0.031 | -0.863 | up | R3HDM2 | -0.598 | 6.896 | -3.063 | 0.003 | 0.043 | -1.833 | down |
| PRR15 | 0.545 | 2.994 | 3.602 | 0.001 | 0.027 | -0.379 | up | EPHX1 | -0.598 | 8.651 | -2.676 | 0.009 | 0.060 | -2.768 | down |
| LOC100129516 | 0.542 | 5.745 | 2.378 | 0.020 | 0.085 | -3.419 | up | APEX1 | -0.598 | 7.556 | -5.061 | 0.000 | 0.007 | 4.224 | down |
| KRMP1 | 0.542 | 4.415 | 2.737 | 0.008 | 0.057 | -2.627 | up | RASA1 | -0.598 | 4.738 | -2.574 | 0.012 | 0.067 | -2.999 | down |
| BARX1 | 0.542 | 5.155 | 3.200 | 0.002 | 0.039 | -1.480 | up | TDP2 | -0.598 | 5.340 | -3.052 | 0.003 | 0.043 | -1.859 | down |
| FOXB1 | 0.541 | 4.055 | 3.388 | 0.001 | 0.033 | -0.977 | up | ZRANB2 | -0.598 | 6.925 | -3.207 | 0.002 | 0.039 | -1.459 | down |
| LOC102724611 | 0.540 | 2.972 | 3.636 | 0.001 | 0.026 | -0.281 | up | TOMM20 | -0.599 | 6.562 | -2.970 | 0.004 | 0.046 | -2.065 | down |
| ICAM4 | 0.540 | 3.158 | 4.594 | 0.000 | 0.010 | 2.665 | up | MCUR1 | -0.599 | 4.794 | -4.173 | 0.000 | 0.016 | 1.324 | down |
| RP11-517C16.2 | 0.540 | 4.727 | 2.564 | 0.013 | 0.068 | -3.021 | up | NT5C2 | -0.599 | 6.654 | -2.966 | 0.004 | 0.046 | -2.076 | down |
| TBC1D29 | 0.540 | 4.630 | 2.855 | 0.006 | 0.051 | -2.346 | up | CD46 | -0.600 | 7.012 | -2.549 | 0.013 | 0.069 | -3.053 | down |
| OPRD1 | 0.538 | 5.028 | 2.503 | 0.015 | 0.073 | -3.153 | up | SMIM15 | -0.600 | 5.422 | -2.326 | 0.023 | 0.090 | -3.526 | down |
| IGFALS | 0.538 | 5.925 | 2.702 | 0.009 | 0.059 | -2.707 | up | GALNT12 | -0.600 | 4.495 | -3.775 | 0.000 | 0.023 | 0.120 | down |
| SHANK1 | 0.537 | 5.430 | 3.164 | 0.002 | 0.039 | -1.572 | up | RBM4 | -0.600 | 3.894 | -3.075 | 0.003 | 0.042 | -1.802 | down |
| EXTL1 | 0.537 | 5.112 | 3.038 | 0.003 | 0.043 | -1.897 | up | TMEM214 | -0.600 | 7.657 | -3.340 | 0.001 | 0.034 | -1.105 | down |
| EBI3 | 0.537 | 5.824 | 3.060 | 0.003 | 0.043 | -1.839 | up | KARS | -0.601 | 6.965 | -3.493 | 0.001 | 0.029 | -0.685 | down |
| TGM2 | 0.537 | 4.596 | 3.801 | 0.000 | 0.022 | 0.196 | up | LAPTM4A | -0.601 | 7.866 | -3.201 | 0.002 | 0.039 | -1.474 | down |
| RHOV | 0.537 | 6.519 | 3.250 | 0.002 | 0.037 | -1.347 | up | MEA1 | -0.601 | 7.775 | -4.210 | 0.000 | 0.016 | 1.438 | down |
| NEK10 | 0.536 | 3.993 | 2.987 | 0.004 | 0.045 | -2.024 | up | CIRH1A | -0.601 | 6.442 | -3.960 | 0.000 | 0.020 | 0.672 | down |
| NT5C1A | 0.535 | 3.308 | 4.966 | 0.000 | 0.007 | 3.903 | up | AXL | -0.601 | 6.556 | -2.636 | 0.010 | 0.063 | -2.859 | down |
| CYP11B2 | 0.535 | 6.223 | 2.606 | 0.011 | 0.065 | -2.927 | up | TRAM1 | -0.602 | 6.123 | -3.215 | 0.002 | 0.038 | -1.438 | down |
| HOXC12 | 0.535 | 4.068 | 2.094 | 0.040 | 0.122 | -3.981 | up | FAM35A | -0.602 | 4.826 | -3.359 | 0.001 | 0.034 | -1.054 | down |
| APLN | 0.535 | 5.268 | 2.896 | 0.005 | 0.049 | -2.247 | up | CDC16 | -0.602 | 5.972 | -3.967 | 0.000 | 0.020 | 0.692 | down |
| TCF15 | 0.534 | 6.801 | 2.495 | 0.015 | 0.074 | -3.172 | up | TSTD1 | -0.602 | 6.287 | -2.265 | 0.027 | 0.097 | -3.650 | down |
| REEP2 | 0.534 | 5.268 | 3.295 | 0.002 | 0.036 | -1.226 | up | NDUFA4 | -0.602 | 8.232 | -3.476 | 0.001 | 0.030 | -0.732 | down |
| TNNC1 | 0.532 | 4.499 | 3.174 | 0.002 | 0.039 | -1.547 | up | TPRKB | -0.602 | 4.932 | -2.917 | 0.005 | 0.048 | -2.196 | down |
| DKFZP434A062 | 0.532 | 5.251 | 3.442 | 0.001 | 0.031 | -0.828 | up | LYPLA1 | -0.602 | 3.984 | -3.070 | 0.003 | 0.042 | -1.815 | down |
| NANOS3 | 0.532 | 5.150 | 3.066 | 0.003 | 0.042 | -1.824 | up | PIAS1 | -0.602 | 5.548 | -2.913 | 0.005 | 0.048 | -2.205 | down |
| ALDH3A1 | 0.531 | 5.074 | 2.724 | 0.008 | 0.057 | -2.657 | up | MRPS21 | -0.603 | 7.204 | -3.337 | 0.001 | 0.034 | -1.115 | down |
| MGC4294 | 0.530 | 4.523 | 2.492 | 0.015 | 0.074 | -3.177 | up | RHOBTB3 | -0.603 | 5.591 | -3.535 | 0.001 | 0.028 | -0.568 | down |
| EPX | 0.530 | 3.575 | 3.321 | 0.001 | 0.035 | -1.158 | up | LOXL1 | -0.603 | 6.733 | -2.998 | 0.004 | 0.045 | -1.996 | down |
| DHDH | 0.530 | 4.448 | 2.784 | 0.007 | 0.054 | -2.517 | up | FUT8 | -0.603 | 4.857 | -2.986 | 0.004 | 0.045 | -2.026 | down |
| PPP1R32 | 0.529 | 6.063 | 3.057 | 0.003 | 0.043 | -1.847 | up | THOC7 | -0.603 | 4.110 | -2.228 | 0.029 | 0.102 | -3.723 | down |
| MED9 | 0.529 | 4.898 | 2.730 | 0.008 | 0.057 | -2.644 | up | TMBIM6 | -0.605 | 8.433 | -4.182 | 0.000 | 0.016 | 1.349 | down |
| TMEM59L | 0.529 | 5.972 | 2.801 | 0.007 | 0.054 | -2.477 | up | CYBRD1 | -0.605 | 7.779 | -2.039 | 0.045 | 0.131 | -4.082 | down |
| IQCF5 | 0.528 | 4.481 | 3.179 | 0.002 | 0.039 | -1.534 | up | GORASP2 | -0.606 | 6.744 | -3.019 | 0.004 | 0.044 | -1.944 | down |
| TMEM102 | 0.528 | 5.184 | 2.624 | 0.011 | 0.064 | -2.886 | up | GNG5 | -0.606 | 8.482 | -3.165 | 0.002 | 0.039 | -1.571 | down |
| RRP9 | 0.527 | 6.740 | 2.827 | 0.006 | 0.052 | -2.414 | up | IRF2BPL | -0.606 | 7.098 | -2.894 | 0.005 | 0.049 | -2.253 | down |
| KHDC3L | 0.527 | 5.329 | 3.039 | 0.003 | 0.043 | -1.894 | up | LINC00998 | -0.607 | 4.376 | -2.739 | 0.008 | 0.057 | -2.623 | down |
| ADAD2 | 0.527 | 5.957 | 2.533 | 0.014 | 0.071 | -3.088 | up | RTN3 | -0.607 | 7.400 | -4.475 | 0.000 | 0.012 | 2.280 | down |
| ASIC4 | 0.526 | 4.349 | 3.050 | 0.003 | 0.043 | -1.866 | up | SH3BGRL | -0.608 | 6.866 | -2.806 | 0.007 | 0.053 | -2.465 | down |
| DKFZp779M0652 | 0.525 | 4.893 | 2.942 | 0.004 | 0.047 | -2.136 | up | PTDSS1 | -0.608 | 5.845 | -3.500 | 0.001 | 0.029 | -0.667 | down |
| ACTL8 | 0.525 | 4.493 | 2.988 | 0.004 | 0.045 | -2.020 | up | UBQLN1 | -0.608 | 5.709 | -2.962 | 0.004 | 0.046 | -2.085 | down |
| FGF3 | 0.523 | 4.822 | 3.021 | 0.004 | 0.044 | -1.938 | up | TCF7L1 | -0.609 | 7.190 | -3.149 | 0.002 | 0.040 | -1.610 | down |
| GLTPD2 | 0.521 | 5.909 | 2.717 | 0.008 | 0.058 | -2.674 | up | TOMM70A | -0.609 | 5.807 | -3.473 | 0.001 | 0.030 | -0.741 | down |
| RNF151 | 0.521 | 4.566 | 2.751 | 0.008 | 0.056 | -2.595 | up | RPN2 | -0.609 | 7.232 | -3.033 | 0.003 | 0.043 | -1.909 | down |
| KIAA0319 | 0.521 | 6.214 | 2.615 | 0.011 | 0.064 | -2.907 | up | CDV3 | -0.609 | 8.118 | -3.580 | 0.001 | 0.027 | -0.440 | down |
| TNFRSF10C | 0.521 | 4.983 | 3.892 | 0.000 | 0.021 | 0.466 | up | HPGDS | -0.609 | 5.003 | -2.912 | 0.005 | 0.048 | -2.208 | down |
| IL34 | 0.521 | 7.647 | 2.435 | 0.018 | 0.079 | -3.301 | up | VBP1 | -0.610 | 5.337 | -2.996 | 0.004 | 0.045 | -2.000 | down |
| TNNI2 | 0.520 | 5.188 | 2.310 | 0.024 | 0.092 | -3.559 | up | SFRP1 | -0.611 | 7.672 | -2.409 | 0.019 | 0.082 | -3.354 | down |
| TMEM151A | 0.519 | 5.193 | 2.271 | 0.026 | 0.096 | -3.638 | up | MAF | -0.611 | 5.299 | -3.494 | 0.001 | 0.029 | -0.683 | down |
| FGF21 | 0.518 | 5.171 | 3.356 | 0.001 | 0.034 | -1.062 | up | ATP5C1 | -0.612 | 6.923 | -3.821 | 0.000 | 0.022 | 0.257 | down |
| CDH16 | 0.518 | 4.059 | 3.685 | 0.000 | 0.025 | -0.142 | up | SESTD1 | -0.612 | 6.529 | -3.115 | 0.003 | 0.041 | -1.700 | down |
| RHCG | 0.517 | 4.654 | 2.574 | 0.012 | 0.067 | -2.998 | up | CD34 | -0.612 | 8.080 | -2.171 | 0.034 | 0.109 | -3.835 | down |
| PRSS35 | 0.517 | 2.532 | 4.515 | 0.000 | 0.012 | 2.410 | up | AFF1 | -0.612 | 7.877 | -3.380 | 0.001 | 0.033 | -0.997 | down |
| FUT7 | 0.517 | 5.691 | 2.595 | 0.012 | 0.066 | -2.950 | up | IQGAP1 | -0.612 | 7.866 | -3.273 | 0.002 | 0.036 | -1.286 | down |
| GPATCH3 | 0.517 | 4.297 | 3.055 | 0.003 | 0.043 | -1.852 | up | NXT2 | -0.612 | 4.114 | -3.830 | 0.000 | 0.022 | 0.282 | down |
| PRR7 | 0.517 | 4.782 | 2.755 | 0.008 | 0.056 | -2.585 | up | LYRM5 | -0.613 | 4.831 | -2.452 | 0.017 | 0.078 | -3.264 | down |
| C17orf53 | 0.516 | 5.519 | 3.087 | 0.003 | 0.042 | -1.772 | up | PSMG2 | -0.613 | 7.076 | -3.086 | 0.003 | 0.042 | -1.773 | down |
| LOC102724975 | 0.516 | 5.498 | 2.428 | 0.018 | 0.080 | -3.315 | up | UCHL3 | -0.613 | 4.912 | -2.917 | 0.005 | 0.048 | -2.196 | down |
| FBXL19 | 0.516 | 6.112 | 2.399 | 0.019 | 0.083 | -3.375 | up | GINM1 | -0.614 | 6.700 | -3.404 | 0.001 | 0.032 | -0.931 | down |
| FAM99B | 0.516 | 6.460 | 3.100 | 0.003 | 0.041 | -1.739 | up | MRPS33 | -0.614 | 5.796 | -3.109 | 0.003 | 0.041 | -1.716 | down |
| TM4SF5 | 0.516 | 5.221 | 2.379 | 0.020 | 0.085 | -3.418 | up | CAPNS1 | -0.614 | 9.335 | -3.839 | 0.000 | 0.022 | 0.309 | down |
| LOC100506271 | 0.516 | 3.948 | 2.870 | 0.005 | 0.050 | -2.311 | up | DYNLT3 | -0.614 | 6.718 | -2.529 | 0.014 | 0.071 | -3.097 | down |
| XAGE3 | 0.515 | 3.964 | 3.912 | 0.000 | 0.020 | 0.525 | up | COPZ1 | -0.615 | 6.280 | -3.588 | 0.001 | 0.027 | -0.419 | down |
| HBM | 0.515 | 5.355 | 3.197 | 0.002 | 0.039 | -1.486 | up | HDGF | -0.615 | 8.126 | -3.998 | 0.000 | 0.020 | 0.784 | down |
| DNAJB8 | 0.515 | 4.458 | 2.817 | 0.006 | 0.053 | -2.439 | up | POLR2I | -0.615 | 8.128 | -3.258 | 0.002 | 0.037 | -1.325 | down |
| C14orf80 | 0.515 | 6.696 | 2.667 | 0.010 | 0.061 | -2.787 | up | PTER | -0.616 | 3.993 | -3.553 | 0.001 | 0.028 | -0.517 | down |
| C19orf81 | 0.515 | 4.574 | 2.410 | 0.019 | 0.081 | -3.353 | up | EXOC1 | -0.617 | 4.594 | -2.875 | 0.005 | 0.050 | -2.299 | down |
| SPN | 0.515 | 5.706 | 2.563 | 0.013 | 0.068 | -3.023 | up | LINC00657 | -0.617 | 6.151 | -2.822 | 0.006 | 0.053 | -2.425 | down |
| BMP2 | 0.514 | 3.909 | 3.438 | 0.001 | 0.031 | -0.839 | up | DEK | -0.617 | 6.655 | -2.377 | 0.020 | 0.085 | -3.422 | down |
| PLCH2 | 0.514 | 6.815 | 2.993 | 0.004 | 0.045 | -2.008 | up | MGST1 | -0.618 | 6.261 | -2.345 | 0.022 | 0.088 | -3.487 | down |
| WNT8B | 0.514 | 5.165 | 2.485 | 0.015 | 0.075 | -3.193 | up | SSR1 | -0.618 | 6.226 | -3.068 | 0.003 | 0.042 | -1.820 | down |
| TRABD2B | 0.514 | 4.793 | 4.990 | 0.000 | 0.007 | 3.983 | up | ATG14 | -0.619 | 5.211 | -3.184 | 0.002 | 0.039 | -1.521 | down |
| PINX1 | 0.513 | 4.506 | 3.313 | 0.001 | 0.035 | -1.179 | up | IL13RA1 | -0.619 | 6.056 | -3.065 | 0.003 | 0.042 | -1.826 | down |
| GLTSCR2 | 0.513 | 6.664 | 2.843 | 0.006 | 0.051 | -2.375 | up | RHOQ | -0.620 | 5.640 | -4.036 | 0.000 | 0.019 | 0.901 | down |
| GPR144 | 0.513 | 5.761 | 2.418 | 0.018 | 0.081 | -3.336 | up | NSA2 | -0.620 | 7.547 | -2.795 | 0.007 | 0.054 | -2.490 | down |
| NRIP2 | 0.513 | 4.744 | 3.496 | 0.001 | 0.029 | -0.677 | up | FCGR2B | -0.620 | 6.082 | -2.800 | 0.007 | 0.054 | -2.478 | down |
| DUSP1 | 0.512 | 7.121 | 2.457 | 0.017 | 0.077 | -3.253 | up | PFKFB3 | -0.621 | 7.199 | -2.145 | 0.036 | 0.113 | -3.884 | down |
| CCDC71 | 0.512 | 6.069 | 2.772 | 0.007 | 0.055 | -2.546 | up | PPAP2A | -0.621 | 6.766 | -2.594 | 0.012 | 0.066 | -2.953 | down |
| FNDC8 | 0.511 | 5.047 | 2.667 | 0.010 | 0.061 | -2.787 | up | RAP1B | -0.622 | 5.980 | -3.006 | 0.004 | 0.045 | -1.976 | down |
| FOXJ1 | 0.511 | 4.505 | 2.590 | 0.012 | 0.066 | -2.962 | up | ENOPH1 | -0.622 | 3.432 | -2.734 | 0.008 | 0.057 | -2.635 | down |
| INS | 0.511 | 3.856 | 3.133 | 0.003 | 0.040 | -1.654 | up | CD209 | -0.623 | 6.259 | -2.711 | 0.009 | 0.058 | -2.688 | down |
| DLL4 | 0.511 | 4.801 | 2.347 | 0.022 | 0.088 | -3.484 | up | APMAP | -0.624 | 7.720 | -3.492 | 0.001 | 0.029 | -0.688 | down |
| LOC101059948 | 0.510 | 4.422 | 3.724 | 0.000 | 0.024 | -0.028 | up | COX6C | -0.624 | 8.334 | -4.103 | 0.000 | 0.018 | 1.107 | down |
| LOC102724094 | 0.510 | 6.126 | 2.837 | 0.006 | 0.052 | -2.390 | up | STAU1 | -0.625 | 6.405 | -3.368 | 0.001 | 0.033 | -1.029 | down |
| RPS6KL1 | 0.509 | 5.044 | 2.490 | 0.015 | 0.074 | -3.182 | up | GPR146 | -0.626 | 6.641 | -5.299 | 0.000 | 0.006 | 5.045 | down |
| ADAMTSL2 | 0.509 | 6.083 | 2.292 | 0.025 | 0.094 | -3.595 | up | SAT1 | -0.626 | 7.371 | -3.187 | 0.002 | 0.039 | -1.511 | down |
| DKKL1 | 0.509 | 5.574 | 2.345 | 0.022 | 0.088 | -3.488 | up | PPIL3 | -0.627 | 5.491 | -2.729 | 0.008 | 0.057 | -2.645 | down |
| GPR97 | 0.509 | 3.776 | 3.685 | 0.000 | 0.025 | -0.141 | up | EIF2AK2 | -0.627 | 6.187 | -2.760 | 0.007 | 0.055 | -2.572 | down |
| CTC-360P9.3 | 0.508 | 4.215 | 2.616 | 0.011 | 0.064 | -2.904 | up | PBX1 | -0.627 | 7.620 | -3.262 | 0.002 | 0.037 | -1.314 | down |
| LOC399884 | 0.508 | 6.033 | 2.716 | 0.008 | 0.058 | -2.676 | up | LEPROT | -0.627 | 5.617 | -3.695 | 0.000 | 0.025 | -0.112 | down |
| USHBP1 | 0.508 | 6.515 | 2.640 | 0.010 | 0.063 | -2.849 | up | ARF4 | -0.628 | 6.559 | -2.772 | 0.007 | 0.055 | -2.544 | down |
| MMP25 | 0.508 | 4.971 | 3.378 | 0.001 | 0.033 | -1.003 | up | ZFR | -0.628 | 6.395 | -3.353 | 0.001 | 0.034 | -1.072 | down |
| LRRC56 | 0.507 | 4.719 | 2.978 | 0.004 | 0.046 | -2.046 | up | MAL2 | -0.628 | 3.197 | -2.393 | 0.020 | 0.083 | -3.388 | down |
| FAM83G | 0.507 | 7.071 | 2.745 | 0.008 | 0.056 | -2.607 | up | GXYLT2 | -0.628 | 5.871 | -2.480 | 0.016 | 0.075 | -3.204 | down |
| RP3-522P13.2 | 0.507 | 7.278 | 3.155 | 0.002 | 0.039 | -1.597 | up | ZMAT2 | -0.630 | 7.155 | -3.935 | 0.000 | 0.020 | 0.594 | down |
| SBSN | 0.507 | 3.678 | 2.262 | 0.027 | 0.097 | -3.656 | up | SNRPG | -0.630 | 7.149 | -2.590 | 0.012 | 0.066 | -2.961 | down |
| GBX2 | 0.507 | 5.032 | 3.173 | 0.002 | 0.039 | -1.548 | up | ZFPM2 | -0.630 | 3.769 | -2.542 | 0.013 | 0.070 | -3.068 | down |
| GGTLC2 | 0.506 | 4.233 | 3.409 | 0.001 | 0.032 | -0.918 | up | LIPT1 | -0.631 | 4.204 | -3.125 | 0.003 | 0.040 | -1.673 | down |
| HES7 | 0.505 | 5.827 | 2.781 | 0.007 | 0.054 | -2.523 | up | GOLPH3 | -0.631 | 3.612 | -3.167 | 0.002 | 0.039 | -1.565 | down |
| CLDN14 | 0.505 | 4.744 | 2.691 | 0.009 | 0.059 | -2.734 | up | BEX4 | -0.631 | 4.741 | -2.850 | 0.006 | 0.051 | -2.360 | down |
| CTD-3080P12.3 | 0.504 | 4.134 | 3.300 | 0.002 | 0.036 | -1.213 | up | PPP6C | -0.631 | 5.366 | -3.613 | 0.001 | 0.026 | -0.347 | down |
| LOC101927608 | 0.504 | 6.332 | 3.280 | 0.002 | 0.036 | -1.267 | up | PTGIS | -0.632 | 5.729 | -2.056 | 0.044 | 0.128 | -4.051 | down |
| LOC102724880 | 0.504 | 4.683 | 3.164 | 0.002 | 0.039 | -1.572 | up | ALOX5AP | -0.632 | 5.948 | -3.349 | 0.001 | 0.034 | -1.081 | down |
| CENPM | 0.504 | 3.742 | 3.139 | 0.003 | 0.040 | -1.637 | up | CD55 | -0.632 | 6.480 | -2.264 | 0.027 | 0.097 | -3.652 | down |
| NKD2 | 0.502 | 5.825 | 3.953 | 0.000 | 0.020 | 0.648 | up | M6PR | -0.633 | 6.869 | -4.681 | 0.000 | 0.009 | 2.952 | down |
| LOC101927550 | 0.502 | 4.121 | 2.635 | 0.010 | 0.063 | -2.862 | up | BOK | -0.633 | 6.296 | -2.188 | 0.032 | 0.107 | -3.802 | down |
| S1PR5 | 0.502 | 4.090 | 2.919 | 0.005 | 0.048 | -2.191 | up | LILRB5 | -0.633 | 6.028 | -2.747 | 0.008 | 0.056 | -2.604 | down |
| NEUROG2 | 0.502 | 4.425 | 3.015 | 0.004 | 0.044 | -1.952 | up | GPX3 | -0.633 | 9.500 | -3.273 | 0.002 | 0.036 | -1.287 | down |
| IER3 | 0.502 | 5.881 | 3.699 | 0.000 | 0.025 | -0.101 | up | UGDH | -0.633 | 4.970 | -2.485 | 0.015 | 0.075 | -3.192 | down |
| LCN6 | 0.501 | 4.969 | 4.233 | 0.000 | 0.016 | 1.509 | up | MTHFD1 | -0.634 | 5.821 | -3.353 | 0.001 | 0.034 | -1.070 | down |
| DUOXA2 | 0.500 | 5.800 | 2.261 | 0.027 | 0.098 | -3.659 | up | BLNK | -0.634 | 3.950 | -3.030 | 0.003 | 0.044 | -1.916 | down |
| HCRT | 0.500 | 4.695 | 3.355 | 0.001 | 0.034 | -1.064 | up | GLUD1 | -0.634 | 7.138 | -3.531 | 0.001 | 0.028 | -0.578 | down |
| PDCD1 | 0.500 | 6.184 | 2.629 | 0.011 | 0.063 | -2.874 | up | ANO6 | -0.634 | 4.957 | -3.455 | 0.001 | 0.030 | -0.791 | down |
| ATP5J2 | 0.500 | 5.579 | 3.569 | 0.001 | 0.027 | -0.473 | up | ATRAID | -0.635 | 7.450 | -3.639 | 0.001 | 0.026 | -0.273 | down |
| STAG2 | -0.500 | 5.475 | -2.778 | 0.007 | 0.055 | -2.531 | down | SLC25A43 | -0.636 | 3.095 | -2.491 | 0.015 | 0.074 | -3.179 | down |
| IER3IP1 | -0.500 | 3.454 | -3.502 | 0.001 | 0.029 | -0.661 | down | ATXN1L | -0.637 | 6.058 | -3.168 | 0.002 | 0.039 | -1.561 | down |
| PEPD | -0.501 | 6.888 | -3.852 | 0.000 | 0.021 | 0.348 | down | PSMD14 | -0.637 | 5.321 | -3.155 | 0.002 | 0.039 | -1.595 | down |
| SON | -0.501 | 6.584 | -3.016 | 0.004 | 0.044 | -1.952 | down | DBI | -0.638 | 7.730 | -3.003 | 0.004 | 0.045 | -1.983 | down |
| SLC26A2 | -0.501 | 3.776 | -3.217 | 0.002 | 0.038 | -1.433 | down | TSPYL5 | -0.638 | 4.476 | -3.452 | 0.001 | 0.030 | -0.799 | down |
| UBE2E2 | -0.501 | 5.299 | -3.984 | 0.000 | 0.020 | 0.743 | down | TUSC1 | -0.638 | 6.559 | -3.814 | 0.000 | 0.022 | 0.236 | down |
| GPD1L | -0.501 | 5.469 | -2.711 | 0.009 | 0.058 | -2.687 | down | CHP1 | -0.639 | 8.188 | -4.718 | 0.000 | 0.008 | 3.073 | down |
| LSM6 | -0.501 | 6.703 | -4.215 | 0.000 | 0.016 | 1.453 | down | C1S | -0.640 | 8.268 | -2.571 | 0.012 | 0.068 | -3.005 | down |
| USP3 | -0.501 | 4.801 | -4.824 | 0.000 | 0.007 | 3.426 | down | EPDR1 | -0.640 | 4.810 | -2.214 | 0.030 | 0.104 | -3.752 | down |
| PRKAG1 | -0.502 | 5.906 | -3.580 | 0.001 | 0.027 | -0.441 | down | TXNDC12 | -0.640 | 7.120 | -3.333 | 0.001 | 0.034 | -1.124 | down |
| ZDHHC6 | -0.502 | 6.230 | -2.239 | 0.029 | 0.100 | -3.702 | down | GLO1 | -0.640 | 6.957 | -3.966 | 0.000 | 0.020 | 0.690 | down |
| ZFHX4 | -0.502 | 4.689 | -2.290 | 0.025 | 0.094 | -3.600 | down | PHYH | -0.640 | 5.501 | -3.230 | 0.002 | 0.038 | -1.400 | down |
| PDZD11 | -0.503 | 5.724 | -2.578 | 0.012 | 0.067 | -2.990 | down | GDI2 | -0.640 | 7.843 | -3.416 | 0.001 | 0.032 | -0.900 | down |
| ADAM9 | -0.504 | 4.872 | -3.176 | 0.002 | 0.039 | -1.542 | down | MORF4L1 | -0.640 | 6.835 | -2.901 | 0.005 | 0.049 | -2.235 | down |
| SCAF8 | -0.504 | 3.897 | -2.302 | 0.024 | 0.093 | -3.576 | down | LIPA | -0.641 | 6.641 | -2.897 | 0.005 | 0.049 | -2.244 | down |
| RPL35 | -0.504 | 9.156 | -3.413 | 0.001 | 0.032 | -0.906 | down | BIRC6 | -0.641 | 5.516 | -2.749 | 0.008 | 0.056 | -2.599 | down |
| ZNF655 | -0.505 | 5.094 | -2.908 | 0.005 | 0.049 | -2.218 | down | PTTG1IP | -0.642 | 7.780 | -2.807 | 0.007 | 0.053 | -2.462 | down |
| MCCC1 | -0.505 | 6.006 | -2.854 | 0.006 | 0.051 | -2.350 | down | SLC2A10 | -0.642 | 3.808 | -2.939 | 0.005 | 0.047 | -2.142 | down |
| SSBP3 | -0.505 | 7.246 | -3.158 | 0.002 | 0.039 | -1.587 | down | GOLGA5 | -0.642 | 5.184 | -3.381 | 0.001 | 0.033 | -0.995 | down |
| OAZ1 | -0.505 | 9.010 | -4.609 | 0.000 | 0.010 | 2.714 | down | PDGFD | -0.642 | 6.083 | -2.174 | 0.033 | 0.109 | -3.829 | down |
| FAM213A | -0.505 | 6.155 | -2.854 | 0.006 | 0.051 | -2.351 | down | TMEM131 | -0.643 | 6.114 | -3.988 | 0.000 | 0.020 | 0.756 | down |
| ADAR | -0.506 | 8.331 | -3.410 | 0.001 | 0.032 | -0.914 | down | SPATA20 | -0.645 | 7.228 | -3.632 | 0.001 | 0.026 | -0.292 | down |
| RBPJ | -0.506 | 5.464 | -3.549 | 0.001 | 0.028 | -0.529 | down | SUCLA2 | -0.646 | 4.583 | -2.900 | 0.005 | 0.049 | -2.237 | down |
| ATG4A | -0.506 | 3.948 | -2.607 | 0.011 | 0.065 | -2.925 | down | NDUFA12 | -0.646 | 7.309 | -3.282 | 0.002 | 0.036 | -1.262 | down |
| COMMD9 | -0.507 | 6.236 | -3.974 | 0.000 | 0.020 | 0.712 | down | ATP5J | -0.647 | 8.257 | -3.763 | 0.000 | 0.023 | 0.084 | down |
| ITGAV | -0.507 | 4.190 | -2.411 | 0.019 | 0.081 | -3.351 | down | C10orf32 | -0.648 | 4.374 | -3.848 | 0.000 | 0.021 | 0.337 | down |
| RABL3 | -0.507 | 4.381 | -4.287 | 0.000 | 0.015 | 1.681 | down | SLC25A1 | -0.648 | 7.274 | -4.499 | 0.000 | 0.012 | 2.356 | down |
| KIAA0922 | -0.508 | 6.266 | -2.459 | 0.017 | 0.077 | -3.248 | down | CD14 | -0.649 | 7.677 | -4.044 | 0.000 | 0.019 | 0.925 | down |
| EBAG9 | -0.508 | 4.747 | -2.387 | 0.020 | 0.084 | -3.401 | down | PRNP | -0.649 | 6.907 | -3.499 | 0.001 | 0.029 | -0.669 | down |
| DNAJA1 | -0.508 | 6.193 | -2.611 | 0.011 | 0.064 | -2.914 | down | DCTN6 | -0.649 | 7.542 | -3.267 | 0.002 | 0.037 | -1.301 | down |
| SYPL1 | -0.508 | 6.249 | -2.799 | 0.007 | 0.054 | -2.482 | down | GTF2I | -0.650 | 7.618 | -3.934 | 0.000 | 0.020 | 0.593 | down |
| UBE2D2 | -0.508 | 5.976 | -3.430 | 0.001 | 0.031 | -0.860 | down | AKR1B1 | -0.650 | 6.402 | -5.157 | 0.000 | 0.007 | 4.554 | down |
| PDHB | -0.508 | 6.455 | -3.518 | 0.001 | 0.029 | -0.616 | down | ANKRD49 | -0.650 | 3.919 | -3.312 | 0.001 | 0.035 | -1.180 | down |
| ABI1 | -0.508 | 4.003 | -3.003 | 0.004 | 0.045 | -1.984 | down | YWHAB | -0.651 | 7.469 | -3.211 | 0.002 | 0.039 | -1.450 | down |
| NUDT19 | -0.509 | 2.904 | -2.546 | 0.013 | 0.070 | -3.061 | down | DPP4 | -0.651 | 5.076 | -2.759 | 0.007 | 0.056 | -2.575 | down |
| PIGP | -0.509 | 6.925 | -2.868 | 0.006 | 0.050 | -2.316 | down | EMB | -0.651 | 4.587 | -4.060 | 0.000 | 0.019 | 0.975 | down |
| ME2 | -0.509 | 5.647 | -3.375 | 0.001 | 0.033 | -1.011 | down | HADHB | -0.651 | 6.841 | -3.913 | 0.000 | 0.020 | 0.528 | down |
| ACOT13 | -0.509 | 4.974 | -2.958 | 0.004 | 0.046 | -2.095 | down | LIMA1 | -0.651 | 7.152 | -2.975 | 0.004 | 0.046 | -2.053 | down |
| RBBP8 | -0.510 | 4.282 | -2.603 | 0.011 | 0.065 | -2.932 | down | KAT7 | -0.652 | 6.321 | -3.668 | 0.000 | 0.025 | -0.190 | down |
| ARHGAP21 | -0.510 | 5.895 | -2.554 | 0.013 | 0.069 | -3.042 | down | TMEM138 | -0.652 | 6.404 | -4.010 | 0.000 | 0.020 | 0.821 | down |
| PITPNB | -0.510 | 6.771 | -2.616 | 0.011 | 0.064 | -2.904 | down | BMS1P6 | -0.652 | 4.967 | -3.424 | 0.001 | 0.031 | -0.876 | down |
| SHPK | -0.510 | 5.999 | -4.080 | 0.000 | 0.018 | 1.037 | down | UBA3 | -0.653 | 4.778 | -2.745 | 0.008 | 0.056 | -2.608 | down |
| CTBS | -0.510 | 3.442 | -2.918 | 0.005 | 0.048 | -2.193 | down | LAIR1 | -0.653 | 6.562 | -4.143 | 0.000 | 0.016 | 1.231 | down |
| SSPN | -0.510 | 4.927 | -3.116 | 0.003 | 0.041 | -1.697 | down | UQCRFS1 | -0.654 | 6.112 | -4.037 | 0.000 | 0.019 | 0.903 | down |
| MTCH2 | -0.510 | 6.050 | -4.081 | 0.000 | 0.018 | 1.038 | down | ALG8 | -0.655 | 4.155 | -4.868 | 0.000 | 0.007 | 3.575 | down |
| PIM3 | -0.511 | 6.747 | -3.095 | 0.003 | 0.042 | -1.751 | down | SUCLG2 | -0.655 | 4.411 | -3.055 | 0.003 | 0.043 | -1.853 | down |
| KPNA3 | -0.511 | 4.577 | -2.385 | 0.020 | 0.084 | -3.405 | down | TSG101 | -0.656 | 5.632 | -3.362 | 0.001 | 0.034 | -1.046 | down |
| HLA-DMA | -0.511 | 7.226 | -2.645 | 0.010 | 0.062 | -2.840 | down | FAM8A1 | -0.656 | 5.001 | -2.602 | 0.011 | 0.065 | -2.935 | down |
| RPL7L1 | -0.511 | 4.987 | -3.536 | 0.001 | 0.028 | -0.567 | down | ANG | -0.657 | 6.454 | -3.191 | 0.002 | 0.039 | -1.501 | down |
| ACSL3 | -0.511 | 4.008 | -2.694 | 0.009 | 0.059 | -2.725 | down | GPI | -0.657 | 7.923 | -4.975 | 0.000 | 0.007 | 3.933 | down |
| DHRS3 | -0.512 | 8.792 | -2.527 | 0.014 | 0.071 | -3.101 | down | ABHD14B | -0.658 | 6.458 | -3.738 | 0.000 | 0.024 | 0.013 | down |
| ECI2 | -0.512 | 5.938 | -3.043 | 0.003 | 0.043 | -1.882 | down | PARK7 | -0.659 | 7.033 | -4.402 | 0.000 | 0.013 | 2.044 | down |
| IRF9 | -0.512 | 7.089 | -2.820 | 0.006 | 0.053 | -2.431 | down | C14orf119 | -0.659 | 6.143 | -4.206 | 0.000 | 0.016 | 1.426 | down |
| ADAP2 | -0.512 | 6.363 | -3.086 | 0.003 | 0.042 | -1.774 | down | CXCL12 | -0.660 | 7.982 | -2.866 | 0.006 | 0.050 | -2.321 | down |
| TYW3 | -0.512 | 5.676 | -2.408 | 0.019 | 0.082 | -3.356 | down | TOP2B | -0.660 | 5.698 | -2.574 | 0.012 | 0.067 | -2.998 | down |
| ZBTB8OS | -0.513 | 5.526 | -3.035 | 0.003 | 0.043 | -1.902 | down | MRPL9 | -0.660 | 6.400 | -3.672 | 0.000 | 0.025 | -0.178 | down |
| RRAS2 | -0.513 | 4.908 | -3.344 | 0.001 | 0.034 | -1.096 | down | EPB41L3 | -0.661 | 5.200 | -2.474 | 0.016 | 0.075 | -3.216 | down |
| LDHB | -0.513 | 7.080 | -3.250 | 0.002 | 0.037 | -1.346 | down | EIF2D | -0.661 | 7.030 | -3.660 | 0.000 | 0.025 | -0.212 | down |
| ACP2 | -0.513 | 6.214 | -3.604 | 0.001 | 0.027 | -0.373 | down | METTL23 | -0.661 | 5.277 | -2.939 | 0.005 | 0.047 | -2.143 | down |
| SSR2 | -0.513 | 7.515 | -3.089 | 0.003 | 0.042 | -1.765 | down | MRFAP1 | -0.661 | 7.713 | -3.078 | 0.003 | 0.042 | -1.795 | down |
| ZNF195 | -0.513 | 4.431 | -2.653 | 0.010 | 0.062 | -2.820 | down | DAPK1 | -0.662 | 5.733 | -3.087 | 0.003 | 0.042 | -1.772 | down |
| ATP6V1E1 | -0.513 | 7.628 | -4.241 | 0.000 | 0.016 | 1.535 | down | PLA2G2A | -0.662 | 9.740 | -2.454 | 0.017 | 0.077 | -3.258 | down |
| TMA7 | -0.514 | 9.013 | -3.336 | 0.001 | 0.034 | -1.118 | down | SNX3 | -0.663 | 7.546 | -3.600 | 0.001 | 0.027 | -0.384 | down |
| PCDH18 | -0.514 | 6.762 | -2.031 | 0.046 | 0.132 | -4.096 | down | GFPT2 | -0.665 | 6.511 | -2.765 | 0.007 | 0.055 | -2.561 | down |
| SLC41A2 | -0.514 | 5.074 | -2.666 | 0.010 | 0.061 | -2.790 | down | CCDC53 | -0.666 | 6.343 | -3.198 | 0.002 | 0.039 | -1.484 | down |
| PTRHD1 | -0.514 | 6.758 | -3.157 | 0.002 | 0.039 | -1.590 | down | KCTD12 | -0.666 | 7.965 | -2.190 | 0.032 | 0.107 | -3.797 | down |
| FBL | -0.514 | 7.764 | -3.683 | 0.000 | 0.025 | -0.148 | down | ATP6V1A | -0.666 | 5.636 | -3.066 | 0.003 | 0.042 | -1.825 | down |
| RAB2B | -0.514 | 5.418 | -2.593 | 0.012 | 0.066 | -2.954 | down | DNAJB6 | -0.667 | 6.488 | -3.189 | 0.002 | 0.039 | -1.507 | down |
| PSMG1 | -0.514 | 3.473 | -2.413 | 0.019 | 0.081 | -3.346 | down | TSNAX | -0.667 | 4.795 | -2.731 | 0.008 | 0.057 | -2.640 | down |
| TMEM56 | -0.515 | 2.985 | -3.758 | 0.000 | 0.023 | 0.072 | down | MRPL3 | -0.667 | 6.641 | -2.839 | 0.006 | 0.052 | -2.386 | down |
| NUP43 | -0.515 | 4.896 | -4.174 | 0.000 | 0.016 | 1.326 | down | SEC11A | -0.668 | 7.334 | -4.143 | 0.000 | 0.016 | 1.228 | down |
| GOT2 | -0.515 | 5.820 | -2.422 | 0.018 | 0.080 | -3.327 | down | AQPEP | -0.668 | 3.646 | -2.140 | 0.036 | 0.114 | -3.894 | down |
| DYNLT1 | -0.515 | 6.179 | -2.882 | 0.005 | 0.049 | -2.282 | down | MMADHC | -0.669 | 6.627 | -2.713 | 0.008 | 0.058 | -2.683 | down |
| AK6 | -0.516 | 5.273 | -2.767 | 0.007 | 0.055 | -2.558 | down | MPV17 | -0.669 | 5.656 | -3.951 | 0.000 | 0.020 | 0.643 | down |
| TMEM184C | -0.516 | 5.766 | -3.079 | 0.003 | 0.042 | -1.791 | down | LOC100996740 | -0.670 | 7.316 | -3.498 | 0.001 | 0.029 | -0.671 | down |
| ZFAND1 | -0.516 | 4.297 | -2.133 | 0.037 | 0.115 | -3.907 | down | DIAPH2 | -0.670 | 5.402 | -4.206 | 0.000 | 0.016 | 1.425 | down |
| ALG9 | -0.517 | 4.548 | -3.489 | 0.001 | 0.029 | -0.698 | down | BNIP3 | -0.673 | 6.171 | -3.244 | 0.002 | 0.037 | -1.363 | down |
| PAFAH1B1 | -0.517 | 6.274 | -2.999 | 0.004 | 0.045 | -1.994 | down | GNS | -0.673 | 6.777 | -3.932 | 0.000 | 0.020 | 0.585 | down |
| ITGB3BP | -0.518 | 5.465 | -2.270 | 0.026 | 0.096 | -3.639 | down | PSMA4 | -0.674 | 6.272 | -3.292 | 0.002 | 0.036 | -1.233 | down |
| DRG1 | -0.518 | 6.136 | -4.610 | 0.000 | 0.010 | 2.718 | down | IFI16 | -0.676 | 7.322 | -3.360 | 0.001 | 0.034 | -1.050 | down |
| DDX17 | -0.518 | 8.111 | -2.931 | 0.005 | 0.047 | -2.161 | down | COX7A2 | -0.676 | 8.083 | -3.540 | 0.001 | 0.028 | -0.553 | down |
| SLTM | -0.518 | 3.641 | -2.801 | 0.007 | 0.054 | -2.477 | down | CETN2 | -0.676 | 6.151 | -3.083 | 0.003 | 0.042 | -1.781 | down |
| CCDC28A | -0.519 | 5.878 | -2.877 | 0.005 | 0.050 | -2.295 | down | ARHGAP18 | -0.676 | 4.870 | -3.550 | 0.001 | 0.028 | -0.525 | down |
| SEC24B | -0.519 | 3.073 | -2.527 | 0.014 | 0.071 | -3.101 | down | SOD2 | -0.677 | 5.291 | -2.687 | 0.009 | 0.059 | -2.743 | down |
| VKORC1L1 | -0.519 | 5.674 | -2.457 | 0.017 | 0.077 | -3.254 | down | RBM3 | -0.678 | 5.310 | -3.992 | 0.000 | 0.020 | 0.768 | down |
| AP2B1 | -0.519 | 6.445 | -3.490 | 0.001 | 0.029 | -0.695 | down | EHBP1 | -0.678 | 6.810 | -3.242 | 0.002 | 0.037 | -1.368 | down |
| ZNF43 | -0.519 | 4.120 | -2.436 | 0.018 | 0.079 | -3.298 | down | RNASE6 | -0.679 | 5.731 | -3.104 | 0.003 | 0.041 | -1.727 | down |
| RPS27L | -0.520 | 4.853 | -3.121 | 0.003 | 0.041 | -1.683 | down | MYL12B | -0.679 | 8.608 | -3.240 | 0.002 | 0.037 | -1.373 | down |
| AGO2 | -0.520 | 6.128 | -2.215 | 0.030 | 0.104 | -3.749 | down | AIDA | -0.680 | 6.151 | -2.813 | 0.006 | 0.053 | -2.448 | down |
| UBE2K | -0.520 | 5.272 | -3.260 | 0.002 | 0.037 | -1.320 | down | ADM | -0.681 | 6.639 | -2.218 | 0.030 | 0.103 | -3.743 | down |
| WLS | -0.520 | 6.014 | -2.521 | 0.014 | 0.072 | -3.114 | down | MMGT1 | -0.681 | 6.577 | -3.146 | 0.002 | 0.040 | -1.619 | down |
| ZMAT3 | -0.521 | 4.701 | -3.666 | 0.000 | 0.025 | -0.196 | down | PPP3CA | -0.681 | 6.738 | -2.815 | 0.006 | 0.053 | -2.443 | down |
| KDM3B | -0.521 | 4.711 | -3.163 | 0.002 | 0.039 | -1.574 | down | RNASE4 | -0.682 | 6.198 | -4.159 | 0.000 | 0.016 | 1.280 | down |
| RAD21 | -0.521 | 6.448 | -2.497 | 0.015 | 0.074 | -3.167 | down | LINC00094 | -0.685 | 5.337 | -4.527 | 0.000 | 0.012 | 2.447 | down |
| LOC100996756 | -0.521 | 5.867 | -2.675 | 0.009 | 0.060 | -2.770 | down | OAT | -0.686 | 6.729 | -2.384 | 0.020 | 0.084 | -3.407 | down |
| EPSTI1 | -0.521 | 5.166 | -3.241 | 0.002 | 0.037 | -1.370 | down | PRDX6 | -0.687 | 7.680 | -3.574 | 0.001 | 0.027 | -0.458 | down |
| SEPT10 | -0.521 | 4.770 | -2.435 | 0.018 | 0.079 | -3.301 | down | RAB10 | -0.688 | 6.915 | -3.323 | 0.001 | 0.035 | -1.151 | down |
| ADH5 | -0.521 | 6.027 | -2.775 | 0.007 | 0.055 | -2.538 | down | NUS1 | -0.689 | 5.899 | -2.878 | 0.005 | 0.050 | -2.292 | down |
| CD63 | -0.522 | 9.502 | -3.970 | 0.000 | 0.020 | 0.701 | down | FAM96A | -0.689 | 5.250 | -2.936 | 0.005 | 0.047 | -2.150 | down |
| GLTP | -0.522 | 6.006 | -2.585 | 0.012 | 0.066 | -2.973 | down | DDHD2 | -0.689 | 5.826 | -3.130 | 0.003 | 0.040 | -1.661 | down |
| BNIP2 | -0.522 | 4.519 | -2.228 | 0.029 | 0.102 | -3.723 | down | GALNT1 | -0.691 | 6.485 | -3.243 | 0.002 | 0.037 | -1.366 | down |
| PPA1 | -0.522 | 6.757 | -3.141 | 0.003 | 0.040 | -1.631 | down | TGFBI | -0.692 | 8.076 | -2.707 | 0.009 | 0.058 | -2.697 | down |
| NUFIP2 | -0.522 | 5.521 | -2.694 | 0.009 | 0.059 | -2.726 | down | HMGN3 | -0.693 | 6.185 | -2.478 | 0.016 | 0.075 | -3.207 | down |
| UBB | -0.522 | 9.458 | -3.155 | 0.002 | 0.039 | -1.596 | down | MICU2 | -0.693 | 3.988 | -2.579 | 0.012 | 0.067 | -2.987 | down |
| MAFB | -0.522 | 7.276 | -2.975 | 0.004 | 0.046 | -2.054 | down | PIK3AP1 | -0.694 | 4.075 | -2.780 | 0.007 | 0.054 | -2.526 | down |
| ZBTB44 | -0.523 | 4.386 | -2.883 | 0.005 | 0.049 | -2.279 | down | KIT | -0.695 | 5.167 | -2.398 | 0.019 | 0.083 | -3.378 | down |
| AKR1A1 | -0.523 | 6.469 | -3.120 | 0.003 | 0.041 | -1.686 | down | SNX6 | -0.695 | 5.438 | -3.409 | 0.001 | 0.032 | -0.917 | down |
| SQRDL | -0.523 | 6.458 | -3.735 | 0.000 | 0.024 | 0.005 | down | SC5D | -0.695 | 5.192 | -4.586 | 0.000 | 0.010 | 2.641 | down |
| RWDD2B | -0.523 | 5.374 | -3.451 | 0.001 | 0.030 | -0.802 | down | CCDC80 | -0.696 | 7.982 | -2.516 | 0.014 | 0.072 | -3.126 | down |
| SHQ1 | -0.524 | 5.310 | -3.028 | 0.003 | 0.044 | -1.920 | down | LOC100190986 | -0.697 | 7.891 | -2.729 | 0.008 | 0.057 | -2.646 | down |
| HCLS1 | -0.525 | 5.934 | -2.104 | 0.039 | 0.120 | -3.962 | down | TMED2 | -0.697 | 7.785 | -2.972 | 0.004 | 0.046 | -2.061 | down |
| CRNKL1 | -0.525 | 5.565 | -2.325 | 0.023 | 0.090 | -3.528 | down | USP54 | -0.698 | 6.751 | -3.178 | 0.002 | 0.039 | -1.536 | down |
| PLEKHF2 | -0.525 | 3.916 | -2.967 | 0.004 | 0.046 | -2.074 | down | HNRNPK | -0.698 | 7.207 | -3.403 | 0.001 | 0.032 | -0.934 | down |
| CHIC2 | -0.525 | 4.681 | -2.321 | 0.023 | 0.091 | -3.537 | down | HCAR1 | -0.699 | 4.360 | -4.122 | 0.000 | 0.017 | 1.164 | down |
| NOTCH2 | -0.525 | 6.592 | -3.540 | 0.001 | 0.028 | -0.555 | down | CYFIP1 | -0.699 | 7.059 | -3.578 | 0.001 | 0.027 | -0.447 | down |
| TOPBP1 | -0.526 | 3.365 | -3.653 | 0.001 | 0.026 | -0.234 | down | DNMBP | -0.699 | 5.667 | -4.048 | 0.000 | 0.019 | 0.938 | down |
| GFPT1 | -0.526 | 4.781 | -2.514 | 0.014 | 0.072 | -3.131 | down | MAN2B2 | -0.699 | 7.835 | -3.656 | 0.001 | 0.026 | -0.225 | down |
| KIAA1191 | -0.526 | 5.032 | -2.295 | 0.025 | 0.094 | -3.590 | down | RAB9A | -0.700 | 6.477 | -3.824 | 0.000 | 0.022 | 0.265 | down |
| TTC19 | -0.526 | 5.272 | -2.424 | 0.018 | 0.080 | -3.324 | down | CTSC | -0.700 | 5.582 | -4.460 | 0.000 | 0.012 | 2.232 | down |
| PGM1 | -0.526 | 6.703 | -2.523 | 0.014 | 0.071 | -3.110 | down | CECR1 | -0.700 | 6.307 | -3.230 | 0.002 | 0.038 | -1.400 | down |
| CCDC104 | -0.527 | 5.207 | -2.566 | 0.013 | 0.068 | -3.016 | down | ENPP2 | -0.701 | 5.756 | -2.012 | 0.048 | 0.136 | -4.131 | down |
| NDUFAB1 | -0.528 | 6.945 | -3.271 | 0.002 | 0.036 | -1.292 | down | GPR137B | -0.702 | 4.766 | -3.402 | 0.001 | 0.032 | -0.938 | down |
| STARD7 | -0.528 | 6.827 | -3.748 | 0.000 | 0.023 | 0.042 | down | YPEL5 | -0.704 | 6.356 | -3.337 | 0.001 | 0.034 | -1.114 | down |
| HINT2 | -0.528 | 7.126 | -3.903 | 0.000 | 0.020 | 0.498 | down | COL3A1 | -0.705 | 7.591 | -2.611 | 0.011 | 0.064 | -2.915 | down |
| TAB2 | -0.528 | 5.874 | -2.995 | 0.004 | 0.045 | -2.004 | down | FMO3 | -0.705 | 3.667 | -3.293 | 0.002 | 0.036 | -1.231 | down |
| FKBP9 | -0.528 | 8.495 | -2.696 | 0.009 | 0.059 | -2.721 | down | GJA1 | -0.706 | 4.824 | -2.327 | 0.023 | 0.090 | -3.525 | down |
| TERF2IP | -0.529 | 6.503 | -2.778 | 0.007 | 0.054 | -2.530 | down | PAN3 | -0.706 | 5.759 | -2.863 | 0.006 | 0.050 | -2.327 | down |
| DENND5A | -0.529 | 6.330 | -3.069 | 0.003 | 0.042 | -1.817 | down | LDHA | -0.707 | 8.485 | -3.660 | 0.001 | 0.025 | -0.214 | down |
| PPP3R1 | -0.529 | 6.288 | -3.274 | 0.002 | 0.036 | -1.284 | down | LY96 | -0.707 | 5.416 | -3.249 | 0.002 | 0.037 | -1.349 | down |
| KIAA1598 | -0.530 | 4.806 | -2.473 | 0.016 | 0.075 | -3.218 | down | TMEM167A | -0.708 | 4.394 | -3.383 | 0.001 | 0.033 | -0.988 | down |
| TIMP2 | -0.530 | 9.099 | -2.048 | 0.045 | 0.129 | -4.066 | down | NPC2 | -0.708 | 7.861 | -5.038 | 0.000 | 0.007 | 4.147 | down |
| WDFY1 | -0.530 | 5.889 | -3.396 | 0.001 | 0.032 | -0.954 | down | OXA1L | -0.708 | 8.196 | -4.780 | 0.000 | 0.008 | 3.279 | down |
| FRMD6 | -0.530 | 3.971 | -3.256 | 0.002 | 0.037 | -1.330 | down | PDPR | -0.709 | 5.621 | -4.364 | 0.000 | 0.014 | 1.923 | down |
| GLT8D1 | -0.531 | 4.061 | -3.789 | 0.000 | 0.022 | 0.162 | down | TMEM14C | -0.711 | 6.310 | -3.128 | 0.003 | 0.040 | -1.666 | down |
| TRDV3 | -0.531 | 4.051 | -3.851 | 0.000 | 0.021 | 0.343 | down | FTO | -0.711 | 8.029 | -3.881 | 0.000 | 0.021 | 0.434 | down |
| EIF3L | -0.531 | 8.775 | -3.532 | 0.001 | 0.028 | -0.576 | down | PEX11B | -0.711 | 5.660 | -2.810 | 0.006 | 0.053 | -2.454 | down |
| NFAT5 | -0.531 | 6.506 | -2.640 | 0.010 | 0.063 | -2.850 | down | TGFBR3 | -0.713 | 6.764 | -2.669 | 0.010 | 0.061 | -2.783 | down |
| CHTF8 | -0.532 | 7.020 | -3.841 | 0.000 | 0.022 | 0.314 | down | MDH1 | -0.714 | 6.465 | -3.451 | 0.001 | 0.030 | -0.804 | down |
| GYS1 | -0.532 | 5.951 | -3.722 | 0.000 | 0.024 | -0.033 | down | PKD2 | -0.714 | 4.600 | -2.478 | 0.016 | 0.075 | -3.207 | down |
| CMPK1 | -0.532 | 4.990 | -3.064 | 0.003 | 0.042 | -1.830 | down | C15orf61 | -0.714 | 4.728 | -3.268 | 0.002 | 0.037 | -1.298 | down |
| PGRMC2 | -0.533 | 4.860 | -2.992 | 0.004 | 0.045 | -2.010 | down | RNF11 | -0.715 | 4.676 | -2.665 | 0.010 | 0.061 | -2.792 | down |
| GIMAP4 | -0.533 | 5.858 | -2.563 | 0.013 | 0.068 | -3.022 | down | SPCS1 | -0.716 | 7.252 | -2.915 | 0.005 | 0.048 | -2.201 | down |
| LINC00893 | -0.533 | 3.176 | -2.738 | 0.008 | 0.057 | -2.625 | down | ADD3 | -0.718 | 6.623 | -2.767 | 0.007 | 0.055 | -2.555 | down |
| C6orf136 | -0.533 | 5.124 | -3.761 | 0.000 | 0.023 | 0.079 | down | ARL6IP1 | -0.722 | 6.732 | -2.911 | 0.005 | 0.048 | -2.211 | down |
| PLP2 | -0.533 | 7.724 | -3.263 | 0.002 | 0.037 | -1.313 | down | PPAP2B | -0.725 | 8.098 | -2.750 | 0.008 | 0.056 | -2.597 | down |
| IGSF10 | -0.533 | 4.640 | -2.917 | 0.005 | 0.048 | -2.196 | down | TNFSF10 | -0.727 | 5.353 | -3.459 | 0.001 | 0.030 | -0.780 | down |
| CCDC6 | -0.533 | 5.676 | -2.026 | 0.047 | 0.133 | -4.105 | down | SERINC1 | -0.730 | 5.563 | -2.506 | 0.015 | 0.073 | -3.146 | down |
| UBL3 | -0.533 | 5.165 | -2.316 | 0.024 | 0.091 | -3.546 | down | SNX9 | -0.731 | 7.138 | -3.753 | 0.000 | 0.023 | 0.055 | down |
| GLRX5 | -0.533 | 7.638 | -3.640 | 0.001 | 0.026 | -0.270 | down | COLEC12 | -0.732 | 5.393 | -2.259 | 0.027 | 0.098 | -3.662 | down |
| GALNT7 | -0.534 | 2.848 | -2.082 | 0.041 | 0.124 | -4.002 | down | IRF2 | -0.733 | 5.626 | -2.717 | 0.008 | 0.058 | -2.673 | down |
| MPP1 | -0.534 | 6.016 | -4.275 | 0.000 | 0.015 | 1.642 | down | RSPO3 | -0.733 | 5.377 | -2.636 | 0.010 | 0.063 | -2.858 | down |
| GNG12 | -0.534 | 6.516 | -3.113 | 0.003 | 0.041 | -1.705 | down | MEIS1 | -0.735 | 5.408 | -2.179 | 0.033 | 0.108 | -3.819 | down |
| CSNK2B | -0.534 | 8.520 | -3.447 | 0.001 | 0.030 | -0.813 | down | PDGFC | -0.735 | 4.702 | -4.466 | 0.000 | 0.012 | 2.250 | down |
| DROSHA | -0.534 | 6.016 | -2.727 | 0.008 | 0.057 | -2.651 | down | ST13 | -0.736 | 6.809 | -3.737 | 0.000 | 0.024 | 0.011 | down |
| KDELR2 | -0.534 | 6.738 | -3.199 | 0.002 | 0.039 | -1.481 | down | MAOA | -0.736 | 6.801 | -3.056 | 0.003 | 0.043 | -1.850 | down |
| TMEM9B | -0.534 | 6.280 | -3.128 | 0.003 | 0.040 | -1.665 | down | SOX9 | -0.736 | 6.775 | -2.674 | 0.009 | 0.060 | -2.772 | down |
| HSPBAP1 | -0.534 | 4.012 | -2.769 | 0.007 | 0.055 | -2.553 | down | NARS | -0.739 | 6.350 | -3.276 | 0.002 | 0.036 | -1.276 | down |
| LPIN1 | -0.535 | 5.773 | -3.027 | 0.004 | 0.044 | -1.922 | down | GNG10 | -0.740 | 6.798 | -3.033 | 0.003 | 0.043 | -1.909 | down |
| CDIPT | -0.535 | 7.044 | -3.312 | 0.001 | 0.035 | -1.181 | down | ZMPSTE24 | -0.740 | 5.812 | -3.574 | 0.001 | 0.027 | -0.459 | down |
| APLP2 | -0.535 | 7.576 | -4.456 | 0.000 | 0.012 | 2.219 | down | ZBED5-AS1 | -0.741 | 5.350 | -3.356 | 0.001 | 0.034 | -1.061 | down |
| RP3-368A4.6 | -0.535 | 4.653 | -2.021 | 0.047 | 0.134 | -4.115 | down | NID2 | -0.743 | 4.913 | -2.557 | 0.013 | 0.069 | -3.036 | down |
| MTX2 | -0.535 | 5.619 | -2.705 | 0.009 | 0.059 | -2.702 | down | CTSO | -0.744 | 6.238 | -3.180 | 0.002 | 0.039 | -1.531 | down |
| ADIRF-AS1 | -0.535 | 5.775 | -2.726 | 0.008 | 0.057 | -2.653 | down | C14orf166 | -0.745 | 7.263 | -3.757 | 0.000 | 0.023 | 0.067 | down |
| NDUFA1 | -0.535 | 7.957 | -3.413 | 0.001 | 0.032 | -0.907 | down | YWHAQ | -0.745 | 6.982 | -3.548 | 0.001 | 0.028 | -0.532 | down |
| MRPL45 | -0.535 | 5.851 | -4.318 | 0.000 | 0.015 | 1.776 | down | DNM1 | -0.746 | 7.846 | -2.781 | 0.007 | 0.054 | -2.525 | down |
| NAE1 | -0.535 | 6.176 | -2.918 | 0.005 | 0.048 | -2.195 | down | BOLA3 | -0.747 | 7.083 | -3.269 | 0.002 | 0.037 | -1.297 | down |
| ARF3 | -0.535 | 6.963 | -3.381 | 0.001 | 0.033 | -0.995 | down | TMEM230 | -0.747 | 7.572 | -3.811 | 0.000 | 0.022 | 0.227 | down |
| IARS2 | -0.536 | 6.681 | -3.696 | 0.000 | 0.025 | -0.110 | down | SFT2D2 | -0.747 | 6.237 | -4.484 | 0.000 | 0.012 | 2.310 | down |
| TUBB | -0.536 | 8.735 | -3.451 | 0.001 | 0.030 | -0.802 | down | C17orf58 | -0.747 | 5.115 | -3.223 | 0.002 | 0.038 | -1.418 | down |
| JAK1 | -0.536 | 6.407 | -3.430 | 0.001 | 0.031 | -0.861 | down | CHRDL1 | -0.747 | 7.621 | -2.387 | 0.020 | 0.084 | -3.400 | down |
| ABCE1 | -0.536 | 5.207 | -2.501 | 0.015 | 0.073 | -3.158 | down | ERH | -0.748 | 7.149 | -3.954 | 0.000 | 0.020 | 0.652 | down |
| SPPL2A | -0.536 | 3.767 | -2.660 | 0.010 | 0.061 | -2.804 | down | CP | -0.749 | 4.570 | -2.429 | 0.018 | 0.080 | -3.313 | down |
| NKIRAS1 | -0.537 | 4.680 | -2.948 | 0.004 | 0.047 | -2.119 | down | ITM2B | -0.750 | 8.252 | -3.250 | 0.002 | 0.037 | -1.347 | down |
| IMPDH2 | -0.537 | 7.652 | -4.342 | 0.000 | 0.014 | 1.854 | down | NDFIP1 | -0.752 | 6.592 | -4.078 | 0.000 | 0.018 | 1.030 | down |
| RPL36AL | -0.537 | 8.680 | -2.664 | 0.010 | 0.061 | -2.795 | down | TM9SF2 | -0.755 | 6.422 | -3.065 | 0.003 | 0.042 | -1.828 | down |
| ABCB7 | -0.537 | 5.547 | -4.088 | 0.000 | 0.018 | 1.059 | down | LRRN4CL | -0.756 | 6.312 | -2.080 | 0.041 | 0.124 | -4.006 | down |
| SIGLEC1 | -0.537 | 5.935 | -2.616 | 0.011 | 0.064 | -2.905 | down | WSB2 | -0.760 | 4.106 | -3.312 | 0.001 | 0.035 | -1.180 | down |
| FAM117A | -0.538 | 6.800 | -3.106 | 0.003 | 0.041 | -1.721 | down | ZNF217 | -0.761 | 3.503 | -3.166 | 0.002 | 0.039 | -1.567 | down |
| CAT | -0.538 | 6.061 | -3.072 | 0.003 | 0.042 | -1.808 | down | CTSB | -0.763 | 8.346 | -4.795 | 0.000 | 0.008 | 3.329 | down |
| CRYBG3 | -0.538 | 5.550 | -2.891 | 0.005 | 0.049 | -2.260 | down | SACM1L | -0.764 | 5.825 | -3.229 | 0.002 | 0.038 | -1.401 | down |
| YIPF5 | -0.538 | 4.871 | -3.111 | 0.003 | 0.041 | -1.710 | down | C6orf120 | -0.765 | 5.057 | -3.935 | 0.000 | 0.020 | 0.595 | down |
| LAMP2 | -0.539 | 4.417 | -2.679 | 0.009 | 0.060 | -2.761 | down | P2RY14 | -0.766 | 4.726 | -2.821 | 0.006 | 0.053 | -2.428 | down |
| IDH1 | -0.539 | 5.108 | -3.454 | 0.001 | 0.030 | -0.794 | down | CCDC69 | -0.769 | 6.787 | -3.782 | 0.000 | 0.022 | 0.141 | down |
| TMEM258 | -0.539 | 8.400 | -2.513 | 0.014 | 0.072 | -3.133 | down | ARL6IP5 | -0.769 | 7.736 | -3.928 | 0.000 | 0.020 | 0.575 | down |
| HIST1H2BE | -0.539 | 8.495 | -3.517 | 0.001 | 0.029 | -0.619 | down | PFN2 | -0.772 | 7.776 | -2.950 | 0.004 | 0.047 | -2.116 | down |
| SLC24A3 | -0.539 | 5.205 | -2.534 | 0.014 | 0.071 | -3.086 | down | PPP1CC | -0.774 | 7.687 | -3.025 | 0.004 | 0.044 | -1.929 | down |
| FKBP1B | -0.540 | 3.653 | -3.019 | 0.004 | 0.044 | -1.943 | down | LOC283070 | -0.775 | 5.922 | -3.076 | 0.003 | 0.042 | -1.800 | down |
| DHX15 | -0.540 | 5.942 | -2.637 | 0.010 | 0.063 | -2.856 | down | C1QA | -0.776 | 8.458 | -3.434 | 0.001 | 0.031 | -0.848 | down |
| EFR3A | -0.540 | 4.089 | -3.005 | 0.004 | 0.045 | -1.978 | down | CD24 | -0.776 | 4.536 | -2.222 | 0.030 | 0.103 | -3.735 | down |
| MBNL3 | -0.541 | 4.017 | -4.840 | 0.000 | 0.007 | 3.478 | down | CSF1R | -0.777 | 7.948 | -3.332 | 0.001 | 0.034 | -1.128 | down |
| RRAGD | -0.541 | 4.917 | -2.764 | 0.007 | 0.055 | -2.563 | down | PLTP | -0.779 | 8.271 | -4.233 | 0.000 | 0.016 | 1.509 | down |
| NFU1 | -0.541 | 5.817 | -2.676 | 0.009 | 0.060 | -2.769 | down | FBN1 | -0.779 | 5.115 | -2.499 | 0.015 | 0.074 | -3.162 | down |
| CWC15 | -0.542 | 7.427 | -3.315 | 0.001 | 0.035 | -1.173 | down | VAMP7 | -0.781 | 6.055 | -2.722 | 0.008 | 0.058 | -2.662 | down |
| ITGAM | -0.542 | 4.947 | -2.477 | 0.016 | 0.075 | -3.210 | down | ALDH1A1 | -0.781 | 5.870 | -4.044 | 0.000 | 0.019 | 0.926 | down |
| SAR1A | -0.542 | 7.096 | -3.487 | 0.001 | 0.029 | -0.702 | down | ISCU | -0.783 | 7.832 | -3.493 | 0.001 | 0.029 | -0.685 | down |
| LAMTOR5 | -0.543 | 6.332 | -3.636 | 0.001 | 0.026 | -0.283 | down | ALDH2 | -0.786 | 8.500 | -3.135 | 0.003 | 0.040 | -1.648 | down |
| SUMO3 | -0.544 | 6.837 | -3.484 | 0.001 | 0.029 | -0.711 | down | GUSBP1 | -0.788 | 5.398 | -2.817 | 0.006 | 0.053 | -2.439 | down |
| GPNMB | -0.544 | 5.351 | -2.924 | 0.005 | 0.048 | -2.179 | down | OSBPL9 | -0.790 | 6.233 | -2.935 | 0.005 | 0.047 | -2.152 | down |
| INTS12 | -0.544 | 5.975 | -3.222 | 0.002 | 0.038 | -1.421 | down | NTN4 | -0.791 | 5.735 | -2.806 | 0.007 | 0.053 | -2.464 | down |
| PHB2 | -0.544 | 8.171 | -3.566 | 0.001 | 0.027 | -0.480 | down | SH3GLB1 | -0.792 | 7.134 | -3.648 | 0.001 | 0.026 | -0.248 | down |
| PIK3R1 | -0.545 | 6.940 | -2.885 | 0.005 | 0.049 | -2.274 | down | PAM | -0.793 | 6.548 | -3.715 | 0.000 | 0.024 | -0.054 | down |
| LSM12 | -0.545 | 5.834 | -4.005 | 0.000 | 0.020 | 0.806 | down | ALDH9A1 | -0.793 | 6.751 | -3.962 | 0.000 | 0.020 | 0.676 | down |
| C7orf49 | -0.545 | 5.454 | -4.746 | 0.000 | 0.008 | 3.166 | down | THRSP | -0.794 | 6.417 | -2.068 | 0.043 | 0.126 | -4.029 | down |
| UBP1 | -0.547 | 6.011 | -2.367 | 0.021 | 0.086 | -3.443 | down | YWHAG | -0.795 | 6.856 | -3.504 | 0.001 | 0.029 | -0.655 | down |
| ITGBL1 | -0.548 | 5.049 | -2.400 | 0.019 | 0.083 | -3.374 | down | CDR1 | -0.796 | 7.868 | -3.294 | 0.002 | 0.036 | -1.228 | down |
| SKI | -0.548 | 8.090 | -2.503 | 0.015 | 0.073 | -3.153 | down | BNIP3L | -0.796 | 7.589 | -3.871 | 0.000 | 0.021 | 0.405 | down |
| LINC00493 | -0.548 | 7.104 | -3.762 | 0.000 | 0.023 | 0.083 | down | RTN4 | -0.797 | 7.742 | -3.504 | 0.001 | 0.029 | -0.656 | down |
| CCDC23 | -0.548 | 5.747 | -2.885 | 0.005 | 0.049 | -2.275 | down | NRIP1 | -0.798 | 4.106 | -4.129 | 0.000 | 0.017 | 1.186 | down |
| SP1 | -0.548 | 7.042 | -3.315 | 0.001 | 0.035 | -1.174 | down | SGCE | -0.798 | 7.178 | -3.324 | 0.001 | 0.035 | -1.149 | down |
| DERA | -0.549 | 4.454 | -3.236 | 0.002 | 0.037 | -1.383 | down | PRDX4 | -0.800 | 5.600 | -2.988 | 0.004 | 0.045 | -2.022 | down |
| ARFIP2 | -0.549 | 5.526 | -2.696 | 0.009 | 0.059 | -2.721 | down | TMEM50A | -0.801 | 6.986 | -3.422 | 0.001 | 0.031 | -0.882 | down |
| ACVR2A | -0.549 | 4.814 | -3.868 | 0.000 | 0.021 | 0.395 | down | COL6A3 | -0.801 | 8.081 | -2.797 | 0.007 | 0.054 | -2.486 | down |
| ARL15 | -0.549 | 5.066 | -2.520 | 0.014 | 0.072 | -3.118 | down | BCAT1 | -0.804 | 4.926 | -5.152 | 0.000 | 0.007 | 4.538 | down |
| DERL2 | -0.549 | 5.419 | -2.748 | 0.008 | 0.056 | -2.601 | down | C1QB | -0.806 | 8.590 | -3.394 | 0.001 | 0.032 | -0.960 | down |
| HSP90AA1 | -0.549 | 8.067 | -2.312 | 0.024 | 0.092 | -3.556 | down | SEC31A | -0.806 | 7.530 | -3.488 | 0.001 | 0.029 | -0.700 | down |
| TBC1D23 | -0.550 | 4.960 | -3.728 | 0.000 | 0.024 | -0.015 | down | CD164 | -0.808 | 7.609 | -3.511 | 0.001 | 0.029 | -0.637 | down |
| PTGFRN | -0.550 | 5.638 | -3.042 | 0.003 | 0.043 | -1.886 | down | CCDC92 | -0.808 | 8.490 | -3.686 | 0.000 | 0.025 | -0.139 | down |
| GPX7 | -0.551 | 5.018 | -3.508 | 0.001 | 0.029 | -0.645 | down | CREG1 | -0.810 | 7.833 | -3.522 | 0.001 | 0.029 | -0.605 | down |
| HTRA1 | -0.551 | 8.655 | -3.121 | 0.003 | 0.041 | -1.683 | down | UBE2E3 | -0.811 | 5.056 | -4.039 | 0.000 | 0.019 | 0.911 | down |
| LOC101929177 | -0.551 | 4.554 | -2.867 | 0.006 | 0.050 | -2.318 | down | MAN1A1 | -0.811 | 4.757 | -3.605 | 0.001 | 0.027 | -0.370 | down |
| CBX1 | -0.551 | 4.999 | -2.507 | 0.015 | 0.073 | -3.146 | down | TRIM22 | -0.813 | 5.073 | -2.477 | 0.016 | 0.075 | -3.210 | down |
| SCARB2 | -0.552 | 5.995 | -3.191 | 0.002 | 0.039 | -1.503 | down | ATP1B1 | -0.814 | 5.311 | -2.597 | 0.012 | 0.066 | -2.946 | down |
| DSG2 | -0.552 | 4.633 | -2.041 | 0.045 | 0.130 | -4.077 | down | TCEAL8 | -0.816 | 4.811 | -2.843 | 0.006 | 0.051 | -2.376 | down |
| TCEB1 | -0.552 | 4.857 | -3.174 | 0.002 | 0.039 | -1.545 | down | ACSL1 | -0.816 | 7.855 | -3.159 | 0.002 | 0.039 | -1.584 | down |
| RPS6KC1 | -0.553 | 5.072 | -2.614 | 0.011 | 0.064 | -2.908 | down | HECTD3 | -0.823 | 8.232 | -4.908 | 0.000 | 0.007 | 3.708 | down |
| F8 | -0.553 | 4.822 | -2.243 | 0.028 | 0.100 | -3.694 | down | SNX10 | -0.826 | 4.890 | -3.069 | 0.003 | 0.042 | -1.818 | down |
| C14orf2 | -0.553 | 7.319 | -3.660 | 0.000 | 0.025 | -0.212 | down | KLHDC2 | -0.826 | 5.808 | -3.691 | 0.000 | 0.025 | -0.125 | down |
| SLC25A46 | -0.554 | 4.455 | -2.871 | 0.005 | 0.050 | -2.308 | down | SCP2 | -0.828 | 6.285 | -3.192 | 0.002 | 0.039 | -1.500 | down |
| ZKSCAN8 | -0.554 | 4.478 | -2.911 | 0.005 | 0.048 | -2.210 | down | SRPX | -0.828 | 7.128 | -3.541 | 0.001 | 0.028 | -0.550 | down |
| EMP3 | -0.555 | 8.945 | -3.654 | 0.001 | 0.026 | -0.231 | down | GHR | -0.833 | 5.579 | -2.277 | 0.026 | 0.096 | -3.625 | down |
| SGMS2 | -0.555 | 4.050 | -3.101 | 0.003 | 0.041 | -1.735 | down | SRP9 | -0.834 | 6.251 | -2.936 | 0.005 | 0.047 | -2.150 | down |
| PJA2 | -0.556 | 5.929 | -2.034 | 0.046 | 0.131 | -4.090 | down | DAB2 | -0.838 | 4.722 | -3.607 | 0.001 | 0.027 | -0.364 | down |
| EID1 | -0.556 | 6.582 | -2.718 | 0.008 | 0.058 | -2.672 | down | GDE1 | -0.840 | 6.494 | -4.868 | 0.000 | 0.007 | 3.573 | down |
| ZNF385A | -0.556 | 8.525 | -3.753 | 0.000 | 0.023 | 0.057 | down | REEP5 | -0.840 | 7.161 | -3.863 | 0.000 | 0.021 | 0.381 | down |
| TXNIP | -0.556 | 9.663 | -3.827 | 0.000 | 0.022 | 0.273 | down | CPNE3 | -0.841 | 6.110 | -3.099 | 0.003 | 0.041 | -1.741 | down |
| THAP9-AS1 | -0.556 | 5.505 | -2.813 | 0.006 | 0.053 | -2.448 | down | IL10RA | -0.842 | 5.094 | -4.641 | 0.000 | 0.009 | 2.820 | down |
| KLF3 | -0.556 | 5.742 | -3.044 | 0.003 | 0.043 | -1.880 | down | MS4A4A | -0.842 | 5.551 | -3.188 | 0.002 | 0.039 | -1.511 | down |
| ASAH1 | -0.556 | 6.123 | -5.356 | 0.000 | 0.006 | 5.241 | down | TRHDE-AS1 | -0.843 | 4.381 | -2.176 | 0.033 | 0.109 | -3.826 | down |
| ABL1 | -0.556 | 8.388 | -2.894 | 0.005 | 0.049 | -2.254 | down | ALX1 | -0.844 | 6.412 | -3.187 | 0.002 | 0.039 | -1.514 | down |
| PEX19 | -0.556 | 6.112 | -3.646 | 0.001 | 0.026 | -0.254 | down | USMG5 | -0.845 | 8.791 | -3.785 | 0.000 | 0.022 | 0.149 | down |
| ANXA4 | -0.556 | 6.071 | -2.253 | 0.028 | 0.099 | -3.674 | down | ANXA5 | -0.846 | 8.476 | -4.231 | 0.000 | 0.016 | 1.502 | down |
| C1QBP | -0.557 | 5.323 | -2.932 | 0.005 | 0.047 | -2.159 | down | ANXA7 | -0.847 | 6.212 | -3.926 | 0.000 | 0.020 | 0.569 | down |
| AK3 | -0.557 | 5.020 | -2.828 | 0.006 | 0.052 | -2.412 | down | SDCBP | -0.850 | 8.798 | -3.613 | 0.001 | 0.026 | -0.347 | down |
| HMGN2 | -0.557 | 8.136 | -3.303 | 0.002 | 0.036 | -1.205 | down | PCYOX1 | -0.853 | 5.929 | -4.712 | 0.000 | 0.008 | 3.055 | down |
| SESN3 | -0.557 | 5.033 | -2.977 | 0.004 | 0.046 | -2.048 | down | CTSK | -0.854 | 6.614 | -2.989 | 0.004 | 0.045 | -2.019 | down |
| SH3BGRL3 | -0.558 | 8.497 | -3.397 | 0.001 | 0.032 | -0.951 | down | LASP1 | -0.857 | 7.843 | -3.705 | 0.000 | 0.024 | -0.084 | down |
| CS | -0.558 | 7.865 | -3.712 | 0.000 | 0.024 | -0.064 | down | IARS | -0.861 | 6.874 | -3.139 | 0.003 | 0.040 | -1.638 | down |
| SSR3 | -0.558 | 5.736 | -3.444 | 0.001 | 0.031 | -0.820 | down | GPR34 | -0.862 | 4.369 | -3.088 | 0.003 | 0.042 | -1.767 | down |
| NR2C2 | -0.558 | 5.022 | -3.032 | 0.003 | 0.043 | -1.910 | down | SEPP1 | -0.862 | 5.486 | -4.191 | 0.000 | 0.016 | 1.379 | down |
| VTI1B | -0.558 | 5.920 | -3.522 | 0.001 | 0.029 | -0.606 | down | KLB | -0.863 | 3.285 | -3.001 | 0.004 | 0.045 | -1.988 | down |
| MPLKIP | -0.558 | 5.216 | -3.503 | 0.001 | 0.029 | -0.659 | down | C1QC | -0.866 | 8.541 | -4.002 | 0.000 | 0.020 | 0.797 | down |
| ANKMY2 | -0.558 | 4.552 | -3.692 | 0.000 | 0.025 | -0.120 | down | VSIG4 | -0.868 | 6.752 | -3.014 | 0.004 | 0.044 | -1.955 | down |
| METTL21B | -0.559 | 5.497 | -2.258 | 0.027 | 0.098 | -3.664 | down | LYVE1 | -0.869 | 7.544 | -2.307 | 0.024 | 0.092 | -3.565 | down |
| MLEC | -0.559 | 6.785 | -4.463 | 0.000 | 0.012 | 2.242 | down | OLFML2A | -0.869 | 6.521 | -3.381 | 0.001 | 0.033 | -0.994 | down |
| CHMP4B | -0.560 | 7.477 | -3.759 | 0.000 | 0.023 | 0.075 | down | OLFML1 | -0.869 | 4.366 | -2.806 | 0.007 | 0.053 | -2.463 | down |
| ASS1 | -0.561 | 8.349 | -3.281 | 0.002 | 0.036 | -1.265 | down | CDK2AP1 | -0.873 | 7.884 | -3.056 | 0.003 | 0.043 | -1.850 | down |
| HEXB | -0.562 | 8.057 | -2.944 | 0.004 | 0.047 | -2.130 | down | SMIM19 | -0.880 | 7.285 | -3.849 | 0.000 | 0.021 | 0.339 | down |
| EVI2B | -0.562 | 3.976 | -2.143 | 0.036 | 0.113 | -3.888 | down | MRC1 | -0.885 | 5.966 | -3.315 | 0.001 | 0.035 | -1.173 | down |
| AIM1 | -0.562 | 5.100 | -2.978 | 0.004 | 0.046 | -2.046 | down | FCER1G | -0.887 | 7.171 | -3.782 | 0.000 | 0.022 | 0.142 | down |
| NIPSNAP3A | -0.562 | 2.121 | -3.521 | 0.001 | 0.029 | -0.607 | down | SFRP4 | -0.893 | 5.948 | -2.988 | 0.004 | 0.045 | -2.020 | down |
| DUSP6 | -0.562 | 5.239 | -2.876 | 0.005 | 0.050 | -2.296 | down | PPT1 | -0.898 | 6.188 | -3.561 | 0.001 | 0.028 | -0.495 | down |
| GTF3A | -0.562 | 5.363 | -3.949 | 0.000 | 0.020 | 0.639 | down | AP3S1 | -0.904 | 7.263 | -3.731 | 0.000 | 0.024 | -0.007 | down |
| IFIT3 | -0.563 | 4.649 | -2.819 | 0.006 | 0.053 | -2.433 | down | CCNG1 | -0.909 | 6.152 | -3.441 | 0.001 | 0.031 | -0.830 | down |
| CRYL1 | -0.563 | 5.854 | -3.728 | 0.000 | 0.024 | -0.017 | down | PPP1R1A | -0.913 | 7.399 | -2.650 | 0.010 | 0.062 | -2.828 | down |
| ATP6AP1 | -0.563 | 6.579 | -3.325 | 0.001 | 0.035 | -1.145 | down | WWP1 | -0.913 | 4.093 | -4.317 | 0.000 | 0.015 | 1.774 | down |
| EPM2AIP1 | -0.563 | 5.422 | -2.888 | 0.005 | 0.049 | -2.266 | down | GBAS | -0.913 | 6.516 | -4.001 | 0.000 | 0.020 | 0.794 | down |
| DIRC2 | -0.564 | 4.217 | -4.433 | 0.000 | 0.013 | 2.143 | down | NCOA4 | -0.919 | 8.332 | -4.223 | 0.000 | 0.016 | 1.480 | down |
| LRRC58 | -0.564 | 5.001 | -2.926 | 0.005 | 0.048 | -2.174 | down | LOC284825 | -0.932 | 4.185 | -3.143 | 0.002 | 0.040 | -1.627 | down |
| GTF3C6 | -0.564 | 6.552 | -2.718 | 0.008 | 0.058 | -2.670 | down | MPEG1 | -0.938 | 6.358 | -4.239 | 0.000 | 0.016 | 1.529 | down |
| HMGN4 | -0.564 | 5.257 | -2.591 | 0.012 | 0.066 | -2.959 | down | DRAM2 | -0.945 | 5.359 | -4.698 | 0.000 | 0.008 | 3.006 | down |
| GABARAPL2 | -0.564 | 7.360 | -3.044 | 0.003 | 0.043 | -1.881 | down | CSTA | -0.963 | 8.253 | -2.574 | 0.012 | 0.067 | -2.997 | down |
| ARPC3 | -0.565 | 7.450 | -3.206 | 0.002 | 0.039 | -1.464 | down | SLC46A3 | -0.970 | 6.740 | -5.091 | 0.000 | 0.007 | 4.328 | down |
| NBPF8 | -0.565 | 3.154 | -2.575 | 0.012 | 0.067 | -2.997 | down | LEPR | -1.000 | 5.824 | -2.344 | 0.022 | 0.088 | -3.490 | down |
| YWHAE | -0.565 | 8.318 | -3.361 | 0.001 | 0.034 | -1.048 | down | PPL | -1.013 | 7.698 | -4.760 | 0.000 | 0.008 | 3.212 | down |
| SEC23B | -0.566 | 3.989 | -2.266 | 0.027 | 0.097 | -3.648 | down | LGMN | -1.068 | 7.264 | -4.168 | 0.000 | 0.016 | 1.307 | down |
| PFDN5 | -0.566 | 9.291 | -3.963 | 0.000 | 0.020 | 0.679 | down | UCP2 | -1.080 | 7.592 | -6.535 | 0.000 | 0.000 | 9.468 | down |
| ABCA9 | -0.566 | 4.905 | -2.130 | 0.037 | 0.116 | -3.913 | down | MXRA5 | -1.094 | 7.758 | -3.635 | 0.001 | 0.026 | -0.285 | down |
| DFNA5 | -0.567 | 3.306 | -3.107 | 0.003 | 0.041 | -1.720 | down | F13A1 | -1.141 | 6.984 | -3.677 | 0.000 | 0.025 | -0.164 | down |
| VEZF1 | -0.567 | 5.927 | -2.849 | 0.006 | 0.051 | -2.361 | down | CFH | -1.203 | 6.507 | -4.549 | 0.000 | 0.011 | 2.518 | down |

**Supplementary Table S3.** The genes contained in each module

| **Gene** | **Module** | **Gene** | **Module** | **Gene** | **Module** | **Gene** | **Module** | **Gene** | **Module** | **Gene** | **Module** | **Gene** | **Module** | **Gene** | **Module** |
| --- | --- | --- | --- | --- | --- | --- | --- | --- | --- | --- | --- | --- | --- | --- | --- |
| A1BG | blue | LINC00176 | blue | TFPT | blue | NMI | cyan | CTSK | darkorange | DDIT3 | grey | LOC101928731 | grey | RP11-506E9.3 | grey |
| A1BG-AS1 | blue | LINC00208 | blue | TFR2 | blue | NNMT | cyan | CUTC | darkorange | DDIT4 | grey | LOC101928751 | grey | RP11-506N2.1 | grey |
| A2ML1 | blue | LINC00226 | blue | TGFB3 | blue | NNT | cyan | CXCL12 | darkorange | DDRGK1 | grey | LOC101928767 | grey | RP11-506O24.2 | grey |
| A4GALT | blue | LINC00282 | blue | TGM1 | blue | NODAL | cyan | CXXC4 | darkorange | DDX1 | grey | LOC101928770 | grey | RP11-50B3.4 | grey |
| A4GNT | blue | LINC00293 | blue | TGM6 | blue | NOL7 | cyan | CYB5A | darkorange | DDX11 | grey | LOC101928779 | grey | RP11-50E11.3 | grey |
| AANAT | blue | LINC00294 | blue | TH | blue | NOL8 | cyan | CYB5R3 | darkorange | DDX19B | grey | LOC101928781 | grey | RP11-510C10.2 | grey |
| AB488780 | blue | LINC00304 | blue | THAP3 | blue | NONO | cyan | CYBB | darkorange | DDX21 | grey | LOC101928790 | grey | RP11-515O17.3 | grey |
| ABCA2 | blue | LINC00311 | blue | THAP7 | blue | NOP58 | cyan | CYBRD1 | darkorange | DDX25 | grey | LOC101928797 | grey | RP11-517B11.7 | grey |
| ABCA3 | blue | LINC00313 | blue | THAP8 | blue | NOTCH2 | cyan | CYP1B1 | darkorange | DDX26B | grey | LOC101928806 | grey | RP11-521D12.1 | grey |
| ABCA4 | blue | LINC00315 | blue | THEG | blue | NPB | cyan | CYYR1 | darkorange | DDX39A | grey | LOC101928817 | grey | RP11-521I2.3 | grey |
| ABCA7 | blue | LINC00334 | blue | THOC1 | blue | NPEPPS | cyan | DAAM2 | darkorange | DDX3Y | grey | LOC101928820 | grey | RP11-521O16.2 | grey |
| ABCB6 | blue | LINC00355 | blue | THOC2 | blue | NPM1 | cyan | DACT1 | darkorange | DDX41 | grey | LOC101928834 | grey | RP11-52A20.2 | grey |
| ABCB8 | blue | LINC00421 | blue | THOC6 | blue | NPTN | cyan | DCLK1 | darkorange | DDX43 | grey | LOC101928861 | grey | RP11-534L20.5 | grey |
| ABCB9 | blue | LINC00427 | blue | THOP1 | blue | NPTN-IT1 | cyan | DCN | darkorange | DDX53 | grey | LOC101928865 | grey | RP11-539L10.3 | grey |
| ABCC10 | blue | LINC00445 | blue | THUMPD3-AS1 | blue | NR1D2 | cyan | DCUN1D3 | darkorange | DDX58 | grey | LOC101928881 | grey | RP11-53B2.2 | grey |
| ABCC3 | blue | LINC00463 | blue | TICAM1 | blue | NR2C2 | cyan | DDR2 | darkorange | DDX60 | grey | LOC101928886 | grey | RP11-540O11.1 | grey |
| ABCC8 | blue | LINC00473 | blue | TICRR | blue | NR2F1 | cyan | DEF6 | darkorange | DECR2 | grey | LOC101928894 | grey | RP11-548H18.2 | grey |
| ABCF2 | blue | LINC00475 | blue | TIGD5 | blue | NR3C1 | cyan | DFNA5 | darkorange | DEFA5 | grey | LOC101928907 | grey | RP11-548M13.1 | grey |
| ABCG4 | blue | LINC00482 | blue | TIMM13 | blue | NR3C2 | cyan | DIP2C | darkorange | DEFA6 | grey | LOC101928917 | grey | RP11-54K16.2 | grey |
| ABCG8 | blue | LINC00494 | blue | TIMM17B | blue | NRD1 | cyan | DLC1 | darkorange | DEFB1 | grey | LOC101928937 | grey | RP11-552M11.8 | grey |
| ABHD1 | blue | LINC00514 | blue | TIMM23B | blue | NRG1 | cyan | DLGAP4 | darkorange | DEFB108B | grey | LOC101928940 | grey | RP11-567I13.1 | grey |
| ABHD13 | blue | LINC00521 | blue | TIMM44 | blue | NRIP1 | cyan | DNAJC18 | darkorange | DEFB118 | grey | LOC101928943 | grey | RP11-568N6.1 | grey |
| ABHD16A | blue | LINC00523 | blue | TIMM50 | blue | NSA2 | cyan | DNM1 | darkorange | DEFB121 | grey | LOC101928954 | grey | RP11-573N10.1 | grey |
| ABHD16B | blue | LINC00527 | blue | TINCR | blue | NSG1 | cyan | DNM3OS | darkorange | DEFB125 | grey | LOC101928968 | grey | RP11-585P4.5 | grey |
| ABLIM2 | blue | LINC00544 | blue | TK1 | blue | NSMCE4A | cyan | DOK5 | darkorange | DEFB126 | grey | LOC101928978 | grey | RP11-587D21.4 | grey |
| ABT1 | blue | LINC00565 | blue | TLDC2 | blue | NT5C3A | cyan | DPT | darkorange | DEFB129 | grey | LOC101928988 | grey | RP11-589P10.5 | grey |
| ABTB1 | blue | LINC00566 | blue | TLE6 | blue | NT5DC1 | cyan | DPYSL2 | darkorange | DEFB132 | grey | LOC101928989 | grey | RP11-59H7.3 | grey |
| ABTB2 | blue | LINC00598 | blue | TLK1 | blue | NTN4 | cyan | DPYSL3 | darkorange | DENND1C | grey | LOC101929007 | grey | RP11-5C23.1 | grey |
| AC003989.4 | blue | LINC00608 | blue | TLX2 | blue | NUBP1 | cyan | DZIP1 | darkorange | DENND2D | grey | LOC101929025 | grey | RP11-60A24.3 | grey |
| AC004941.5 | blue | LINC00620 | blue | TM4SF1 | blue | NUCKS1 | cyan | EBF1 | darkorange | DENND3 | grey | LOC101929034 | grey | RP11-61L19.2 | grey |
| AC005256.1 | blue | LINC00628 | blue | TM4SF20 | blue | NUDT19 | cyan | EBF3 | darkorange | DEPDC5 | grey | LOC101929040 | grey | RP11-631N16.2 | grey |
| AC005306.3 | blue | LINC00642 | blue | TM4SF5 | blue | NUDT21 | cyan | ECM2 | darkorange | DERA | grey | LOC101929050 | grey | RP11-634B7.4 | grey |
| AC005498.3 | blue | LINC00652 | blue | TM6SF2 | blue | NUDT3 | cyan | ECSCR | darkorange | DES | grey | LOC101929064 | grey | RP11-63A1.2 | grey |
| AC005523.3 | blue | LINC00658 | blue | TM7SF2 | blue | NUDT9P1 | cyan | EDIL3 | darkorange | DET1 | grey | LOC101929109 | grey | RP11-63A11.1 | grey |
| AC005606.14 | blue | LINC00659 | blue | TMCC2 | blue | NUFIP2 | cyan | EFEMP1 | darkorange | DEXI | grey | LOC101929114 | grey | RP11-644F5.11 | grey |
| AC005785.2 | blue | LINC00661 | blue | TMED1 | blue | NUP160 | cyan | EFEMP2 | darkorange | DFNB31 | grey | LOC101929116 | grey | RP11-646E18.4 | grey |
| AC006538.1 | blue | LINC00672 | blue | TMEM101 | blue | NUP43 | cyan | EFHD1 | darkorange | DFNB59 | grey | LOC101929118 | grey | RP11-646J21.6 | grey |
| AC007362.3 | blue | LINC00682 | blue | TMEM102 | blue | NUP62CL | cyan | EFNB2 | darkorange | DGAT1 | grey | LOC101929122 | grey | RP11-649A18.7 | grey |
| AC007365.3 | blue | LINC00689 | blue | TMEM104 | blue | NUPL2 | cyan | ELF3 | darkorange | DGCR11 | grey | LOC101929123 | grey | RP11-650K20.3 | grey |
| AC007787.2 | blue | LINC00851 | blue | TMEM105 | blue | NUTM1 | cyan | ELK3 | darkorange | DGKD | grey | LOC101929133 | grey | RP11-65D24.2 | grey |
| AC008753.4 | blue | LINC00868 | blue | TMEM108 | blue | NUTM2A-AS1 | cyan | ELN | darkorange | DGKI | grey | LOC101929147 | grey | RP11-669I1.1 | grey |
| AC013463.2 | blue | LINC00880 | blue | TMEM110 | blue | NXF1 | cyan | ELTD1 | darkorange | DGKQ | grey | LOC101929148 | grey | RP11-669M16.1 | grey |
| AC017104.6 | blue | LINC00881 | blue | TMEM114 | blue | NXNL1 | cyan | EMCN | darkorange | DHCR24 | grey | LOC101929153 | grey | RP11-673E11.2 | grey |
| AC018755.16 | blue | LINC00895 | blue | TMEM121 | blue | NXT1 | cyan | EMILIN2 | darkorange | DHCR7 | grey | LOC101929164 | grey | RP11-676J12.4 | grey |
| AC018766.6 | blue | LINC00906 | blue | TMEM129 | blue | NYNRIN | cyan | EMP1 | darkorange | DHDDS | grey | LOC101929167 | grey | RP11-678G15.1 | grey |
| AC025442.3 | blue | LINC00908 | blue | TMEM132A | blue | OAT | cyan | EMP2 | darkorange | DHFRL1 | grey | LOC101929243 | grey | RP11-680C21.1 | grey |
| AC068831.3 | blue | LINC00917 | blue | TMEM132E | blue | OAZ1 | cyan | EMP3 | darkorange | DHPS | grey | LOC101929255 | grey | RP11-680F20.10 | grey |
| AC073321.4 | blue | LINC00925 | blue | TMEM134 | blue | OCIAD1 | cyan | ENG | darkorange | DHRS1 | grey | LOC101929260 | grey | RP11-680F8.4 | grey |
| AC074212.6 | blue | LINC00926 | blue | TMEM135 | blue | OFD1 | cyan | ENPP2 | darkorange | DHRS11 | grey | LOC101929261 | grey | RP11-680G24.5 | grey |
| AC084219.3 | blue | LINC00927 | blue | TMEM141 | blue | OGFOD1 | cyan | ENPP6 | darkorange | DHRS4-AS1 | grey | LOC101929279 | grey | RP11-68I3.11 | grey |
| AC104667.3 | blue | LINC00937 | blue | TMEM151A | blue | OGFRL1 | cyan | EPAS1 | darkorange | DHRS7B | grey | LOC101929280 | grey | RP11-692P14.1 | grey |
| AC114730.11 | blue | LINC00939 | blue | TMEM151B | blue | OGT | cyan | EPDR1 | darkorange | DHRS9 | grey | LOC101929284 | grey | RP11-69I8.2 | grey |
| AC141928.1 | blue | LINC00998 | blue | TMEM160 | blue | OIP5-AS1 | cyan | EPHA3 | darkorange | DHX33 | grey | LOC101929288 | grey | RP11-6F2.5 | grey |
| AC144652.1 | blue | LINC01003 | blue | TMEM161A | blue | OMA1 | cyan | EPHB1 | darkorange | DIO2 | grey | LOC101929289 | grey | RP11-6I2.3 | grey |
| AC145343.2 | blue | LINC01004 | blue | TMEM170A | blue | OPTN | cyan | EPHX1 | darkorange | DIRC3 | grey | LOC101929297 | grey | RP11-708J19.1 | grey |
| ACAD10 | blue | LINC01010 | blue | TMEM175 | blue | OR10D3 | cyan | EPS8 | darkorange | DIS3L | grey | LOC101929312 | grey | RP11-710C12.1 | grey |
| ACADM | blue | LINC01023 | blue | TMEM177 | blue | OR2M4 | cyan | ESYT1 | darkorange | DISP1 | grey | LOC101929325 | grey | RP11-727A23.11 | grey |
| ACADS | blue | LINC01056 | blue | TMEM178B | blue | OR7E156P | cyan | EVC | darkorange | DKFZP586I1420 | grey | LOC101929328 | grey | RP11-728F11.4 | grey |
| ACAN | blue | LINC01082 | blue | TMEM179 | blue | OR8B8 | cyan | EXTL2 | darkorange | DKFZp434E1119 | grey | LOC101929335 | grey | RP11-732A19.1 | grey |
| ACAP1 | blue | LINC01101 | blue | TMEM184A | blue | OR8D1 | cyan | EYA1 | darkorange | DKFZp451B082 | grey | LOC101929340 | grey | RP11-73M18.7 | grey |
| ACHE | blue | LINC01104 | blue | TMEM184C | blue | ORC3 | cyan | EZH1 | darkorange | DKFZp564H213 | grey | LOC101929406 | grey | RP11-73M18.8 | grey |
| ACOT11 | blue | LINC01107 | blue | TMEM186 | blue | ORMDL1 | cyan | F13A1 | darkorange | DKFZp667F0711 | grey | LOC101929410 | grey | RP11-740C1.2 | grey |
| ACOT7 | blue | LINC01118 | blue | TMEM190 | blue | OSBPL1A | cyan | F8 | darkorange | DKK1 | grey | LOC101929441 | grey | RP11-742B18.1 | grey |
| ACOT8 | blue | LINC01120 | blue | TMEM191A | blue | OSBPL8 | cyan | FABP5 | darkorange | DKK2 | grey | LOC101929456 | grey | RP11-748H22.1 | grey |
| ACOXL | blue | LINC01121 | blue | TMEM196 | blue | OSBPL9 | cyan | FAM127A | darkorange | DKK3 | grey | LOC101929459 | grey | RP11-753A21.2 | grey |
| ACRBP | blue | LINC01134 | blue | TMEM198 | blue | OSMR | cyan | FAM13A | darkorange | DKK4 | grey | LOC101929460 | grey | RP11-753D20.4 | grey |
| ACSM1 | blue | LINC01137 | blue | TMEM200C | blue | OSTC | cyan | FAM65A | darkorange | DLEU1 | grey | LOC101929464 | grey | RP11-75C9.1 | grey |
| ACSM2B | blue | LINC01144 | blue | TMEM221 | blue | OSTM1-AS1 | cyan | FAM84A | darkorange | DLEU2 | grey | LOC101929465 | grey | RP11-764E7.1 | grey |
| ACTL10 | blue | LINC01159 | blue | TMEM238 | blue | OTUD6B-AS1 | cyan | FAT4 | darkorange | DLEU7-AS1 | grey | LOC101929473 | grey | RP11-769O8.3 | grey |
| ACTL6B | blue | LINC01169 | blue | TMEM243 | blue | OXA1L | cyan | FBLN1 | darkorange | DLG2 | grey | LOC101929475 | grey | RP11-774O3.3 | grey |
| ACTL7A | blue | LINC01187 | blue | TMEM25 | blue | OXR1 | cyan | FBLN5 | darkorange | DLG3-AS1 | grey | LOC101929480 | grey | RP11-787B4.2 | grey |
| ACTL7B | blue | LINC01191 | blue | TMEM254 | blue | P2RY2 | cyan | FBN1 | darkorange | DLGAP1-AS2 | grey | LOC101929488 | grey | RP11-791M20.1 | grey |
| ACTL8 | blue | LINC01204 | blue | TMEM255B | blue | PA2G4 | cyan | FBXL7 | darkorange | DLGAP2-AS1 | grey | LOC101929497 | grey | RP11-79P5.9 | grey |
| ACTL9 | blue | LINC01209 | blue | TMEM262 | blue | PABPC1 | cyan | FCER1G | darkorange | DLGAP5 | grey | LOC101929504 | grey | RP11-804H8.6 | grey |
| ACTR3 | blue | LINC01226 | blue | TMEM38A | blue | PABPC3 | cyan | FCGR2B | darkorange | DLK2 | grey | LOC101929505 | grey | RP11-806L2.2 | grey |
| ACTRT2 | blue | LINC01241 | blue | TMEM40 | blue | PACRG | cyan | FER | darkorange | DLL1 | grey | LOC101929511 | grey | RP11-82L18.2 | grey |
| ACY1 | blue | LINC01260 | blue | TMEM50B | blue | PAEP | cyan | FERMT2 | darkorange | DLST | grey | LOC101929529 | grey | RP11-846E15.4 | grey |
| ACY3 | blue | LINC01271 | blue | TMEM51-AS1 | blue | PAFAH1B1 | cyan | FGFR1 | darkorange | DLX3 | grey | LOC101929550 | grey | RP11-84D1.2 | grey |
| ADAD2 | blue | LINC01314 | blue | TMEM53 | blue | PAIP1 | cyan | FIBIN | darkorange | DLX5 | grey | LOC101929586 | grey | RP11-862L9.3 | grey |
| ADAM11 | blue | LINC01339 | blue | TMEM59L | blue | PAIP2 | cyan | FKBP9 | darkorange | DLX6-AS1 | grey | LOC101929592 | grey | RP11-874J12.4 | grey |
| ADAM8 | blue | LINC01341 | blue | TMEM62 | blue | PAK2 | cyan | FN1 | darkorange | DMBX1 | grey | LOC101929631 | grey | RP11-893F2.14 | grey |
| ADAMTS10 | blue | LINC01342 | blue | TMEM63A | blue | PAK6 | cyan | FOXP2 | darkorange | DMC1 | grey | LOC101929645 | grey | RP11-91I8.1 | grey |
| ADAMTS12 | blue | LINC01348 | blue | TMEM63B | blue | PALLD | cyan | FOXRED2 | darkorange | DMKN | grey | LOC101929648 | grey | RP11-932O9.10 | grey |
| ADAMTS13 | blue | LINC01350 | blue | TMEM63C | blue | PAM | cyan | FPR3 | darkorange | DMRT1 | grey | LOC101929657 | grey | RP11-932O9.4 | grey |
| ADAMTS14 | blue | LINC01405 | blue | TMEM65 | blue | PAN3 | cyan | FRMD5 | darkorange | DMRTA2 | grey | LOC101929662 | grey | RP11-945A11.2 | grey |
| ADAMTS16 | blue | LINC01428 | blue | TMEM86B | blue | PANK3 | cyan | FSTL1 | darkorange | DMTN | grey | LOC101929668 | grey | RP11-960B9.2 | grey |
| ADAMTS7 | blue | LINGO1 | blue | TMEM87B | blue | PAPOLA | cyan | FTL | darkorange | DMWD | grey | LOC101929683 | grey | RP11-96D1.11 | grey |
| ADAMTS8 | blue | LINGO3 | blue | TMEM8B | blue | PAPSS1 | cyan | FUT6 | darkorange | DNA2 | grey | LOC101929684 | grey | RP11-96K19.4 | grey |
| ADAMTSL2 | blue | LIPE-AS1 | blue | TMEM92 | blue | PARD3-AS1 | cyan | FZD4 | darkorange | DNAAF3 | grey | LOC101929687 | grey | RP11-97C16.1 | grey |
| ADAMTSL5 | blue | LKAAEAR1 | blue | TMF1 | blue | PARK7 | cyan | GALNT15 | darkorange | DNAH8 | grey | LOC101929715 | grey | RP11-999E24.3 | grey |
| ADAP1 | blue | LMBRD1 | blue | TMIE | blue | PARN | cyan | GAS1 | darkorange | DNAJB1 | grey | LOC101929717 | grey | RP13-20L14.1 | grey |
| ADCK1 | blue | LMNTD2 | blue | TMOD3 | blue | PATL1 | cyan | GAS7 | darkorange | DNAJB5 | grey | LOC101929719 | grey | RP13-270P17.3 | grey |
| ADCK4 | blue | LMO1 | blue | TMPO | blue | PAX4 | cyan | GBE1 | darkorange | DNAJB8-AS1 | grey | LOC101929741 | grey | RP13-379O24.2 | grey |
| ADCK5 | blue | LMOD2 | blue | TMPRSS5 | blue | PBX1 | cyan | GBP2 | darkorange | DNAJC17 | grey | LOC101929747 | grey | RP1L1 | grey |
| ADCY1 | blue | LMTK2 | blue | TMPRSS6 | blue | PBX3 | cyan | GEM | darkorange | DNAJC24 | grey | LOC101929752 | grey | RP3-333B15.4 | grey |
| ADCY8 | blue | LOC100126784 | blue | TMTC3 | blue | PCBP1 | cyan | GFPT2 | darkorange | DNAJC28 | grey | LOC101929757 | grey | RP3-334F4.2 | grey |
| ADK | blue | LOC100128239 | blue | TMUB1 | blue | PCBP2 | cyan | GHR | darkorange | DNAJC5G | grey | LOC101929760 | grey | RP3-359N14.1 | grey |
| ADM2 | blue | LOC100128325 | blue | TMX1 | blue | PCDH11X | cyan | GIMAP6 | darkorange | DNAJC9 | grey | LOC101929761 | grey | RP3-388M5.9 | grey |
| ADM5 | blue | LOC100128343 | blue | TMX3 | blue | PCDHB17 | cyan | GJA1 | darkorange | DNAJC9-AS1 | grey | LOC101929765 | grey | RP3-406A7.7 | grey |
| ADORA1 | blue | LOC100128644 | blue | TNFRSF12A | blue | PCDHB8 | cyan | GNA12 | darkorange | DNAL1 | grey | LOC101929774 | grey | RP3-412A9.16 | grey |
| ADRA1B | blue | LOC100128653 | blue | TNFRSF13B | blue | PCDP1 | cyan | GNAI2 | darkorange | DNAL4 | grey | LOC101929787 | grey | RP3-428L16.2 | grey |
| ADRA1D | blue | LOC100129112 | blue | TNFRSF18 | blue | PCF11 | cyan | GNG11 | darkorange | DNASE1L1 | grey | LOC101929897 | grey | RP3-476K8.3 | grey |
| ADRA2B | blue | LOC100129175 | blue | TNFRSF25 | blue | PCGEM1 | cyan | GNG2 | darkorange | DNASE1L3 | grey | LOC101929964 | grey | RP3-486D24.1 | grey |
| ADRA2C | blue | LOC100129476 | blue | TNFRSF4 | blue | PCGF5 | cyan | GOLIM4 | darkorange | DNM1P46 | grey | LOC101929988 | grey | RP3-496C20.1 | grey |
| ADRB3 | blue | LOC100129516 | blue | TNFRSF8 | blue | PCM1 | cyan | GPC6 | darkorange | DNM3 | grey | LOC101930097 | grey | RP3-497J21.1 | grey |
| ADSL | blue | LOC100129722 | blue | TNFRSF9 | blue | PCMT1 | cyan | GPNMB | darkorange | DNMT1 | grey | LOC101930541 | grey | RP3-508I15.21 | grey |
| ADSSL1 | blue | LOC100129935 | blue | TNFSF14 | blue | PCMTD1 | cyan | GPR116 | darkorange | DNTTIP1 | grey | LOC101930657 | grey | RP3-525N10.2 | grey |
| AEN | blue | LOC100130232 | blue | TNIP1 | blue | PCNP | cyan | GPR124 | darkorange | DOCK10 | grey | LOC102467079 | grey | RP4-545K15.5 | grey |
| AF067845.1 | blue | LOC100130285 | blue | TNK1 | blue | PCNX | cyan | GPR133 | darkorange | DOCK2 | grey | LOC102546226 | grey | RP4-575N6.5 | grey |
| AF213884.2 | blue | LOC100130370 | blue | TNKS2 | blue | PCYOX1 | cyan | GPR34 | darkorange | DOCK3 | grey | LOC102546294 | grey | RP4-581F12.1 | grey |
| AFAP1-AS1 | blue | LOC100130417 | blue | TNNI1 | blue | PDAP1 | cyan | GPR98 | darkorange | DOCK7 | grey | LOC102606465 | grey | RP4-584D14.7 | grey |
| AFF4 | blue | LOC100130502 | blue | TNNT1 | blue | PDCD6IP | cyan | GPX1 | darkorange | DOCK8 | grey | LOC102659288 | grey | RP4-594L9.2 | grey |
| AFMID | blue | LOC100130654 | blue | TNNT2 | blue | PDE3B | cyan | GPX8 | darkorange | DOK3 | grey | LOC102723380 | grey | RP4-647C14.3 | grey |
| AGAP2 | blue | LOC100130700 | blue | TNP1 | blue | PDE4D | cyan | GRAMD1C | darkorange | DOLK | grey | LOC102723542 | grey | RP4-657D16.3 | grey |
| AGBL4 | blue | LOC100130815 | blue | TNPO2 | blue | PDGFC | cyan | GRB7 | darkorange | DONSON | grey | LOC102723620 | grey | RP4-665N4.4 | grey |
| AGER | blue | LOC100130938 | blue | TOB1 | blue | PDHA1 | cyan | GRIP1 | darkorange | DPCR1 | grey | LOC102723692 | grey | RP4-710M16.1 | grey |
| AGK | blue | LOC100130950 | blue | TOB1-AS1 | blue | PDHB | cyan | GRK6 | darkorange | DPEP2 | grey | LOC102723697 | grey | RP4-714D9.5 | grey |
| AGRP | blue | LOC100131170 | blue | TOM1 | blue | PDILT | cyan | GSTM3 | darkorange | DPF2 | grey | LOC102723742 | grey | RP4-730D4.1 | grey |
| AGXT | blue | LOC100131347 | blue | TOMM40 | blue | PDLIM1 | cyan | GSTM4 | darkorange | DPH2 | grey | LOC102723757 | grey | RP4-813F11.4 | grey |
| AHCTF1 | blue | LOC100131532 | blue | TONSL | blue | PDP1 | cyan | GSTM5 | darkorange | DPH3P1 | grey | LOC102723779 | grey | RP5-1039K5.17 | grey |
| AHI1 | blue | LOC100131662 | blue | TOP3B | blue | PDPR | cyan | GSTO2 | darkorange | DPM3 | grey | LOC102723847 | grey | RP5-1065J22.8 | grey |
| AHSA1 | blue | LOC100131864 | blue | TOPBP1 | blue | PDS5A | cyan | GUCY1A2 | darkorange | DPP3 | grey | LOC102723886 | grey | RP5-1068B5.3 | grey |
| AHSP | blue | LOC100133461 | blue | TOR1AIP2 | blue | PDYN | cyan | GULP1 | darkorange | DPP6 | grey | LOC102723918 | grey | RP5-1074L1.4 | grey |
| AIFM3 | blue | LOC100147773 | blue | TOR2A | blue | PDZD11 | cyan | GXYLT2 | darkorange | DPPA5 | grey | LOC102723927 | grey | RP5-1098D14.1 | grey |
| AIM1L | blue | LOC100240734 | blue | TOR3A | blue | PDZD8 | cyan | GYPC | darkorange | DPY19L2 | grey | LOC102723932 | grey | RP5-1103G7.10 | grey |
| AIPL1 | blue | LOC100270804 | blue | TP53I11 | blue | PEBP1 | cyan | HDGFRP3 | darkorange | DPY19L2P2 | grey | LOC102723954 | grey | RP5-1119A7.11 | grey |
| AIRE | blue | LOC100286925 | blue | TP53I13 | blue | PER2 | cyan | HEG1 | darkorange | DQ570835 | grey | LOC102724009 | grey | RP5-1136G13.2 | grey |
| AK055458 | blue | LOC100287098 | blue | TP53TG5 | blue | PEX11B | cyan | HGSNAT | darkorange | DQ576800 | grey | LOC102724030 | grey | RP5-1154L15.2 | grey |
| AK056098 | blue | LOC100287210 | blue | TP73 | blue | PEX19 | cyan | HIF3A | darkorange | DQ581328 | grey | LOC102724156 | grey | RP5-1170D6.1 | grey |
| AK074476 | blue | LOC100287221 | blue | TPD52L3 | blue | PEX2 | cyan | HLF | darkorange | DQ582785 | grey | LOC102724162 | grey | RP5-1189K21.2 | grey |
| AK093205 | blue | LOC100287497 | blue | TPGS1 | blue | PFDN2 | cyan | HNRNPA0 | darkorange | DQ583756 | grey | LOC102724165 | grey | RP5-855D21.1 | grey |
| AK097370 | blue | LOC100287525 | blue | TPM4 | blue | PFKFB3 | cyan | HPGDS | darkorange | DQ586822 | grey | LOC102724201 | grey | RP5-856G1.1 | grey |
| AK097453 | blue | LOC100287704 | blue | TPO | blue | PFN1 | cyan | HTRA1 | darkorange | DQ592442 | grey | LOC102724275 | grey | RP5-890E16.2 | grey |
| AKAP4 | blue | LOC100287808 | blue | TPPP | blue | PFN2 | cyan | ICAM3 | darkorange | DQ594366 | grey | LOC102724316 | grey | RP5-892K4.1 | grey |
| AKAP5 | blue | LOC100288123 | blue | TPSD1 | blue | PGAM2 | cyan | IFIT3 | darkorange | DRAM1 | grey | LOC102724387 | grey | RP5-894A10.6 | grey |
| AKAP6 | blue | LOC100288175 | blue | TRA2B | blue | PGC | cyan | IFITM2 | darkorange | DRAP1 | grey | LOC102724487 | grey | RP5-894D12.4 | grey |
| AKAP9 | blue | LOC100288893 | blue | TRABD | blue | PGLYRP1 | cyan | IFITM3 | darkorange | DRC1 | grey | LOC102724508 | grey | RP5-930J4.4 | grey |
| AKR1E2 | blue | LOC100289058 | blue | TRABD2A | blue | PGRMC1 | cyan | IGF1 | darkorange | DRD1 | grey | LOC102724511 | grey | RP5-935K16.1 | grey |
| AL022341.3 | blue | LOC100289061 | blue | TRADD | blue | PGRMC2 | cyan | IGFBP3 | darkorange | DRD5 | grey | LOC102724561 | grey | RP6-24A23.7 | grey |
| AL590762.11 | blue | LOC100289230 | blue | TRAF2 | blue | PHACTR2 | cyan | IGFBP4 | darkorange | DSC1 | grey | LOC102724587 | grey | RP6-91H8.1 | grey |
| ALCAM | blue | LOC100289283 | blue | TRAF5 | blue | PHB2 | cyan | IGFBP5 | darkorange | DSC3 | grey | LOC102724611 | grey | RPA2 | grey |
| ALDH16A1 | blue | LOC100289361 | blue | TRAF7 | blue | PHF10 | cyan | IGFBP6 | darkorange | DSCAM-AS1 | grey | LOC102724612 | grey | RPA3 | grey |
| ALDH1L1-AS2 | blue | LOC100291666 | blue | TRAFD1 | blue | PHF12 | cyan | IGFBP7 | darkorange | DSCR10 | grey | LOC102724689 | grey | RPA3OS | grey |
| ALDH3A1 | blue | LOC100294145 | blue | TRBV27 | blue | PHF21B | cyan | IGHG1 | darkorange | DSE | grey | LOC102724782 | grey | RPA4 | grey |
| ALDH3B1 | blue | LOC100499489 | blue | TREH | blue | PHF3 | cyan | IL17RD | darkorange | DSPP | grey | LOC102724809 | grey | RPAP1 | grey |
| ALDOB | blue | LOC100505540 | blue | TREM2 | blue | PHF5A | cyan | IL1R1 | darkorange | DSTYK | grey | LOC102724814 | grey | RPARP-AS1 | grey |
| ALG1 | blue | LOC100505592 | blue | TRIB2 | blue | PHYH | cyan | IL33 | darkorange | DTWD2 | grey | LOC102724842 | grey | RPGR | grey |
| ALG6 | blue | LOC100505664 | blue | TRIB3 | blue | PIAS1 | cyan | IQSEC1 | darkorange | DTX1 | grey | LOC102724870 | grey | RPGRIP1 | grey |
| ALK | blue | LOC100505666 | blue | TRIM11 | blue | PICALM | cyan | IRAK3 | darkorange | DUOX1 | grey | LOC102724938 | grey | RPH3A | grey |
| ALKBH4 | blue | LOC100505835 | blue | TRIM13 | blue | PIF1 | cyan | IRS2 | darkorange | DUOX2 | grey | LOC102725017 | grey | RPL10L | grey |
| ALKBH7 | blue | LOC100505841 | blue | TRIM15 | blue | PIGF | cyan | ITGA1 | darkorange | DUSP11 | grey | LOC102725383 | grey | RPL18 | grey |
| ALMS1P | blue | LOC100505942 | blue | TRIM17 | blue | PIGK | cyan | ITGA5 | darkorange | DUSP12 | grey | LOC102725408 | grey | RPL22L1 | grey |
| ALOX12B | blue | LOC100505978 | blue | TRIM31 | blue | PIGO | cyan | ITGAM | darkorange | DUSP14 | grey | LOC102725438 | grey | RPL23AP32 | grey |
| ALPI | blue | LOC100506114 | blue | TRIM38 | blue | PIGP | cyan | ITGB5 | darkorange | DUSP23 | grey | LOC142937 | grey | RPL27A | grey |
| ALPP | blue | LOC100506142 | blue | TRIM44 | blue | PIGV | cyan | ITGBL1 | darkorange | DUSP28 | grey | LOC145678 | grey | RPL32P3 | grey |
| ALPPL2 | blue | LOC100506175 | blue | TRIM46 | blue | PIK3C2A | cyan | ITPRIPL2 | darkorange | DUSP4 | grey | LOC145845 | grey | RPL39L | grey |
| ALS2CL | blue | LOC100506271 | blue | TRIM5 | blue | PIK3CB | cyan | ITSN1 | darkorange | DUSP5 | grey | LOC148696 | grey | RPL7AL2 | grey |
| ALX3 | blue | LOC100506274 | blue | TRIM50 | blue | PIK3R1 | cyan | JAG1 | darkorange | DUSP7 | grey | LOC148709 | grey | RPLP2 | grey |
| AMBP | blue | LOC100506286 | blue | TRIM54 | blue | PIN4 | cyan | JAM2 | darkorange | DUXAP10 | grey | LOC149373 | grey | RPP25L | grey |
| AMER1 | blue | LOC100506446 | blue | TRIM62 | blue | PITPNB | cyan | JAM3 | darkorange | DVL1 | grey | LOC149703 | grey | RPP38 | grey |
| AMHR2 | blue | LOC100506472 | blue | TRIM63 | blue | PITRM1 | cyan | JDP2 | darkorange | DXO | grey | LOC150051 | grey | RPRM | grey |
| AMICA1 | blue | LOC100506504 | blue | TRIM69 | blue | PJA2 | cyan | KCNE3 | darkorange | DYDC1 | grey | LOC151657 | grey | RPS10L | grey |
| AMIGO2 | blue | LOC100506557 | blue | TRIM72 | blue | PKD2 | cyan | KCTD20 | darkorange | DYDC2 | grey | LOC152225 | grey | RPS10P7 | grey |
| AMN | blue | LOC100506571 | blue | TRIML1 | blue | PKD2L2 | cyan | KDR | darkorange | DYNC1I1 | grey | LOC152586 | grey | RPS15 | grey |
| AMZ1 | blue | LOC100506679 | blue | TRIML2 | blue | PKIG | cyan | KIAA1377 | darkorange | DYNC2H1 | grey | LOC153682 | grey | RPS16 | grey |
| ANAPC10 | blue | LOC100506791 | blue | TRIQK | blue | PKLR | cyan | KIAA1462 | darkorange | DYNLRB2 | grey | LOC153910 | grey | RPS16P5 | grey |
| ANAPC13 | blue | LOC100506813 | blue | TRMT1 | blue | PKN2 | cyan | KIFAP3 | darkorange | DYRK3 | grey | LOC154761 | grey | RPS19BP1 | grey |
| ANGEL2 | blue | LOC100507006 | blue | TRMT11 | blue | PLA2G12A | cyan | KIRREL | darkorange | DYRK4 | grey | LOC154872 | grey | RPS23 | grey |
| ANGPTL4 | blue | LOC100507033 | blue | TRMT12 | blue | PLCB4 | cyan | KITLG | darkorange | DYSF | grey | LOC155060 | grey | RPS3 | grey |
| ANKFN1 | blue | LOC100507217 | blue | TRMT2A | blue | PLEKHA2 | cyan | KLF4 | darkorange | DYX1C1 | grey | LOC157562 | grey | RPS4Y1 | grey |
| ANKMY1 | blue | LOC100507291 | blue | TRMT61A | blue | PLEKHA5 | cyan | KLF6 | darkorange | Dbpht2 | grey | LOC158402 | grey | RPS6KA1 | grey |
| ANKRD10 | blue | LOC100507377 | blue | TRMT61B | blue | PLEKHB2 | cyan | LAIR1 | darkorange | E2F1 | grey | LOC158960 | grey | RPS6KA2-AS1 | grey |
| ANKRD13C | blue | LOC100507391 | blue | TRMU | blue | PLRG1 | cyan | LAMB1 | darkorange | E2F2 | grey | LOC200609 | grey | RPS9 | grey |
| ANKRD13D | blue | LOC100507494 | blue | TROAP | blue | PMCHL2 | cyan | LAMB2 | darkorange | E2F5 | grey | LOC202025 | grey | RRM1 | grey |
| ANKRD2 | blue | LOC100507507 | blue | TRPC2 | blue | PMS2P1 | cyan | LAMC1 | darkorange | E2F6 | grey | LOC219688 | grey | RRM2 | grey |
| ANKRD20A12P | blue | LOC100507513 | blue | TRPC4AP | blue | PNISR | cyan | LATS2 | darkorange | EAF1 | grey | LOC219690 | grey | RRM2B | grey |
| ANKRD24 | blue | LOC100507562 | blue | TRPM2 | blue | PNMAL2 | cyan | LDB2 | darkorange | EBF2 | grey | LOC221122 | grey | RRN3P2 | grey |
| ANKRD34A | blue | LOC100507630 | blue | TRPM2-AS | blue | PNPLA8 | cyan | LEPR | darkorange | EBLN2 | grey | LOC221272 | grey | RRNAD1 | grey |
| ANKRD35 | blue | LOC100507656 | blue | TRPM4 | blue | PNRC1 | cyan | LGMN | darkorange | EBPL | grey | LOC221946 | grey | RRS1-AS1 | grey |
| ANKRD36 | blue | LOC100507670 | blue | TRPM5 | blue | PNRC2 | cyan | LHFP | darkorange | ECH1 | grey | LOC253044 | grey | RSAD2 | grey |
| ANKRD39 | blue | LOC100630918 | blue | TRPM7 | blue | POGK | cyan | LILRB1 | darkorange | ECHDC2 | grey | LOC254057 | grey | RSL1D1 | grey |
| ANKRD53 | blue | LOC100652768 | blue | TRPM8 | blue | POGZ | cyan | LILRB5 | darkorange | ECHDC3 | grey | LOC257152 | grey | RSPH1 | grey |
| ANKS3 | blue | LOC100653086 | blue | TRPV3 | blue | POLA1 | cyan | LINC00883 | darkorange | ECI1 | grey | LOC257396 | grey | RSPH9 | grey |
| ANKS6 | blue | LOC100996246 | blue | TRPV4 | blue | POLD1 | cyan | LINC01279 | darkorange | ECM1 | grey | LOC283194 | grey | RSPO2 | grey |
| ANO9 | blue | LOC100996263 | blue | TRPV6 | blue | POLE3 | cyan | LLGL2 | darkorange | EDARADD | grey | LOC283214 | grey | RTBDN | grey |
| ANP32C | blue | LOC100996342 | blue | TRUB2 | blue | POLI | cyan | LMTK3 | darkorange | EDEM1 | grey | LOC283387 | grey | RTDR1 | grey |
| ANXA13 | blue | LOC100996654 | blue | TSC22D4 | blue | POLR1D | cyan | LOC100505938 | darkorange | EDEM2 | grey | LOC283454 | grey | RTN1 | grey |
| AOAH | blue | LOC100996671 | blue | TSEN15 | blue | POLR1E | cyan | LOC101930405 | darkorange | EDN1 | grey | LOC283585 | grey | RTN4IP1 | grey |
| AOC4P | blue | LOC100996843 | blue | TSEN54 | blue | POLR2I | cyan | LOC102723845 | darkorange | EDNRB | grey | LOC283588 | grey | RTN4RL2 | grey |
| AP000265.1 | blue | LOC101059948 | blue | TSGA10IP | blue | POLR2J | cyan | LOC400043 | darkorange | EEF1A1P42 | grey | LOC283665 | grey | RTP4 | grey |
| AP000347.2 | blue | LOC101060004 | blue | TSGA13 | blue | POLR2J4 | cyan | LOC654342 | darkorange | EFCAB12 | grey | LOC283731 | grey | RUNDC3B | grey |
| AP000696.2 | blue | LOC101060400 | blue | TSKS | blue | POLR2K | cyan | LOX | darkorange | EFCC1 | grey | LOC283737 | grey | RUNX2 | grey |
| AP001462.6 | blue | LOC101060691 | blue | TSNARE1 | blue | POLR3C | cyan | LOXL1 | darkorange | EFHB | grey | LOC283788 | grey | RUNX3 | grey |
| AP001630.5 | blue | LOC101926934 | blue | TSPAN10 | blue | POLR3E | cyan | LPAR1 | darkorange | EFNA1 | grey | LOC283856 | grey | RUSC1-AS1 | grey |
| AP006216.10 | blue | LOC101926963 | blue | TSPAN16 | blue | POM121C | cyan | LPAR3 | darkorange | EFNA3 | grey | LOC283861 | grey | RUVBL1 | grey |
| AP006547.3 | blue | LOC101927040 | blue | TSPAN32 | blue | POMP | cyan | LRRC17 | darkorange | EFNA4 | grey | LOC283914 | grey | RWDD3 | grey |
| AP1AR | blue | LOC101927051 | blue | TSPAN9 | blue | PON1 | cyan | LRRC32 | darkorange | EFNB3 | grey | LOC284014 | grey | RXFP3 | grey |
| AP1S1 | blue | LOC101927060 | blue | TSPY26P | blue | PON2 | cyan | LRRN4CL | darkorange | EFS | grey | LOC284023 | grey | RXRB | grey |
| AP2A1 | blue | LOC101927081 | blue | TSR3 | blue | POP5 | cyan | LTBP1 | darkorange | EGF | grey | LOC284263 | grey | RXRG | grey |
| AP3B1 | blue | LOC101927126 | blue | TSSC1 | blue | POT1 | cyan | LTBP2 | darkorange | EGFL6 | grey | LOC284373 | grey | RYR1 | grey |
| AP5M1 | blue | LOC101927150 | blue | TSSK1B | blue | POTEKP | cyan | LUM | darkorange | EGFLAM | grey | LOC284379 | grey | S100A12 | grey |
| APBB1 | blue | LOC101927164 | blue | TSSK2 | blue | POU3F1 | cyan | LYVE1 | darkorange | EGFLAM-AS2 | grey | LOC284395 | grey | S100A13 | grey |
| APC2 | blue | LOC101927166 | blue | TSSK3 | blue | PPA1 | cyan | MAF | darkorange | EGR1 | grey | LOC284513 | grey | S100A14 | grey |
| API5 | blue | LOC101927181 | blue | TSSK6 | blue | PPCS | cyan | MAFB | darkorange | EGR2 | grey | LOC284578 | grey | S100A2 | grey |
| APLN | blue | LOC101927247 | blue | TTC16 | blue | PPHLN1 | cyan | MAGI2-AS3 | darkorange | EGR3 | grey | LOC284632 | grey | S100A6 | grey |
| APLP1 | blue | LOC101927248 | blue | TTC25 | blue | PPIB | cyan | MAMDC2 | darkorange | EHBP1L1 | grey | LOC284648 | grey | S100A7 | grey |
| APOA4 | blue | LOC101927254 | blue | TTC36 | blue | PPIC | cyan | MAN1A1 | darkorange | EHD1 | grey | LOC284788 | grey | S100A8 | grey |
| APOBEC1 | blue | LOC101927278 | blue | TTC38 | blue | PPIG | cyan | MAN1C1 | darkorange | EHMT1-IT1 | grey | LOC284865 | grey | S100A9 | grey |
| APOBR | blue | LOC101927330 | blue | TTC39A | blue | PPIL3 | cyan | MAP1A | darkorange | EID3 | grey | LOC284898 | grey | S100G | grey |
| APOC2 | blue | LOC101927411 | blue | TTC7A | blue | PPIL4 | cyan | MAP1B | darkorange | EIF1AY | grey | LOC284933 | grey | S100Z | grey |
| APOC3 | blue | LOC101927447 | blue | TTC9B | blue | PPP1CB | cyan | MAP3K9 | darkorange | EIF2B3 | grey | LOC285000 | grey | S1PR3 | grey |
| APOF | blue | LOC101927479 | blue | TTI1 | blue | PPP1CC | cyan | MAP4 | darkorange | EIF2B4 | grey | LOC285043 | grey | SAAL1 | grey |
| APOL2 | blue | LOC101927499 | blue | TTLL10 | blue | PPP1R12A | cyan | MARC2 | darkorange | EIF2B5 | grey | LOC285178 | grey | SAC3D1 | grey |
| APOL5 | blue | LOC101927503 | blue | TTLL9 | blue | PPP1R14C | cyan | MEI1 | darkorange | EIF3C | grey | LOC285419 | grey | SAG | grey |
| APRT | blue | LOC101927507 | blue | TTR | blue | PPP1R17 | cyan | MET | darkorange | EIF3D | grey | LOC285422 | grey | SALL2 | grey |
| AQP6 | blue | LOC101927539 | blue | TUBA4B | blue | PPP1R27 | cyan | MFAP4 | darkorange | EIF3I | grey | LOC285556 | grey | SALL4 | grey |
| AQP8 | blue | LOC101927550 | blue | TUBA8 | blue | PPP1R8 | cyan | MFAP5 | darkorange | EIF4A3 | grey | LOC285627 | grey | SAMD13 | grey |
| ARAP1 | blue | LOC101927603 | blue | TUBB7P | blue | PPP2R5C | cyan | MIR100HG | darkorange | EIF6 | grey | LOC285638 | grey | SAMD15 | grey |
| ARFGAP2 | blue | LOC101927604 | blue | TUBGCP3 | blue | PPP2R5E | cyan | MIR205 | darkorange | ELAC1 | grey | LOC285740 | grey | SAMD4A | grey |
| ARFRP1 | blue | LOC101927608 | blue | TULP1 | blue | PPP3CA | cyan | MITF | darkorange | ELAVL2 | grey | LOC285768 | grey | SAMD9 | grey |
| ARHGAP27 | blue | LOC101927651 | blue | TUSC2 | blue | PPP3CB | cyan | MKNK1 | darkorange | ELF4 | grey | LOC285778 | grey | SAP30BP | grey |
| ARHGAP33 | blue | LOC101927760 | blue | TUT1 | blue | PPP3R1 | cyan | MLLT10 | darkorange | ELK2AP | grey | LOC285812 | grey | SARS2 | grey |
| ARHGAP4 | blue | LOC101927770 | blue | TVP23A | blue | PPP4R1 | cyan | MMP7 | darkorange | ELOF1 | grey | LOC285819 | grey | SASH3 | grey |
| ARHGDIG | blue | LOC101927783 | blue | TWF1 | blue | PPP4R2 | cyan | MMRN2 | darkorange | ELOVL2-AS1 | grey | LOC285857 | grey | SAT2 | grey |
| ARHGEF16 | blue | LOC101927787 | blue | TWF2 | blue | PPP6C | cyan | MN1 | darkorange | ELOVL3 | grey | LOC285889 | grey | SBF2-AS1 | grey |
| ARHGEF19 | blue | LOC101927824 | blue | TXNL1 | blue | PPT1 | cyan | MPDZ | darkorange | ELOVL4 | grey | LOC286009 | grey | SBNO2 | grey |
| ARHGEF9 | blue | LOC101927839 | blue | TXNRD1 | blue | PPTC7 | cyan | MPEG1 | darkorange | ELOVL6 | grey | LOC286058 | grey | SBSPON | grey |
| ARID2 | blue | LOC101927843 | blue | TXNRD2 | blue | PRCD | cyan | MPRIP | darkorange | ELOVL7 | grey | LOC286161 | grey | SCAI | grey |
| ARID3A | blue | LOC101927914 | blue | TYMSOS | blue | PRDM2 | cyan | MPZL3 | darkorange | ELP4 | grey | LOC286177 | grey | SCAMP1-AS1 | grey |
| ARL16 | blue | LOC101927972 | blue | TYRO3 | blue | PRDX1 | cyan | MRC1 | darkorange | ELSPBP1 | grey | LOC286178 | grey | SCAMP2 | grey |
| ARL5B | blue | LOC101928035 | blue | TYSND1 | blue | PRDX4 | cyan | MS4A4A | darkorange | EMB | grey | LOC286189 | grey | SCAMP3 | grey |
| ARL5C | blue | LOC101928069 | blue | U2AF2 | blue | PRDX6 | cyan | MS4A6A | darkorange | EMC10 | grey | LOC286238 | grey | SCAMPER | grey |
| ARMC10 | blue | LOC101928076 | blue | U2SURP | blue | PREPL | cyan | MS4A7 | darkorange | EMC3-AS1 | grey | LOC286272 | grey | SCARA3 | grey |
| ARMC5 | blue | LOC101928087 | blue | UAP1 | blue | PRICKLE1 | cyan | MSRB3 | darkorange | EMC6 | grey | LOC286367 | grey | SCARNA15 | grey |
| ARMC6 | blue | LOC101928111 | blue | UBA6 | blue | PRKACA | cyan | MTSS1 | darkorange | EMG1 | grey | LOC286370 | grey | SCARNA17 | grey |
| ARMC7 | blue | LOC101928222 | blue | UBAC2 | blue | PRKAR1A | cyan | MXRA7 | darkorange | EMILIN3 | grey | LOC286437 | grey | SCARNA2 | grey |
| ARMC9 | blue | LOC101928230 | blue | UBALD1 | blue | PRKAR2A | cyan | MYH10 | darkorange | EML1 | grey | LOC338667 | grey | SCART1 | grey |
| ARR3 | blue | LOC101928231 | blue | UBAP1L | blue | PRKCI | cyan | MYH14 | darkorange | EMR1 | grey | LOC338694 | grey | SCCPDH | grey |
| ARRDC3-AS1 | blue | LOC101928251 | blue | UBASH3A | blue | PRKCSH | cyan | MYOF | darkorange | EMR2 | grey | LOC339298 | grey | SCD | grey |
| ARSA | blue | LOC101928278 | blue | UBE2D1 | blue | PRKD3 | cyan | NAP1L3 | darkorange | EMR3 | grey | LOC339468 | grey | SCFD2 | grey |
| ARSE | blue | LOC101928284 | blue | UBE2J2 | blue | PRKDC | cyan | NAP1L5 | darkorange | EMX2 | grey | LOC339666 | grey | SCG2 | grey |
| ARSF | blue | LOC101928303 | blue | UBE2O | blue | PRKRIR | cyan | NAV1 | darkorange | EMX2OS | grey | LOC339685 | grey | SCG5 | grey |
| ARSI | blue | LOC101928304 | blue | UBE2V2 | blue | PRNP | cyan | NAV3 | darkorange | EN1 | grey | LOC339988 | grey | SCGB1A1 | grey |
| ART1 | blue | LOC101928323 | blue | UBE2W | blue | PRO0471 | cyan | NDN | darkorange | ENC1 | grey | LOC340074 | grey | SCIMP | grey |
| ARVCF | blue | LOC101928378 | blue | UBIAD1 | blue | PRODH2 | cyan | NDNF | darkorange | ENDOD1 | grey | LOC340178 | grey | SCML2 | grey |
| ASB1 | blue | LOC101928399 | blue | UBOX5 | blue | PROSC | cyan | NECAB1 | darkorange | ENDOU | grey | LOC340340 | grey | SCML4 | grey |
| ASB10 | blue | LOC101928424 | blue | UBQLN2 | blue | PROSER1 | cyan | NEGR1 | darkorange | ENO2 | grey | LOC340581 | grey | SCN10A | grey |
| ASB2 | blue | LOC101928443 | blue | UBR2 | blue | PRPS1 | cyan | NEK3 | darkorange | ENPEP | grey | LOC344887 | grey | SCN1B | grey |
| ASB6 | blue | LOC101928460 | blue | UBR3 | blue | PRR14 | cyan | NFE2L1 | darkorange | ENPP1 | grey | LOC374443 | grey | SCN3B | grey |
| ASCC3 | blue | LOC101928464 | blue | UBTD1 | blue | PRR26 | cyan | NID1 | darkorange | ENPP4 | grey | LOC375196 | grey | SCN4A | grey |
| ASCL5 | blue | LOC101928521 | blue | UBTF | blue | PRR30 | cyan | NID2 | darkorange | ENTPD1 | grey | LOC388942 | grey | SCN4B | grey |
| ASF1A | blue | LOC101928612 | blue | UBXN1 | blue | PRRC1 | cyan | NOX4 | darkorange | ENTPD3-AS1 | grey | LOC389247 | grey | SCN5A | grey |
| ASGR1 | blue | LOC101928651 | blue | UBXN2A | blue | PRRC2C | cyan | NPC2 | darkorange | ENTPD7 | grey | LOC389641 | grey | SCN7A | grey |
| ASGR2 | blue | LOC101928682 | blue | UBXN2B | blue | PRSS22 | cyan | NR2F2 | darkorange | EOGT | grey | LOC389765 | grey | SCN9A | grey |
| ASH2L | blue | LOC101928697 | blue | UCK1 | blue | PRSS36 | cyan | NRN1 | darkorange | EOMES | grey | LOC389831 | grey | SCO1 | grey |
| ASIC2 | blue | LOC101928707 | blue | UCKL1 | blue | PRSS53 | cyan | NRP1 | darkorange | EP300-AS1 | grey | LOC389834 | grey | SCO2 | grey |
| ASIC4 | blue | LOC101928729 | blue | UCN | blue | PSG9 | cyan | NT5E | darkorange | EPB41L1 | grey | LOC389895 | grey | SCOC | grey |
| ASIP | blue | LOC101928762 | blue | UCN2 | blue | PSIP1 | cyan | NTAN1 | darkorange | EPB41L4A-AS2 | grey | LOC399491 | grey | SCPEP1 | grey |
| ASMT | blue | LOC101928787 | blue | UCN3 | blue | PSMA1 | cyan | NTRK2 | darkorange | EPB41L4B | grey | LOC399900 | grey | SCRG1 | grey |
| ASMTL-AS1 | blue | LOC101928837 | blue | UCP3 | blue | PSMA4 | cyan | NUAK1 | darkorange | EPB41L5 | grey | LOC400620 | grey | SCRIB | grey |
| ASPDH | blue | LOC101928844 | blue | UEVLD | blue | PSMB1 | cyan | NUP210 | darkorange | EPB42 | grey | LOC400622 | grey | SCRN1 | grey |
| ASPG | blue | LOC101928845 | blue | UFSP1 | blue | PSMB2 | cyan | OGN | darkorange | EPC2 | grey | LOC400748 | grey | SCUBE2 | grey |
| ASPHD1 | blue | LOC101928869 | blue | UGCG | blue | PSMB4 | cyan | OLFML1 | darkorange | EPHA2 | grey | LOC400756 | grey | SCUBE3 | grey |
| ATAD3A | blue | LOC101928896 | blue | UHRF2 | blue | PSMC2 | cyan | OLFML2B | darkorange | EPHA5 | grey | LOC400794 | grey | SCYL3 | grey |
| ATAD3B | blue | LOC101929004 | blue | ULBP3 | blue | PSMC5 | cyan | OLFML3 | darkorange | EPHA7 | grey | LOC400891 | grey | SDCCAG3 | grey |
| ATAD3C | blue | LOC101929078 | blue | ULK1 | blue | PSMC6 | cyan | OMD | darkorange | EPHA8 | grey | LOC400965 | grey | SDF2 | grey |
| ATF1 | blue | LOC101929125 | blue | UMOD | blue | PSMD1 | cyan | OSR2 | darkorange | EPHB2 | grey | LOC401068 | grey | SDHA | grey |
| ATF5 | blue | LOC101929132 | blue | UNC119 | blue | PSMD10 | cyan | OVOL1 | darkorange | EPHB6 | grey | LOC401320 | grey | SDHAF2 | grey |
| ATG16L1 | blue | LOC101929143 | blue | UNC13A | blue | PSMD14 | cyan | OVOL2 | darkorange | EPHX4 | grey | LOC414300 | grey | SDK1 | grey |
| ATG2A | blue | LOC101929144 | blue | UNC13D | blue | PSMD5 | cyan | PAPSS2 | darkorange | EPN2-AS1 | grey | LOC439911 | grey | SDR16C5 | grey |
| ATG3 | blue | LOC101929172 | blue | UNC5A | blue | PSMD6 | cyan | PARVA | darkorange | EPSTI1 | grey | LOC439938 | grey | SDR39U1 | grey |
| ATG4D | blue | LOC101929181 | blue | UNC5B | blue | PSMD8 | cyan | PCDH18 | darkorange | ERAL1 | grey | LOC439951 | grey | SEC14L5 | grey |
| ATHL1 | blue | LOC101929248 | blue | UNC5B-AS1 | blue | PSME1 | cyan | PCDHGA3 | darkorange | ERAP2 | grey | LOC440570 | grey | SEC22A | grey |
| ATIC | blue | LOC101929269 | blue | UNC79 | blue | PSME3 | cyan | PCOLCE | darkorange | ERC2-IT1 | grey | LOC440602 | grey | SEC22B | grey |
| ATM | blue | LOC101929384 | blue | UNC93B1 | blue | PSMF1 | cyan | PCOLCE2 | darkorange | ERI3 | grey | LOC440704 | grey | SEC24B-AS1 | grey |
| ATP11B | blue | LOC101929450 | blue | UPB1 | blue | PSMG1 | cyan | PCYT1A | darkorange | ERICH4 | grey | LOC441052 | grey | SEC31B | grey |
| ATP12A | blue | LOC101929526 | blue | UPF1 | blue | PSMG2 | cyan | PDE5A | darkorange | ERMAP | grey | LOC441081 | grey | SECTM1 | grey |
| ATP13A3 | blue | LOC101929552 | blue | UPK3A | blue | PSORS1C2 | cyan | PDGFD | darkorange | ERMARD | grey | LOC441086 | grey | SEL1L2 | grey |
| ATP1A1-AS1 | blue | LOC101929609 | blue | UPP1 | blue | PSORS1C3 | cyan | PDGFRL | darkorange | ERMN | grey | LOC441124 | grey | SELE | grey |
| ATP2B1 | blue | LOC101929655 | blue | UQCC2 | blue | PTBP3 | cyan | PDK4 | darkorange | ERP27 | grey | LOC441204 | grey | SELL | grey |
| ATP2B3 | blue | LOC101929705 | blue | UROC1 | blue | PTEN | cyan | PEA15 | darkorange | ERP29 | grey | LOC441242 | grey | SELM | grey |
| ATP2C1 | blue | LOC101929718 | blue | USB1 | blue | PTER | cyan | PEAK1 | darkorange | ERRFI1 | grey | LOC441454 | grey | SELO | grey |
| ATP2C2 | blue | LOC101929926 | blue | USHBP1 | blue | PTGES3 | cyan | PECAM1 | darkorange | ERV3-1 | grey | LOC441461 | grey | SELP | grey |
| ATP4A | blue | LOC101929949 | blue | USP19 | blue | PTGES3L | cyan | PHLDB2 | darkorange | ERV3-2 | grey | LOC541472 | grey | SELPLG | grey |
| ATP4B | blue | LOC101930026 | blue | USP2 | blue | PTGFRN | cyan | PI3 | darkorange | ERV9-1 | grey | LOC553103 | grey | SEMA3D | grey |
| ATP5F1 | blue | LOC101930421 | blue | USP22 | blue | PTK2 | cyan | PIM2 | darkorange | ERVFH21-1 | grey | LOC554174 | grey | SEMA3E | grey |
| ATP5J2 | blue | LOC102723526 | blue | USP27X-AS1 | blue | PTP4A1 | cyan | PITPNM2 | darkorange | ERVFRD-1 | grey | LOC57399 | grey | SEMA4A | grey |
| ATP6V0E2-AS1 | blue | LOC102723559 | blue | USP28 | blue | PTP4A2 | cyan | PLA2G5 | darkorange | ERVH-1 | grey | LOC613266 | grey | SEMA4B | grey |
| ATP6V1B1 | blue | LOC102723721 | blue | USP29 | blue | PTPLAD1 | cyan | PLAGL1 | darkorange | ERVH-3 | grey | LOC642533 | grey | SEMA4D | grey |
| ATP6V1B2 | blue | LOC102724021 | blue | USP4 | blue | PTPLAD2 | cyan | PLAT | darkorange | ERVH-4 | grey | LOC642620 | grey | SEMA5A | grey |
| ATP6V1H | blue | LOC102724094 | blue | USP42 | blue | PTPLB | cyan | PLBD1 | darkorange | ERVMER34-1 | grey | LOC642776 | grey | SEMA5B | grey |
| ATP8A2 | blue | LOC102724312 | blue | USP5 | blue | PTPN1 | cyan | PLCD3 | darkorange | ERVW-1 | grey | LOC642980 | grey | SENCR | grey |
| ATP8B3 | blue | LOC102724323 | blue | USP8 | blue | PTPN11 | cyan | PLCH1 | darkorange | ESPL1 | grey | LOC643072 | grey | SENP5 | grey |
| ATPAF1 | blue | LOC102724880 | blue | USPL1 | blue | PTPN12 | cyan | PLEK | darkorange | ETAA1 | grey | LOC643355 | grey | SENP7 | grey |
| ATXN2L | blue | LOC102724891 | blue | UTF1 | blue | PTPN18 | cyan | PLEKHH2 | darkorange | ETFB | grey | LOC643549 | grey | SEPHS2 | grey |
| ATXN7 | blue | LOC102724965 | blue | UTP18 | blue | PTPRK | cyan | PLS3 | darkorange | ETFDH | grey | LOC644090 | grey | SEPN1 | grey |
| ATXN7L3 | blue | LOC102724967 | blue | UTP23 | blue | PTRH2 | cyan | PLSCR4 | darkorange | ETHE1 | grey | LOC644656 | grey | SEPSECS-AS1 | grey |
| AURKAIP1 | blue | LOC102724975 | blue | UTS2R | blue | PTTG1IP | cyan | PLVAP | darkorange | EVA1A | grey | LOC644794 | grey | SEPT1 | grey |
| AURKB | blue | LOC102725022 | blue | VAC14 | blue | PUM1 | cyan | PLXDC2 | darkorange | EVA1C | grey | LOC644852 | grey | SEPT7P2 | grey |
| AVP | blue | LOC102725343 | blue | VAMP4 | blue | PUM2 | cyan | PMEPA1 | darkorange | EVC2 | grey | LOC645261 | grey | SEPT9 | grey |
| AVPR2 | blue | LOC102725345 | blue | VAPA | blue | PVRL4 | cyan | PMP22 | darkorange | EVI2A | grey | LOC645984 | grey | SERHL2 | grey |
| AX748157 | blue | LOC102725382 | blue | VARS | blue | PWAR6 | cyan | PNMA1 | darkorange | EXO1 | grey | LOC646241 | grey | SERPINA1 | grey |
| AX748267 | blue | LOC102725454 | blue | VAV2 | blue | PWP1 | cyan | POF1B | darkorange | EXOC2 | grey | LOC646268 | grey | SERPINA12 | grey |
| AY940074 | blue | LOC145694 | blue | VCX2 | blue | PWRN2 | cyan | PPAP2A | darkorange | EXOC3L2 | grey | LOC646482 | grey | SERPINA9 | grey |
| AZIN2 | blue | LOC146513 | blue | VEZT | blue | PXK | cyan | PPAP2B | darkorange | EXOSC5 | grey | LOC646484 | grey | SERPINB10 | grey |
| B3GALT4 | blue | LOC146795 | blue | VGF | blue | QSER1 | cyan | PPP1R12B | darkorange | EXOSC9 | grey | LOC646588 | grey | SERPINB11 | grey |
| B3GAT3 | blue | LOC146880 | blue | VILL | blue | R3HDM2 | cyan | PRELP | darkorange | EXT1 | grey | LOC646626 | grey | SERPINB7 | grey |
| B3GNT1 | blue | LOC147791 | blue | VMA21 | blue | RAB10 | cyan | PROS1 | darkorange | EYA2 | grey | LOC646736 | grey | SERPINB9 | grey |
| B3GNT2 | blue | LOC148413 | blue | VMO1 | blue | RAB11B | cyan | PRRT3-AS1 | darkorange | EYS | grey | LOC646903 | grey | SERPINB9P1 | grey |
| B3GNT4 | blue | LOC151174 | blue | VPS13A | blue | RAB11FIP2 | cyan | PRRX1 | darkorange | F11R | grey | LOC647323 | grey | SERPIND1 | grey |
| B3GNT6 | blue | LOC153546 | blue | VPS13A-AS1 | blue | RAB11FIP4 | cyan | PRSS23 | darkorange | F2R | grey | LOC652276 | grey | SERPINE1 | grey |
| B3GNT8 | blue | LOC153577 | blue | VPS13C | blue | RAB12 | cyan | PSD3 | darkorange | F2RL2 | grey | LOC652993 | grey | SERPINE2 | grey |
| B4GALNT2 | blue | LOC153684 | blue | VPS26A | blue | RAB14 | cyan | PTGIS | darkorange | F2RL3 | grey | LOC653160 | grey | SERPINI1 | grey |
| B4GALNT3 | blue | LOC157273 | blue | VPS36 | blue | RAB18 | cyan | PTPN14 | darkorange | FAAH2 | grey | LOC654841 | grey | SERTAD1 | grey |
| B4GALNT4 | blue | LOC157860 | blue | VPS37D | blue | RAB1A | cyan | PTPRB | darkorange | FAHD1 | grey | LOC727820 | grey | SERTAD3 | grey |
| B4GALT2 | blue | LOC157931 | blue | VPS9D1 | blue | RAB22A | cyan | PTPRG | darkorange | FAIM | grey | LOC728024 | grey | SERTAD4-AS1 | grey |
| B9D1 | blue | LOC200772 | blue | VPS9D1-AS1 | blue | RAB2A | cyan | PTPRM | darkorange | FAIM2 | grey | LOC728073 | grey | SESN1 | grey |
| B9D2 | blue | LOC220077 | blue | VRTN | blue | RAB2B | cyan | RAB23 | darkorange | FAM101B | grey | LOC728099 | grey | SESN2 | grey |
| BAALCOS | blue | LOC253805 | blue | VSIG2 | blue | RAB30 | cyan | RAB28 | darkorange | FAM106A | grey | LOC728392 | grey | SETD1B | grey |
| BAAT | blue | LOC254028 | blue | VSTM2L | blue | RAB3B | cyan | RAB31 | darkorange | FAM107A | grey | LOC728613 | grey | SETD7 | grey |
| BAD | blue | LOC255654 | blue | VSX1 | blue | RAB5A | cyan | RAMP2 | darkorange | FAM109B | grey | LOC728730 | grey | SETD9 | grey |
| BAG4 | blue | LOC283045 | blue | VTA1 | blue | RAB5B | cyan | RAP2B | darkorange | FAM110A | grey | LOC728805 | grey | SF3A2 | grey |
| BAG5 | blue | LOC283335 | blue | VWA1 | blue | RAB5C | cyan | RBFOX2 | darkorange | FAM110B | grey | LOC728819 | grey | SF3B5 | grey |
| BAHCC1 | blue | LOC283357 | blue | VWA5B1 | blue | RAB8A | cyan | RBMS1 | darkorange | FAM110C | grey | LOC728868 | grey | SFN | grey |
| BAHD1 | blue | LOC283674 | blue | VWA5B2 | blue | RAB8B | cyan | RBMS2 | darkorange | FAM118A | grey | LOC729173 | grey | SFR1 | grey |
| BAI1 | blue | LOC284600 | blue | VWA7 | blue | RAB9A | cyan | RBMS3 | darkorange | FAM120C | grey | LOC729224 | grey | SFRP1 | grey |
| BAIAP2 | blue | LOC284661 | blue | WBSCR16 | blue | RABEP1 | cyan | RBPMS | darkorange | FAM124B | grey | LOC729291 | grey | SFTPD | grey |
| BAIAP2L2 | blue | LOC284669 | blue | WBSCR17 | blue | RABGAP1 | cyan | RCAN2 | darkorange | FAM127B | grey | LOC729506 | grey | SFXN3 | grey |
| BAIAP3 | blue | LOC284798 | blue | WBSCR28 | blue | RABL3 | cyan | RFTN1 | darkorange | FAM129C | grey | LOC729658 | grey | SGCZ | grey |
| BAK1 | blue | LOC284889 | blue | WDR24 | blue | RAC1 | cyan | RGL1 | darkorange | FAM132B | grey | LOC729680 | grey | SGIP1 | grey |
| BANP | blue | LOC285095 | blue | WDR25 | blue | RAD17 | cyan | RIMKLB | darkorange | FAM134C | grey | LOC729866 | grey | SGK1 | grey |
| BARHL1 | blue | LOC285191 | blue | WDR26 | blue | RAD21 | cyan | RNASE1 | darkorange | FAM135B | grey | LOC729870 | grey | SGK2 | grey |
| BARX1 | blue | LOC285300 | blue | WDR27 | blue | RAI14 | cyan | ROBO1 | darkorange | FAM13A-AS1 | grey | LOC729970 | grey | SGK223 | grey |
| BARX1-AS1 | blue | LOC285847 | blue | WDR38 | blue | RAI2 | cyan | RP11-642D21.1 | darkorange | FAM13B | grey | LOC730101 | grey | SGPL1 | grey |
| BAZ1A | blue | LOC286083 | blue | WDR44 | blue | RALA | cyan | RP4-781L3.1 | darkorange | FAM13C | grey | LOC730102 | grey | SGSM2 | grey |
| BAZ2A | blue | LOC286114 | blue | WDR46 | blue | RALB | cyan | RPS6KA2 | darkorange | FAM149A | grey | LOC730202 | grey | SGTB | grey |
| BBIP1 | blue | LOC286121 | blue | WDR47 | blue | RANBP9 | cyan | RRAS | darkorange | FAM150B | grey | LOC730961 | grey | SH2B2 | grey |
| BBS9 | blue | LOC286359 | blue | WDR53 | blue | RAP1B | cyan | RSPO3 | darkorange | FAM154A | grey | LOC731424 | grey | SH2D2A | grey |
| BC017209 | blue | LOC338963 | blue | WDR55 | blue | RAPH1 | cyan | RUNX1T1 | darkorange | FAM154B | grey | LOC80154 | grey | SH2D4A | grey |
| BC022892 | blue | LOC339539 | blue | WDR5B | blue | RASA1 | cyan | S100A10 | darkorange | FAM155A | grey | LOC91548 | grey | SH2D4B | grey |
| BC023201 | blue | LOC339807 | blue | WDR62 | blue | RASSF2 | cyan | S100A4 | darkorange | FAM155B | grey | LOC93444 | grey | SH3BGR | grey |
| BC030152 | blue | LOC340357 | blue | WDR64 | blue | RASSF3 | cyan | SCARA5 | darkorange | FAM159A | grey | LOC93463 | grey | SH3BP5-AS1 | grey |
| BC043540 | blue | LOC374890 | blue | WDR66 | blue | RASSF7 | cyan | SCML1 | darkorange | FAM160A2 | grey | LOC93622 | grey | SH3GL1 | grey |
| BC045779 | blue | LOC388692 | blue | WDR7 | blue | RB1 | cyan | SDC2 | darkorange | FAM160B1 | grey | LONRF1 | grey | SH3PXD2A-AS1 | grey |
| BC069004 | blue | LOC388780 | blue | WDR74 | blue | RB1CC1 | cyan | SDF2L1 | darkorange | FAM162B | grey | LONRF2 | grey | SH3PXD2B | grey |
| BC079832 | blue | LOC388882 | blue | WDR83 | blue | RBBP4 | cyan | SDPR | darkorange | FAM166B | grey | LONRF3 | grey | SH3RF1 | grey |
| BC130595 | blue | LOC389199 | blue | WDR87 | blue | RBBP8 | cyan | SDR42E1 | darkorange | FAM169B | grey | LOR | grey | SH3RF2 | grey |
| BCAN | blue | LOC389332 | blue | WDR90 | blue | RBFOX1 | cyan | SEC23A | darkorange | FAM175A | grey | LOXL1-AS1 | grey | SH3TC1 | grey |
| BCAR1 | blue | LOC390705 | blue | WFDC3 | blue | RBL2 | cyan | SEMA3C | darkorange | FAM184B | grey | LOXL3 | grey | SHARPIN | grey |
| BCAR4 | blue | LOC399884 | blue | WFDC8 | blue | RBM12 | cyan | SEMA3G | darkorange | FAM186B | grey | LPA | grey | SHC1 | grey |
| BCAS1 | blue | LOC400548 | blue | WFIKKN1 | blue | RBM15B | cyan | SEPP1 | darkorange | FAM189A1 | grey | LPAR2 | grey | SHC3 | grey |
| BCAS4 | blue | LOC400568 | blue | WFIKKN2 | blue | RBM22 | cyan | SEPT11 | darkorange | FAM189A2 | grey | LPCAT1 | grey | SHISA2 | grey |
| BCAT2 | blue | LOC400768 | blue | WHAMMP2 | blue | RBM25 | cyan | SEPT8 | darkorange | FAM193A | grey | LPHN1 | grey | SHISA3 | grey |
| BCDIN3D-AS1 | blue | LOC400958 | blue | WNK4 | blue | RBM26 | cyan | SERPINF1 | darkorange | FAM193B | grey | LPHN3 | grey | SHISA6 | grey |
| BCL10 | blue | LOC401176 | blue | WNT1 | blue | RBM27 | cyan | SERPING1 | darkorange | FAM195B | grey | LPP-AS2 | grey | SHISA7 | grey |
| BCL2L10 | blue | LOC401463 | blue | WNT11 | blue | RBM3 | cyan | SERPINH1 | darkorange | FAM198A | grey | LPPR4 | grey | SHISA8 | grey |
| BCL2L11 | blue | LOC401913 | blue | WNT4 | blue | RBM39 | cyan | SFRP2 | darkorange | FAM198B | grey | LRCH2 | grey | SHMT2 | grey |
| BDKRB1 | blue | LOC402160 | blue | WNT7A | blue | RBM43 | cyan | SFRP4 | darkorange | FAM19A2 | grey | LRFN5 | grey | SHOX | grey |
| BECN1 | blue | LOC403323 | blue | WNT7B | blue | RBM5 | cyan | SGCB | darkorange | FAM19A5 | grey | LRGUK | grey | SHOX2 | grey |
| BEGAIN | blue | LOC440028 | blue | WNT8B | blue | RBM7 | cyan | SGCD | darkorange | FAM206A | grey | LRIF1 | grey | SHPK | grey |
| BEST1 | blue | LOC440149 | blue | WNT9A | blue | RBM8A | cyan | SH3D19 | darkorange | FAM20B | grey | LRIG1 | grey | SHPRH | grey |
| BEST4 | blue | LOC440173 | blue | WRAP53 | blue | RBMXL2 | cyan | SH3KBP1 | darkorange | FAM212A | grey | LRMP | grey | SHROOM2 | grey |
| BHLHE22 | blue | LOC440330 | blue | WSCD2 | blue | RBMY3AP | cyan | SH3PXD2A | darkorange | FAM219A | grey | LRP1B | grey | SIDT1 | grey |
| BHLHE23 | blue | LOC440792 | blue | WTAP | blue | RBPJ | cyan | SHANK3 | darkorange | FAM219B | grey | LRP2 | grey | SIDT2 | grey |
| BICD2 | blue | LOC441025 | blue | WTIP | blue | RCN2 | cyan | SHE | darkorange | FAM224A | grey | LRP2BP | grey | SIGIRR | grey |
| BIN3-IT1 | blue | LOC441601 | blue | WWC2-AS2 | blue | RCOR3 | cyan | SIGLEC1 | darkorange | FAM228A | grey | LRP4 | grey | SIGLEC10 | grey |
| BIRC5 | blue | LOC54944 | blue | WWTR1-AS1 | blue | RDX | cyan | SKP2 | darkorange | FAM228B | grey | LRP5 | grey | SIGLEC17P | grey |
| BIRC7 | blue | LOC554207 | blue | XAGE3 | blue | RECQL | cyan | SLC16A7 | darkorange | FAM229B | grey | LRRC1 | grey | SIGLEC5 | grey |
| BIVM | blue | LOC642236 | blue | XPOT | blue | REEP5 | cyan | SLC9B2 | darkorange | FAM230B | grey | LRRC10B | grey | SIGLEC9 | grey |
| BLK | blue | LOC644135 | blue | XPR1 | blue | REEP6 | cyan | SLCO2B1 | darkorange | FAM230C | grey | LRRC15 | grey | SIK1 | grey |
| BLOC1S3 | blue | LOC645321 | blue | XRCC1 | blue | RER1 | cyan | SLIT2 | darkorange | FAM26D | grey | LRRC2-AS1 | grey | SIK2 | grey |
| BMP7 | blue | LOC646014 | blue | XRCC3 | blue | RERE | cyan | SMAD3 | darkorange | FAM26E | grey | LRRC20 | grey | SIL1 | grey |
| BMP8A | blue | LOC646214 | blue | XRCC5 | blue | REV3L | cyan | SMIM22 | darkorange | FAM26F | grey | LRRC23 | grey | SIMC1 | grey |
| BMP8B | blue | LOC646522 | blue | XRN1 | blue | RGR | cyan | SNAI2 | darkorange | FAM32A | grey | LRRC25 | grey | SIN3B | grey |
| BNIPL | blue | LOC648987 | blue | XRRA1 | blue | RGS12 | cyan | SNED1 | darkorange | FAM3C | grey | LRRC37A2 | grey | SIRPA | grey |
| BOD1L1 | blue | LOC653581 | blue | XYLT1 | blue | RGS5 | cyan | SNX10 | darkorange | FAM43A | grey | LRRC37A4P | grey | SIRPB2 | grey |
| BOD1L2 | blue | LOC654780 | blue | YDJC | blue | RHAG | cyan | SPARC | darkorange | FAM43B | grey | LRRC37A5P | grey | SIRPD | grey |
| BOLA1 | blue | LOC692247 | blue | YES1 | blue | RHBDL2 | cyan | SPARCL1 | darkorange | FAM47C | grey | LRRC37BP1 | grey | SIRPG | grey |
| BOLA2 | blue | LOC728114 | blue | YIF1B | blue | RHOA | cyan | SPOCK1 | darkorange | FAM47E | grey | LRRC4 | grey | SIRT4 | grey |
| BRD7 | blue | LOC728353 | blue | YIPF6 | blue | RHOBTB3 | cyan | SPRY4 | darkorange | FAM49A | grey | LRRC46 | grey | SIX1 | grey |
| BRD7P3 | blue | LOC728445 | blue | YJEFN3 | blue | RHOQ | cyan | SPTAN1 | darkorange | FAM50A | grey | LRRC47 | grey | SIX2 | grey |
| BREA2 | blue | LOC728485 | blue | YLPM1 | blue | RHOU | cyan | SPTBN1 | darkorange | FAM53C | grey | LRRC49 | grey | SIX5 | grey |
| BRF1 | blue | LOC728743 | blue | YPEL2 | blue | RIIAD1 | cyan | SRGAP2 | darkorange | FAM57A | grey | LRRC4C | grey | SIX6 | grey |
| BRICD5 | blue | LOC729164 | blue | YTHDC1 | blue | RIMS1 | cyan | SRPX | darkorange | FAM58A | grey | LRRC52 | grey | SKA3 | grey |
| BRIX1 | blue | LOC729296 | blue | YTHDC2 | blue | RIN2 | cyan | STAB1 | darkorange | FAM65B | grey | LRRC61 | grey | SKAP1 | grey |
| BROX | blue | LOC729683 | blue | YTHDF3-AS1 | blue | RING1 | cyan | STEAP2 | darkorange | FAM69A | grey | LRRC75A | grey | SKAP2 | grey |
| BRSK1 | blue | LOC729887 | blue | ZAN | blue | RIPK1 | cyan | STOM | darkorange | FAM71D | grey | LRRC8D | grey | SKIDA1 | grey |
| BRSK2 | blue | LOC730098 | blue | ZAP70 | blue | RIPK4 | cyan | STON2 | darkorange | FAM71F2 | grey | LRRCC1 | grey | SKIL | grey |
| BRWD1 | blue | LOC79999 | blue | ZBTB12 | blue | RLIM | cyan | STS | darkorange | FAM81A | grey | LRRK1 | grey | SLA2 | grey |
| BRWD3 | blue | LOC91450 | blue | ZBTB17 | blue | RNASE4 | cyan | STXBP1 | darkorange | FAM83B | grey | LRRN1 | grey | SLAIN1 | grey |
| BSND | blue | LONP1 | blue | ZBTB2 | blue | RNASEH1 | cyan | SULF1 | darkorange | FAM83C-AS1 | grey | LRRN3 | grey | SLAMF1 | grey |
| BTBD16 | blue | LOXHD1 | blue | ZBTB20-AS1 | blue | RND3 | cyan | SVIL | darkorange | FAM86A | grey | LRRTM1 | grey | SLAMF6 | grey |
| BTBD17 | blue | LPAL2 | blue | ZBTB21 | blue | RNF10 | cyan | SYNE1 | darkorange | FAM86C1 | grey | LRRTM2 | grey | SLAMF8 | grey |
| BTF3L4 | blue | LPAR5 | blue | ZBTB22 | blue | RNF11 | cyan | SYNPO2 | darkorange | FAM89B | grey | LSM10 | grey | SLC10A1 | grey |
| BTNL2 | blue | LPAR6 | blue | ZBTB25 | blue | RNF111 | cyan | SYTL1 | darkorange | FAM90A1 | grey | LSM3 | grey | SLC10A3 | grey |
| BUD31 | blue | LPCAT2 | blue | ZBTB32 | blue | RNF114 | cyan | SYTL2 | darkorange | FAM95A | grey | LSM4 | grey | SLC12A1 | grey |
| C10orf2 | blue | LPCAT3 | blue | ZBTB34 | blue | RNF115 | cyan | TACC1 | darkorange | FAM96B | grey | LSM6 | grey | SLC12A5 | grey |
| C10orf35 | blue | LPGAT1 | blue | ZBTB45 | blue | RNF13 | cyan | TACSTD2 | darkorange | FAM98C | grey | LSM7 | grey | SLC12A7 | grey |
| C10orf55 | blue | LPIN3 | blue | ZBTB46-AS1 | blue | RNF138 | cyan | TBL3 | darkorange | FAM9C | grey | LSMEM1 | grey | SLC12A8 | grey |
| C10orf62 | blue | LPPR2 | blue | ZBTB48 | blue | RNF139 | cyan | TBX18 | darkorange | FANCD2OS | grey | LSP1 | grey | SLC15A3 | grey |
| C10orf71 | blue | LPPR3 | blue | ZBTB7B | blue | RNF146 | cyan | TCF4 | darkorange | FANCF | grey | LST1 | grey | SLC16A14 | grey |
| C10orf88 | blue | LQFBS-1 | blue | ZC3H14 | blue | RNF19A | cyan | TCF7L1 | darkorange | FANCG | grey | LTB | grey | SLC16A2 | grey |
| C10orf91 | blue | LRCH4 | blue | ZC3H15 | blue | RNF214 | cyan | TEAD1 | darkorange | FANK1 | grey | LUC7L2 | grey | SLC16A9 | grey |
| C11orf16 | blue | LRFN1 | blue | ZC3H4 | blue | RNF38 | cyan | TENC1 | darkorange | FAP | grey | LUZP2 | grey | SLC17A2 | grey |
| C11orf21 | blue | LRFN2 | blue | ZC3HAV1L | blue | RNF4 | cyan | TGFBI | darkorange | FASN | grey | LUZP4 | grey | SLC17A3 | grey |
| C11orf42 | blue | LRFN3 | blue | ZCCHC14 | blue | RNF43 | cyan | TGFBR2 | darkorange | FASTKD1 | grey | LY6E | grey | SLC17A4 | grey |
| C11orf49 | blue | LRFN4 | blue | ZCCHC3 | blue | RNPC3 | cyan | TGFBR3 | darkorange | FASTKD3 | grey | LY6G5C | grey | SLC17A7 | grey |
| C11orf53 | blue | LRIG3 | blue | ZCCHC7 | blue | ROCK2 | cyan | THBS2 | darkorange | FASTKD5 | grey | LY75 | grey | SLC18A1 | grey |
| C11orf54 | blue | LRIT1 | blue | ZCCHC9 | blue | ROS1 | cyan | THSD4 | darkorange | FAT1 | grey | LY86 | grey | SLC18A2 | grey |
| C11orf85 | blue | LRP3 | blue | ZDHHC1 | blue | RP1-170O19.14 | cyan | TIMP2 | darkorange | FAXC | grey | LYG1 | grey | SLC18B1 | grey |
| C11orf86 | blue | LRP6 | blue | ZDHHC11 | blue | RP11-1137G4.3 | cyan | TIMP3 | darkorange | FAXDC2 | grey | LYN | grey | SLC19A2 | grey |
| C11orf88 | blue | LRRC16B | blue | ZDHHC12 | blue | RP11-120K24.5 | cyan | TLN1 | darkorange | FBLN7 | grey | LYPD6B | grey | SLC1A5 | grey |
| C11orf97 | blue | LRRC27 | blue | ZDHHC18 | blue | RP11-138A9.1 | cyan | TLR4 | darkorange | FBXL12 | grey | LYPLA2 | grey | SLC20A2 | grey |
| C12orf29 | blue | LRRC29 | blue | ZDHHC19 | blue | RP11-1L12.3 | cyan | TMED3 | darkorange | FBXL19-AS1 | grey | LYRM1 | grey | SLC22A13 | grey |
| C12orf42 | blue | LRRC3 | blue | ZDHHC20 | blue | RP11-225H22.4 | cyan | TMEM176A | darkorange | FBXL2 | grey | LYRM4 | grey | SLC22A15 | grey |
| C12orf65 | blue | LRRC36 | blue | ZDHHC22 | blue | RP11-24P14.1 | cyan | TMEM200B | darkorange | FBXL22 | grey | LYSMD1 | grey | SLC22A17 | grey |
| C14orf178 | blue | LRRC37A3 | blue | ZDHHC24 | blue | RP11-285A1.1 | cyan | TMEM237 | darkorange | FBXO15 | grey | LYSMD2 | grey | SLC22A18 | grey |
| C14orf182 | blue | LRRC40 | blue | ZDHHC6 | blue | RP11-305K5.1 | cyan | TMEM255A | darkorange | FBXO17 | grey | LYSMD4 | grey | SLC22A2 | grey |
| C14orf23 | blue | LRRC43 | blue | ZDHHC8 | blue | RP11-350F4.2 | cyan | TMEM43 | darkorange | FBXO27 | grey | LYZL1 | grey | SLC22A24 | grey |
| C14orf79 | blue | LRRC45 | blue | ZDHHC8P1 | blue | RP11-38L15.2 | cyan | TMEM61 | darkorange | FBXO3 | grey | LYZL4 | grey | SLC22A3 | grey |
| C14orf80 | blue | LRRC48 | blue | ZFAND6 | blue | RP11-395B7.7 | cyan | TMEM64 | darkorange | FBXO43 | grey | LYZL6 | grey | SLC22A9 | grey |
| C15orf45 | blue | LRRC4B | blue | ZFC3H1 | blue | RP11-402G3.5 | cyan | TMOD1 | darkorange | FBXO46 | grey | LZTR1 | grey | SLC23A1 | grey |
| C15orf59 | blue | LRRC56 | blue | ZFHX2 | blue | RP11-410C4.5 | cyan | TMOD2 | darkorange | FBXO6 | grey | LZTS1-AS1 | grey | SLC23A2 | grey |
| C15orf62 | blue | LRRC57 | blue | ZFP3 | blue | RP11-44N11.3 | cyan | TMSB10 | darkorange | FBXW12 | grey | LZTS2 | grey | SLC23A3 | grey |
| C16orf13 | blue | LRRC58 | blue | ZFP62 | blue | RP11-456H18.2 | cyan | TMSB4X | darkorange | FBXW4 | grey | MAB21L2 | grey | SLC25A1 | grey |
| C16orf59 | blue | LRRC71 | blue | ZFP64 | blue | RP11-513N24.1 | cyan | TMTC1 | darkorange | FCAMR | grey | MAD1L1 | grey | SLC25A11 | grey |
| C16orf71 | blue | LRRC72 | blue | ZFPL1 | blue | RP11-559M23.1 | cyan | TNFRSF21 | darkorange | FCER1A | grey | MAD2L2 | grey | SLC25A14 | grey |
| C16orf74 | blue | LRRC73 | blue | ZFPM1 | blue | RP11-664D1.1 | cyan | TNRC6A | darkorange | FCER2 | grey | MADCAM1 | grey | SLC25A18 | grey |
| C16orf82 | blue | LRRC74 | blue | ZFX | blue | RP11-669C19.1 | cyan | TOB2 | darkorange | FCGBP | grey | MAEL | grey | SLC25A2 | grey |
| C16orf86 | blue | LRRIQ1 | blue | ZFYVE19 | blue | RP11-749H17.2 | cyan | TPP1 | darkorange | FCGR1B | grey | MAF1 | grey | SLC25A20 | grey |
| C16orf95 | blue | LRSAM1 | blue | ZFYVE27 | blue | RP11-753A21.1 | cyan | TRAF4 | darkorange | FCGR2A | grey | MAFF | grey | SLC25A23 | grey |
| C17orf50 | blue | LRTM2 | blue | ZFYVE28 | blue | RP11-803D5.4 | cyan | TRIO | darkorange | FCGR2C | grey | MAFK | grey | SLC25A25 | grey |
| C17orf53 | blue | LSAMP-AS1 | blue | ZG16 | blue | RP11-846E15.2 | cyan | TRIP10 | darkorange | FCGR3B | grey | MAGEA12 | grey | SLC25A27 | grey |
| C17orf59 | blue | LSM14B | blue | ZGLP1 | blue | RP3-337H4.8 | cyan | TRPC1 | darkorange | FCGRT | grey | MAGEA6 | grey | SLC25A29 | grey |
| C17orf67 | blue | LSM5 | blue | ZKSCAN2 | blue | RP3-368A4.6 | cyan | TSHZ3 | darkorange | FCN1 | grey | MAGEB4 | grey | SLC25A33 | grey |
| C17orf70 | blue | LSMEM2 | blue | ZKSCAN3 | blue | RP3-507I15.1 | cyan | TSKU | darkorange | FCRL1 | grey | MAGEC2 | grey | SLC25A37 | grey |
| C17orf74 | blue | LTA | blue | ZMYM1 | blue | RP3-507I15.2 | cyan | TSPAN18 | darkorange | FCRL2 | grey | MAGEC3 | grey | SLC25A38 | grey |
| C17orf82 | blue | LTB4R2 | blue | ZMYM5 | blue | RP3-508D13.1 | cyan | TSPAN7 | darkorange | FCRL3 | grey | MAGEE2 | grey | SLC25A39 | grey |
| C17orf89 | blue | LTBP3 | blue | ZMYND10 | blue | RP4-635E18.8 | cyan | TTC28 | darkorange | FCRL4 | grey | MAGEF1 | grey | SLC25A3P1 | grey |
| C17orf96 | blue | LTBR | blue | ZMYND11 | blue | RP5-1039K5.16 | cyan | TUBA1A | darkorange | FCRLB | grey | MAGEH1 | grey | SLC25A40 | grey |
| C18orf61 | blue | LTC4S | blue | ZMYND15 | blue | RPF2 | cyan | TUBB2A | darkorange | FDPS | grey | MAGEL2 | grey | SLC25A45 | grey |
| C19orf25 | blue | LTK | blue | ZMYND19 | blue | RPL22 | cyan | TUBB6 | darkorange | FDPSP2 | grey | MAGI1-IT1 | grey | SLC25A51 | grey |
| C19orf26 | blue | LTV1 | blue | ZNF114 | blue | RPL30 | cyan | TULP3 | darkorange | FDX1L | grey | MAGI2 | grey | SLC26A11 | grey |
| C19orf44 | blue | LURAP1 | blue | ZNF124 | blue | RPL31 | cyan | TYROBP | darkorange | FDXACB1 | grey | MAGI2-AS2 | grey | SLC26A6 | grey |
| C19orf45 | blue | LURAP1L | blue | ZNF14 | blue | RPL36AL | cyan | UACA | darkorange | FEM1A | grey | MAGI2-IT1 | grey | SLC26A8 | grey |
| C19orf47 | blue | LY6D | blue | ZNF143 | blue | RPL6 | cyan | UBE2Q2L | darkorange | FEN1 | grey | MAK | grey | SLC26A9 | grey |
| C19orf57 | blue | LY6G6E | blue | ZNF146 | blue | RPL7L1 | cyan | UCHL1 | darkorange | FER1L5 | grey | MAL | grey | SLC27A1 | grey |
| C19orf68 | blue | LY6H | blue | ZNF182 | blue | RPL9 | cyan | UPF3A | darkorange | FER1L6-AS1 | grey | MALRD1 | grey | SLC27A6 | grey |
| C19orf81 | blue | LY6K | blue | ZNF19 | blue | RPN2 | cyan | VAT1 | darkorange | FES | grey | MAMDC4 | grey | SLC29A3 | grey |
| C19orf83 | blue | LYG2 | blue | ZNF195 | blue | RPRD1A | cyan | VCAN | darkorange | FETUB | grey | MAMLD1 | grey | SLC29A4 | grey |
| C1QBP | blue | LYL1 | blue | ZNF205 | blue | RPRD2 | cyan | VCL | darkorange | FFAR2 | grey | MAN2B1 | grey | SLC2A1 | grey |
| C1QL1 | blue | LYPD1 | blue | ZNF212 | blue | RPS25 | cyan | VIM | darkorange | FGD1 | grey | MANBA | grey | SLC2A12 | grey |
| C1QTNF1-AS1 | blue | LYPD5 | blue | ZNF217 | blue | RPS6KB1 | cyan | VIT | darkorange | FGD2 | grey | MAOA | grey | SLC2A2 | grey |
| C1QTNF2 | blue | LYRM7 | blue | ZNF219 | blue | RPS6KC1 | cyan | VSIG4 | darkorange | FGD3 | grey | MAP10 | grey | SLC2A3 | grey |
| C1QTNF4 | blue | LYSMD3 | blue | ZNF23 | blue | RPS8 | cyan | VWF | darkorange | FGF1 | grey | MAP1LC3A | grey | SLC2A4 | grey |
| C1QTNF6 | blue | LZIC | blue | ZNF236 | blue | RPUSD4 | cyan | WLS | darkorange | FGF10-AS1 | grey | MAP1LC3C | grey | SLC2A4RG | grey |
| C1RL-AS1 | blue | LZTS1 | blue | ZNF252P | blue | RRAGA | cyan | XDH | darkorange | FGF13 | grey | MAP2 | grey | SLC2A9 | grey |
| C1orf111 | blue | MACROD1 | blue | ZNF253 | blue | RRAGC | cyan | ZAK | darkorange | FGF13-AS1 | grey | MAP2K1 | grey | SLC30A1 | grey |
| C1orf112 | blue | MAFG | blue | ZNF259P1 | blue | RRAS2 | cyan | ZDHHC7 | darkorange | FGF14-IT1 | grey | MAP2K2 | grey | SLC30A3 | grey |
| C1orf158 | blue | MAFG-AS1 | blue | ZNF273 | blue | RRP1B | cyan | ZFHX4 | darkorange | FGF16 | grey | MAP2K4 | grey | SLC35A5 | grey |
| C1orf159 | blue | MAG | blue | ZNF276 | blue | RSF1 | cyan | ZFP36L2 | darkorange | FGF18 | grey | MAP2K6 | grey | SLC35B4 | grey |
| C1orf174 | blue | MAGEA10 | blue | ZNF277 | blue | RSRC1 | cyan | ZNF117 | darkorange | FGF19 | grey | MAP3K14 | grey | SLC35F1 | grey |
| C1orf192 | blue | MAGEA11 | blue | ZNF282 | blue | RSRC2 | cyan | ZNF423 | darkorange | FGF20 | grey | MAP3K14-AS1 | grey | SLC35F6 | grey |
| C1orf200 | blue | MAGEB1 | blue | ZNF292 | blue | RTN2 | cyan | ZNF436 | darkorange | FGF23 | grey | MAP3K15 | grey | SLC35G1 | grey |
| C1orf204 | blue | MALSU1 | blue | ZNF302 | blue | RTN3 | cyan | ZNF438 | darkorange | FGF7 | grey | MAP3K3 | grey | SLC37A1 | grey |
| C1orf213 | blue | MALT1 | blue | ZNF304 | blue | RTN4 | cyan | ZNF521 | darkorange | FGF8 | grey | MAP3K5 | grey | SLC37A2 | grey |
| C1orf228 | blue | MAML3 | blue | ZNF316 | blue | RTP3 | cyan | ZNF532 | darkorange | FGFBP1 | grey | MAP3K8 | grey | SLC37A3 | grey |
| C1orf233 | blue | MAN1A2 | blue | ZNF324B | blue | RUFY2 | cyan | ZNF704 | darkorange | FGFBP2 | grey | MAP4K1 | grey | SLC37A4 | grey |
| C1orf35 | blue | MAN2A1 | blue | ZNF326 | blue | RUFY3 | cyan | ZNHIT6 | darkorange | FGFBP3 | grey | MAP4K2 | grey | SLC38A11 | grey |
| C1orf53 | blue | MAN2C1 | blue | ZNF335 | blue | RUSC1 | cyan | ALDOA | darkred | FGFR2 | grey | MAPK3 | grey | SLC39A1 | grey |
| C1orf64 | blue | MANEA | blue | ZNF341 | blue | RWDD2B | cyan | AP2S1 | darkred | FGFR3 | grey | MAPK8IP1 | grey | SLC39A10 | grey |
| C1orf68 | blue | MANEAL | blue | ZNF354C | blue | RWDD4 | cyan | ATP5E | darkred | FGR | grey | MARC1 | grey | SLC39A2 | grey |
| C1orf74 | blue | MAP1LC3B2 | blue | ZNF358 | blue | RYK | cyan | ATP5I | darkred | FHDC1 | grey | MARCKSL1 | grey | SLC39A4 | grey |
| C1orf86 | blue | MAP2K7 | blue | ZNF414 | blue | S100A11 | cyan | ATP5L | darkred | FHIT | grey | MARCO | grey | SLC43A1 | grey |
| C20orf141 | blue | MAP3K10 | blue | ZNF425 | blue | S100A16 | cyan | ATP6V0E1 | darkred | FHL2 | grey | MARK1 | grey | SLC43A3 | grey |
| C20orf144 | blue | MAP3K2 | blue | ZNF428 | blue | S100PBP | cyan | C19orf53 | darkred | FHL5 | grey | MARS2 | grey | SLC44A2 | grey |
| C20orf166 | blue | MAP3K6 | blue | ZNF429 | blue | SACM1L | cyan | CHCHD10 | darkred | FHOD1 | grey | MAS1L | grey | SLC45A4 | grey |
| C20orf181 | blue | MAP3K7 | blue | ZNF44 | blue | SALL3 | cyan | COX2 | darkred | FHOD3 | grey | MAT2A | grey | SLC47A1 | grey |
| C20orf26 | blue | MAP4K3 | blue | ZNF444 | blue | SAMD8 | cyan | COX5B | darkred | FIBP | grey | MATN3 | grey | SLC48A1 | grey |
| C21orf119 | blue | MAP4K5 | blue | ZNF446 | blue | SAMD9L | cyan | COX6B1 | darkred | FICD | grey | MATR3 | grey | SLC4A2 | grey |
| C21orf2 | blue | MAP6D1 | blue | ZNF467 | blue | SAMHD1 | cyan | COX7C | darkred | FIGN | grey | MB | grey | SLC4A3 | grey |
| C21orf58 | blue | MAPK11 | blue | ZNF469 | blue | SAP130 | cyan | DYNLT1 | darkred | FILIP1 | grey | MB21D2 | grey | SLC51A | grey |
| C21orf62 | blue | MAPK12 | blue | ZNF48 | blue | SAP18 | cyan | EDF1 | darkred | FIRRE | grey | MBIP | grey | SLC51B | grey |
| C21orf67 | blue | MAPK15 | blue | ZNF500 | blue | SAR1A | cyan | EEF1D | darkred | FIS1 | grey | MBLAC2 | grey | SLC52A1 | grey |
| C21orf90 | blue | MAPK4 | blue | ZNF503-AS2 | blue | SAR1B | cyan | EIF3G | darkred | FJX1 | grey | MBNL1-AS1 | grey | SLC52A2 | grey |
| C21orf91 | blue | MAPK8 | blue | ZNF506 | blue | SARAF | cyan | EPB41L4A-AS1 | darkred | FKBP1B | grey | MBOAT1 | grey | SLC5A10 | grey |
| C22orf31 | blue | MAPK8IP2 | blue | ZNF507 | blue | SASH1 | cyan | FAU | darkred | FKSG29 | grey | MBTPS1 | grey | SLC5A2 | grey |
| C22orf34 | blue | MAPK8IP3 | blue | ZNF517 | blue | SAV1 | cyan | GABARAP | darkred | FKSG49 | grey | MC5R | grey | SLC5A3 | grey |
| C2CD4B | blue | MAPRE3 | blue | ZNF524 | blue | SBF2 | cyan | HIGD2A | darkred | FLAD1 | grey | MCAT | grey | SLC6A12 | grey |
| C2CD4C | blue | MAPT | blue | ZNF529 | blue | SBSN | cyan | KRTCAP2 | darkred | FLG | grey | MCCC1 | grey | SLC6A16 | grey |
| C2orf16 | blue | MAPT-AS1 | blue | ZNF548 | blue | SC5D | cyan | LINC00493 | darkred | FLII | grey | MCEE | grey | SLC6A18 | grey |
| C2orf69 | blue | MARK2 | blue | ZNF550 | blue | SCAF11 | cyan | MIF | darkred | FLJ12825 | grey | MCEMP1 | grey | SLC6A9 | grey |
| C2orf72 | blue | MASP1 | blue | ZNF556 | blue | SCAMP1 | cyan | MRPL36 | darkred | FLJ16734 | grey | MCF2L-AS1 | grey | SLC7A11 | grey |
| C2orf82 | blue | MASP2 | blue | ZNF558 | blue | SCAMP5 | cyan | MRPL57 | darkred | FLJ20518 | grey | MCL1 | grey | SLC7A13 | grey |
| C2orf83 | blue | MAST1 | blue | ZNF562 | blue | SCARB2 | cyan | NDUFA1 | darkred | FLJ21369 | grey | MCM2 | grey | SLC7A7 | grey |
| C3P1 | blue | MAST2 | blue | ZNF571 | blue | SCFD1 | cyan | NDUFA3 | darkred | FLJ21408 | grey | MCM3 | grey | SLC7A8 | grey |
| C3orf18 | blue | MAST3 | blue | ZNF576 | blue | SCN8A | cyan | NDUFB10 | darkred | FLJ27354 | grey | MCM5 | grey | SLC7A9 | grey |
| C3orf20 | blue | MAT1A | blue | ZNF579 | blue | SCNN1G | cyan | NDUFB11 | darkred | FLJ30403 | grey | MCM6 | grey | SLC8A2 | grey |
| C3orf22 | blue | MAT2B | blue | ZNF585B | blue | SCP2 | cyan | OST4 | darkred | FLJ31715 | grey | MCOLN1 | grey | SLC8A3 | grey |
| C3orf27 | blue | MATK | blue | ZNF598 | blue | SCTR | cyan | PCBD1 | darkred | FLJ32255 | grey | MCOLN2 | grey | SLC9A3 | grey |
| C3orf38 | blue | MATN1 | blue | ZNF616 | blue | SDAD1 | cyan | PET100 | darkred | FLJ32955 | grey | MCU | grey | SLC9A3R2 | grey |
| C4BPB | blue | MATN4 | blue | ZNF619 | blue | SDC3 | cyan | PFDN5 | darkred | FLJ33360 | grey | MDH1 | grey | SLC9A9 | grey |
| C4orf29 | blue | MBD2 | blue | ZNF644 | blue | SDCBP | cyan | PPDPF | darkred | FLJ34503 | grey | MDH1B | grey | SLCO1B1 | grey |
| C5AR2 | blue | MBD3L2 | blue | ZNF646 | blue | SDK2 | cyan | RAD23A | darkred | FLJ35934 | grey | MDH2 | grey | SLCO1B3 | grey |
| C5orf28 | blue | MBD4 | blue | ZNF653 | blue | SDR9C7 | cyan | RBCK1 | darkred | FLJ36848 | grey | MDK | grey | SLCO2A1 | grey |
| C5orf42 | blue | MBD6 | blue | ZNF654 | blue | SDS | cyan | RNF181 | darkred | FLJ37201 | grey | MDM1 | grey | SLCO4C1 | grey |
| C5orf51 | blue | MBLAC1 | blue | ZNF664 | blue | SEC11A | cyan | RP4-595K12.1 | darkred | FLJ38717 | grey | MED10 | grey | SLCO6A1 | grey |
| C5orf66-AS1 | blue | MBNL3 | blue | ZNF668 | blue | SEC13 | cyan | RP5-882O7.1 | darkred | FLJ38773 | grey | MED11 | grey | SLFN12 | grey |
| C6orf15 | blue | MBTPS2 | blue | ZNF671 | blue | SEC14L1 | cyan | RPL10A | darkred | FLJ41170 | grey | MED15 | grey | SLFNL1-AS1 | grey |
| C6orf163 | blue | MC1R | blue | ZNF672 | blue | SEC24A | cyan | RPL11 | darkred | FLJ41455 | grey | MED15P9 | grey | SLIT2-IT1 | grey |
| C6orf164 | blue | MC3R | blue | ZNF683 | blue | SEC24B | cyan | RPL12 | darkred | FLJ42627 | grey | MED24 | grey | SLITRK1 | grey |
| C6orf165 | blue | MCHR1 | blue | ZNF688 | blue | SEC31A | cyan | RPL14 | darkred | FLJ45513 | grey | MED4 | grey | SLITRK2 | grey |
| C6orf211 | blue | MDFI | blue | ZNF696 | blue | SEC62 | cyan | RPL15 | darkred | FLJ45825 | grey | MEF2D | grey | SLITRK3 | grey |
| C6orf223 | blue | MDFIC | blue | ZNF71 | blue | SEC63 | cyan | RPL19 | darkred | FLJ46875 | grey | MEFV | grey | SLN | grey |
| C6orf226 | blue | MDGA1 | blue | ZNF710 | blue | SECISBP2 | cyan | RPL23A | darkred | FLJ90680 | grey | MEG3 | grey | SMA4 | grey |
| C6orf25 | blue | MECR | blue | ZNF74 | blue | SECISBP2L | cyan | RPL24 | darkred | FLNA | grey | MEG9 | grey | SMAD5-AS1 | grey |
| C6orf47 | blue | MED14OS | blue | ZNF740 | blue | SEH1L | cyan | RPL27 | darkred | FLNB | grey | MEGF6 | grey | SMAD7 | grey |
| C7orf13 | blue | MED16 | blue | ZNF746 | blue | SEL1L | cyan | RPL29 | darkred | FLOT2 | grey | MEGF9 | grey | SMAGP | grey |
| C7orf26 | blue | MED22 | blue | ZNF770 | blue | SELT | cyan | RPL3 | darkred | FLRT1 | grey | MELK | grey | SMARCAL1 | grey |
| C7orf43 | blue | MED23 | blue | ZNF771 | blue | SEMA4G | cyan | RPL32 | darkred | FLRT3 | grey | MEOX2 | grey | SMC1B | grey |
| C7orf50 | blue | MED27 | blue | ZNF775 | blue | SEPHS1 | cyan | RPL34 | darkred | FLT1 | grey | MEP1A | grey | SMC5-AS1 | grey |
| C7orf61 | blue | MED31 | blue | ZNF777 | blue | SEPT10 | cyan | RPL35 | darkred | FLT3 | grey | MEPCE | grey | SMC6 | grey |
| C8G | blue | MED6 | blue | ZNF780B | blue | SEPT12 | cyan | RPL36 | darkred | FLYWCH1 | grey | MERTK | grey | SMCP | grey |
| C8orf44 | blue | MED9 | blue | ZNF782 | blue | SEPT2 | cyan | RPL37 | darkred | FMN1 | grey | MESDC1 | grey | SMCR5 | grey |
| C8orf58 | blue | MEF2C-AS1 | blue | ZNF787 | blue | SEPT6 | cyan | RPL37A | darkred | FMNL3 | grey | MESP2 | grey | SMG8 | grey |
| C8orf66 | blue | MEGF11 | blue | ZNF789 | blue | SEPT7 | cyan | RPL38 | darkred | FMO1 | grey | METRNL | grey | SMIM10 | grey |
| C8orf74 | blue | MEGF8 | blue | ZNF793 | blue | SERBP1 | cyan | RPL39 | darkred | FMO3 | grey | METTL1 | grey | SMIM13 | grey |
| C8orf82 | blue | MEIS3 | blue | ZNF837 | blue | SERINC1 | cyan | RPL41 | darkred | FMOD | grey | METTL13 | grey | SMIM20 | grey |
| C9orf114 | blue | MEPE | blue | ZNF839 | blue | SERINC2 | cyan | RPL8 | darkred | FN3K | grey | METTL17 | grey | SMIM6 | grey |
| C9orf116 | blue | MESP1 | blue | ZNF853 | blue | SERINC3 | cyan | RPLP0 | darkred | FN3KRP | grey | METTL18 | grey | SMKR1 | grey |
| C9orf131 | blue | METRN | blue | ZNF91 | blue | SERINC5 | cyan | RPS10 | darkred | FNDC1 | grey | METTL21A | grey | SMO | grey |
| C9orf142 | blue | METTL6 | blue | ZNHIT2 | blue | SERPINB1 | cyan | RPS11 | darkred | FNDC4 | grey | METTL21EP | grey | SMOC2 | grey |
| C9orf172 | blue | MEX3A | blue | ZNRD1 | blue | SERPINB12 | cyan | RPS12 | darkred | FNDC7 | grey | METTL25 | grey | SMPD1 | grey |
| C9orf24 | blue | MEX3D | blue | ZNRF4 | blue | SERPINB5 | cyan | RPS17 | darkred | FNDC9 | grey | METTL2B | grey | SMPD2 | grey |
| C9orf37 | blue | MFAP3 | blue | ZP1 | blue | SERPINC1 | cyan | RPS18 | darkred | FOLH1 | grey | METTL7B | grey | SMPX | grey |
| C9orf43 | blue | MFI2-AS1 | blue | ZRANB2-AS1 | blue | SESN3 | cyan | RPS19 | darkred | FOLR2 | grey | MEX3B | grey | SMUG1 | grey |
| C9orf50 | blue | MFN1 | blue | ZSCAN10 | blue | SESTD1 | cyan | RPS21 | darkred | FOS | grey | MFAP1 | grey | SMURF1 | grey |
| C9orf53 | blue | MFSD10 | blue | ZSCAN2 | blue | SET | cyan | RPS27L | darkred | FOSB | grey | MFAP2 | grey | SMYD3 | grey |
| C9orf62 | blue | MFSD12 | blue | ZSCAN5A | blue | SETBP1 | cyan | RPS29 | darkred | FOSL1 | grey | MFGE8 | grey | SMYD4 | grey |
| C9orf89 | blue | MFSD2A | blue | ZSWIM3 | blue | SETD2 | cyan | RPS4X | darkred | FOSL2 | grey | MFNG | grey | SMYD5 | grey |
| CA5A | blue | MFSD6L | blue | ZSWIM4 | blue | SETX | cyan | RPS5 | darkred | FOXD1 | grey | MFSD3 | grey | SNAI3 | grey |
| CA7 | blue | MFSD7 | blue | ZSWIM8 | blue | SEZ6L | cyan | RPS7 | darkred | FOXF1 | grey | MFSD5 | grey | SNAP91 | grey |
| CA9 | blue | MGA | blue | ZW10 | blue | SF1 | cyan | SEPW1 | darkred | FOXI1 | grey | MGAM | grey | SNAPC1 | grey |
| CABP1 | blue | MGAT3 | blue | ZWILCH | blue | SF3A1 | cyan | SLC25A6 | darkred | FOXM1 | grey | MGAT1 | grey | SNAPIN | grey |
| CABP2 | blue | MGAT5B | blue | ZYG11A | blue | SF3A3 | cyan | TMEM256 | darkred | FOXN3-AS2 | grey | MGAT4B | grey | SND1 | grey |
| CABP4 | blue | MGC10814 | blue | A2M-AS1 | cyan | SF3B1 | cyan | UBA52 | darkred | FOXO6 | grey | MGC12488 | grey | SND1-IT1 | grey |
| CABP7 | blue | MGC34796 | blue | AAK1 | cyan | SF3B4 | cyan | UBB | darkred | FOXP1-IT1 | grey | MGC16275 | grey | SNF8 | grey |
| CACFD1 | blue | MGC4294 | blue | AARS2 | cyan | SFPQ | cyan | UQCRQ | darkred | FOXP4 | grey | MGC20647 | grey | SNHG17 | grey |
| CACNA1B | blue | MGC45922 | blue | AASDH | cyan | SFSWAP | cyan | UXT | darkred | FOXR2 | grey | MGC24103 | grey | SNHG18 | grey |
| CACNA1D | blue | MGC50722 | blue | AASDHPPT | cyan | SFT2D2 | cyan | WDR83OS | darkred | FOXRED1 | grey | MGC2889 | grey | SNHG19 | grey |
| CACNA1G | blue | MIA | blue | AASS | cyan | SFXN5 | cyan | A2MP1 | grey | FPR1 | grey | MGC32805 | grey | SNHG22 | grey |
| CACNA1H | blue | MIB2 | blue | ABCA17P | cyan | SGCE | cyan | AA06 | grey | FPR2 | grey | MGC34800 | grey | SNN | grey |
| CACNA1I | blue | MICAL1 | blue | ABCB4 | cyan | SGMS2 | cyan | AAAS | grey | FRAT1 | grey | MGC40069 | grey | SNORA21 | grey |
| CACNA1S | blue | MICALCL | blue | ABCB7 | cyan | SH2B3 | cyan | AACS | grey | FRAT2 | grey | MGC45800 | grey | SNORA37 | grey |
| CACNA2D2 | blue | MICALL2 | blue | ABCC11 | cyan | SH3BGRL | cyan | AADAC | grey | FREM1 | grey | MGC57346 | grey | SNORA65 | grey |
| CACNG1 | blue | MID2 | blue | ABCD3 | cyan | SH3BGRL3 | cyan | AADAT | grey | FRG1B | grey | MGC70870 | grey | SNORA68 | grey |
| CACNG2 | blue | MIER3 | blue | ABCE1 | cyan | SH3BP5 | cyan | AAMDC | grey | FRMD3 | grey | MGLL | grey | SNORA72 | grey |
| CACNG3 | blue | MIIP | blue | ABHD10 | cyan | SH3GLB1 | cyan | AAR2 | grey | FRMD4B | grey | MGME1 | grey | SNORA74A | grey |
| CACNG5 | blue | MIOX | blue | ABHD2 | cyan | SHISA5 | cyan | AARSD1 | grey | FRMD7 | grey | MGMT | grey | SNORD114-3 | grey |
| CACNG7 | blue | MIPEPP3 | blue | ABI1 | cyan | SHOC2 | cyan | AATK | grey | FRRS1 | grey | MGP | grey | SNRK-AS1 | grey |
| CACNG8 | blue | MIR1244-3 | blue | ABL1 | cyan | SIGMAR1 | cyan | ABCA11P | grey | FRRS1L | grey | MGST2 | grey | SNRNP200 | grey |
| CACTIN | blue | MIR142 | blue | ABLIM1 | cyan | SIKE1 | cyan | ABCA8 | grey | FRS3 | grey | MIA2 | grey | SNRNP25 | grey |
| CADM4 | blue | MIR146A | blue | AC010524.4 | cyan | SIRT3 | cyan | ABCB1 | grey | FRY | grey | MIATNB | grey | SNRNP70 | grey |
| CALCA | blue | MIR194-2 | blue | AC016999.2 | cyan | SKA2 | cyan | ABCB10 | grey | FRZB | grey | MICA | grey | SNRPA1 | grey |
| CALCR | blue | MIR210HG | blue | ACAP2 | cyan | SKI | cyan | ABCB11 | grey | FSCN1 | grey | MICAL2 | grey | SNRPB | grey |
| CALHM3 | blue | MIR4435-1HG | blue | ACBD3 | cyan | SKP1 | cyan | ABCC2 | grey | FSD2 | grey | MICB | grey | SNRPF | grey |
| CALY | blue | MIR7-3HG | blue | ACLY | cyan | SLAIN2 | cyan | ABCC4 | grey | FTH1P5 | grey | MICU3 | grey | SNRPN | grey |
| CAMK2A | blue | MIRLET7BHG | blue | ACOT9 | cyan | SLBP | cyan | ABCC6P1 | grey | FTSJ2 | grey | MID1 | grey | SNTG1 | grey |
| CAMK2B | blue | MIS18BP1 | blue | ACSL3 | cyan | SLC16A4 | cyan | ABCD1 | grey | FTSJ3 | grey | MIDN | grey | SNX12 | grey |
| CAMK2N2 | blue | MKRN7P | blue | ACSM2A | cyan | SLC17A6 | cyan | ABCD2 | grey | FUBP3 | grey | MIEF2 | grey | SNX15 | grey |
| CAMKK1 | blue | MLC1 | blue | ACSS1 | cyan | SLC17A9 | cyan | ABCF1 | grey | FUCA2 | grey | MIF4GD | grey | SNX16 | grey |
| CAMKV | blue | MLN | blue | ACTR10 | cyan | SLC1A6 | cyan | ABCG2 | grey | FUT4 | grey | MILR1 | grey | SNX17 | grey |
| CAMP | blue | MLNR | blue | ACTR2 | cyan | SLC22A11 | cyan | ABHD11 | grey | FXYD4 | grey | MINPP1 | grey | SNX20 | grey |
| CAPN11 | blue | MLST8 | blue | ACVR1 | cyan | SLC22A14 | cyan | ABHD12 | grey | FXYD6 | grey | MIR10A | grey | SNX27 | grey |
| CAPN5 | blue | MLXIP | blue | ACVR2A | cyan | SLC25A12 | cyan | ABHD14B | grey | FXYD7 | grey | MIR143HG | grey | SNX29 | grey |
| CAPN7 | blue | MLXIPL | blue | ADAM10 | cyan | SLC25A3 | cyan | ABHD15 | grey | FYB | grey | MIR205HG | grey | SNX30 | grey |
| CAPZB | blue | MLYCD | blue | ADAM17 | cyan | SLC25A36 | cyan | ABHD17A | grey | FYCO1 | grey | MIR31HG | grey | SNX31 | grey |
| CARD10 | blue | MMAB | blue | ADAM30 | cyan | SLC25A43 | cyan | ABHD3 | grey | FZD1 | grey | MIR4296 | grey | SNX33 | grey |
| CARD11 | blue | MMP11 | blue | ADAM6 | cyan | SLC25A46 | cyan | ABHD5 | grey | FZD10-AS1 | grey | MIR4313 | grey | SNX5 | grey |
| CARD14 | blue | MMP15 | blue | ADAM9 | cyan | SLC26A2 | cyan | ABHD8 | grey | FZD6 | grey | MIR4500HG | grey | SOCS2 | grey |
| CARS-AS1 | blue | MMP17 | blue | ADAR | cyan | SLC28A3 | cyan | ABI3 | grey | G6PC2 | grey | MIR4755 | grey | SOCS3 | grey |
| CASC14 | blue | MMP24 | blue | ADAT3 | cyan | SLC2A10 | cyan | ABO | grey | G6PD | grey | MIR622 | grey | SOD2 | grey |
| CASC18 | blue | MMP25 | blue | ADCY10 | cyan | SLC30A5 | cyan | ABRACL | grey | GAA | grey | MIR646HG | grey | SOGA1 | grey |
| CASC22 | blue | MNX1 | blue | ADCY9 | cyan | SLC30A7 | cyan | AC002059.10 | grey | GAB2 | grey | MIR670HG | grey | SOHLH2 | grey |
| CASC4 | blue | MOB2 | blue | ADD3 | cyan | SLC35A1 | cyan | AC003973.4 | grey | GAB3 | grey | MIRLET7D | grey | SORBS2 | grey |
| CASKIN1 | blue | MOB4 | blue | ADI1 | cyan | SLC35A4 | cyan | AC004692.5 | grey | GABARAPL1 | grey | MIS12 | grey | SORBS3 | grey |
| CASP16 | blue | MOGAT3 | blue | ADIPOR1 | cyan | SLC35F4 | cyan | AC005162.4 | grey | GABPB1-AS1 | grey | MISP | grey | SORT1 | grey |
| CASP2 | blue | MON1A | blue | ADNP | cyan | SLC38A2 | cyan | AC005224.2 | grey | GABRA3 | grey | MIXL1 | grey | SOST | grey |
| CASR | blue | MON2 | blue | ADO | cyan | SLC40A1 | cyan | AC005523.2 | grey | GABRA6 | grey | MKS1 | grey | SOWAHA | grey |
| CATSPERD | blue | MORN1 | blue | AES | cyan | SLC41A1 | cyan | AC005592.3 | grey | GABRP | grey | MLANA | grey | SOWAHC | grey |
| CATSPERG | blue | MORN3 | blue | AF289551 | cyan | SLC41A2 | cyan | AC006026.13 | grey | GAD1 | grey | MLH3 | grey | SOX13 | grey |
| CBLC | blue | MORN5 | blue | AF520793 | cyan | SLC44A1 | cyan | AC007292.3 | grey | GADD45A | grey | MLIP | grey | SOX17 | grey |
| CBX2 | blue | MOS | blue | AFF1 | cyan | SLC46A3 | cyan | AC007349.5 | grey | GADD45B | grey | MLLT11 | grey | SOX21-AS1 | grey |
| CBX8 | blue | MOSPD2 | blue | AGAP6 | cyan | SLC47A2 | cyan | AC007389.3 | grey | GADL1 | grey | MLLT4-AS1 | grey | SOX7 | grey |
| CCAR1 | blue | MPC2 | blue | AGFG1 | cyan | SLC4A1 | cyan | AC007401.2 | grey | GAFA2 | grey | MMACHC | grey | SOX9-AS1 | grey |
| CCBL1 | blue | MPHOSPH10 | blue | AGL | cyan | SLC4A1AP | cyan | AC007680.2 | grey | GAFA3 | grey | MME | grey | SP4 | grey |
| CCBL2 | blue | MPHOSPH6 | blue | AGO2 | cyan | SLC4A5 | cyan | AC008088.4 | grey | GAGE1 | grey | MMEL1 | grey | SP7 | grey |
| CCDC105 | blue | MPO | blue | AGO3 | cyan | SLC5A4 | cyan | AC008746.12 | grey | GAK | grey | MMP10 | grey | SPACA1 | grey |
| CCDC108 | blue | MPPED1 | blue | AGPS | cyan | SLC5A8 | cyan | AC009133.15 | grey | GAL3ST4 | grey | MMP12 | grey | SPACA6P | grey |
| CCDC114 | blue | MPV17L | blue | AHCYL1 | cyan | SLC6A20 | cyan | AC009502.4 | grey | GALNT11 | grey | MMP19 | grey | SPACA7 | grey |
| CCDC116 | blue | MRFAP1L1 | blue | AHCYL2 | cyan | SLC6A5 | cyan | AC010145.4 | grey | GALNT12 | grey | MMP24-AS1 | grey | SPAG1 | grey |
| CCDC129 | blue | MRGPRG-AS1 | blue | AHSA2 | cyan | SLC9A6 | cyan | AC012065.7 | grey | GALNT16 | grey | MMP26 | grey | SPAG17 | grey |
| CCDC13 | blue | MRM1 | blue | AHSG | cyan | SLFN11 | cyan | AC012531.25 | grey | GALNT18 | grey | MMP27 | grey | SPAG4 | grey |
| CCDC132 | blue | MRPL19 | blue | AIDA | cyan | SLFN5 | cyan | AC016831.7 | grey | GALNTL5 | grey | MMP3 | grey | SPAG5 | grey |
| CCDC135 | blue | MRPL28 | blue | AIM1 | cyan | SLIRP | cyan | AC017002.2 | grey | GALNTL6 | grey | MMP8 | grey | SPATA12 | grey |
| CCDC136 | blue | MRPL32 | blue | AIMP1 | cyan | SLK | cyan | AC018755.17 | grey | GALR1 | grey | MMP9 | grey | SPATA16 | grey |
| CCDC142 | blue | MRPL41 | blue | AJAP1 | cyan | SLMO2 | cyan | AC018816.3 | grey | GALR2 | grey | MMRN1 | grey | SPATA18 | grey |
| CCDC147 | blue | MRPL50 | blue | AK026905 | cyan | SLTM | cyan | AC024560.2 | grey | GAP43 | grey | MMS19 | grey | SPATA2 | grey |
| CCDC148-AS1 | blue | MRPL55 | blue | AK2 | cyan | SLU7 | cyan | AC064852.4 | grey | GAPDH | grey | MNDA | grey | SPATA2L | grey |
| CCDC150 | blue | MRPS12 | blue | AK3 | cyan | SMAD1 | cyan | AC067956.1 | grey | GAPT | grey | MNS1 | grey | SPATA32 | grey |
| CCDC151 | blue | MRPS18A | blue | AK6 | cyan | SMAD2 | cyan | AC068138.1 | grey | GAREML | grey | MNX1-AS1 | grey | SPATA41 | grey |
| CCDC153 | blue | MRPS26 | blue | AKAP13 | cyan | SMAD5 | cyan | AC073283.7 | grey | GAS2L1 | grey | MOAP1 | grey | SPATA45 | grey |
| CCDC154 | blue | MRPS30 | blue | AKIRIN1 | cyan | SMAD9 | cyan | AC079305.10 | grey | GAS5 | grey | MOB3B | grey | SPATA7 | grey |
| CCDC185 | blue | MRVI1-AS1 | blue | AKIRIN2 | cyan | SMAP1 | cyan | AC079767.4 | grey | GAS5-AS1 | grey | MOB3C | grey | SPATA8 | grey |
| CCDC19 | blue | MSANTD2 | blue | AKR1C6P | cyan | SMAP2 | cyan | AC079807.4 | grey | GAST | grey | MOCS3 | grey | SPATC1L | grey |
| CCDC22 | blue | MSANTD3 | blue | AKTIP | cyan | SMARCA1 | cyan | AC083843.1 | grey | GATA6 | grey | MOG | grey | SPATS1 | grey |
| CCDC24 | blue | MSC | blue | ALAS2 | cyan | SMARCA2 | cyan | AC083949.1 | grey | GATA6-AS1 | grey | MOGAT1 | grey | SPC25 | grey |
| CCDC27 | blue | MSH6 | blue | ALDH1A1 | cyan | SMARCA5 | cyan | AC090627.1 | grey | GATS | grey | MOGAT2 | grey | SPDYE2 | grey |
| CCDC28B | blue | MSL2 | blue | ALDH3A2 | cyan | SMARCAD1 | cyan | AC091133.1 | grey | GBF1 | grey | MOGS | grey | SPECC1 | grey |
| CCDC33 | blue | MST1 | blue | ALDH4A1 | cyan | SMARCC1 | cyan | AC092192.1 | grey | GBGT1 | grey | MOK | grey | SPECC1L | grey |
| CCDC37 | blue | MST1R | blue | ALDH6A1 | cyan | SMARCD1 | cyan | AC100830.4 | grey | GBP1P1 | grey | MORC2-AS1 | grey | SPESP1 | grey |
| CCDC40 | blue | MT3 | blue | ALDH9A1 | cyan | SMARCE1 | cyan | AC106801.1 | grey | GBP3 | grey | MORC4 | grey | SPG20 | grey |
| CCDC42B | blue | MT4 | blue | ALG13 | cyan | SMIM14 | cyan | AC108056.1 | grey | GCA | grey | MORF4L2-AS1 | grey | SPG20OS | grey |
| CCDC61 | blue | MTERF3 | blue | ALG8 | cyan | SMIM15 | cyan | AC112198.1 | grey | GCDH | grey | MORN2 | grey | SPHK1 | grey |
| CCDC63 | blue | MTF2 | blue | ALG9 | cyan | SMIM19 | cyan | AC114752.3 | grey | GCLM | grey | MORN4 | grey | SPHKAP | grey |
| CCDC64 | blue | MTFP1 | blue | ALOXE3 | cyan | SMIM2-AS1 | cyan | AC124997.1 | grey | GCM1 | grey | MOSPD3 | grey | SPI1 | grey |
| CCDC71 | blue | MTG1 | blue | AMD1 | cyan | SMPDL3B | cyan | AC139100.3 | grey | GCNT4 | grey | MOV10 | grey | SPIC | grey |
| CCDC78 | blue | MTG2 | blue | AMER3 | cyan | SMS | cyan | ACAD8 | grey | GCSAML | grey | MPG | grey | SPICE1 | grey |
| CCDC8 | blue | MTMR10 | blue | AMZ2 | cyan | SMTN | cyan | ACAD9 | grey | GCSAML-AS1 | grey | MPP1 | grey | SPIDR | grey |
| CCDC85B | blue | MTMR9LP | blue | ANAPC16 | cyan | SMURF2 | cyan | ACADL | grey | GDAP2 | grey | MPP2 | grey | SPIN4 | grey |
| CCDC88A | blue | MTNR1B | blue | ANAPC4 | cyan | SNAP23 | cyan | ACADVL | grey | GDF10 | grey | MPP3 | grey | SPINK1 | grey |
| CCDC88B | blue | MTRR | blue | ANAPC5 | cyan | SNORA71A | cyan | ACAP3 | grey | GDF3 | grey | MPPED2 | grey | SPINK13 | grey |
| CCDC9 | blue | MUC13 | blue | ANG | cyan | SNRNP27 | cyan | ACAT2 | grey | GDF5 | grey | MPZ | grey | SPINK2 | grey |
| CCIN | blue | MUC17 | blue | ANK1 | cyan | SNRNP48 | cyan | ACBD4 | grey | GDF9 | grey | MRAP2 | grey | SPINK4 | grey |
| CCKAR | blue | MUC3B | blue | ANKMY2 | cyan | SNRPA | cyan | ACBD5 | grey | GDI1 | grey | MREG | grey | SPINK5 | grey |
| CCL1 | blue | MUC4 | blue | ANKRD10-IT1 | cyan | SNRPD2 | cyan | ACBD6 | grey | GDPD1 | grey | MRGBP | grey | SPNS2 | grey |
| CCL17 | blue | MUC8 | blue | ANKRD12 | cyan | SNRPG | cyan | ACCS | grey | GDPD3 | grey | MRGPRX2 | grey | SPOCK2 | grey |
| CCL22 | blue | MURC | blue | ANKRD17 | cyan | SNX1 | cyan | ACD | grey | GDPGP1 | grey | MRGPRX3 | grey | SPON1 | grey |
| CCL24 | blue | MUT | blue | ANKRD27 | cyan | SNX18 | cyan | ACE | grey | GEMIN5 | grey | MRO | grey | SPP1 | grey |
| CCL26 | blue | MVB12B | blue | ANKRD28 | cyan | SNX19 | cyan | ACER1 | grey | GFI1 | grey | MROH2A | grey | SPP2 | grey |
| CCNE1 | blue | MVD | blue | ANKRD30B | cyan | SNX2 | cyan | ACIN1 | grey | GFRA1 | grey | MROH6 | grey | SPRED2 | grey |
| CCNF | blue | MVK | blue | ANKRD36B | cyan | SNX3 | cyan | ACKR4 | grey | GGCT | grey | MROH7 | grey | SPRR2B | grey |
| CCNJL | blue | MYADML2 | blue | ANKRD49 | cyan | SNX4 | cyan | ACMSD | grey | GGH | grey | MROH8 | grey | SPRR2G | grey |
| CCPG1 | blue | MYBL1 | blue | ANKRD6 | cyan | SNX6 | cyan | ACN9 | grey | GGT1 | grey | MRPL10 | grey | SPRR4 | grey |
| CCR10 | blue | MYBL2 | blue | ANKRD60 | cyan | SNX7 | cyan | ACO2 | grey | GGTA1P | grey | MRPL11 | grey | SPRY1 | grey |
| CCS | blue | MYBPC2 | blue | ANO6 | cyan | SNX9 | cyan | ACOT13 | grey | GGTLC1 | grey | MRPL13 | grey | SPRY3 | grey |
| CCSER2 | blue | MYBPC3 | blue | ANO8 | cyan | SOAT2 | cyan | ACOT4 | grey | GH1 | grey | MRPL14 | grey | SPRY4-IT1 | grey |
| CCT6A | blue | MYBPH | blue | ANP32A | cyan | SOD1 | cyan | ACOX2 | grey | GHDC | grey | MRPL15 | grey | SPRYD7 | grey |
| CCT8 | blue | MYCBPAP | blue | ANP32B | cyan | SON | cyan | ACP2 | grey | GIGYF2 | grey | MRPL16 | grey | SPSB1 | grey |
| CCT8L2 | blue | MYCN | blue | ANP32E | cyan | SP1 | cyan | ACP5 | grey | GIMAP1 | grey | MRPL2 | grey | SPTA1 | grey |
| CD276 | blue | MYEF2 | blue | ANXA11 | cyan | SP110 | cyan | ACP6 | grey | GIMAP2 | grey | MRPL21 | grey | SPX | grey |
| CD2BP2 | blue | MYEOV | blue | ANXA2P2 | cyan | SPAG16 | cyan | ACR | grey | GIMAP4 | grey | MRPL22 | grey | SQSTM1 | grey |
| CD300A | blue | MYF5 | blue | ANXA4 | cyan | SPAG9 | cyan | ACRC | grey | GIN1 | grey | MRPL23 | grey | SRBD1 | grey |
| CD300C | blue | MYH13 | blue | ANXA5 | cyan | SPATA20 | cyan | ACSBG1 | grey | GINS1 | grey | MRPL24 | grey | SRD5A3-AS1 | grey |
| CD300LB | blue | MYH3 | blue | ANXA7 | cyan | SPATA25 | cyan | ACSBG2 | grey | GIPC1 | grey | MRPL27 | grey | SRF | grey |
| CD320 | blue | MYH6 | blue | AP001063.1 | cyan | SPATA31C2 | cyan | ACSL1 | grey | GJA10 | grey | MRPL34 | grey | SRGN | grey |
| CD58 | blue | MYH7 | blue | AP1G1 | cyan | SPATA6 | cyan | ACSL4 | grey | GJA4 | grey | MRPL35 | grey | SRM | grey |
| CD7 | blue | MYH7B | blue | AP1S2 | cyan | SPCS1 | cyan | ACSM5 | grey | GJA5 | grey | MRPL37 | grey | SRP68 | grey |
| CD70 | blue | MYL4 | blue | AP2B1 | cyan | SPCS2 | cyan | ACSS3 | grey | GJB2 | grey | MRPL38 | grey | SRRD | grey |
| CD79B | blue | MYL7 | blue | AP3S1 | cyan | SPEN | cyan | ACTA1 | grey | GJB4 | grey | MRPL4 | grey | SRRM2-AS1 | grey |
| CD82 | blue | MYLK2 | blue | APC | cyan | SPG11 | cyan | ACTA2-AS1 | grey | GJC1 | grey | MRPL43 | grey | SRRM5 | grey |
| CD8B | blue | MYNN | blue | APEX1 | cyan | SPG21 | cyan | ACTB | grey | GK | grey | MRPL44 | grey | SRXN1 | grey |
| CDAN1 | blue | MYO15A | blue | APLP2 | cyan | SPG7 | cyan | ACTC1 | grey | GK2 | grey | MRPL45 | grey | SRY | grey |
| CDC23 | blue | MYO18B | blue | APOM | cyan | SPIB | cyan | ACTG2 | grey | GK3P | grey | MRPL46 | grey | SS18L1 | grey |
| CDC25A | blue | MYO1A | blue | APOOL | cyan | SPIN1 | cyan | ACTL6A | grey | GKN2 | grey | MRPL47 | grey | SS18L2 | grey |
| CDC34 | blue | MYO1E | blue | APP | cyan | SPINK6 | cyan | ACTN1 | grey | GLB1L | grey | MRPL48 | grey | SSBP3-AS1 | grey |
| CDC42SE1 | blue | MYO1G | blue | APPBP2 | cyan | SPIRE2 | cyan | ACTN3 | grey | GLCE | grey | MRPL52 | grey | SSH1 | grey |
| CDC45 | blue | MYO7B | blue | APPL1 | cyan | SPOP | cyan | ACTN4 | grey | GLDC | grey | MRPL54 | grey | SSRP1 | grey |
| CDC73 | blue | MYOD1 | blue | ARCN1 | cyan | SPRED1 | cyan | ACTR1A | grey | GLI2 | grey | MRPS14 | grey | SSSCA1 | grey |
| CDCA3 | blue | MYOG | blue | ARF3 | cyan | SPRNP1 | cyan | ACTR1B | grey | GLIDR | grey | MRPS17 | grey | SSSCA1-AS1 | grey |
| CDH15 | blue | MYOM2 | blue | ARF4 | cyan | SPRY2 | cyan | ACTR3C | grey | GLIPR2 | grey | MRPS2 | grey | SSTR5-AS1 | grey |
| CDH16 | blue | MYOM3 | blue | ARF6 | cyan | SPTLC1 | cyan | ACTRT3 | grey | GLIS1 | grey | MRPS28 | grey | SSX1 | grey |
| CDH22 | blue | MYOZ3 | blue | ARFGEF1 | cyan | SPTSSA | cyan | ACVR1C | grey | GLIS2 | grey | MRPS34 | grey | ST18 | grey |
| CDH26 | blue | MYRF | blue | ARFGEF2 | cyan | SPTY2D1 | cyan | ACVR2B-AS1 | grey | GLIS3 | grey | MRPS35 | grey | ST20 | grey |
| CDH4 | blue | MYSM1 | blue | ARFIP1 | cyan | SQRDL | cyan | ACYP1 | grey | GLMN | grey | MRPS7 | grey | ST3GAL4-AS1 | grey |
| CDH7 | blue | MYT1 | blue | ARHGAP12 | cyan | SRCIN1 | cyan | ADA | grey | GLRB | grey | MRPS9 | grey | ST3GAL5 | grey |
| CDHR2 | blue | MZT2B | blue | ARHGAP17 | cyan | SRGAP2C | cyan | ADAM12 | grey | GLRX | grey | MRRF | grey | ST5 | grey |
| CDHR5 | blue | N4BP1 | blue | ARHGAP21 | cyan | SRI | cyan | ADAM15 | grey | GLRX2 | grey | MRTO4 | grey | ST6GALNAC1 | grey |
| CDK10 | blue | NAA16 | blue | ARHGAP22 | cyan | SRP14 | cyan | ADAM32 | grey | GLT1D1 | grey | MRVI1 | grey | ST6GALNAC2 | grey |
| CDK12 | blue | NAA50 | blue | ARHGDIA | cyan | SRP19 | cyan | ADAM3A | grey | GLYAT | grey | MS4A12 | grey | ST6GALNAC3 | grey |
| CDK20 | blue | NAALADL1 | blue | ARHGDIB | cyan | SRP72 | cyan | ADAMDEC1 | grey | GLYATL2 | grey | MS4A14 | grey | ST7-AS1 | grey |
| CDK5R2 | blue | NAB2 | blue | ARID1A | cyan | SRP9 | cyan | ADAMTS1 | grey | GLYCTK-AS1 | grey | MS4A2 | grey | ST7-OT4 | grey |
| CDK5RAP1 | blue | NACAD | blue | ARID5B | cyan | SRPR | cyan | ADAMTS15 | grey | GM2A | grey | MS4A5 | grey | ST8SIA6-AS1 | grey |
| CDKL5 | blue | NACC1 | blue | ARIH1 | cyan | SRRM1 | cyan | ADAMTS18 | grey | GMFG | grey | MS4A8 | grey | STAC | grey |
| CDKN2A | blue | NADK2 | blue | ARL1 | cyan | SRRM2 | cyan | ADAMTS4 | grey | GMIP | grey | MSANTD1 | grey | STAC2 | grey |
| CDKN2AIP | blue | NAF1 | blue | ARL15 | cyan | SRSF1 | cyan | ADAMTS9 | grey | GML | grey | MSH2 | grey | STAG3L3 | grey |
| CDKN2B | blue | NAGLU | blue | ARL2BP | cyan | SRSF10 | cyan | ADAMTSL1 | grey | GNA14 | grey | MSI1 | grey | STAG3L4 | grey |
| CDKN2D | blue | NAGS | blue | ARL6IP1 | cyan | SRSF11 | cyan | ADAMTSL3 | grey | GNA14-AS1 | grey | MSMO1 | grey | STAM-AS1 | grey |
| CDRT15 | blue | NAIF1 | blue | ARL6IP5 | cyan | SRSF3 | cyan | ADARB2-AS1 | grey | GNAS-AS1 | grey | MSN | grey | STAMBPL1 | grey |
| CDRT15L2 | blue | NALCN-AS1 | blue | ARL8B | cyan | SRSF4 | cyan | ADAT1 | grey | GNAT2 | grey | MSR1 | grey | STAP1 | grey |
| CDX1 | blue | NANOS2 | blue | ARMC1 | cyan | SRSF5 | cyan | ADCY10P1 | grey | GNB2 | grey | MSRB1 | grey | STARD13-AS | grey |
| CDX2 | blue | NANOS3 | blue | ARMCX2 | cyan | SRSF6 | cyan | ADCY2 | grey | GNG13 | grey | MSS51 | grey | STARD3NL | grey |
| CDX4 | blue | NAPA-AS1 | blue | ARMCX3 | cyan | SRSF7 | cyan | ADCY6 | grey | GNG3 | grey | MSTO1 | grey | STARD4 | grey |
| CEACAM3 | blue | NAPRT | blue | ARMCX5 | cyan | SS18 | cyan | ADCY7 | grey | GNG4 | grey | MSX1 | grey | STARD5 | grey |
| CEBPA-AS1 | blue | NAPSA | blue | ARNT2 | cyan | SSB | cyan | ADCYAP1 | grey | GNL3 | grey | MSX2 | grey | STARD6 | grey |
| CEBPE | blue | NARFL | blue | ARPC1A | cyan | SSBP2 | cyan | ADCYAP1R1 | grey | GNLY | grey | MT1E | grey | STARD7-AS1 | grey |
| CEBPZOS | blue | NAT14 | blue | ARPC2 | cyan | SSBP3 | cyan | ADD3-AS1 | grey | GNPDA1 | grey | MT1F | grey | STARD8 | grey |
| CECR5-AS1 | blue | NAT16 | blue | ARPC3 | cyan | SSFA2 | cyan | ADH1A | grey | GNRH1 | grey | MT1G | grey | STARD9 | grey |
| CEL | blue | NAT6 | blue | ARPC5 | cyan | SSPN | cyan | ADHFE1 | grey | GOLGA1 | grey | MT1H | grey | STAT2 | grey |
| CELA1 | blue | NAT8L | blue | ARPP19 | cyan | SSR1 | cyan | ADIG | grey | GOLGA2P5 | grey | MT1HL1 | grey | STAT4 | grey |
| CELA2B | blue | NAT9 | blue | ARRDC3 | cyan | SSR3 | cyan | ADIRF | grey | GOLGA6A | grey | MT1M | grey | STAT5B | grey |
| CELA3A | blue | NAV2-IT1 | blue | ARRDC4 | cyan | SST | cyan | ADIRF-AS1 | grey | GOLM1 | grey | MT1X | grey | STAU2-AS1 | grey |
| CELF3 | blue | NBAS | blue | ARV1 | cyan | SSU72 | cyan | ADORA2A-AS1 | grey | GOLT1A | grey | MT2A | grey | STBD1 | grey |
| CELP | blue | NBPF8 | blue | ARX | cyan | ST13 | cyan | ADORA2B | grey | GOT1L1 | grey | MTA2 | grey | STC1 | grey |
| CELSR3 | blue | NBR1 | blue | ASAH1 | cyan | ST14 | cyan | ADORA3 | grey | GP1BA | grey | MTCP1 | grey | STGC3 | grey |
| CEMP1 | blue | NBR2 | blue | ASAP1 | cyan | ST6GALNAC4 | cyan | ADPRHL1 | grey | GP6 | grey | MTFR1 | grey | STIL | grey |
| CEND1 | blue | NCAPH2 | blue | ASCC1 | cyan | ST7-AS2 | cyan | ADPRHL2 | grey | GPA33 | grey | MTHFD1L | grey | STIP1 | grey |
| CENPA | blue | NCKAP1 | blue | ASCC2 | cyan | STAG1 | cyan | ADRA1A | grey | GPAA1 | grey | MTHFD2 | grey | STK10 | grey |
| CENPBD1P1 | blue | NCKAP5L | blue | ASCL1 | cyan | STAG2 | cyan | ADRB1 | grey | GPATCH1 | grey | MTIF2 | grey | STK17A | grey |
| CENPM | blue | NCKIPSD | blue | ASCL2 | cyan | STAM2 | cyan | ADRB2 | grey | GPATCH11 | grey | MTMR11 | grey | STK32A | grey |
| CENPT | blue | NCLN | blue | ASH1L | cyan | STAT3 | cyan | ADRBK2 | grey | GPHA2 | grey | MTMR12 | grey | STK32B | grey |
| CEP131 | blue | NCOA3 | blue | ASPH | cyan | STAT6 | cyan | AEBP2 | grey | GPR101 | grey | MTMR14 | grey | STK36 | grey |
| CEP250 | blue | NCOA6 | blue | ASPRV1 | cyan | STAU1 | cyan | AF001548.5 | grey | GPR112 | grey | MTMR4 | grey | STK4-AS1 | grey |
| CEP83-AS1 | blue | NCOR1 | blue | ATAD1 | cyan | STEAP4 | cyan | AF007147 | grey | GPR125 | grey | MTMR6 | grey | STMN2 | grey |
| CEP97 | blue | NCR1 | blue | ATAD2B | cyan | STIM1 | cyan | AF070581 | grey | GPR128 | grey | MTNR1A | grey | STOML2 | grey |
| CERS4 | blue | NCR2 | blue | ATF2 | cyan | STIM2 | cyan | AF131215.4 | grey | GPR143 | grey | MTOR | grey | STOML3 | grey |
| CES1P1 | blue | NCR3 | blue | ATF6 | cyan | STRAP | cyan | AF131215.8 | grey | GPR146 | grey | MTPN | grey | STOX1 | grey |
| CES4A | blue | NCS1 | blue | ATF7 | cyan | STT3B | cyan | AF198444 | grey | GPR15 | grey | MTR | grey | STPG2 | grey |
| CFB | blue | NDC80 | blue | ATG12 | cyan | STX12 | cyan | AFAP1 | grey | GPR158 | grey | MTTP | grey | STRA13 | grey |
| CGREF1 | blue | NDOR1 | blue | ATG14 | cyan | STX16 | cyan | AFAP1L2 | grey | GPR171 | grey | MTURN | grey | STRADA | grey |
| CHADL | blue | NDUFA4L2 | blue | ATG5 | cyan | STX17 | cyan | AFF3 | grey | GPR174 | grey | MTX1 | grey | STRADB | grey |
| CHAF1B | blue | NDUFA5 | blue | ATG9B | cyan | STXBP5-AS1 | cyan | AFP | grey | GPR18 | grey | MUC1 | grey | STRIP1 | grey |
| CHCHD5 | blue | NDUFB2-AS1 | blue | ATL3 | cyan | SUB1 | cyan | AGAP11 | grey | GPR183 | grey | MUC12 | grey | STRN3 | grey |
| CHCHD6 | blue | NDUFS7 | blue | ATMIN | cyan | SUCLA2 | cyan | AGAP2-AS1 | grey | GPR19 | grey | MUC20 | grey | STRN4 | grey |
| CHD1 | blue | NEBL-AS1 | blue | ATP10D | cyan | SUCLG2 | cyan | AGAP4 | grey | GPR21 | grey | MUC5AC | grey | STT3A | grey |
| CHD2 | blue | NECAB2 | blue | ATP11C | cyan | SUCO | cyan | AGAP9 | grey | GPR35 | grey | MUC6 | grey | STX10 | grey |
| CHD5 | blue | NECAB3 | blue | ATP13A2 | cyan | SUDS3 | cyan | AGBL2 | grey | GPR4 | grey | MUC7 | grey | STX11 | grey |
| CHGA | blue | NECAP1 | blue | ATP13A4 | cyan | SUGT1 | cyan | AGPAT4 | grey | GPR50 | grey | MUL1 | grey | STX18 | grey |
| CHIA | blue | NEDD1 | blue | ATP2A1 | cyan | SULF2 | cyan | AGPAT9 | grey | GPR63 | grey | MUM1 | grey | STX19 | grey |
| CHIC1 | blue | NEFH | blue | ATP5C1 | cyan | SUMF2 | cyan | AGT | grey | GPR64 | grey | MUM1L1 | grey | STX1B | grey |
| CHID1 | blue | NEK1 | blue | ATP5J | cyan | SUMO1 | cyan | AGTR2 | grey | GPR65 | grey | MUS81 | grey | STX3 | grey |
| CHIT1 | blue | NEK10 | blue | ATP5O | cyan | SUMO3 | cyan | AGXT2 | grey | GPR75 | grey | MUTYH | grey | STX4 | grey |
| CHKB-AS1 | blue | NENF | blue | ATP6AP2 | cyan | SUMO4 | cyan | AHCY | grey | GPR83 | grey | MVB12A | grey | STX5 | grey |
| CHM | blue | NEU2 | blue | ATP6V0C | cyan | SUN1 | cyan | AHR | grey | GPR84 | grey | MVP | grey | STX8 | grey |
| CHMP1A | blue | NEU4 | blue | ATP6V0D1 | cyan | SUPT16H | cyan | AHRR | grey | GPR87 | grey | MX1 | grey | STXBP2 | grey |
| CHMP6 | blue | NEURL1 | blue | ATP6V1A | cyan | SUPT20H | cyan | AIF1 | grey | GPR88 | grey | MX2 | grey | STXBP3 | grey |
| CHORDC1 | blue | NEURL4 | blue | ATP6V1D | cyan | SUPT4H1 | cyan | AIF1L | grey | GPRC5A | grey | MXRA5 | grey | STYXL1 | grey |
| CHP2 | blue | NEUROD2 | blue | ATP6V1E1 | cyan | SUPT7L | cyan | AIG1 | grey | GPRC5B | grey | MYADM | grey | SUGCT | grey |
| CHRD | blue | NEUROG1 | blue | ATP6V1G1 | cyan | SUV420H1 | cyan | AIP | grey | GPRC5D | grey | MYB | grey | SUGP2 | grey |
| CHRDL2 | blue | NEUROG2 | blue | ATRAID | cyan | SV2B | cyan | AK000798 | grey | GPSM3 | grey | MYBPC1 | grey | SUGT1P1 | grey |
| CHRM4 | blue | NEUROG3 | blue | ATRX | cyan | SVOP | cyan | AK021537 | grey | GPT2 | grey | MYC | grey | SULT1A1 | grey |
| CHRNA10 | blue | NFAM1 | blue | ATXN1 | cyan | SWAP70 | cyan | AK021933 | grey | GPX2 | grey | MYCT1 | grey | SULT1B1 | grey |
| CHRNA2 | blue | NFASC | blue | ATXN10 | cyan | SYCP1 | cyan | AK021977 | grey | GPX7 | grey | MYEOV2 | grey | SULT1C4 | grey |
| CHRNA4 | blue | NFE2 | blue | ATXN1L | cyan | SYNCRIP | cyan | AK024936 | grey | GRAMD1B | grey | MYF6 | grey | SUMF1 | grey |
| CHRNA6 | blue | NFKBIB | blue | ATXN2 | cyan | SYNE2 | cyan | AK025288 | grey | GRAMD2 | grey | MYH1 | grey | SUOX | grey |
| CHRNB2 | blue | NFKBIL1 | blue | ATXN7L3B | cyan | SYPL1 | cyan | AK054988 | grey | GRAMD3 | grey | MYH11 | grey | SUPT6H | grey |
| CHRND | blue | NGB | blue | AUH | cyan | SZRD1 | cyan | AK055981 | grey | GRB10 | grey | MYH15 | grey | SUPV3L1 | grey |
| CHRNE | blue | NGF | blue | AUP1 | cyan | TAB2 | cyan | AK056982 | grey | GRB14 | grey | MYH16 | grey | SURF1 | grey |
| CHRNG | blue | NHLRC4 | blue | AVIL | cyan | TAB3 | cyan | AK090844 | grey | GRIA1 | grey | MYH2 | grey | SUSD1 | grey |
| CHST13 | blue | NIFK | blue | AVPR1B | cyan | TAF11 | cyan | AK091729 | grey | GRID1-AS1 | grey | MYH4 | grey | SUSD2 | grey |
| CHST5 | blue | NIFK-AS1 | blue | AWAT1 | cyan | TAF2 | cyan | AK131021 | grey | GRID2 | grey | MYL1 | grey | SUSD5 | grey |
| CHST6 | blue | NIM1K | blue | AX747031 | cyan | TAF6L | cyan | AK8 | grey | GRIK1-AS2 | grey | MYL3 | grey | SUV39H1 | grey |
| CHST8 | blue | NIP7 | blue | AX747191 | cyan | TAGLN2 | cyan | AKAP1 | grey | GRIN2D | grey | MYL6 | grey | SV2A | grey |
| CHTF18 | blue | NIPBL | blue | AY927499 | cyan | TAOK1 | cyan | AKAP11 | grey | GRINA | grey | MYL6B | grey | SWI5 | grey |
| CIAO1 | blue | NKAIN1 | blue | AZIN1 | cyan | TAPBP | cyan | AKAP17A | grey | GRM3 | grey | MYL9 | grey | SWT1 | grey |
| CIB2 | blue | NKAIN4 | blue | AZU1 | cyan | TARDBP | cyan | AKNA | grey | GRN | grey | MYLK | grey | SYAP1 | grey |
| CIB3 | blue | NKAPP1 | blue | B2M | cyan | TARS | cyan | AKR1B10 | grey | GRP | grey | MYO15B | grey | SYBU | grey |
| CIC | blue | NKD2 | blue | B3GAT2 | cyan | TAS1R1 | cyan | AKR1D1 | grey | GRPEL1 | grey | MYO16 | grey | SYCE1L | grey |
| CIRBP-AS1 | blue | NKPD1 | blue | B3GNT3 | cyan | TATDN1 | cyan | AKR7A2 | grey | GRPR | grey | MYO1B | grey | SYCE2 | grey |
| CITED1 | blue | NKX1-1 | blue | B3GNTL1 | cyan | TAX1BP1 | cyan | AKT1 | grey | GS1-111G14.1 | grey | MYO1D | grey | SYCE3 | grey |
| CITED4 | blue | NKX2-1 | blue | BACE2 | cyan | TBC1D20 | cyan | AKT1S1 | grey | GS1-124K5.11 | grey | MYO1F | grey | SYCN | grey |
| CLASP2 | blue | NKX2-1-AS1 | blue | BANF1 | cyan | TBC1D23 | cyan | AL109706 | grey | GS1-24F4.2 | grey | MYO9B | grey | SYCP2L | grey |
| CLCF1 | blue | NKX2-3 | blue | BAZ2B | cyan | TBC1D28 | cyan | AL132709.8 | grey | GS1-259H13.2 | grey | MYOC | grey | SYCP3 | grey |
| CLCN1 | blue | NKX2-5 | blue | BBX | cyan | TBC1D5 | cyan | AL832163 | grey | GS1-279B7.1 | grey | MYOM1 | grey | SYDE2 | grey |
| CLCN2 | blue | NKX2-8 | blue | BC022047 | cyan | TBCA | cyan | AL833181 | grey | GS1-600G8.3 | grey | MYOT | grey | SYN1 | grey |
| CLCN7 | blue | NKX6-1 | blue | BC045788 | cyan | TBCK | cyan | AL928742.12 | grey | GSKIP | grey | MYOZ1 | grey | SYNDIG1 | grey |
| CLCNKB | blue | NKX6-2 | blue | BCAS2 | cyan | TBL1XR1 | cyan | ALAD | grey | GSS | grey | MYPOP | grey | SYNGR3 | grey |
| CLDN14 | blue | NKX6-3 | blue | BCL6 | cyan | TCEAL1 | cyan | ALAS1 | grey | GSTA1 | grey | MYRFL | grey | SYNGR4 | grey |
| CLDN15 | blue | NLE1 | blue | BCL6B | cyan | TCEAL3 | cyan | ALDH1A2 | grey | GSTA4 | grey | MYRIP | grey | SYNM | grey |
| CLDN19 | blue | NLGN2 | blue | BCLAF1 | cyan | TCEAL4 | cyan | ALDH1B1 | grey | GSTM1 | grey | MYZAP | grey | SYPL2 | grey |
| CLDN6 | blue | NLGN3 | blue | BCRP3 | cyan | TCEAL8 | cyan | ALDH5A1 | grey | GSTM2 | grey | MZT1 | grey | SYT1 | grey |
| CLDN9 | blue | NLRC4 | blue | BEND4 | cyan | TCEB1 | cyan | ALDOAP2 | grey | GSTP1 | grey | N4BP2 | grey | SYT15 | grey |
| CLDND2 | blue | NLRP12 | blue | BET1 | cyan | TCF7L2 | cyan | ALG12 | grey | GSTT1 | grey | N4BP3 | grey | SYT17 | grey |
| CLEC10A | blue | NLRP4 | blue | BEX4 | cyan | TCFL5 | cyan | ALG3 | grey | GSTT2 | grey | N6AMT2 | grey | SYT3 | grey |
| CLEC11A | blue | NLRP5 | blue | BFSP2-AS1 | cyan | TCP11L1 | cyan | ALG5 | grey | GSTTP1 | grey | NAA11 | grey | SYT4 | grey |
| CLEC16A | blue | NLRP6 | blue | BIRC6 | cyan | TCTA | cyan | ALKBH2 | grey | GSTZ1 | grey | NAA35 | grey | SYTL5 | grey |
| CLEC1A | blue | NLRP7 | blue | BLOC1S2 | cyan | TCTE3 | cyan | ALOX12 | grey | GTF2F2 | grey | NAA38 | grey | T-18 | grey |
| CLEC1B | blue | NLRP8 | blue | BLOC1S6 | cyan | TCTN3 | cyan | ALOX12P2 | grey | GTF2H2B | grey | NAA60 | grey | TAAR3 | grey |
| CLINT1 | blue | NLRX1 | blue | BMP2K | cyan | TDH | cyan | ALOX15 | grey | GTF2H3 | grey | NAALADL2 | grey | TAAR5 | grey |
| CLIP1-AS1 | blue | NMD3 | blue | BMPR1A | cyan | TDP2 | cyan | ALOX5 | grey | GTF2IRD1 | grey | NAALADL2-AS3 | grey | TAAR8 | grey |
| CLN5 | blue | NME3 | blue | BMPR2 | cyan | TDRD3 | cyan | ALOX5AP | grey | GTF2IRD2B | grey | NABP1 | grey | TAAR9 | grey |
| CLN6 | blue | NMNAT3 | blue | BMS1P5 | cyan | TERF1 | cyan | ALPK2 | grey | GTSF1 | grey | NABP2 | grey | TAB1 | grey |
| CLP1 | blue | NMRK2 | blue | BMS1P6 | cyan | TERF2IP | cyan | ALPK3 | grey | GTSF1L | grey | NACA2 | grey | TAC3 | grey |
| CLPS | blue | NMU | blue | BNIP2 | cyan | TERT | cyan | ALS2CR12 | grey | GUCD1 | grey | NACAP1 | grey | TACR1 | grey |
| CLPX | blue | NMUR1 | blue | BNIP3 | cyan | TES | cyan | ALX4 | grey | GUCY1B2 | grey | NACC2 | grey | TACR3 | grey |
| CLSTN3 | blue | NOA1 | blue | BNIP3L | cyan | TESC | cyan | ALYREF | grey | GUF1 | grey | NADK2-AS1 | grey | TADA3 | grey |
| CLTC | blue | NOBOX | blue | BPGM | cyan | TEX13B | cyan | AMBN | grey | GUK1 | grey | NAGA | grey | TAF12 | grey |
| CLUH | blue | NOC4L | blue | BPIFB1 | cyan | TFAP2A-AS1 | cyan | AMDHD1 | grey | GUSBP11 | grey | NAGPA | grey | TAF13 | grey |
| CMTM5 | blue | NOMO3 | blue | BPIFB4 | cyan | TFDP2 | cyan | AMELX | grey | GVINP1 | grey | NAIP | grey | TAF1A-AS1 | grey |
| CNBD2 | blue | NOP16 | blue | BPTF | cyan | TFEB | cyan | AMFR | grey | GXYLT1 | grey | NALCN | grey | TAF1C | grey |
| CNFN | blue | NOP9 | blue | BRAF | cyan | TFF2 | cyan | AMMECR1L | grey | GYS1 | grey | NAMPT | grey | TAF1D | grey |
| CNGA4 | blue | NOS2 | blue | BRD1 | cyan | TGFB2 | cyan | AMN1 | grey | GZMA | grey | NANOG | grey | TAF1L | grey |
| CNGB1 | blue | NOS3 | blue | BRD2 | cyan | TGFBR1 | cyan | AMOT | grey | GZMB | grey | NANP | grey | TAF6 | grey |
| CNIH1 | blue | NOTUM | blue | BRDT | cyan | TGOLN2 | cyan | AMPD2 | grey | GZMH | grey | NAP1L2 | grey | TAGLN | grey |
| CNIH2 | blue | NOX5 | blue | BTAF1 | cyan | THAP5 | cyan | AMPD3 | grey | GZMK | grey | NAPB | grey | TALDO1 | grey |
| CNNM4 | blue | NOXA1 | blue | BTBD1 | cyan | THAP7-AS1 | cyan | AMPH | grey | H1F0 | grey | NAPSB | grey | TANC2 | grey |
| CNOT6L | blue | NOXO1 | blue | BTF3 | cyan | THAP9-AS1 | cyan | AMT | grey | H2AFY2 | grey | NARS2 | grey | TANGO2 | grey |
| CNPY4 | blue | NPAS1 | blue | BTG1 | cyan | THEG5 | cyan | ANAPC11 | grey | H3F3A | grey | NAT1 | grey | TANGO6 | grey |
| CNTD1 | blue | NPAS4 | blue | BTN3A3 | cyan | THEM4 | cyan | ANAPC15 | grey | HAAO | grey | NAT10 | grey | TAP1 | grey |
| CNTLN | blue | NPAT | blue | BUB3 | cyan | THOC7 | cyan | ANGPT2 | grey | HABP2 | grey | NATD1 | grey | TAP2 | grey |
| CNTN2 | blue | NPBWR1 | blue | BZW1 | cyan | THRB | cyan | ANGPT4 | grey | HACE1 | grey | NAV2-AS2 | grey | TAPBPL | grey |
| CNTROB | blue | NPC1L1 | blue | C10orf53 | cyan | THUMPD1 | cyan | ANGPTL6 | grey | HAND1 | grey | NAV2-AS5 | grey | TAPT1-AS1 | grey |
| COA3 | blue | NPFFR1 | blue | C11orf58 | cyan | THYN1 | cyan | ANGPTL7 | grey | HAPLN4 | grey | NBL1 | grey | TARBP1 | grey |
| COG4 | blue | NPHP4 | blue | C11orf73 | cyan | TIA1 | cyan | ANHX | grey | HAS2 | grey | NCAPD3 | grey | TARBP2 | grey |
| COL13A1 | blue | NPHS1 | blue | C11orf94 | cyan | TIAL1 | cyan | ANKDD1A | grey | HAUS1 | grey | NCAPG | grey | TARP | grey |
| COL18A1-AS1 | blue | NPM2 | blue | C12orf4 | cyan | TIGD3 | cyan | ANKLE1 | grey | HAUS3 | grey | NCAPG2 | grey | TAS1R2 | grey |
| COL19A1 | blue | NPM3 | blue | C12orf50 | cyan | TIMM10B | cyan | ANKRA2 | grey | HAVCR2 | grey | NCAPH | grey | TAS2R1 | grey |
| COL20A1 | blue | NPPA | blue | C12orf54 | cyan | TIMM9 | cyan | ANKRD1 | grey | HAX1 | grey | NCBP1 | grey | TAS2R10 | grey |
| COL23A1 | blue | NPPB | blue | C12orf60 | cyan | TIMMDC1 | cyan | ANKRD13A | grey | HBB | grey | NCBP2-AS2 | grey | TAS2R13 | grey |
| COL26A1 | blue | NPPC | blue | C14orf119 | cyan | TINF2 | cyan | ANKRD13B | grey | HBBP1 | grey | NCEH1 | grey | TAS2R19 | grey |
| COMMD10 | blue | NPRL2 | blue | C14orf166 | cyan | TIPRL | cyan | ANKRD18B | grey | HBD | grey | NCF1 | grey | TAS2R3 | grey |
| COMMD2 | blue | NPW | blue | C14orf2 | cyan | TJP1 | cyan | ANKRD19P | grey | HBEGF | grey | NCF2 | grey | TAS2R38 | grey |
| COMMD5 | blue | NR0B1 | blue | C14orf28 | cyan | TM2D3 | cyan | ANKRD20A11P | grey | HCAR1 | grey | NCF4 | grey | TAS2R4 | grey |
| COMP | blue | NR0B2 | blue | C15orf32 | cyan | TM9SF2 | cyan | ANKRD22 | grey | HCAR3 | grey | NCK1-AS1 | grey | TAS2R41 | grey |
| COMTD1 | blue | NR1I2 | blue | C15orf61 | cyan | TM9SF3 | cyan | ANKRD23 | grey | HCCAT5 | grey | NCKAP1L | grey | TAS2R5 | grey |
| COPA | blue | NR2C2AP | blue | C16orf72 | cyan | TM9SF4 | cyan | ANKRD26P3 | grey | HCCS | grey | NCOR2 | grey | TAS2R50 | grey |
| CORO1B | blue | NR2E3 | blue | C16orf78 | cyan | TMA7 | cyan | ANKRD30A | grey | HCFC1R1 | grey | ND6 | grey | TAS2R7 | grey |
| CORO7 | blue | NR2F6 | blue | C17orf104 | cyan | TMBIM4 | cyan | ANKRD30BP2 | grey | HCG11 | grey | NDNL2 | grey | TAS2R9 | grey |
| CORT | blue | NR5A1 | blue | C18orf21 | cyan | TMBIM6 | cyan | ANKRD33 | grey | HCG22 | grey | NDP | grey | TBC1D10B | grey |
| COX19 | blue | NR6A1 | blue | C19orf66 | cyan | TMCO1 | cyan | ANKRD33B | grey | HCG26 | grey | NDRG1 | grey | TBC1D10C | grey |
| COX6B2 | blue | NRARP | blue | C19orf73 | cyan | TMED10 | cyan | ANKRD34C | grey | HCG27 | grey | NDRG4 | grey | TBC1D12 | grey |
| CPA1 | blue | NRAS | blue | C1D | cyan | TMED2 | cyan | ANKRD36BP2 | grey | HCG4B | grey | NDST1 | grey | TBC1D14 | grey |
| CPA5 | blue | NRIP2 | blue | C1GALT1 | cyan | TMED6 | cyan | ANKRD37 | grey | HCK | grey | NDUFA11 | grey | TBC1D16 | grey |
| CPEB2-AS1 | blue | NRL | blue | C1RL | cyan | TMEM106B | cyan | ANKRD46 | grey | HCLS1 | grey | NDUFA13 | grey | TBC1D17 | grey |
| CPEB4 | blue | NRSN2 | blue | C1orf100 | cyan | TMEM123 | cyan | ANKRD50 | grey | HCP5 | grey | NDUFA9 | grey | TBC1D25 | grey |
| CPLX2 | blue | NS3BP | blue | C1orf27 | cyan | TMEM126B | cyan | ANKRD52 | grey | HCRP1 | grey | NDUFAF2 | grey | TBC1D27 | grey |
| CPLX3 | blue | NSMF | blue | C1orf43 | cyan | TMEM131 | cyan | ANKRD55 | grey | HCRTR1 | grey | NDUFAF3 | grey | TBC1D4 | grey |
| CPNE6 | blue | NT5C | blue | C1orf61 | cyan | TMEM138 | cyan | ANKS1A | grey | HCST | grey | NDUFAF4 | grey | TBC1D7 | grey |
| CPNE7 | blue | NT5DC2 | blue | C22orf15 | cyan | TMEM145 | cyan | ANKS4B | grey | HDAC1 | grey | NDUFB2 | grey | TBC1D9B | grey |
| CPNE9 | blue | NT5DC3 | blue | C22orf39 | cyan | TMEM14A | cyan | ANKZF1 | grey | HDAC5 | grey | NDUFB6 | grey | TBCC | grey |
| CPSF2 | blue | NT5M | blue | C22orf46 | cyan | TMEM14B | cyan | ANO10 | grey | HDAC7 | grey | NDUFB7 | grey | TBL1Y | grey |
| CPSF3L | blue | NTHL1 | blue | C2CD2L | cyan | TMEM14C | cyan | ANO5 | grey | HDC | grey | NDUFC1 | grey | TBP | grey |
| CPT1C | blue | NTM | blue | C2CD5 | cyan | TMEM155 | cyan | ANO7 | grey | HDDC3 | grey | NDUFS2 | grey | TBX15 | grey |
| CRADD | blue | NTMT1 | blue | C2orf49 | cyan | TMEM167A | cyan | ANP32D | grey | HDGFL1 | grey | NDUFS3 | grey | TBX20 | grey |
| CRAT | blue | NTN3 | blue | C2orf50 | cyan | TMEM167B | cyan | ANXA1 | grey | HDX | grey | NDUFS4 | grey | TBX21 | grey |
| CRB2 | blue | NTN5 | blue | C2orf76 | cyan | TMEM168 | cyan | ANXA10 | grey | HEATR3 | grey | NDUFS6 | grey | TCEA3 | grey |
| CREB3L3 | blue | NTNG2 | blue | C3orf17 | cyan | TMEM181 | cyan | ANXA2 | grey | HEBP2 | grey | NDUFV3 | grey | TCEAL2 | grey |
| CREB3L4 | blue | NTRK1 | blue | C3orf36 | cyan | TMEM185B | cyan | ANXA2P1 | grey | HECW2 | grey | NEB | grey | TCEAL7 | grey |
| CRH | blue | NTRK3-AS1 | blue | C4orf3 | cyan | TMEM207 | cyan | ANXA2P3 | grey | HEMGN | grey | NEDD4 | grey | TCEB2 | grey |
| CRHR1 | blue | NTSR1 | blue | C5orf22 | cyan | TMEM230 | cyan | ANXA2R | grey | HENMT1 | grey | NEDD9 | grey | TCEB3-AS1 | grey |
| CRIP3 | blue | NUBP2 | blue | C5orf56 | cyan | TMEM245 | cyan | AOC1 | grey | HEPH | grey | NEFM | grey | TCERG1L | grey |
| CRIPAK | blue | NUDT1 | blue | C6orf106 | cyan | TMEM248 | cyan | AOC2 | grey | HERC5 | grey | NEK4 | grey | TCF19 | grey |
| CRIPT | blue | NUDT14 | blue | C6orf118 | cyan | TMEM261 | cyan | AOX2P | grey | HES1 | grey | NEK8 | grey | TCF24 | grey |
| CRLF1 | blue | NUDT16L1 | blue | C6orf120 | cyan | TMEM263 | cyan | AP000230.1 | grey | HEXIM1 | grey | NELFB | grey | TCF7 | grey |
| CRLF3 | blue | NUDT18 | blue | C6orf62 | cyan | TMEM30A | cyan | AP000253.1 | grey | HEY1 | grey | NELL1 | grey | TCHH | grey |
| CRNKL1 | blue | NUDT22 | blue | C6orf89 | cyan | TMEM33 | cyan | AP000462.1 | grey | HEY2 | grey | NELL2 | grey | TCIRG1 | grey |
| CRNN | blue | NUDT4 | blue | C7orf34 | cyan | TMEM41B | cyan | AP000473.8 | grey | HEYL | grey | NEO1 | grey | TCL1A | grey |
| CRP | blue | NUP153 | blue | C7orf49 | cyan | TMEM47 | cyan | AP000525.9 | grey | HFE2 | grey | NEU1 | grey | TCL1B | grey |
| CRTC1 | blue | NUP155 | blue | C8A | cyan | TMEM5 | cyan | AP001171.1 | grey | HGH1 | grey | NEURL2 | grey | TCP10 | grey |
| CRX | blue | NUP205 | blue | C9orf135-AS1 | cyan | TMEM50A | cyan | AP001189.4 | grey | HHAT | grey | NEUROD4 | grey | TCP11 | grey |
| CRYAA | blue | NUP54 | blue | CAB39 | cyan | TMEM57 | cyan | AP001605.4 | grey | HHATL | grey | NEUROD6 | grey | TCP11L2 | grey |
| CRYBA1 | blue | NUPL1 | blue | CACNA1F | cyan | TMEM59 | cyan | AP006222.2 | grey | HHEX | grey | NEXN | grey | TCRBV15S1 | grey |
| CRYBA2 | blue | NUTM2B | blue | CACNA1G-AS1 | cyan | TMEM69 | cyan | AP1B1 | grey | HHIP-AS1 | grey | NFATC4 | grey | TCTE1 | grey |
| CRYBA4 | blue | NXF5 | blue | CACYBP | cyan | TMEM79 | cyan | AP1M1 | grey | HHIPL1 | grey | NFIL3 | grey | TCTEX1D2 | grey |
| CRYBB1 | blue | NXT2 | blue | CALCOCO1 | cyan | TMEM87A | cyan | AP2M1 | grey | HHIPL2 | grey | NFIX | grey | TCTN1 | grey |
| CRYBB2 | blue | NYX | blue | CALCOCO2 | cyan | TMEM91 | cyan | AP4M1 | grey | HHLA3 | grey | NFKB2 | grey | TCTN2 | grey |
| CRYBB2P1 | blue | Ndufaf4 | blue | CALML3 | cyan | TMEM9B | cyan | AP5B1 | grey | HIATL2 | grey | NFKBIA | grey | TDRD10 | grey |
| CRYBB3 | blue | OAZ3 | blue | CALU | cyan | TMPRSS11D | cyan | AP5S1 | grey | HIBCH | grey | NFKBID | grey | TDRD12 | grey |
| CRYGA | blue | OBP2A | blue | CAMSAP2 | cyan | TMPRSS13 | cyan | APAF1 | grey | HIC1 | grey | NFKBIE | grey | TDRD5 | grey |
| CRYGC | blue | OBP2B | blue | CAMTA1 | cyan | TMX2 | cyan | APBA1 | grey | HINFP | grey | NFS1 | grey | TDRD6 | grey |
| CRYGD | blue | OBSCN | blue | CANX | cyan | TNFSF10 | cyan | APBA3 | grey | HINT2 | grey | NFXL1 | grey | TDRD7 | grey |
| CRYGEP | blue | OCEL1 | blue | CAP1 | cyan | TNNC1 | cyan | APCS | grey | HINT3 | grey | NFYC-AS1 | grey | TDRG1 | grey |
| CRYM | blue | OCRL | blue | CAPN2 | cyan | TNNI2 | cyan | APEH | grey | HIPK4 | grey | NGFR | grey | TEAD3 | grey |
| CSDC2 | blue | ODF3 | blue | CAPNS1 | cyan | TNNI3 | cyan | APEX2 | grey | HIST1H1A | grey | NHEG1 | grey | TEAD4 | grey |
| CSF2 | blue | ODF3B | blue | CAPNS2 | cyan | TNPO1 | cyan | APIP | grey | HIST1H1C | grey | NHLH1 | grey | TEC | grey |
| CSF2RA | blue | ODF3L2 | blue | CAPRIN1 | cyan | TNRC6B | cyan | APOA1 | grey | HIST1H1D | grey | NHLH2 | grey | TECTB | grey |
| CSF3 | blue | OGDHL | blue | CAPZA1 | cyan | TOMM20 | cyan | APOA1BP | grey | HIST1H1E | grey | NHP2 | grey | TEF | grey |
| CSHL1 | blue | OGFOD2 | blue | CAPZA2 | cyan | TOMM5 | cyan | APOA2 | grey | HIST1H2AC | grey | NHSL2 | grey | TEK | grey |
| CSMD2 | blue | OGFR | blue | CARM1 | cyan | TOMM70A | cyan | APOB | grey | HIST1H2AE | grey | NICN1 | grey | TEKT3 | grey |
| CSNK1G2-AS1 | blue | OLA1 | blue | CASC15 | cyan | TOP1 | cyan | APOBEC2 | grey | HIST1H2AJ | grey | NIF3L1 | grey | TENM1 | grey |
| CSNK2A2 | blue | OLIG2 | blue | CASP14 | cyan | TOP2B | cyan | APOBEC3D | grey | HIST1H2AK | grey | NINJ1 | grey | TENM2 | grey |
| CSPG4 | blue | OMP | blue | CAST | cyan | TOPORS | cyan | APOBEC3F | grey | HIST1H2AL | grey | NINJ2 | grey | TENM3 | grey |
| CSPG4P1Y | blue | ONECUT3 | blue | CAV3 | cyan | TOR1AIP1 | cyan | APOBEC3G | grey | HIST1H2BA | grey | NINL | grey | TENM4 | grey |
| CST9 | blue | OPA1 | blue | CBFB | cyan | TP53AIP1 | cyan | APOBEC4 | grey | HIST1H2BC | grey | NIPA1 | grey | TEPP | grey |
| CST9L | blue | OPCML | blue | CBLB | cyan | TPH1 | cyan | APOC1 | grey | HIST1H2BM | grey | NIPSNAP3B | grey | TESK1 | grey |
| CSTF2T | blue | OPLAH | blue | CBX1 | cyan | TPI1 | cyan | APOH | grey | HIST1H2BO | grey | NIT2 | grey | TESK2 | grey |
| CSTF3 | blue | OPN1SW | blue | CBX3 | cyan | TPM1 | cyan | APOL1 | grey | HIST1H3A | grey | NKAIN3 | grey | TESPA1 | grey |
| CT62 | blue | OPN3 | blue | CBX5 | cyan | TPR | cyan | APOL3 | grey | HIST1H3B | grey | NKAP | grey | TEX14 | grey |
| CTA-384D8.35 | blue | OPRD1 | blue | CCDC104 | cyan | TPRA1 | cyan | APOL4 | grey | HIST1H3C | grey | NKD1 | grey | TEX22 | grey |
| CTAG2 | blue | OPRL1 | blue | CCDC157 | cyan | TPRKB | cyan | APOLD1 | grey | HIST1H3E | grey | NKG7 | grey | TEX26 | grey |
| CTB-25B13.12 | blue | OR10A4 | blue | CCDC182 | cyan | TPRN | cyan | APPL2 | grey | HIST1H3F | grey | NKRF | grey | TEX33 | grey |
| CTB-31O20.9 | blue | OR10D1P | blue | CCDC186 | cyan | TPT1-AS1 | cyan | AQP1 | grey | HIST1H3G | grey | NKX3-1 | grey | TEX36 | grey |
| CTB-43E15.1 | blue | OR10H1 | blue | CCDC28A | cyan | TRAK2 | cyan | AQP10 | grey | HIST1H3I | grey | NKX3-2 | grey | TEX36-AS1 | grey |
| CTB-50L17.7 | blue | OR10H2 | blue | CCDC37-AS1 | cyan | TRAM1 | cyan | AQP9 | grey | HIST1H4A | grey | NLGN1 | grey | TEX37 | grey |
| CTBP1-AS | blue | OR11A1 | blue | CCDC43 | cyan | TRAM2 | cyan | ARAP3 | grey | HIST1H4D | grey | NLRC3 | grey | TEX38 | grey |
| CTC-360P9.3 | blue | OR13C4 | blue | CCDC47 | cyan | TRAPPC1 | cyan | ARC | grey | HIST1H4E | grey | NLRC5 | grey | TEX41 | grey |
| CTC-425F1.4 | blue | OR1I1 | blue | CCDC53 | cyan | TRAPPC8 | cyan | ARF1 | grey | HIST1H4J | grey | NLRP1 | grey | TEX43 | grey |
| CTC-444N24.11 | blue | OR1J2 | blue | CCDC59 | cyan | TREML5P | cyan | ARF5 | grey | HIST1H4L | grey | NLRP10 | grey | TEX9 | grey |
| CTC-510F12.4 | blue | OR2C1 | blue | CCDC64B | cyan | TRIM10 | cyan | ARG2 | grey | HIST2H2BE | grey | NLRP11 | grey | TF | grey |
| CTC-550B14.7 | blue | OR2F1 | blue | CCDC70 | cyan | TRIM21 | cyan | ARHGAP10 | grey | HIVEP2 | grey | NLRP13 | grey | TFAP2E | grey |
| CTCF | blue | OR2H2 | blue | CCDC81 | cyan | TRIM22 | cyan | ARHGAP11A | grey | HJURP | grey | NLRP14 | grey | TFE3 | grey |
| CTD-2012K14.6 | blue | OR2K2 | blue | CCDC86 | cyan | TRIM33 | cyan | ARHGAP20 | grey | HLA-A | grey | NLRP3 | grey | TFF1 | grey |
| CTD-2033C11.1 | blue | OR2L1P | blue | CCDC92 | cyan | TRIM36 | cyan | ARHGAP24 | grey | HLA-B | grey | NLRP9 | grey | TFF3 | grey |
| CTD-2083E4.7 | blue | OR2L2 | blue | CCL27 | cyan | TRIM4 | cyan | ARHGAP26 | grey | HLA-DMA | grey | NMB | grey | TFIP11 | grey |
| CTD-2130O13.1 | blue | OR3A1 | blue | CCNB1IP1 | cyan | TRIM41 | cyan | ARHGAP26-AS1 | grey | HLA-DMB | grey | NME1 | grey | TFPI2 | grey |
| CTD-2256P15.2 | blue | OR3A2 | blue | CCNDBP1 | cyan | TRIM52 | cyan | ARHGAP28 | grey | HLA-DOA | grey | NME4 | grey | TFRC | grey |
| CTD-2269F5.1 | blue | OR3A3 | blue | CCNG1 | cyan | TRIM61 | cyan | ARHGAP30 | grey | HLA-DOB | grey | NME5 | grey | TG | grey |
| CTD-2281E23.2 | blue | OR4D1 | blue | CCNG2 | cyan | TRIP11 | cyan | ARHGAP31 | grey | HLA-DPA1 | grey | NME6 | grey | TGFB1 | grey |
| CTD-2287O16.5 | blue | OR5AK4P | blue | CCNH | cyan | TRIP12 | cyan | ARHGAP39 | grey | HLA-DPB1 | grey | NME8 | grey | TGFB1I1 | grey |
| CTD-2534I21.8 | blue | OR6W1P | blue | CCNI | cyan | TRMT112 | cyan | ARHGAP42 | grey | HLA-DPB2 | grey | NME9 | grey | TGIF2 | grey |
| CTD-2541M15.1 | blue | OR7A17 | blue | CCNK | cyan | TRMT13 | cyan | ARHGAP6 | grey | HLA-DQA1 | grey | NMRAL1 | grey | TGIF2LY | grey |
| CTD-2547L24.4 | blue | OR7C2 | blue | CCNL1 | cyan | TRMT1L | cyan | ARHGAP9 | grey | HLA-DQB1 | grey | NMRK1 | grey | TGM2 | grey |
| CTD-2587H24.10 | blue | OR7E104P | blue | CCNT1 | cyan | TRMT5 | cyan | ARHGEF1 | grey | HLA-DQB2 | grey | NMUR2 | grey | TGM3 | grey |
| CTD-3028N15.1 | blue | OR7E12P | blue | CCNYL1 | cyan | TROVE2 | cyan | ARHGEF11 | grey | HLA-DRA | grey | NNT-AS1 | grey | TGM5 | grey |
| CTD-3064M3.3 | blue | ORC2 | blue | CCT2 | cyan | TRPM1 | cyan | ARHGEF17 | grey | HLA-DRB4 | grey | NOC3L | grey | TGM7 | grey |
| CTD-3080P12.3 | blue | ORF1 | blue | CD164 | cyan | TRPS1 | cyan | ARHGEF18 | grey | HLA-E | grey | NOD1 | grey | THAP10 | grey |
| CTD-3126B10.1 | blue | ORM1 | blue | CD46 | cyan | TRPV5 | cyan | ARHGEF25 | grey | HLA-F | grey | NOD2 | grey | THAP11 | grey |
| CTD-3193O13.1 | blue | OSBP2 | blue | CD47 | cyan | TRRAP | cyan | ARHGEF26 | grey | HLA-F-AS1 | grey | NOG | grey | THBD | grey |
| CTDSPL2 | blue | OSBPL5 | blue | CD55 | cyan | TSC1 | cyan | ARHGEF3 | grey | HLA-G | grey | NOL10 | grey | THBS1 | grey |
| CTF1 | blue | OSBPL7 | blue | CD59 | cyan | TSG101 | cyan | ARHGEF33 | grey | HLA-J | grey | NOL11 | grey | THBS3 | grey |
| CTIF | blue | OSCAR | blue | CD63 | cyan | TSN | cyan | ARHGEF38 | grey | HMCN1 | grey | NOP10 | grey | THBS4 | grey |
| CTLA4 | blue | OSGIN1 | blue | CDC16 | cyan | TSNAX | cyan | ARHGEF4 | grey | HMG20A | grey | NOP14 | grey | THEM5 | grey |
| CTR9 | blue | OSM | blue | CDC26 | cyan | TSNAXIP1 | cyan | ARHGEF7-AS2 | grey | HMGB3 | grey | NOP2 | grey | THEM6 | grey |
| CTRC | blue | OTOF | blue | CDC27 | cyan | TSPAN31 | cyan | ARHGEF7-IT1 | grey | HMGB3P1 | grey | NOTCH1 | grey | THEMIS2 | grey |
| CTRL | blue | OTOP2 | blue | CDC40 | cyan | TSPAN6 | cyan | ARID5A | grey | HMGCR | grey | NOTCH2NL | grey | THG1L | grey |
| CTU2 | blue | OTOR | blue | CDC42SE2 | cyan | TSPEAR | cyan | ARIH2OS | grey | HMGCS1 | grey | NOTCH3 | grey | THNSL2 | grey |
| CTXN1 | blue | OTOS | blue | CDC5L | cyan | TSPYL1 | cyan | ARL13B | grey | HMGN5 | grey | NOV | grey | THSD7A | grey |
| CUL3 | blue | OTP | blue | CDHR1 | cyan | TSPYL4 | cyan | ARL14 | grey | HMHA1 | grey | NOVA1 | grey | THSD7B | grey |
| CUL7 | blue | OTUB2 | blue | CDIPT | cyan | TSPYL5 | cyan | ARL14EP | grey | HMOX1 | grey | NOXRED1 | grey | THTPA | grey |
| CUL9 | blue | OTUD4 | blue | CDK14 | cyan | TTC14 | cyan | ARL2 | grey | HMP19 | grey | NPAP1 | grey | THUMPD2 | grey |
| CWC22 | blue | OTUD7A | blue | CDK17 | cyan | TTC17 | cyan | ARL4A | grey | HMX2 | grey | NPBWR2 | grey | THY1 | grey |
| CXCL3 | blue | OVCH1-AS1 | blue | CDK2AP1 | cyan | TTC19 | cyan | ARL4C | grey | HN1 | grey | NPDC1 | grey | TIAF1 | grey |
| CXCR3 | blue | OVOL3 | blue | CDK4 | cyan | TTC23L | cyan | ARL8A | grey | HNRNPF | grey | NPFF | grey | TIAM1 | grey |
| CXorf30 | blue | OXCT2 | blue | CDKN1B | cyan | TTC37 | cyan | ARL9 | grey | HOGA1 | grey | NPHP3 | grey | TIFAB | grey |
| CXorf31 | blue | OXLD1 | blue | CDS2 | cyan | TTC7B | cyan | ARMC12 | grey | HOMEZ | grey | NPHP3-AS1 | grey | TIGD1 | grey |
| CXorf58 | blue | OXSR1 | blue | CDT1 | cyan | TTTY7 | cyan | ARMC2 | grey | HOOK2 | grey | NPHS2 | grey | TIGD2 | grey |
| CYAT1 | blue | OXT | blue | CDV3 | cyan | TUBB | cyan | ARMCX6 | grey | HOPX | grey | NPIPA1 | grey | TIGD7 | grey |
| CYB561D1 | blue | P2RX2 | blue | CEACAM4 | cyan | TUBBP5 | cyan | ARNTL | grey | HOTS | grey | NPIPB15 | grey | TIGIT | grey |
| CYB5RL | blue | P2RX3 | blue | CEBPZ | cyan | TUBGCP4 | cyan | ARPC1B | grey | HOXA1 | grey | NPL | grey | TIMD4 | grey |
| CYHR1 | blue | P2RX6 | blue | CECR1 | cyan | TUG1 | cyan | ARPC4 | grey | HOXA2 | grey | NPLOC4 | grey | TIMM10 | grey |
| CYMP | blue | PACSIN1 | blue | CELF4 | cyan | TUSC1 | cyan | ARRB1 | grey | HOXB2 | grey | NPNT | grey | TIMM22 | grey |
| CYP11B1 | blue | PACSIN3 | blue | CELF5 | cyan | TVP23B | cyan | ARRB2 | grey | HOXB6 | grey | NPRL3 | grey | TIMM23 | grey |
| CYP11B2 | blue | PADI1 | blue | CEP120 | cyan | TWSG1 | cyan | ARRDC1 | grey | HOXB7 | grey | NPTX1 | grey | TIMM8B | grey |
| CYP17A1 | blue | PAGE1 | blue | CEP350 | cyan | TXLNG | cyan | ARRDC2 | grey | HOXC11 | grey | NPY | grey | TIMP1 | grey |
| CYP2A13 | blue | PAGE5 | blue | CEP57 | cyan | TXN | cyan | ARSJ | grey | HOXC13 | grey | NPY1R | grey | TIPARP | grey |
| CYP2D6 | blue | PAGR1 | blue | CEP63 | cyan | TXNDC12 | cyan | ART5 | grey | HOXC4 | grey | NPY5R | grey | TIPARP-AS1 | grey |
| CYP2W1 | blue | PAK1IP1 | blue | CEPT1 | cyan | TXNDC15 | cyan | ARTN | grey | HOXC6 | grey | NR1H2 | grey | TIPIN | grey |
| CYP46A1 | blue | PALD1 | blue | CERS6 | cyan | TXNDC9 | cyan | AS3MT | grey | HOXC8 | grey | NR1H3 | grey | TJAP1 | grey |
| CYP4F12 | blue | PANX2 | blue | CETN2 | cyan | TXNIP | cyan | ASAP1-IT1 | grey | HOXC9 | grey | NR2C1 | grey | TLCD1 | grey |
| CYP4F2 | blue | PAOX | blue | CETN3 | cyan | TYMP | cyan | ASAP1-IT2 | grey | HOXD-AS2 | grey | NR2E1 | grey | TLCD2 | grey |
| CYP4F22 | blue | PAPD4 | blue | CFC1 | cyan | TYW3 | cyan | ASAP3 | grey | HOXD4 | grey | NR2F2-AS1 | grey | TLE1 | grey |
| CYP4F62P | blue | PAPD5 | blue | CFDP1 | cyan | U91328.2 | cyan | ASB11 | grey | HOXD8 | grey | NR4A1 | grey | TLL1 | grey |
| CYSRT1 | blue | PAPOLG | blue | CFL2 | cyan | UBA2 | cyan | ASB12 | grey | HP | grey | NR4A2 | grey | TLR1 | grey |
| D2HGDH | blue | PAQR4 | blue | CFLAR | cyan | UBA3 | cyan | ASB13 | grey | HP09025 | grey | NR4A3 | grey | TLR10 | grey |
| DACT3 | blue | PAQR6 | blue | CGGBP1 | cyan | UBAP1 | cyan | ASB16 | grey | HPCAL1 | grey | NRAP | grey | TLR2 | grey |
| DAGLA | blue | PARD6A | blue | CHCHD2 | cyan | UBE2A | cyan | ASB16-AS1 | grey | HPD | grey | NRBF2 | grey | TLR5 | grey |
| DALRD3 | blue | PARP10 | blue | CHD6 | cyan | UBE2D2 | cyan | ASB8 | grey | HPGD | grey | NRBP1 | grey | TLR6 | grey |
| DAO | blue | PARP3 | blue | CHD9 | cyan | UBE2D3 | cyan | ASCL3 | grey | HPS5 | grey | NREP | grey | TLR8 | grey |
| DAPK2 | blue | PARP8 | blue | CHIC2 | cyan | UBE2E1 | cyan | ASF1B | grey | HPSE | grey | NRG1-IT1 | grey | TLR8-AS1 | grey |
| DAPK3 | blue | PASD1 | blue | CHMP2B | cyan | UBE2E2 | cyan | ASH1L-AS1 | grey | HPSE2 | grey | NRG2 | grey | TLR9 | grey |
| DBH | blue | PATE1 | blue | CHMP4B | cyan | UBE2E3 | cyan | ASL | grey | HPYR1 | grey | NRG3 | grey | TLX1 | grey |
| DBH-AS1 | blue | PAX1 | blue | CHMP5 | cyan | UBE2F | cyan | ASMTL | grey | HRC | grey | NRGN | grey | TLX3 | grey |
| DBR1 | blue | PAX2 | blue | CHP1 | cyan | UBE2G1 | cyan | ASNA1 | grey | HRCT1 | grey | NRK | grey | TM2D2 | grey |
| DCAF10 | blue | PAX3 | blue | CHPT1 | cyan | UBE2H | cyan | ASNSD1 | grey | HRH1 | grey | NRM | grey | TM4SF18 | grey |
| DCAF13 | blue | PAX8 | blue | CHRAC1 | cyan | UBE2K | cyan | ASPHD2 | grey | HS3ST2 | grey | NRROS | grey | TM4SF4 | grey |
| DCAF15 | blue | PAXBP1 | blue | CHST14 | cyan | UBE2L3 | cyan | ASPM | grey | HS3ST3A1 | grey | NRSN1 | grey | TM6SF1 | grey |
| DCDC2B | blue | PCBP3 | blue | CHSY1 | cyan | UBE2N | cyan | ASPN | grey | HS3ST4 | grey | NRTN | grey | TM7SF3 | grey |
| DCLRE1C | blue | PCDH1 | blue | CHTF8 | cyan | UBE2Q1 | cyan | ASPSCR1 | grey | HSCB | grey | NRXN2 | grey | TM9SF1 | grey |
| DCP1A | blue | PCDHA10 | blue | CHURC1 | cyan | UBE2Q2 | cyan | ASRGL1 | grey | HSD11B2 | grey | NSAP11 | grey | TMA16 | grey |
| DCTN2 | blue | PCDHA5 | blue | CIR1 | cyan | UBE2R2 | cyan | ASS1 | grey | HSD17B10 | grey | NSDHL | grey | TMC1 | grey |
| DCTN4 | blue | PCDHB18 | blue | CIRH1A | cyan | UBE3A | cyan | ASTE1 | grey | HSD17B3 | grey | NSF | grey | TMC2 | grey |
| DCUN1D1 | blue | PCDHGC4 | blue | CISD2 | cyan | UBE4A | cyan | ASTN1 | grey | HSD17B7 | grey | NSRP1 | grey | TMC5 | grey |
| DDC | blue | PCDHGC5 | blue | CKAP5 | cyan | UBE4B | cyan | ASUN | grey | HSD17B7P2 | grey | NSUN2 | grey | TMC6 | grey |
| DDN | blue | PCNA | blue | CLCC1 | cyan | UBL3 | cyan | ATCAY | grey | HSD17B8 | grey | NSUN5 | grey | TMCC1-AS1 | grey |
| DDR1-AS1 | blue | PCNXL4 | blue | CLCN3 | cyan | UBLCP1 | cyan | ATF3 | grey | HSD3B2 | grey | NSUN5P1 | grey | TMCC3 | grey |
| DDX18 | blue | PCP2 | blue | CLDND1 | cyan | UBN2 | cyan | ATF7IP2 | grey | HSD3B7 | grey | NT5C1A | grey | TMCO2 | grey |
| DDX28 | blue | PCSK1N | blue | CLEC4C | cyan | UBP1 | cyan | ATG101 | grey | HSDL1 | grey | NT5C3B | grey | TMCO6 | grey |
| DDX42 | blue | PCSK4 | blue | CLIC2 | cyan | UBQLN1 | cyan | ATG4B | grey | HSF1 | grey | NTF3 | grey | TMEM100 | grey |
| DDX46 | blue | PCSK9 | blue | CLIC4 | cyan | UBR1 | cyan | ATG4C | grey | HSF2BP | grey | NTN1 | grey | TMEM108-AS1 | grey |
| DDX49 | blue | PCYT1B | blue | CLK1 | cyan | UBR5 | cyan | ATG9A | grey | HSPA12A | grey | NTPCR | grey | TMEM109 | grey |
| DDX51 | blue | PCYT2 | blue | CLK4 | cyan | UBR7 | cyan | ATL1 | grey | HSPA1L | grey | NTSR2 | grey | TMEM11 | grey |
| DDX54 | blue | PDCD1 | blue | CLNS1A | cyan | UBTD2 | cyan | ATN1 | grey | HSPA2 | grey | NUBPL | grey | TMEM115 | grey |
| DEFA4 | blue | PDCD10 | blue | CLOCK | cyan | UBXN10 | cyan | ATOH1 | grey | HSPA6 | grey | NUCB1 | grey | TMEM117 | grey |
| DEFB123 | blue | PDCD11 | blue | CLTA | cyan | UBXN4 | cyan | ATOH8 | grey | HSPB1 | grey | NUDT11 | grey | TMEM119 | grey |
| DEGS2 | blue | PDE1B | blue | CMAHP | cyan | UCHL3 | cyan | ATOX1 | grey | HSPG2 | grey | NUDT13 | grey | TMEM120A | grey |
| DENND2C | blue | PDE4A | blue | CMPK1 | cyan | UCP1 | cyan | ATP10B | grey | HTN3 | grey | NUDT15 | grey | TMEM126A | grey |
| DENND4A | blue | PDE6B | blue | CNBP | cyan | UFC1 | cyan | ATP13A1 | grey | HTR1B | grey | NUDT2 | grey | TMEM128 | grey |
| DENND4B | blue | PDGFB | blue | CNEP1R1 | cyan | UFD1L | cyan | ATP13A5 | grey | HTR1E | grey | NUDT6 | grey | TMEM130 | grey |
| DENND6A | blue | PDIA2 | blue | CNN2 | cyan | UFL1 | cyan | ATP1B2 | grey | HTR1F | grey | NUDT9 | grey | TMEM132D | grey |
| DFFB | blue | PDLIM2 | blue | CNOT1 | cyan | UFM1 | cyan | ATP1B3 | grey | HTR2B | grey | NUF2 | grey | TMEM133 | grey |
| DGCR10 | blue | PDLIM4 | blue | CNOT11 | cyan | UGDH | cyan | ATP1B4 | grey | HTR3A | grey | NUMA1 | grey | TMEM143 | grey |
| DGCR14 | blue | PDLIM5 | blue | CNOT7 | cyan | UGGT1 | cyan | ATP2A2 | grey | HTR3B | grey | NUMBL | grey | TMEM147 | grey |
| DGCR5 | blue | PDX1 | blue | CNOT8 | cyan | UGP2 | cyan | ATP5B | grey | HTR3C | grey | NUP107 | grey | TMEM150A | grey |
| DGCR6L | blue | PDZD3 | blue | CNTNAP5 | cyan | UGT1A1 | cyan | ATP5D | grey | HTRA3 | grey | NUP210L | grey | TMEM150B | grey |
| DGCR7 | blue | PDZD4 | blue | COBLL1 | cyan | UHMK1 | cyan | ATP5G1 | grey | HTRA4 | grey | NUP210P1 | grey | TMEM150C | grey |
| DGKZ | blue | PDZD7 | blue | COG5 | cyan | ULBP1 | cyan | ATP5G2 | grey | HUNK | grey | NUP35 | grey | TMEM154 | grey |
| DHDH | blue | PEG3-AS1 | blue | COL25A1 | cyan | ULBP2 | cyan | ATP5G3 | grey | HVCN1 | grey | NUP85 | grey | TMEM156 | grey |
| DHH | blue | PELI1 | blue | COL4A3BP | cyan | UPF2 | cyan | ATP6 | grey | HYAL1 | grey | NUP88 | grey | TMEM158 | grey |
| DHODH | blue | PERM1 | blue | COL7A1 | cyan | UQCRB | cyan | ATP6V0A1 | grey | HYDIN | grey | NUP98 | grey | TMEM159 | grey |
| DHRS12 | blue | PES1 | blue | COL9A1 | cyan | UQCRC2 | cyan | ATP6V0B | grey | HYI | grey | NUPR1 | grey | TMEM170B | grey |
| DHRS13 | blue | PEX1 | blue | COLQ | cyan | URI1 | cyan | ATP6V0E2 | grey | HYKK | grey | NVL | grey | TMEM171 | grey |
| DHRS7 | blue | PEX11G | blue | COMMD3 | cyan | USMG5 | cyan | ATP6V1E2 | grey | HYLS1 | grey | NXN | grey | TMEM173 | grey |
| DHRS7C | blue | PEX13 | blue | COMMD6 | cyan | USO1 | cyan | ATP6V1G2 | grey | HYMAI | grey | NXPE3 | grey | TMEM176B | grey |
| DHX34 | blue | PEX16 | blue | COPB1 | cyan | USP1 | cyan | ATP6V1G3 | grey | HYPK | grey | NXPE4 | grey | TMEM178A | grey |
| DHX37 | blue | PFAS | blue | COPB2 | cyan | USP10 | cyan | ATP8A1 | grey | ICAM1 | grey | NXPH4 | grey | TMEM180 | grey |
| DHX58 | blue | PFKFB4 | blue | COPS2 | cyan | USP11 | cyan | ATP8B1 | grey | ICAM2 | grey | NYAP2 | grey | TMEM184B | grey |
| DIEXF | blue | PFKL | blue | COPS3 | cyan | USP14 | cyan | ATP8B2 | grey | ICAM4 | grey | OAF | grey | TMEM187 | grey |
| DIO3 | blue | PFN4 | blue | COPS5 | cyan | USP16 | cyan | ATP8B4 | grey | ICMT | grey | OARD1 | grey | TMEM194A | grey |
| DIO3OS | blue | PGAP3 | blue | COX11 | cyan | USP26 | cyan | ATP8B5P | grey | ICOS | grey | OAS1 | grey | TMEM194B | grey |
| DIRAS1 | blue | PGBD2 | blue | COX16 | cyan | USP30-AS1 | cyan | ATPIF1 | grey | ICT1 | grey | OASL | grey | TMEM198B | grey |
| DISP2 | blue | PGD | blue | COX20 | cyan | USP32 | cyan | ATXN7L2 | grey | ID1 | grey | OAZ2 | grey | TMEM2 | grey |
| DKFZP434A062 | blue | PGK1 | blue | COX6C | cyan | USP32P2 | cyan | AUNIP | grey | ID2B | grey | OBFC1 | grey | TMEM200A | grey |
| DKFZP434H168 | blue | PGK2 | blue | COX7A2 | cyan | USP33 | cyan | AURKC | grey | ID3 | grey | OCA2 | grey | TMEM203 | grey |
| DKFZp434J0226 | blue | PGLYRP2 | blue | COX7A2L | cyan | USP34 | cyan | AVEN | grey | ID4 | grey | OCLM | grey | TMEM205 | grey |
| DKFZp779M0652 | blue | PGP | blue | COX7B | cyan | USP47 | cyan | AVPI1 | grey | IDH3B | grey | OCM2 | grey | TMEM208 | grey |
| DKKL1 | blue | PHACTR4 | blue | CPEB2 | cyan | USP48 | cyan | AX746830 | grey | IDI1 | grey | OCR1 | grey | TMEM209 | grey |
| DLEC1 | blue | PHAX | blue | CPNE3 | cyan | USP53 | cyan | AX746968 | grey | IDI2 | grey | ODC1 | grey | TMEM216 | grey |
| DLG1 | blue | PHC1 | blue | CPS1-IT1 | cyan | USP54 | cyan | AX747064 | grey | IDI2-AS1 | grey | ODF1 | grey | TMEM217 | grey |
| DLG4 | blue | PHC3 | blue | CPSF6 | cyan | USP9X | cyan | AX747405 | grey | IDNK | grey | ODF3L1 | grey | TMEM219 | grey |
| DLG5-AS1 | blue | PHF11 | blue | CPSF7 | cyan | UTP11L | cyan | AX747507 | grey | IDO1 | grey | ODF4 | grey | TMEM222 | grey |
| DLGAP3 | blue | PHF19 | blue | CRB3 | cyan | UTP3 | cyan | AX747630 | grey | IER2 | grey | OGDH | grey | TMEM235 | grey |
| DLL3 | blue | PHF20L1 | blue | CRBN | cyan | UTRN | cyan | AX747826 | grey | IER3 | grey | OGFRP1 | grey | TMEM239 | grey |
| DLL4 | blue | PHF7 | blue | CRCT1 | cyan | UXS1 | cyan | AX748273 | grey | IER5 | grey | OLFM2 | grey | TMEM241 | grey |
| DLX1 | blue | PHGR1 | blue | CREB1 | cyan | VAMP2 | cyan | AX748292 | grey | IFFO1 | grey | OLIG1 | grey | TMEM246 | grey |
| DLX4 | blue | PHIP | blue | CREB3L2 | cyan | VAMP3 | cyan | AX748339 | grey | IFFO2 | grey | OLR1 | grey | TMEM251 | grey |
| DMAP1 | blue | PHKB | blue | CREBL2 | cyan | VAMP7 | cyan | AXIN1 | grey | IFI27 | grey | ONECUT1 | grey | TMEM252 | grey |
| DMPK | blue | PHKG1 | blue | CREBRF | cyan | VAPB | cyan | B3GALT2 | grey | IFI27L1 | grey | OOEP | grey | TMEM253 | grey |
| DMRTA1 | blue | PHLDA2 | blue | CREG1 | cyan | VBP1 | cyan | B3GAT1 | grey | IFI44 | grey | OOSP2 | grey | TMEM254-AS1 | grey |
| DMRTB1 | blue | PHLDA3 | blue | CRHR2 | cyan | VDAC1 | cyan | B3GNT5 | grey | IFI44L | grey | OPALIN | grey | TMEM257 | grey |
| DMXL1 | blue | PHLDB3 | blue | CRK | cyan | VDAC2 | cyan | B3GNT9 | grey | IFI6 | grey | OPHN1 | grey | TMEM26 | grey |
| DMXL2 | blue | PHOX2A | blue | CRKL | cyan | VDAC3 | cyan | B4GALT5 | grey | IFIT1 | grey | OPN4 | grey | TMEM30B | grey |
| DNAAF1 | blue | PHRF1 | blue | CRNDE | cyan | VDR | cyan | BABAM1 | grey | IFIT2 | grey | OPTC | grey | TMEM35 | grey |
| DNAH17 | blue | PHYHIP | blue | CROCC | cyan | VEGFA | cyan | BAG3 | grey | IFIT5 | grey | OR10C1 | grey | TMEM39B | grey |
| DNAH17-AS1 | blue | PI16 | blue | CRYBG3 | cyan | VEZF1 | cyan | BAI2 | grey | IFITM1 | grey | OR10H3 | grey | TMEM42 | grey |
| DNAH2 | blue | PIANP | blue | CRYGB | cyan | VGLL1 | cyan | BAMBI | grey | IFNA10 | grey | OR12D2 | grey | TMEM44-AS1 | grey |
| DNAH5 | blue | PIAS2 | blue | CRYZ | cyan | VIPAS39 | cyan | BANF2 | grey | IFNA17 | grey | OR12D3 | grey | TMEM45B | grey |
| DNAH9 | blue | PIAS4 | blue | CS | cyan | VIPR2 | cyan | BANK1 | grey | IFNA21 | grey | OR14J1 | grey | TMEM52 | grey |
| DNAI1 | blue | PIBF1 | blue | CSDE1 | cyan | VKORC1 | cyan | BAP1 | grey | IFNA4 | grey | OR1A1 | grey | TMEM54 | grey |
| DNAJB8 | blue | PICK1 | blue | CSE1L | cyan | VOPP1 | cyan | BASP1 | grey | IFNE | grey | OR1A2 | grey | TMEM55A | grey |
| DNAJC16 | blue | PIDD1 | blue | CSGALNACT2 | cyan | VPS13B | cyan | BATF | grey | IFNG | grey | OR1D5 | grey | TMEM55B | grey |
| DNAJC2 | blue | PIEZO1 | blue | CSNK1A1 | cyan | VPS29 | cyan | BATF2 | grey | IFNK | grey | OR1F1 | grey | TMEM60 | grey |
| DNAJC27-AS1 | blue | PIGG | blue | CSNK2A1 | cyan | VPS35 | cyan | BATF3 | grey | IFRD1 | grey | OR1F2P | grey | TMEM68 | grey |
| DNAJC4 | blue | PIGQ | blue | CSNK2B | cyan | VPS37A | cyan | BAX | grey | IFRD2 | grey | OR1G1 | grey | TMEM71 | grey |
| DNASE1L2 | blue | PIK3R5 | blue | CSRNP2 | cyan | VPS52 | cyan | BBOX1 | grey | IFT172 | grey | OR1Q1 | grey | TMEM72-AS1 | grey |
| DNLZ | blue | PIKFYVE | blue | CST3 | cyan | VPS54 | cyan | BBS10 | grey | IFT20 | grey | OR2A4 | grey | TMEM74B | grey |
| DNM2 | blue | PIN1P1 | blue | CTB-174D11.1 | cyan | VRK2 | cyan | BBS12 | grey | IFT46 | grey | OR2B6 | grey | TMEM75 | grey |
| DNMT3L | blue | PINX1 | blue | CTBP1 | cyan | VTI1B | cyan | BBS2 | grey | IFT88 | grey | OR2F2 | grey | TMEM86A | grey |
| DNPH1 | blue | PIP5KL1 | blue | CTBP2 | cyan | VWA3A | cyan | BBS4 | grey | IGBP1 | grey | OR2J2 | grey | TMEM88 | grey |
| DNTT | blue | PIPOX | blue | CTD-2537I9.16 | cyan | VWA5A | cyan | BBS5 | grey | IGDCC4 | grey | OR2S2 | grey | TMEM8A | grey |
| DOC2A | blue | PITPNM1 | blue | CTD-2540F13.2 | cyan | VWA8 | cyan | BBS7 | grey | IGFBP2 | grey | OR2W1 | grey | TMEM95 | grey |
| DOC2B | blue | PITX1 | blue | CTDSP1 | cyan | VWCE | cyan | BC010186 | grey | IGFBP7-AS1 | grey | OR4C1P | grey | TMEM97 | grey |
| DOCK6 | blue | PITX3 | blue | CTNNA1 | cyan | WAC | cyan | BC012193 | grey | IGFBPL1 | grey | OR51B4 | grey | TMEM98 | grey |
| DOCK9 | blue | PIWIL1 | blue | CTNNB1 | cyan | WARS2 | cyan | BC015159 | grey | IGFL1 | grey | OR51B5 | grey | TMIGD2 | grey |
| DOCK9-AS2 | blue | PKD2L1 | blue | CTSA | cyan | WASF1 | cyan | BC016361 | grey | IGFL2 | grey | OR51E1 | grey | TMPO-AS1 | grey |
| DOHH | blue | PKDREJ | blue | CTSB | cyan | WASF2 | cyan | BC021061 | grey | IGHV1-69 | grey | OR51I1 | grey | TMPRSS11E | grey |
| DOK1 | blue | PKN1 | blue | CTSO | cyan | WASL | cyan | BC024169 | grey | IGSF1 | grey | OR52A1 | grey | TMPRSS12 | grey |
| DOK2 | blue | PKNOX1 | blue | CTSS | cyan | WBP11 | cyan | BC028670 | grey | IGSF22 | grey | OR52D1 | grey | TMPRSS4 | grey |
| DOK4 | blue | PLA2G12B | blue | CTSV | cyan | WBP1L | cyan | BC032415 | grey | IGSF6 | grey | OR52K3P | grey | TMSB4Y | grey |
| DOK6 | blue | PLA2G15 | blue | CUL4B | cyan | WBP4 | cyan | BC033164 | grey | IGSF8 | grey | OR5E1P | grey | TMUB2 | grey |
| DOLPP1 | blue | PLA2G2E | blue | CUX1 | cyan | WDFY1 | cyan | BC034416 | grey | IL10 | grey | OR5L2 | grey | TNAP | grey |
| DPEP1 | blue | PLA2G4F | blue | CWC15 | cyan | WDFY3 | cyan | BC034444 | grey | IL10RA | grey | OR5P2 | grey | TNC | grey |
| DPEP3 | blue | PLA2G6 | blue | CWC25 | cyan | WDR1 | cyan | BC035400 | grey | IL10RB | grey | OR5P3 | grey | TNF | grey |
| DPF1 | blue | PLAC1 | blue | CWC27 | cyan | WDR11 | cyan | BC038205 | grey | IL11RA | grey | OR5V1 | grey | TNFAIP2 | grey |
| DPH5 | blue | PLB1 | blue | CXCR5 | cyan | WDR36 | cyan | BC039122 | grey | IL12A | grey | OR6B1 | grey | TNFAIP3 | grey |
| DPP10-AS1 | blue | PLCB3 | blue | CXCR6 | cyan | WDR48 | cyan | BC039319 | grey | IL12RB2 | grey | OR7A10 | grey | TNFAIP6 | grey |
| DPPA2 | blue | PLCH2 | blue | CXorf40B | cyan | WDR72 | cyan | BC039673 | grey | IL13RA2 | grey | OR7A5 | grey | TNFAIP8L1 | grey |
| DPY19L4 | blue | PLD2 | blue | CYB5D1 | cyan | WDR75 | cyan | BC040833 | grey | IL15 | grey | OR7C1 | grey | TNFAIP8L2 | grey |
| DPYD | blue | PLD4 | blue | CYFIP1 | cyan | WDR76 | cyan | BC041025 | grey | IL15RA | grey | OR7E14P | grey | TNFRSF10A | grey |
| DPYSL4 | blue | PLD6 | blue | CYP1A1 | cyan | WDR82 | cyan | BC042029 | grey | IL17D | grey | OR7E19P | grey | TNFRSF10B | grey |
| DQ588163 | blue | PLEKHA1 | blue | CYP2F1 | cyan | WIPF1 | cyan | BC042366 | grey | IL17RC | grey | OR7E24 | grey | TNFRSF10C | grey |
| DQ599616 | blue | PLEKHA3 | blue | CYP4F8 | cyan | WNT10A | cyan | BC042374 | grey | IL18R1 | grey | OR7E2P | grey | TNFRSF10D | grey |
| DR1 | blue | PLEKHA4 | blue | DAD1 | cyan | WNT6 | cyan | BC042590 | grey | IL18RAP | grey | OR7E37P | grey | TNFRSF11B | grey |
| DRD2 | blue | PLEKHF2 | blue | DAPK1 | cyan | WRB | cyan | BC043291 | grey | IL19 | grey | OR8B2 | grey | TNFRSF13C | grey |
| DRD3 | blue | PLEKHG3 | blue | DARS | cyan | WSB2 | cyan | BC043356 | grey | IL1B | grey | OR8G1 | grey | TNFRSF1A | grey |
| DRD4 | blue | PLEKHG4B | blue | DAZAP2 | cyan | WWC3 | cyan | BC044596 | grey | IL1R2 | grey | ORAI1 | grey | TNFRSF1B | grey |
| DRG2 | blue | PLEKHG5 | blue | DCAF12 | cyan | WWP1 | cyan | BC045560 | grey | IL1RAPL2 | grey | ORAI3 | grey | TNFSF13B | grey |
| DRICH1 | blue | PLEKHH3 | blue | DCAF16 | cyan | WWTR1 | cyan | BC045784 | grey | IL2 | grey | ORAOV1 | grey | TNFSF15 | grey |
| DSCC1 | blue | PLEKHN1 | blue | DCAF17 | cyan | XAF1 | cyan | BC045789 | grey | IL21 | grey | ORC1 | grey | TNFSF18 | grey |
| DSCR3 | blue | PLG | blue | DCP1B | cyan | XIAP | cyan | BC047364 | grey | IL21R | grey | ORC4 | grey | TNFSF8 | grey |
| DSCR4 | blue | PLIN5 | blue | DCTD | cyan | XPO1 | cyan | BC047626 | grey | IL22RA1 | grey | ORC5 | grey | TNFSF9 | grey |
| DSCR9 | blue | PLK1 | blue | DCTN6 | cyan | XPO4 | cyan | BC047644 | grey | IL23A | grey | ORC6 | grey | TNIP2 | grey |
| DTWD1 | blue | PLK3 | blue | DCUN1D4 | cyan | XXyac-YX155B6.7 | cyan | BC047651 | grey | IL23R | grey | ORMDL3 | grey | TNK2 | grey |
| DTX3 | blue | PLK5 | blue | DDHD2 | cyan | XYLT2 | cyan | BC048132 | grey | IL27RA | grey | OS9 | grey | TNK2-AS1 | grey |
| DTX3L | blue | PLSCR1 | blue | DDO | cyan | YAE1D1 | cyan | BC048141 | grey | IL2RA | grey | OSBPL11 | grey | TNKS1BP1 | grey |
| DUOXA1 | blue | PM20D2 | blue | DDOST | cyan | YAP1 | cyan | BC053951 | grey | IL2RB | grey | OSBPL3 | grey | TNMD | grey |
| DUOXA2 | blue | PMEL | blue | DDX17 | cyan | YIPF5 | cyan | BC062763 | grey | IL32 | grey | OSER1 | grey | TNN | grey |
| DUS3L | blue | PMFBP1 | blue | DDX24 | cyan | YPEL5 | cyan | BC069776 | grey | IL36B | grey | OSGEPL1 | grey | TNNC2 | grey |
| DUSP13 | blue | PMM1 | blue | DDX31 | cyan | YTHDF2 | cyan | BC070118 | grey | IL36G | grey | OSGIN2 | grey | TNNI3K | grey |
| DUSP15 | blue | PMPCB | blue | DDX3X | cyan | YTHDF3 | cyan | BC113958 | grey | IL37 | grey | OSR1 | grey | TNP2 | grey |
| DUSP2 | blue | PMVK | blue | DDX4 | cyan | YWHAB | cyan | BCAM | grey | IL4R | grey | OSTF1 | grey | TNR | grey |
| DUSP21 | blue | PNCK | blue | DDX5 | cyan | YWHAE | cyan | BCAP31 | grey | IL6 | grey | OTC | grey | TNS3 | grey |
| DUSP26 | blue | PNLIP | blue | DDX6 | cyan | YWHAG | cyan | BCAR3 | grey | IL6R | grey | OTOGL | grey | TOE1 | grey |
| DUSP27 | blue | PNLIPRP1 | blue | DDX60L | cyan | YWHAQ | cyan | BCAS3 | grey | IL7R | grey | OTX2-AS1 | grey | TOLLIP-AS1 | grey |
| DUSP6 | blue | PNMA3 | blue | DECR1 | cyan | YWHAZ | cyan | BCAT1 | grey | IL9 | grey | OVGP1 | grey | TOMM34 | grey |
| DUSP9 | blue | PNMA6A | blue | DEDD | cyan | YY1 | cyan | BCDIN3D | grey | ILDR2 | grey | OXCT1 | grey | TOPORS-AS1 | grey |
| DUX1 | blue | PNMT | blue | DEDD2 | cyan | ZADH2 | cyan | BCHE | grey | ILF2 | grey | OXER1 | grey | TOR1B | grey |
| DVL2 | blue | PNN | blue | DEFB119 | cyan | ZBTB18 | cyan | BCKDHA | grey | ILF3-AS1 | grey | OXGR1 | grey | TOR4A | grey |
| DYNC2LI1 | blue | PNPLA3 | blue | DEGS1 | cyan | ZBTB20 | cyan | BCKDK | grey | IMPA2 | grey | OXNAD1 | grey | TOX3 | grey |
| DYRK1B | blue | PNPLA5 | blue | DEK | cyan | ZBTB33 | cyan | BCL11B | grey | IMPDH1 | grey | OXSM | grey | TP53 | grey |
| DZIP1L | blue | PNPLA7 | blue | DENND4C | cyan | ZBTB38 | cyan | BCL2 | grey | INA | grey | OXTR | grey | TP53BP2 | grey |
| E2F4 | blue | PNPT1 | blue | DENND5A | cyan | ZBTB4 | cyan | BCL2A1 | grey | INCENP | grey | P2RX1 | grey | TP53I3 | grey |
| E4F1 | blue | POC1A | blue | DENR | cyan | ZBTB44 | cyan | BCL2L12 | grey | INE1 | grey | P2RX4 | grey | TP53INP2 | grey |
| EBF4 | blue | PODXL | blue | DERL2 | cyan | ZBTB8OS | cyan | BCL2L2 | grey | INHBB | grey | P2RX5 | grey | TP53TG1 | grey |
| EBI3 | blue | PODXL2 | blue | DFFA | cyan | ZC2HC1A | cyan | BCL7A | grey | INHBC | grey | P2RY1 | grey | TPBG | grey |
| ECD | blue | POGLUT1 | blue | DGCR9 | cyan | ZC3H13 | cyan | BCL7B | grey | INHBE | grey | P2RY10 | grey | TPBGL | grey |
| ECE2 | blue | POLK | blue | DHRS2 | cyan | ZC3H7A | cyan | BCL7C | grey | INMT | grey | P2RY13 | grey | TPCN1 | grey |
| ECEL1 | blue | POLL | blue | DHRS3 | cyan | ZC3HAV1 | cyan | BCL9 | grey | INO80E | grey | P2RY14 | grey | TPD52L2 | grey |
| ECRP | blue | POLN | blue | DHX15 | cyan | ZCCHC13 | cyan | BCL9L | grey | INPP1 | grey | P2RY4 | grey | TPM2 | grey |
| EDC4 | blue | POLR1B | blue | DHX29 | cyan | ZCCHC2 | cyan | BCO2 | grey | INPP4B | grey | P2RY6 | grey | TPPP3 | grey |
| EDN2 | blue | POLR1C | blue | DHX36 | cyan | ZCCHC6 | cyan | BCORP1 | grey | INPP5A | grey | P2RY8 | grey | TPRG1L | grey |
| EEF1A2 | blue | POLR2B | blue | DHX40 | cyan | ZCCHC8 | cyan | BCS1L | grey | INPP5B | grey | P4HA1 | grey | TPRXL | grey |
| EEFSEC | blue | POLR2J2 | blue | DIABLO | cyan | ZCRB1 | cyan | BD495725 | grey | INPP5E | grey | PAAF1 | grey | TPSAB1 | grey |
| EFNB1 | blue | POLR3D | blue | DIAPH2 | cyan | ZFAND1 | cyan | BDH2 | grey | INSC | grey | PABPC4L | grey | TPSB2 | grey |
| EGFL7 | blue | POLRMT | blue | DICER1 | cyan | ZFAS1 | cyan | BDKRB2 | grey | INSL4 | grey | PABPN1 | grey | TPST1 | grey |
| EGFR | blue | POM121L10P | blue | DIMT1 | cyan | ZFP28 | cyan | BDP1 | grey | INTS2 | grey | PACS1 | grey | TPST2 | grey |
| EGLN2 | blue | POMC | blue | DIO1 | cyan | ZFP90 | cyan | BEND3 | grey | INTS5 | grey | PADI3 | grey | TPTE2P5 | grey |
| EGOT | blue | POMT2 | blue | DIRC2 | cyan | ZFP91 | cyan | BEND5 | grey | INTU | grey | PADI4 | grey | TPTEP1 | grey |
| EGR4 | blue | PORCN | blue | DKFZP434K028 | cyan | ZFR | cyan | BEST2 | grey | INVS | grey | PAGE4 | grey | TPX2 | grey |
| EHMT1 | blue | POU2F2 | blue | DLAT | cyan | ZFYVE21 | cyan | BEX1 | grey | IPCEF1 | grey | PAK3 | grey | TRABD2B | grey |
| EHMT2 | blue | POU3F2 | blue | DLD | cyan | ZFYVE26 | cyan | BEX5 | grey | IPO11 | grey | PALM2 | grey | TRAC | grey |
| EID2 | blue | POU3F4 | blue | DLX2 | cyan | ZHX1 | cyan | BFAR | grey | IPO4 | grey | PALMD | grey | TRAF3IP1 | grey |
| EIF2B1 | blue | POU4F3 | blue | DMD | cyan | ZHX2 | cyan | BFSP1 | grey | IPO9-AS1 | grey | PAM16 | grey | TRAIP | grey |
| EIF2S1 | blue | POU5F1B | blue | DMRT3 | cyan | ZIC4 | cyan | BFSP2 | grey | IQCF4 | grey | PAMR1 | grey | TRAM1L1 | grey |
| EIF3A | blue | POU5F1P3 | blue | DMTF1 | cyan | ZKSCAN1 | cyan | BGLT3 | grey | IQCF5-AS1 | grey | PAN3-AS1 | grey | TRAM2-AS1 | grey |
| EIF3E | blue | POU5F1P4 | blue | DNAH10 | cyan | ZKSCAN8 | cyan | BGN | grey | IQGAP2 | grey | PANK1 | grey | TRANK1 | grey |
| EIF3M | blue | POU5F2 | blue | DNAJA1 | cyan | ZMAT2 | cyan | BHLHB9 | grey | IRAK1 | grey | PANX3 | grey | TRAPPC2L | grey |
| EIF4E | blue | POU6F1 | blue | DNAJA2 | cyan | ZMAT3 | cyan | BHLHE40 | grey | IRAK1BP1 | grey | PAPD7 | grey | TRAPPC3L | grey |
| ELANE | blue | POU6F2 | blue | DNAJB11 | cyan | ZMPSTE24 | cyan | BHLHE41 | grey | IRF1 | grey | PARD6G-AS1 | grey | TRAPPC5 | grey |
| ELAVL3 | blue | PPCDC | blue | DNAJB13 | cyan | ZMYM4 | cyan | BHMT | grey | IRF3 | grey | PARG | grey | TRAPPC6A | grey |
| ELF2 | blue | PPFIA3 | blue | DNAJB14 | cyan | ZNF106 | cyan | BID | grey | IRF4 | grey | PARM1 | grey | TRAPPC6B | grey |
| ELFN1 | blue | PPFIA4 | blue | DNAJB4 | cyan | ZNF12 | cyan | BIK | grey | IRF7 | grey | PARP12 | grey | TRAT1 | grey |
| ELFN1-AS1 | blue | PPFIBP1 | blue | DNAJB6 | cyan | ZNF121 | cyan | BIN1 | grey | IRF8 | grey | PARP14 | grey | TRAV12-2 | grey |
| ELFN2 | blue | PPIE | blue | DNAJC10 | cyan | ZNF148 | cyan | BIN2 | grey | IRG1 | grey | PARP15 | grey | TRAV8-3 | grey |
| ELMO2 | blue | PPIEL | blue | DNAJC14 | cyan | ZNF189 | cyan | BIN3 | grey | IRGM | grey | PARP16 | grey | TRBC1 | grey |
| ELP6 | blue | PPIL2 | blue | DNAJC15 | cyan | ZNF205-AS1 | cyan | BIRC2 | grey | IRS1 | grey | PARP9 | grey | TRDV3 | grey |
| EMC7 | blue | PPIL6 | blue | DNAJC22 | cyan | ZNF207 | cyan | BIRC3 | grey | IRX1 | grey | PARS2 | grey | TREM1 | grey |
| EMD | blue | PPM1A | blue | DNAJC7 | cyan | ZNF22 | cyan | BLACAT1 | grey | IRX2 | grey | PASK | grey | TREML1 | grey |
| EMID1 | blue | PPM1B | blue | DNAJC8 | cyan | ZNF229 | cyan | BLID | grey | IRX5 | grey | PATE2 | grey | TREML2 | grey |
| EMILIN1 | blue | PPM1J | blue | DNM1L | cyan | ZNF24 | cyan | BLMH | grey | ISCA2 | grey | PATL2 | grey | TREML3P | grey |
| EML2 | blue | PPM1N | blue | DNM1P35 | cyan | ZNF260 | cyan | BLNK | grey | ISL1 | grey | PAWR | grey | TRG-AS1 | grey |
| EML4 | blue | PPOX | blue | DNMBP | cyan | ZNF275 | cyan | BLOC1S1 | grey | ISL2 | grey | PAX5 | grey | TRH | grey |
| EMX1 | blue | PPP1R12C | blue | DNTTIP2 | cyan | ZNF281 | cyan | BLOC1S4 | grey | ISM1 | grey | PAX8-AS1 | grey | TRHDE | grey |
| ENAH | blue | PPP1R15B | blue | DPH3 | cyan | ZNF337 | cyan | BLVRB | grey | ISM2 | grey | PAXIP1 | grey | TRIAP1 | grey |
| ENDOG | blue | PPP1R21 | blue | DPM1 | cyan | ZNF33A | cyan | BMF | grey | ISOC2 | grey | PAXIP1-AS1 | grey | TRIB1 | grey |
| ENDOV | blue | PPP1R26-AS1 | blue | DPP8 | cyan | ZNF383 | cyan | BMP10 | grey | ISX | grey | PAXIP1OS | grey | TRIL | grey |
| ENHO | blue | PPP1R2P9 | blue | DPY19L3 | cyan | ZNF43 | cyan | BMP2 | grey | ITFG2 | grey | PBDC1 | grey | TRIM14 | grey |
| ENKD1 | blue | PPP1R32 | blue | DPY30 | cyan | ZNF445 | cyan | BMP3 | grey | ITFG3 | grey | PBOV1 | grey | TRIM16 | grey |
| ENO1-AS1 | blue | PPP1R37 | blue | DPYSL5 | cyan | ZNF460 | cyan | BMP4 | grey | ITGA10 | grey | PBX4 | grey | TRIM24 | grey |
| ENOX2 | blue | PPP1R3F | blue | DQ580846 | cyan | ZNF512 | cyan | BMP6 | grey | ITGA11 | grey | PC | grey | TRIM26 | grey |
| ENPP7 | blue | PPP2CA | blue | DQX1 | cyan | ZNF559 | cyan | BMPER | grey | ITGA4 | grey | PCAT18 | grey | TRIM28 | grey |
| ENTHD2 | blue | PPP2R2C | blue | DRAM2 | cyan | ZNF584 | cyan | BMPR1B | grey | ITGA8 | grey | PCAT19 | grey | TRIM32 | grey |
| ENTPD2 | blue | PPP2R4 | blue | DRG1 | cyan | ZNF587B | cyan | BMS1 | grey | ITGA9-AS1 | grey | PCAT6 | grey | TRIM35 | grey |
| EP300 | blue | PPP2R5B | blue | DROSHA | cyan | ZNF600 | cyan | BOC | grey | ITGAD | grey | PCBP4 | grey | TRIM37 | grey |
| EPHA10 | blue | PPP6R2 | blue | DSG1 | cyan | ZNF615 | cyan | BOD1 | grey | ITGAE | grey | PCDH12 | grey | TRIM40 | grey |
| EPHA5-AS1 | blue | PPY | blue | DST | cyan | ZNF621 | cyan | BORA | grey | ITGAL | grey | PCDH17 | grey | TRIM47 | grey |
| EPHB4 | blue | PPY2 | blue | DSTN | cyan | ZNF623 | cyan | BPHL | grey | ITGAX | grey | PCDH19 | grey | TRIM51 | grey |
| EPHX2 | blue | PQBP1 | blue | DSTNP2 | cyan | ZNF639 | cyan | BPIFA1 | grey | ITGB1BP2 | grey | PCDHA2 | grey | TRIM52-AS1 | grey |
| EPHX3 | blue | PQLC1 | blue | DTD1 | cyan | ZNF641 | cyan | BPIFA2 | grey | ITGB2 | grey | PCDHAC2 | grey | TRIM56 | grey |
| EPN1 | blue | PQLC2 | blue | DUS2 | cyan | ZNF655 | cyan | BPIFA3 | grey | ITGB4 | grey | PCDHB1 | grey | TRIM59 | grey |
| EPN3 | blue | PRAC1 | blue | DUSP22 | cyan | ZNF667-AS1 | cyan | BPIFA4P | grey | ITIH3 | grey | PCDHB11 | grey | TRIM6 | grey |
| EPO | blue | PRAM1 | blue | DUSP3 | cyan | ZNF675 | cyan | BPIFB2 | grey | ITK | grey | PCDHB12 | grey | TRIM67 | grey |
| EPX | blue | PRAME | blue | DUT | cyan | ZNF706 | cyan | BPY2 | grey | ITPA | grey | PCDHB14 | grey | TRIM68 | grey |
| ERCC2 | blue | PRAP1 | blue | DVL3 | cyan | ZNF83 | cyan | BRAT1 | grey | ITPKC | grey | PCDHB15 | grey | TRIM78P | grey |
| ERCC3 | blue | PRDM11 | blue | DYNC1I2 | cyan | ZNF843 | cyan | BRCA2 | grey | ITPR1 | grey | PCDHB16 | grey | TRIP13 | grey |
| ERCC5 | blue | PRDM14 | blue | DYNC1LI1 | cyan | ZNF852 | cyan | BRD9 | grey | ITPR1-AS1 | grey | PCDHB4 | grey | TRIP4 | grey |
| ERCC6 | blue | PRDM8 | blue | DYNLL1 | cyan | ZRANB1 | cyan | BRE-AS1 | grey | IVL | grey | PCDHB5 | grey | TRIP6 | grey |
| ERF | blue | PRDX3 | blue | DYNLL2 | cyan | ZRANB2 | cyan | BRF2 | grey | IZUMO2 | grey | PCDHB9 | grey | TRIT1 | grey |
| ERI1 | blue | PRG2 | blue | DYNLT3 | cyan | ZSWIM6 | cyan | BRI3 | grey | Igk | grey | PCDHGA1 | grey | TRMT10C | grey |
| ERICH1-AS1 | blue | PRG3 | blue | DYRK1A | cyan | ZYG11B | cyan | BRI3BP | grey | JADE1 | grey | PCDHGA10 | grey | TRNP1 | grey |
| ERICH3-AS1 | blue | PRICKLE3 | blue | EAPP | cyan | ABHD17C | darkgrey | BRINP1 | grey | JADE3 | grey | PCDHGA4 | grey | TRPC5OS | grey |
| ERMP1 | blue | PRKACG | blue | EBAG9 | cyan | ABHD6 | darkgrey | BRINP2 | grey | JAK3 | grey | PCDHGA8 | grey | TRPV2 | grey |
| ERN2 | blue | PRKAG2-AS1 | blue | EBLN3 | cyan | ABLIM3 | darkgrey | BRINP3 | grey | JAKMIP2-AS1 | grey | PCDHGA9 | grey | TRY2P | grey |
| ERVH-6 | blue | PRKCE | blue | ECHDC1 | cyan | ACAA2 | darkgrey | BRK1 | grey | JAKMIP3 | grey | PCDHGB5 | grey | TSC22D2 | grey |
| ERVH48-1 | blue | PRKCG | blue | ECHS1 | cyan | ACKR1 | darkgrey | BRMS1 | grey | JHDM1D-AS1 | grey | PCDHGB6 | grey | TSC22D3 | grey |
| ERVK3-2 | blue | PRKD2 | blue | ECI2 | cyan | ACKR3 | darkgrey | BRPF1 | grey | JMJD1C-AS1 | grey | PCDHGB7 | grey | TSEN2 | grey |
| ESPN | blue | PRKRA | blue | ECSIT | cyan | ACSF2 | darkgrey | BRWD1-IT2 | grey | JOSD1 | grey | PCDHGB8P | grey | TSEN34 | grey |
| ESPNL | blue | PRLH | blue | EDEM3 | cyan | ACSM3 | darkgrey | BSDC1 | grey | JOSD2 | grey | PCDHGC3 | grey | TSHB | grey |
| ESR2 | blue | PRM2 | blue | EEA1 | cyan | ADCY3 | darkgrey | BSG | grey | JPX | grey | PCED1B | grey | TSHZ2 | grey |
| ESRRB | blue | PRM3 | blue | EEF1E1 | cyan | ADCY4 | darkgrey | BSN | grey | JUN | grey | PCED1B-AS1 | grey | TSIX | grey |
| ESYT2 | blue | PRMT3 | blue | EEF2 | cyan | ADM | darkgrey | BST2 | grey | JUNB | grey | PCGF1 | grey | TSPAN1 | grey |
| ETF1 | blue | PRMT7 | blue | EFCAB14 | cyan | AFAP1L1 | darkgrey | BTBD10 | grey | JUND | grey | PCGF2 | grey | TSPAN11 | grey |
| ETNK2 | blue | PRMT8 | blue | EFHC1 | cyan | AFTPH | darkgrey | BTBD18 | grey | KAL1 | grey | PCGF6 | grey | TSPAN14 | grey |
| ETV2 | blue | PRNT | blue | EFNA2 | cyan | AGR2 | darkgrey | BTBD19 | grey | KANK4 | grey | PCIF1 | grey | TSPAN15 | grey |
| ETV4 | blue | PRO2949 | blue | EFR3A | cyan | AHNAK | darkgrey | BTBD2 | grey | KANSL1-AS1 | grey | PCK2 | grey | TSPAN2 | grey |
| ETV7 | blue | PROC | blue | EFR3B | cyan | AHNAK2 | darkgrey | BTBD3 | grey | KAT2B | grey | PCMTD2 | grey | TSPAN8 | grey |
| EVA1B | blue | PROCA1 | blue | EGLN1 | cyan | AIM2 | darkgrey | BTBD6 | grey | KAT5 | grey | PCNXL3 | grey | TSPO | grey |
| EVI5L | blue | PROP1 | blue | EHBP1 | cyan | AKR1A1 | darkgrey | BTC | grey | KAT8 | grey | PCOLCE-AS1 | grey | TSPO2 | grey |
| EVPLL | blue | PRORY | blue | EID1 | cyan | ALDH18A1 | darkgrey | BTF3P11 | grey | KATNA1 | grey | PCP4 | grey | TSPY1 | grey |
| EVX1 | blue | PROSER3 | blue | EIF1 | cyan | ALDH1A3 | darkgrey | BTG2 | grey | KB-1000E4.2 | grey | PCP4L1 | grey | TSPYL2 | grey |
| EXD2 | blue | PROZ | blue | EIF1AX | cyan | ALDH1L2 | darkgrey | BTK | grey | KB-1836B5.1 | grey | PCTP | grey | TSPYL6 | grey |
| EXD3 | blue | PRPF31 | blue | EIF1B | cyan | ALG14 | darkgrey | BTLA | grey | KB-431C1.5 | grey | PCYOX1L | grey | TSSC4 | grey |
| EXOC3 | blue | PRPF38B | blue | EIF2A | cyan | ALG2 | darkgrey | BTN2A3P | grey | KBTBD11 | grey | PDCD1LG2 | grey | TST | grey |
| EXOC3L4 | blue | PRPF4 | blue | EIF2AK1 | cyan | ALPL | darkgrey | BTN3A1 | grey | KBTBD3 | grey | PDCD2L | grey | TSTD2 | grey |
| EXOC5 | blue | PRPF40A | blue | EIF2AK2 | cyan | AMPD1 | darkgrey | BTN3A2 | grey | KCNA1 | grey | PDCD4 | grey | TTC1 | grey |
| EXOSC6 | blue | PRPF4B | blue | EIF2D | cyan | ANGPTL1 | darkgrey | BTNL3 | grey | KCNA2 | grey | PDCD4-AS1 | grey | TTC13 | grey |
| EXTL1 | blue | PRR15 | blue | EIF2S2 | cyan | ANK3 | darkgrey | BTNL9 | grey | KCNA4 | grey | PDDC1 | grey | TTC27 | grey |
| EXTL3 | blue | PRR18 | blue | EIF2S3 | cyan | ANO1 | darkgrey | BUB1B | grey | KCNA5 | grey | PDE10A | grey | TTC29 | grey |
| F10 | blue | PRR19 | blue | EIF3H | cyan | ANXA3 | darkgrey | BUD13 | grey | KCNA6 | grey | PDE2A | grey | TTC31 | grey |
| F11-AS1 | blue | PRR22 | blue | EIF3J | cyan | AP3M1 | darkgrey | BVES | grey | KCNAB1 | grey | PDE3A | grey | TTC32 | grey |
| F12 | blue | PRR29 | blue | EIF3K | cyan | APLNR | darkgrey | BVES-AS1 | grey | KCNB1 | grey | PDE6A | grey | TTC39C | grey |
| F2 | blue | PRR34 | blue | EIF3L | cyan | APOO | darkgrey | BYSL | grey | KCNB2 | grey | PDE6G | grey | TTC4 | grey |
| FAAH | blue | PRR7 | blue | EIF4B | cyan | AQP3 | darkgrey | BZRAP1 | grey | KCND3 | grey | PDE6H | grey | TTC8 | grey |
| FABP1 | blue | PRRC2A | blue | EIF4E3 | cyan | AQP5 | darkgrey | BZRAP1-AS1 | grey | KCNE1L | grey | PDE8B | grey | TTC9 | grey |
| FABP2 | blue | PRRG2 | blue | EIF4G1 | cyan | ARFGAP3 | darkgrey | BZW2 | grey | KCNE2 | grey | PDGFA | grey | TTI2 | grey |
| FADS2 | blue | PRRT2 | blue | EIF4G2 | cyan | ARFIP2 | darkgrey | C10orf10 | grey | KCNE4 | grey | PDGFRA | grey | TTL | grey |
| FAHD2CP | blue | PRRX2-AS1 | blue | EIF4H | cyan | ARHGAP18 | darkgrey | C10orf107 | grey | KCNG2 | grey | PDHA2 | grey | TTLL1 | grey |
| FAM102A | blue | PRSS2 | blue | EIF5 | cyan | ARHGAP5 | darkgrey | C10orf11 | grey | KCNG4 | grey | PDHX | grey | TTLL11-IT1 | grey |
| FAM109A | blue | PRSS27 | blue | EIF5B | cyan | ARHGAP5-AS1 | darkgrey | C10orf113 | grey | KCNIP1 | grey | PDIK1L | grey | TTLL6 | grey |
| FAM110D | blue | PRSS3 | blue | ELF1 | cyan | ARHGEF15 | darkgrey | C10orf25 | grey | KCNIP2-AS1 | grey | PDK2 | grey | TTLL7-IT1 | grey |
| FAM114A2 | blue | PRSS30P | blue | ELMO1 | cyan | ARHGEF28 | darkgrey | C10orf32 | grey | KCNJ11 | grey | PDLIM7 | grey | TTN-AS1 | grey |
| FAM118B | blue | PRSS3P3 | blue | ELMO3 | cyan | ATF4 | darkgrey | C10orf40 | grey | KCNJ14 | grey | PDPK1 | grey | TTPA | grey |
| FAM122A | blue | PRSS50 | blue | ELMSAN1 | cyan | ATG13 | darkgrey | C10orf54 | grey | KCNJ2 | grey | PDRG1 | grey | TTTY10 | grey |
| FAM122B | blue | PRSS54 | blue | EMC2 | cyan | ATG4A | darkgrey | C10orf71-AS1 | grey | KCNJ5 | grey | PDSS2 | grey | TTTY11 | grey |
| FAM126B | blue | PRSS58 | blue | EMC3 | cyan | ATL2 | darkgrey | C10orf76 | grey | KCNJ8 | grey | PDZD2 | grey | TTTY12 | grey |
| FAM129B | blue | PRTN3 | blue | EMC4 | cyan | ATP1A1 | darkgrey | C10orf82 | grey | KCNK13 | grey | PDZRN3 | grey | TTTY13 | grey |
| FAM131A | blue | PRX | blue | EML3 | cyan | ATP1B1 | darkgrey | C10orf85 | grey | KCNK17 | grey | PDZRN3-AS1 | grey | TTTY15 | grey |
| FAM131B | blue | PSD | blue | ENO3 | cyan | ATP2A3 | darkgrey | C10orf95 | grey | KCNK2 | grey | PDZRN4 | grey | TTTY5 | grey |
| FAM131C | blue | PSG5 | blue | ENOPH1 | cyan | ATP6AP1 | darkgrey | C11orf1 | grey | KCNK3 | grey | PEAR1 | grey | TTYH1 | grey |
| FAM132A | blue | PSKH1 | blue | ENOSF1 | cyan | ATP6V0A4 | darkgrey | C11orf24 | grey | KCNMA1 | grey | PEF1 | grey | TTYH2 | grey |
| FAM151A | blue | PSMA2 | blue | ENSA | cyan | AZGP1 | darkgrey | C11orf40 | grey | KCNMB1 | grey | PEG3 | grey | TTYH3 | grey |
| FAM153A | blue | PSMC4 | blue | ENY2 | cyan | B3GNT7 | darkgrey | C11orf44 | grey | KCNMB3 | grey | PELP1 | grey | TUBA1B | grey |
| FAM166A | blue | PSMD12 | blue | EPB41L2 | cyan | BARX2 | darkgrey | C11orf65 | grey | KCNN2 | grey | PEMT | grey | TUBA1C | grey |
| FAM167B | blue | PSMD13 | blue | EPC1 | cyan | BCKDHB | darkgrey | C11orf68 | grey | KCNQ1-AS1 | grey | PENK | grey | TUBA3FP | grey |
| FAM170A | blue | PSPN | blue | EPHA1-AS1 | cyan | BCL11A | darkgrey | C11orf72 | grey | KCNQ3 | grey | PEPD | grey | TUBA4A | grey |
| FAM170B | blue | PSTK | blue | EPM2AIP1 | cyan | BEX2 | darkgrey | C11orf74 | grey | KCNQ5 | grey | PER3 | grey | TUBAL3 | grey |
| FAM171A2 | blue | PTAR1 | blue | EPS15 | cyan | BLM | darkgrey | C11orf83 | grey | KCNRG | grey | PET112 | grey | TUBB1 | grey |
| FAM173A | blue | PTBP2 | blue | EPS8L1 | cyan | BMP1 | darkgrey | C11orf84 | grey | KCNS3 | grey | PET117 | grey | TUBB2B | grey |
| FAM175B | blue | PTCHD2 | blue | EPS8L3 | cyan | BMS1P20 | darkgrey | C11orf92 | grey | KCNT2 | grey | PEX11A | grey | TUBB3 | grey |
| FAM178B | blue | PTCRA | blue | ERAP1 | cyan | BOLA3 | darkgrey | C11orf95 | grey | KCTD18 | grey | PEX12 | grey | TUBB4A | grey |
| FAM179A | blue | PTDSS2 | blue | ERBB2IP | cyan | BSPRY | darkgrey | C11orf96 | grey | KCTD21 | grey | PEX6 | grey | TUBD1 | grey |
| FAM181A | blue | PTGDR2 | blue | ERCC1 | cyan | BST1 | darkgrey | C12orf10 | grey | KCTD9 | grey | PF4 | grey | TUBE1 | grey |
| FAM181A-AS1 | blue | PTGER1 | blue | ERGIC1 | cyan | C14orf1 | darkgrey | C12orf43 | grey | KDELC1 | grey | PFDN1 | grey | TUBG1 | grey |
| FAM181B | blue | PTGER4P2-CDK2AP2P2 | blue | ERGIC2 | cyan | C15orf65 | darkgrey | C12orf45 | grey | KDM4A | grey | PFKM | grey | TUBG2 | grey |
| FAM183CP | blue | PTGES2 | blue | ERH | cyan | C1orf116 | darkgrey | C12orf5 | grey | KDM5D | grey | PGAM1 | grey | TUSC8 | grey |
| FAM187B | blue | PTGIR | blue | ERLEC1 | cyan | C1orf210 | darkgrey | C12orf55 | grey | KDM7A | grey | PGAP1 | grey | TVP23C | grey |
| FAM188A | blue | PTH2 | blue | ERLIN1 | cyan | C20orf194 | darkgrey | C12orf57 | grey | KEAP1 | grey | PGBD1 | grey | TWIST1 | grey |
| FAM204A | blue | PTK2B | blue | ERLIN2 | cyan | C4orf19 | darkgrey | C12orf61 | grey | KEL | grey | PGBD4 | grey | TWISTNB | grey |
| FAM205A | blue | PTOV1-AS1 | blue | ESCO1 | cyan | C5orf46 | darkgrey | C12orf75 | grey | KERA | grey | PGBD5 | grey | TXLNGY | grey |
| FAM207A | blue | PTP4A3 | blue | ESD | cyan | C6orf136 | darkgrey | C12orf79 | grey | KGFLP2 | grey | PGF | grey | TXN2 | grey |
| FAM20C | blue | PTPN5 | blue | ESF1 | cyan | C8orf4 | darkgrey | C14orf142 | grey | KHDC1 | grey | PGLYRP4 | grey | TXNDC16 | grey |
| FAM214B | blue | PTPRH | blue | ETFA | cyan | C8orf59 | darkgrey | C14orf169 | grey | KHDC1L | grey | PGM2 | grey | TXNDC17 | grey |
| FAM215A | blue | PTRH1 | blue | ETS1 | cyan | C9orf152 | darkgrey | C14orf183 | grey | KHDRBS2 | grey | PGM5 | grey | TXNDC8 | grey |
| FAM217B | blue | PURB | blue | ETV3 | cyan | CA2 | darkgrey | C14orf37 | grey | KHDRBS3 | grey | PHACTR1 | grey | TYK2 | grey |
| FAM220A | blue | PVR | blue | EVI2B | cyan | CACHD1 | darkgrey | C14orf39 | grey | KIAA0141 | grey | PHACTR3 | grey | TYMS | grey |
| FAM221B | blue | PVRL1 | blue | EWSR1 | cyan | CADM1 | darkgrey | C14orf64 | grey | KIAA0226L | grey | PHEX | grey | TYR | grey |
| FAM222A-AS1 | blue | PWAR5 | blue | EXOC1 | cyan | CADM3 | darkgrey | C14orf93 | grey | KIAA0232 | grey | PHEX-AS1 | grey | TYRO3P | grey |
| FAM223B | blue | PWP2 | blue | EXOC8 | cyan | CADPS2 | darkgrey | C15orf27 | grey | KIAA0247 | grey | PHF1 | grey | TYRP1 | grey |
| FAM229A | blue | PWWP2B | blue | EXOSC8 | cyan | CAMSAP3 | darkgrey | C15orf37 | grey | KIAA0319L | grey | PHF13 | grey | TYW1 | grey |
| FAM24B | blue | PXDC1 | blue | FABP6 | cyan | CANT1 | darkgrey | C15orf39 | grey | KIAA0355 | grey | PHF14 | grey | U2AF1L4 | grey |
| FAM3A | blue | PYDC1 | blue | FAF2 | cyan | CBR3-AS1 | darkgrey | C15orf48 | grey | KIAA0408 | grey | PHF2 | grey | U47924.29 | grey |
| FAM53A | blue | PYGO2 | blue | FAM102B | cyan | CBX4 | darkgrey | C15orf49 | grey | KIAA0485 | grey | PHF23 | grey | U91328.20 | grey |
| FAM64A | blue | PYROXD1 | blue | FAM103A1 | cyan | CCDC117 | darkgrey | C15orf52 | grey | KIAA0509 | grey | PHF2P1 | grey | UAP1L1 | grey |
| FAM65C | blue | PYY | blue | FAM104A | cyan | CCDC160 | darkgrey | C15orf54 | grey | KIAA0556 | grey | PHF8 | grey | UBAC1 | grey |
| FAM69B | blue | PYY2 | blue | FAM107B | cyan | CCDC6 | darkgrey | C15orf56 | grey | KIAA0753 | grey | PHGDH | grey | UBAP2 | grey |
| FAM69C | blue | QPCTL | blue | FAM111A | cyan | CCL28 | darkgrey | C16orf54 | grey | KIAA0754 | grey | PHLDB1 | grey | UBC | grey |
| FAM71A | blue | QRICH2 | blue | FAM114A1 | cyan | CCL5 | darkgrey | C16orf80 | grey | KIAA0895 | grey | PHOSPHO1 | grey | UBE2C | grey |
| FAM71B | blue | QSOX2 | blue | FAM117A | cyan | CCNC | darkgrey | C16orf89 | grey | KIAA0895L | grey | PHOSPHO2 | grey | UBE2DNL | grey |
| FAM71E1 | blue | R3HCC1L | blue | FAM120A | cyan | CCR2 | darkgrey | C16orf91 | grey | KIAA0930 | grey | PHOX2B | grey | UBE2E2-AS1 | grey |
| FAM71E2 | blue | R3HDM1 | blue | FAM126A | cyan | CCSAP | darkgrey | C16orf92 | grey | KIAA1107 | grey | PHPT1 | grey | UBE2L6 | grey |
| FAM71F1 | blue | R3HDML | blue | FAM129A | cyan | CD2 | darkgrey | C16orf93 | grey | KIAA1161 | grey | PHYHD1 | grey | UBE2M | grey |
| FAM73A | blue | RAB11A | blue | FAM134A | cyan | CD24 | darkgrey | C17orf105 | grey | KIAA1210 | grey | PHYHIPL | grey | UBE2NL | grey |
| FAM73B | blue | RAB21 | blue | FAM160B2 | cyan | CD248 | darkgrey | C17orf58 | grey | KIAA1324L | grey | PHYKPL | grey | UBE2QL1 | grey |
| FAM76B | blue | RAB27A | blue | FAM162A | cyan | CD27 | darkgrey | C17orf62 | grey | KIAA1407 | grey | PI4K2A | grey | UBE2S | grey |
| FAM83C | blue | RAB30-AS1 | blue | FAM163A | cyan | CD44 | darkgrey | C17orf64 | grey | KIAA1586 | grey | PI4KA | grey | UBE2T | grey |
| FAM83E | blue | RAB33B | blue | FAM168A | cyan | CD52 | darkgrey | C17orf75 | grey | KIAA1614-AS1 | grey | PIAS3 | grey | UBE2Z | grey |
| FAM83G | blue | RAB3GAP1 | blue | FAM168B | cyan | CDC42EP2 | darkgrey | C17orf80 | grey | KIAA1644 | grey | PIFO | grey | UBL4A | grey |
| FAM86B3P | blue | RAB40A | blue | FAM171A1 | cyan | CDH1 | darkgrey | C17orf97 | grey | KIAA1661 | grey | PIGA | grey | UBL4B | grey |
| FAM91A1 | blue | RAB40AL | blue | FAM172A | cyan | CDK2AP2 | darkgrey | C17orf99 | grey | KIAA1755 | grey | PIGB | grey | UBL5 | grey |
| FAM98A | blue | RAB42 | blue | FAM174A | cyan | CDKN1C | darkgrey | C18orf12 | grey | KIAA1958 | grey | PIGH | grey | UBQLNL | grey |
| FAM99B | blue | RABEP2 | blue | FAM179B | cyan | CDS1 | darkgrey | C18orf15 | grey | KIAA2013 | grey | PIGM | grey | UBXN10-AS1 | grey |
| FANCA | blue | RABGAP1L | blue | FAM192A | cyan | CEACAM1 | darkgrey | C19orf12 | grey | KIF12 | grey | PIGN | grey | UBXN11 | grey |
| FANCE | blue | RAC3 | blue | FAM199X | cyan | CEACAM6 | darkgrey | C19orf24 | grey | KIF13B | grey | PIGT | grey | UBXN7-AS1 | grey |
| FANCL | blue | RAD23B | blue | FAM200B | cyan | CELSR1 | darkgrey | C19orf33 | grey | KIF15 | grey | PIGU | grey | UBXN8 | grey |
| FARSA | blue | RAD51D | blue | FAM210B | cyan | CFD | darkgrey | C19orf40 | grey | KIF16B | grey | PIGZ | grey | UCA1 | grey |
| FARSB | blue | RAD54L | blue | FAM213A | cyan | CHCHD7 | darkgrey | C19orf52 | grey | KIF18A | grey | PIH1D1 | grey | UCP2 | grey |
| FAS | blue | RAE1 | blue | FAM213B | cyan | CHI3L2 | darkgrey | C19orf54 | grey | KIF20A | grey | PIH1D3 | grey | UFSP2 | grey |
| FAS-AS1 | blue | RAET1E | blue | FAM214A | cyan | CHMP4C | darkgrey | C19orf70 | grey | KIF20B | grey | PIH2 | grey | UGT1A6 | grey |
| FASLG | blue | RAF1 | blue | FAM222B | cyan | CHRM1 | darkgrey | C19orf80 | grey | KIF21B | grey | PIK3C2B | grey | UGT2B28 | grey |
| FASTK | blue | RALGAPB | blue | FAM35A | cyan | CHRM3 | darkgrey | C19orf82 | grey | KIF24 | grey | PIK3CD | grey | UGT3A1 | grey |
| FATE1 | blue | RAMP1 | blue | FAM47B | cyan | CHST9 | darkgrey | C1QTNF1 | grey | KIF2B | grey | PIK3CG | grey | UHRF1 | grey |
| FBF1 | blue | RAN | blue | FAM63B | cyan | CIB1 | darkgrey | C1QTNF3 | grey | KIF3A | grey | PIK3IP1 | grey | ULK3 | grey |
| FBLL1 | blue | RANBP2 | blue | FAM71C | cyan | CLDN10 | darkgrey | C1QTNF7 | grey | KIF5A | grey | PIK3R3 | grey | UNC119B | grey |
| FBRS | blue | RANBP3 | blue | FAM83F | cyan | CLDN5 | darkgrey | C1QTNF9B-AS1 | grey | KIF7 | grey | PIK3R6 | grey | UNC13B | grey |
| FBXL14 | blue | RANGRF | blue | FAM8A1 | cyan | CLEC14A | darkgrey | C1orf106 | grey | KIF9 | grey | PIM3 | grey | UNC45A | grey |
| FBXL15 | blue | RAP2C | blue | FAM92A1 | cyan | CLIC6 | darkgrey | C1orf109 | grey | KIF9-AS1 | grey | PIN1 | grey | UNC45B | grey |
| FBXL18 | blue | RAPSN | blue | FAM96A | cyan | CLMN | darkgrey | C1orf115 | grey | KIR2DL2 | grey | PINK1 | grey | UNC50 | grey |
| FBXL19 | blue | RARA-AS1 | blue | FAM98B | cyan | CMTM6 | darkgrey | C1orf122 | grey | KIR2DL4 | grey | PINK1-AS | grey | UNC5CL | grey |
| FBXL6 | blue | RARG | blue | FAR1 | cyan | CNPY2 | darkgrey | C1orf127 | grey | KIR2DL5A | grey | PINLYP | grey | UNC5D | grey |
| FBXL8 | blue | RASA2 | blue | FAT2 | cyan | COBL | darkgrey | C1orf131 | grey | KIR2DS1 | grey | PIP4K2C | grey | UNG | grey |
| FBXO10 | blue | RASAL3 | blue | FBL | cyan | COL5A3 | darkgrey | C1orf162 | grey | KIR2DS2 | grey | PIP5K1C | grey | UPK1A | grey |
| FBXO11 | blue | RASGEF1C | blue | FBN3 | cyan | COL6A1 | darkgrey | C1orf167 | grey | KIR2DS3 | grey | PIRT | grey | UPK1B | grey |
| FBXO2 | blue | RASGRF1 | blue | FBP2 | cyan | COL6A2 | darkgrey | C1orf168 | grey | KIR2DS5 | grey | PITPNA-AS1 | grey | UPK2 | grey |
| FBXO24 | blue | RASGRP2 | blue | FBXL16 | cyan | COL9A2 | darkgrey | C1orf177 | grey | KIR3DL1 | grey | PITPNM3 | grey | UPP2 | grey |
| FBXO30 | blue | RASGRP4 | blue | FBXL20 | cyan | COL9A3 | darkgrey | C1orf21 | grey | KIR3DL3 | grey | PITRM1-AS1 | grey | UPRT | grey |
| FBXO39 | blue | RASIP1 | blue | FBXL21 | cyan | COPG1 | darkgrey | C1orf216 | grey | KIR3DS1 | grey | PITX2 | grey | UQCR10 | grey |
| FBXO40 | blue | RASL10B | blue | FBXL3 | cyan | COPZ1 | darkgrey | C1orf220 | grey | KIRREL2 | grey | PIWIL4 | grey | UQCR11 | grey |
| FBXO44 | blue | RASSF1-AS1 | blue | FBXL5 | cyan | COX5A | darkgrey | C1orf226 | grey | KIRREL3-AS2 | grey | PJA1 | grey | UQCRC1 | grey |
| FBXO8 | blue | RAX | blue | FBXO33 | cyan | CP | darkgrey | C1orf234 | grey | KL | grey | PKD1L2 | grey | UQCRFS1 | grey |
| FBXO9 | blue | RAX2 | blue | FBXO34 | cyan | CPD | darkgrey | C1orf50 | grey | KLC1 | grey | PKD1P1 | grey | URB2 | grey |
| FBXW4P1 | blue | RBAKDN | blue | FBXO38 | cyan | CPNE5 | darkgrey | C1orf52 | grey | KLC2 | grey | PKHD1L1 | grey | URGCP | grey |
| FBXW5 | blue | RBBP8NL | blue | FBXO7 | cyan | CRELD2 | darkgrey | C1orf85 | grey | KLF11 | grey | PKI55 | grey | URM1 | grey |
| FCF1 | blue | RBFADN | blue | FBXW11 | cyan | CRISP2 | darkgrey | C1orf87 | grey | KLF13 | grey | PKIA | grey | USH1C | grey |
| FCHO1 | blue | RBFOX3 | blue | FBXW9 | cyan | CSN3 | darkgrey | C1orf94 | grey | KLF14 | grey | PKIB | grey | USP13 | grey |
| FCHSD2 | blue | RBM18 | blue | FCAR | cyan | CST1 | darkgrey | C1orf95 | grey | KLF15 | grey | PKM | grey | USP18 | grey |
| FCN3 | blue | RBM38 | blue | FCHO2 | cyan | CST2 | darkgrey | C2 | grey | KLF17 | grey | PKMYT1 | grey | USP2-AS1 | grey |
| FDXR | blue | RBM4 | blue | FCN2 | cyan | CST4 | darkgrey | C20orf166-AS1 | grey | KLF2 | grey | PKN3 | grey | USP21 | grey |
| FENDRR | blue | RBM4B | blue | FDFT1 | cyan | CST5 | darkgrey | C20orf173 | grey | KLF9 | grey | PKP1 | grey | USP3 | grey |
| FERD3L | blue | RBP2 | blue | FEM1B | cyan | CTA-292E10.6 | darkgrey | C20orf195 | grey | KLHDC1 | grey | PLA1A | grey | USP35 | grey |
| FFAR1 | blue | RBP3 | blue | FEM1C | cyan | CTBS | darkgrey | C20orf197 | grey | KLHDC3 | grey | PLA2G2A | grey | USP36 | grey |
| FGB | blue | RBPJL | blue | FER1L4 | cyan | CUTA | darkgrey | C20orf27 | grey | KLHDC7B | grey | PLA2G2D | grey | USP43 | grey |
| FGD4 | blue | RBPMS-AS1 | blue | FEZ2 | cyan | CXADR | darkgrey | C20orf96 | grey | KLHDC8B | grey | PLA2G2F | grey | USP46-AS1 | grey |
| FGF11 | blue | RC3H1 | blue | FEZF2 | cyan | CXCL10 | darkgrey | C21orf128 | grey | KLHDC9 | grey | PLA2G3 | grey | USP51 | grey |
| FGF17 | blue | RCC1 | blue | FGF4 | cyan | CXCL17 | darkgrey | C21orf15 | grey | KLHL11 | grey | PLA2G4A | grey | UST | grey |
| FGF21 | blue | RCE1 | blue | FGF5 | cyan | CXXC5 | darkgrey | C21orf33 | grey | KLHL12 | grey | PLA2G4C | grey | UVRAG | grey |
| FGF22 | blue | RCHY1 | blue | FIG4 | cyan | CXorf36 | darkgrey | C21orf37 | grey | KLHL13 | grey | PLA2G4D | grey | VAC14-AS1 | grey |
| FGF3 | blue | RCOR1 | blue | FIP1L1 | cyan | CYB561 | darkgrey | C21orf49 | grey | KLHL2 | grey | PLA2G7 | grey | VAMP1 | grey |
| FGF6 | blue | RCOR2 | blue | FKBP15 | cyan | CYGB | darkgrey | C22orf23 | grey | KLHL21 | grey | PLAA | grey | VAMP5 | grey |
| FGFR1OP2 | blue | RD3 | blue | FKBP5 | cyan | CYP26B1 | darkgrey | C22orf24 | grey | KLHL26 | grey | PLAC8 | grey | VANGL2 | grey |
| FGFR4 | blue | RDH13 | blue | FKBP7 | cyan | CYP39A1 | darkgrey | C2CD4A | grey | KLHL29 | grey | PLAG1 | grey | VASN | grey |
| FGG | blue | RDH16 | blue | FLI1 | cyan | CYP4B1 | darkgrey | C2orf27A | grey | KLHL3 | grey | PLAU | grey | VASP | grey |
| FGL1 | blue | REC8 | blue | FLJ13224 | cyan | CYTIP | darkgrey | C2orf42 | grey | KLHL30-AS1 | grey | PLAUR | grey | VAT1L | grey |
| FHL3 | blue | RECQL4 | blue | FLJ16779 | cyan | DANCR | darkgrey | C2orf44 | grey | KLHL41 | grey | PLBD2 | grey | VAV1 | grey |
| FIBCD1 | blue | REEP2 | blue | FLJ22184 | cyan | DAP | darkgrey | C2orf47 | grey | KLHL42 | grey | PLCB2 | grey | VAX2 | grey |
| FITM1 | blue | REEP3 | blue | FLJ31306 | cyan | DBI | darkgrey | C2orf54 | grey | KLK10 | grey | PLCD1 | grey | VCAM1 | grey |
| FKBP6 | blue | REG3A | blue | FLJ32790 | cyan | DBN1 | darkgrey | C2orf57 | grey | KLK11 | grey | PLCD4 | grey | VCP | grey |
| FKBP8 | blue | REG3G | blue | FLJ34521 | cyan | DENND2A | darkgrey | C2orf61 | grey | KLK7 | grey | PLCG1-AS1 | grey | VEGFB | grey |
| FKBPL | blue | REL | blue | FMNL2 | cyan | DEPTOR | darkgrey | C2orf71 | grey | KLLN | grey | PLCL1 | grey | VEGFC | grey |
| FKTN | blue | REM1 | blue | FMO9P | cyan | DGKA | darkgrey | C2orf73 | grey | KLRAP1 | grey | PLCL2 | grey | VENTX | grey |
| FLJ10038 | blue | RERG | blue | FMR1 | cyan | DHTKD1 | darkgrey | C2orf80 | grey | KLRB1 | grey | PLCXD1 | grey | VGLL2 | grey |
| FLJ11710 | blue | RETN | blue | FMR1NB | cyan | DMBT1 | darkgrey | C2orf81 | grey | KLRF1 | grey | PLCXD2 | grey | VGLL3 | grey |
| FLJ13744 | blue | RETNLB | blue | FNBP1 | cyan | DNAJB9 | darkgrey | C2orf88 | grey | KLRG1 | grey | PLCXD3 | grey | VGLL4 | grey |
| FLJ13773 | blue | REXO1 | blue | FNBP4 | cyan | DNAJC1 | darkgrey | C3 | grey | KLRG2 | grey | PLD1 | grey | VIPR1 | grey |
| FLJ20021 | blue | REXO4 | blue | FNDC3A | cyan | DNAJC3 | darkgrey | C3AR1 | grey | KMO | grey | PLD3 | grey | VLDLR | grey |
| FLJ20712 | blue | RFC4 | blue | FNIP1 | cyan | DNASE2B | darkgrey | C3orf14 | grey | KNTC1 | grey | PLEK2 | grey | VMP1 | grey |
| FLJ25758 | blue | RFWD2 | blue | FNIP2 | cyan | DNER | darkgrey | C3orf33 | grey | KPTN | grey | PLEKHA6 | grey | VN1R1 | grey |
| FLJ26850 | blue | RFX5 | blue | FNTA | cyan | DPAGT1 | darkgrey | C3orf35 | grey | KRBA1 | grey | PLEKHA7 | grey | VN1R2 | grey |
| FLJ30679 | blue | RFX7 | blue | FOXE3 | cyan | DPM2 | darkgrey | C3orf43 | grey | KRBOX1-AS1 | grey | PLEKHF1 | grey | VN1R4 | grey |
| FLJ31356 | blue | RFXANK | blue | FOXJ3 | cyan | DPP4 | darkgrey | C3orf49 | grey | KRBOX4 | grey | PLEKHG1 | grey | VN1R5 | grey |
| FLJ31713 | blue | RGAG4 | blue | FOXN1 | cyan | DSC2 | darkgrey | C3orf58 | grey | KRIT1 | grey | PLEKHG2 | grey | VNN2 | grey |
| FLJ31945 | blue | RGL4 | blue | FOXN2 | cyan | DSG2 | darkgrey | C3orf70 | grey | KRT1 | grey | PLEKHG4 | grey | VNN3 | grey |
| FLJ32154 | blue | RGMB-AS1 | blue | FOXN3 | cyan | DSP | darkgrey | C4BPA | grey | KRT10 | grey | PLEKHG6 | grey | VPREB1 | grey |
| FLJ33544 | blue | RGS14 | blue | FOXO3 | cyan | DTNB | darkgrey | C4orf17 | grey | KRT12 | grey | PLEKHM2 | grey | VPREB3 | grey |
| FLJ36840 | blue | RGS16 | blue | FOXP1 | cyan | DTX4 | darkgrey | C4orf32 | grey | KRT13 | grey | PLEKHM3 | grey | VPS26B | grey |
| FLJ37453 | blue | RGS20 | blue | FRMD6 | cyan | DUS1L | darkgrey | C4orf45 | grey | KRT17P5 | grey | PLEKHO1 | grey | VPS28 | grey |
| FLJ38668 | blue | RGS6 | blue | FRS2 | cyan | DUSP1 | darkgrey | C4orf46 | grey | KRT2 | grey | PLEKHO2 | grey | VPS33B | grey |
| FLJ40288 | blue | RGS9BP | blue | FTH1 | cyan | EFCAB4A | darkgrey | C4orf48 | grey | KRT222 | grey | PLGLB2 | grey | VPS37B | grey |
| FLJ44087 | blue | RHBDD3 | blue | FTO | cyan | EFCAB4B | darkgrey | C4orf6 | grey | KRT25 | grey | PLGRKT | grey | VPS4A | grey |
| FLJ45482 | blue | RHBDF2 | blue | FTSJ1 | cyan | EHD2 | darkgrey | C5 | grey | KRT27 | grey | PLIN2 | grey | VPS4B | grey |
| FLJ46026 | blue | RHBDL1 | blue | FTX | cyan | EHF | darkgrey | C5orf17 | grey | KRT31 | grey | PLIN3 | grey | VPS72 | grey |
| FLNC | blue | RHBG | blue | FUNDC1 | cyan | EI24 | darkgrey | C5orf30 | grey | KRT33B | grey | PLK2 | grey | VPS8 | grey |
| FLVCR1-AS1 | blue | RHCG | blue | FUNDC2 | cyan | EIF2AK3 | darkgrey | C5orf45 | grey | KRT36 | grey | PLN | grey | VRK1 | grey |
| FLYWCH2 | blue | RHEBL1 | blue | FUT11 | cyan | EIF4EBP1 | darkgrey | C5orf64 | grey | KRT38 | grey | PLOD1 | grey | VSIG10 | grey |
| FMN2 | blue | RHOBTB2 | blue | FUT3 | cyan | ELF5 | darkgrey | C5orf66 | grey | KRT71 | grey | PLOD3 | grey | VSTM1 | grey |
| FNDC8 | blue | RHOD | blue | FXYD5 | cyan | ELL2 | darkgrey | C6orf1 | grey | KRT75 | grey | PLP1 | grey | VWA9 | grey |
| FOLR3 | blue | RHOF | blue | FYTTD1 | cyan | ENPP3 | darkgrey | C6orf141 | grey | KRT82 | grey | PLP2 | grey | WAC-AS1 | grey |
| FOPNL | blue | RHOT1 | blue | FZD7 | cyan | ENTPD3 | darkgrey | C6orf195 | grey | KRT84 | grey | PLS1 | grey | WARS | grey |
| FOXA2 | blue | RHOT2 | blue | G3BP1 | cyan | ENTPD4 | darkgrey | C6orf201 | grey | KRT9 | grey | PLSCR2 | grey | WAS | grey |
| FOXA3 | blue | RHOV | blue | GABARAPL2 | cyan | EPB41 | darkgrey | C6orf203 | grey | KRTAP11-1 | grey | PLTP | grey | WASF3 | grey |
| FOXB1 | blue | RHPN1 | blue | GABBR1 | cyan | EPCAM | darkgrey | C6orf48 | grey | KRTAP13-1 | grey | PLXDC1 | grey | WBP2 | grey |
| FOXC2 | blue | RHPN1-AS1 | blue | GABPB2 | cyan | EPPK1 | darkgrey | C6orf57 | grey | KRTAP17-1 | grey | PLXNA1 | grey | WBP2NL | grey |
| FOXD2 | blue | RIBC2 | blue | GAD2 | cyan | EPRS | darkgrey | C6orf99 | grey | KRTAP19-3 | grey | PLXNA3 | grey | WBP5 | grey |
| FOXD2-AS1 | blue | RICTOR | blue | GAGE3 | cyan | ERG | darkgrey | C7 | grey | KRTAP3-1 | grey | PLXNB2 | grey | WBSCR22 | grey |
| FOXD3 | blue | RIMS3 | blue | GALC | cyan | ERO1L | darkgrey | C7orf31 | grey | KRTAP4-1 | grey | PM20D1 | grey | WBSCR27 | grey |
| FOXD3-AS1 | blue | RIMS4 | blue | GALNT1 | cyan | ERO1LB | darkgrey | C7orf33 | grey | KRTAP4-11 | grey | PMCH | grey | WDFY3-AS2 | grey |
| FOXE1 | blue | RIN1 | blue | GAMT | cyan | ESAM | darkgrey | C7orf55 | grey | KRTAP4-12 | grey | PMF1 | grey | WDR16 | grey |
| FOXG1 | blue | RIN3 | blue | GANAB | cyan | ESRP1 | darkgrey | C7orf60 | grey | KRTAP4-5 | grey | PMM2 | grey | WDR18 | grey |
| FOXH1 | blue | RIOK2 | blue | GANC | cyan | ETV6 | darkgrey | C7orf62 | grey | KRTAP4-8 | grey | PMPCA | grey | WDR20 | grey |
| FOXJ1 | blue | RIPK2 | blue | GAPDHS | cyan | EXPH5 | darkgrey | C7orf63 | grey | KRTAP4-9 | grey | PMS2L2 | grey | WDR34 | grey |
| FOXL1 | blue | RIPPLY2 | blue | GARS | cyan | EZR | darkgrey | C7orf69 | grey | KRTAP5-2 | grey | PMS2P3 | grey | WDR35 | grey |
| FOXN3-AS1 | blue | RIT1 | blue | GATAD1 | cyan | F2RL1 | darkgrey | C8B | grey | KRTAP5-9 | grey | PMS2P4 | grey | WDR41 | grey |
| FOXN4 | blue | RLBP1 | blue | GBAS | cyan | F3 | darkgrey | C8orf22 | grey | KRTAP7-1 | grey | PMS2P8 | grey | WDR43 | grey |
| FOXO4 | blue | RLN3 | blue | GDE1 | cyan | FA2H | darkgrey | C8orf31 | grey | KRTAP8-1 | grey | PNLDC1 | grey | WDR45 | grey |
| FOXP3 | blue | RLTPR | blue | GDI2 | cyan | FABP3 | darkgrey | C8orf33 | grey | KRTAP9-4 | grey | PNLIPRP3 | grey | WDR45B | grey |
| FOXS1 | blue | RMDN3 | blue | GDPD2 | cyan | FADS3 | darkgrey | C8orf34 | grey | KRTDAP | grey | PNMA2 | grey | WDR52 | grey |
| FPGS | blue | RMND5B | blue | GFM2 | cyan | FAIM3 | darkgrey | C8orf37 | grey | L1CAM | grey | PNMAL1 | grey | WDR54 | grey |
| FPGT | blue | RNASE3 | blue | GGPS1 | cyan | FAM160A1 | darkgrey | C8orf48 | grey | L3HYPDH | grey | PNP | grey | WDR63 | grey |
| FRMD1 | blue | RND1 | blue | GHITM | cyan | FAM171B | darkgrey | C8orf60 | grey | L3MBTL2 | grey | PNPLA1 | grey | WDR70 | grey |
| FRMD8P1 | blue | RND2 | blue | GIF | cyan | FAM174B | darkgrey | C8orf76 | grey | L3MBTL4 | grey | PNPLA4 | grey | WDR73 | grey |
| FRMPD1 | blue | RNF103 | blue | GIMAP7 | cyan | FAM20A | darkgrey | C9orf16 | grey | LA16c-358B7.3 | grey | PNPO | grey | WDR77 | grey |
| FRMPD4 | blue | RNF121 | blue | GINM1 | cyan | FAM3B | darkgrey | C9orf163 | grey | LA16c-380H5.5 | grey | POC1B | grey | WDR81 | grey |
| FRYL | blue | RNF126P1 | blue | GINS2 | cyan | FAM46A | darkgrey | C9orf173 | grey | LA16c-395F10.1 | grey | PODNL1 | grey | WDR86 | grey |
| FSCN2 | blue | RNF14 | blue | GINS4 | cyan | FAM46C | darkgrey | C9orf3 | grey | LACTB | grey | POFUT1 | grey | WDR86-AS1 | grey |
| FSD1 | blue | RNF149 | blue | GIP | cyan | FAM60A | darkgrey | C9orf38 | grey | LACTB2 | grey | POLA2 | grey | WDR93 | grey |
| FSHB | blue | RNF151 | blue | GJB3 | cyan | FAM84B | darkgrey | C9orf41 | grey | LAG3 | grey | POLD4 | grey | WDSUB1 | grey |
| FSTL3 | blue | RNF167 | blue | GJD2 | cyan | FBLN2 | darkgrey | C9orf47 | grey | LAIR2 | grey | POLDIP2 | grey | WEE1 | grey |
| FTCD | blue | RNF168 | blue | GLG1 | cyan | FBXO16 | darkgrey | C9orf64 | grey | LALBA | grey | POLE | grey | WFDC1 | grey |
| FTCDNL1 | blue | RNF186 | blue | GLIPR1 | cyan | FCRL5 | darkgrey | C9orf72 | grey | LAMA2 | grey | POLE2 | grey | WFDC10B | grey |
| FTHL17 | blue | RNF20 | blue | GLO1 | cyan | FDCSP | darkgrey | C9orf9 | grey | LAMA5 | grey | POLG2 | grey | WFDC11 | grey |
| FUBP1 | blue | RNF207 | blue | GLOD4 | cyan | FEZ1 | darkgrey | C9orf91 | grey | LAMB3 | grey | POLM | grey | WFDC12 | grey |
| FUK | blue | RNF212 | blue | GLOD5 | cyan | FGD5 | darkgrey | CA1 | grey | LAMP3 | grey | POLR1A | grey | WFDC21P | grey |
| FUOM | blue | RNF217 | blue | GLRX5 | cyan | FGF12 | darkgrey | CA11 | grey | LAMP5 | grey | POLR2E | grey | WFDC5 | grey |
| FUT1 | blue | RNF25 | blue | GLT8D1 | cyan | FGF14-AS2 | darkgrey | CA12 | grey | LAMTOR1 | grey | POLR2G | grey | WFDC9 | grey |
| FUT5 | blue | RNF26 | blue | GLUD1 | cyan | FHL1 | darkgrey | CA14 | grey | LAMTOR2 | grey | POLR2H | grey | WFS1 | grey |
| FUT7 | blue | RNF31 | blue | GLUD2 | cyan | FKBP10 | darkgrey | CA3 | grey | LAMTOR4 | grey | POLR2L | grey | WHAMM | grey |
| FUT8 | blue | RNF44 | blue | GLUL | cyan | FKBP11 | darkgrey | CA4 | grey | LAMTOR5-AS1 | grey | POLR3F | grey | WI2-89031B12.1 | grey |
| FUZ | blue | RNF6 | blue | GMCL1 | cyan | FLT4 | darkgrey | CA8 | grey | LAPTM5 | grey | POLR3GL | grey | WIBG | grey |
| FXR1 | blue | RNGTT | blue | GMFB | cyan | FMO5 | darkgrey | CAAP1 | grey | LARGE | grey | POM121 | grey | WIF1 | grey |
| FXR2 | blue | RNPEPL1 | blue | GMNN | cyan | FMO6P | darkgrey | CAB39L | grey | LAS1L | grey | POM121L2 | grey | WISP3 | grey |
| FXYD2 | blue | ROM1 | blue | GNA13 | cyan | FNBP1L | darkgrey | CABLES2 | grey | LAT2 | grey | POM121L8P | grey | WNT10B | grey |
| FZD2 | blue | RORA | blue | GNAI3 | cyan | FOLH1B | darkgrey | CABP5 | grey | LAX1 | grey | POM121L9P | grey | WNT2 | grey |
| FZD9 | blue | RP1-101G11.2 | blue | GNAQ | cyan | FOLR1 | darkgrey | CACNA2D3 | grey | LBH | grey | POMGNT2 | grey | WNT2B | grey |
| FZR1 | blue | RP1-118J21.25 | blue | GNB1 | cyan | FOXC1 | darkgrey | CACNA2D4 | grey | LBP | grey | POMK | grey | WNT8A | grey |
| G2E3 | blue | RP1-149A16.17 | blue | GNB4 | cyan | FOXQ1 | darkgrey | CACNG6 | grey | LBX2-AS1 | grey | POMT1 | grey | WNT9B | grey |
| G3BP2 | blue | RP1-155D22.1 | blue | GNG10 | cyan | FRK | darkgrey | CADM2 | grey | LCA5 | grey | POMZP3 | grey | WT1 | grey |
| GAB4 | blue | RP1-155D22.2 | blue | GNG12 | cyan | FRMPD3 | darkgrey | CADM2-AS1 | grey | LCE1B | grey | POP4 | grey | WT1-AS | grey |
| GABARAPL3 | blue | RP1-170O19.17 | blue | GNG5 | cyan | FXN | darkgrey | CADM3-AS1 | grey | LCE1E | grey | POP7 | grey | XAB2 | grey |
| GABBR2 | blue | RP1-192P9.1 | blue | GNG7 | cyan | FXYD1 | darkgrey | CAHM | grey | LCE2B | grey | POPDC3 | grey | XAGE-4 | grey |
| GABPA | blue | RP1-199J3.7 | blue | GNL2 | cyan | FXYD3 | darkgrey | CALCB | grey | LCE3D | grey | POR | grey | XAGE2 | grey |
| GABPB1 | blue | RP1-202O8.3 | blue | GNPAT | cyan | FZD3 | darkgrey | CALCRL | grey | LCK | grey | POTEM | grey | XCL1 | grey |
| GABRD | blue | RP1-20C7.6 | blue | GNPDA2 | cyan | GALNT13 | darkgrey | CALHM1 | grey | LCLAT1 | grey | POU2F3 | grey | XCR1 | grey |
| GABRQ | blue | RP1-212P9.2 | blue | GNS | cyan | GALNT3 | darkgrey | CALM1 | grey | LCN10 | grey | POU3F3 | grey | XG | grey |
| GABRR2 | blue | RP1-263J7.2 | blue | GOLGA4 | cyan | GALNT7 | darkgrey | CALML6 | grey | LCN12 | grey | POU4F1 | grey | XIRP1 | grey |
| GADD45G | blue | RP1-286D6.5 | blue | GOLGA5 | cyan | GATA3 | darkgrey | CALR | grey | LCN6 | grey | POU4F2 | grey | XIST | grey |
| GADD45GIP1 | blue | RP1-28O17.1 | blue | GOLGA7 | cyan | GCH1 | darkgrey | CALR3 | grey | LCORL | grey | PP12613 | grey | XKR7 | grey |
| GAL | blue | RP1-305G21.1 | blue | GOLGA8A | cyan | GCNT2 | darkgrey | CAMK1 | grey | LCT | grey | PP12719 | grey | XKRX | grey |
| GAL3ST1 | blue | RP11-1007O24.3 | blue | GOLGA8N | cyan | GFPT1 | darkgrey | CAMK1D | grey | LDB1 | grey | PP13 | grey | XPA | grey |
| GAL3ST2 | blue | RP11-1017G21.5 | blue | GOLGB1 | cyan | GGT5 | darkgrey | CAMK1G | grey | LDHAL6A | grey | PP13439 | grey | XPC | grey |
| GAL3ST3 | blue | RP11-102C16.3 | blue | GOLPH3 | cyan | GK5 | darkgrey | CAMK4 | grey | LDHAL6B | grey | PP14571 | grey | XPO5 | grey |
| GALE | blue | RP11-1078H9.6 | blue | GOLPH3L | cyan | GLCCI1 | darkgrey | CAMLG | grey | LDLR | grey | PPAPDC1A | grey | XRCC2 | grey |
| GALK1 | blue | RP11-108B14.5 | blue | GORASP2 | cyan | GLTP | darkgrey | CAMTA2 | grey | LDLRAD4-AS1 | grey | PPAPDC3 | grey | XRN2 | grey |
| GALNT8 | blue | RP11-108L7.15 | blue | GOT2 | cyan | GLYR1 | darkgrey | CAND1 | grey | LECT2 | grey | PPARGC1A | grey | XXYLT1 | grey |
| GALNT9 | blue | RP11-109E24.2 | blue | GP2 | cyan | GMDS | darkgrey | CAND1.11 | grey | LEF1 | grey | PPBP | grey | XXbac-B476C20.9 | grey |
| GALR3 | blue | RP11-109M19.1 | blue | GPATCH8 | cyan | GMPR | darkgrey | CAPG | grey | LEMD1 | grey | PPBPP2 | grey | Y16709 | grey |
| GALT | blue | RP11-10N23.4 | blue | GPBP1 | cyan | GNPNAT1 | darkgrey | CAPN15 | grey | LEMD2 | grey | PPEF2 | grey | YAF2 | grey |
| GAPVD1 | blue | RP11-1103G16.1 | blue | GPCPD1 | cyan | GPBAR1 | darkgrey | CAPN3 | grey | LENEP | grey | PPFIBP2 | grey | YARS | grey |
| GAS2L2 | blue | RP11-111M22.3 | blue | GPD1L | cyan | GPIHBP1 | darkgrey | CAPN6 | grey | LENG9 | grey | PPIF | grey | YARS2 | grey |
| GAS6-AS1 | blue | RP11-114H24.6 | blue | GPD2 | cyan | GPM6B | darkgrey | CAPN8 | grey | LEPRE1 | grey | PPIH | grey | YBX2 | grey |
| GATA1 | blue | RP11-1151B14.3 | blue | GPI | cyan | GPR160 | darkgrey | CAPRIN2 | grey | LEPREL4 | grey | PPIP5K1 | grey | YEATS2 | grey |
| GATA2 | blue | RP11-1191J2.5 | blue | GPN1 | cyan | GPRIN3 | darkgrey | CAPSL | grey | LFNG | grey | PPIP5K2 | grey | YEATS4 | grey |
| GATA3-AS1 | blue | RP11-1277A3.3 | blue | GPR114 | cyan | GRHL1 | darkgrey | CAPZA3 | grey | LGALS13 | grey | PPL | grey | YIF1A | grey |
| GATA4 | blue | RP11-12M5.1 | blue | GPR137B | cyan | GRHL2 | darkgrey | CARD16 | grey | LGALS3 | grey | PPM1M | grey | YIPF1 | grey |
| GATA5 | blue | RP11-155O18.6 | blue | GPR150 | cyan | GSN | darkgrey | CARD6 | grey | LGALS3BP | grey | PPP1R10 | grey | YIPF4 | grey |
| GATAD2B | blue | RP11-161D15.1 | blue | GPR55 | cyan | GSR | darkgrey | CARNS1 | grey | LGALS9 | grey | PPP1R13B | grey | YOD1 | grey |
| GATC | blue | RP11-164P12.3 | blue | GPR6 | cyan | HEIH | darkgrey | CASC10 | grey | LGR5 | grey | PPP1R13L | grey | YPEL3 | grey |
| GATSL2 | blue | RP11-190A12.8 | blue | GPRASP1 | cyan | HERPUD1 | darkgrey | CASC17 | grey | LGR6 | grey | PPP1R14A | grey | YPEL4 | grey |
| GATSL3 | blue | RP11-196G18.23 | blue | GPS2 | cyan | HINT1 | darkgrey | CASC7 | grey | LHCGR | grey | PPP1R14B | grey | YRDC | grey |
| GBP1 | blue | RP11-203B7.1 | blue | GPX3 | cyan | HIP1R | darkgrey | CASD1 | grey | LHFPL2 | grey | PPP1R14D | grey | YTHDF1 | grey |
| GBX1 | blue | RP11-209D14.2 | blue | GRAMD1A | cyan | HIPK2 | darkgrey | CASKIN2 | grey | LHFPL5 | grey | PPP1R15A | grey | YY1AP1 | grey |
| GBX2 | blue | RP11-210M15.2 | blue | GRASPOS | cyan | HIST1H2BN | darkgrey | CASP1 | grey | LHPP | grey | PPP1R18 | grey | ZBED1 | grey |
| GCAT | blue | RP11-217B1.2 | blue | GREB1 | cyan | HMGA1 | darkgrey | CASP12 | grey | LHX2 | grey | PPP1R26 | grey | ZBED2 | grey |
| GCC1 | blue | RP11-217B7.2 | blue | GRHPR | cyan | HMGCS2 | darkgrey | CASP3 | grey | LHX3 | grey | PPP1R36 | grey | ZBED3 | grey |
| GCFC2 | blue | RP11-245P10.8 | blue | GRIK3 | cyan | HS3ST3B1 | darkgrey | CASP4 | grey | LHX4-AS1 | grey | PPP1R3C | grey | ZBED4 | grey |
| GCGR | blue | RP11-24D15.1 | blue | GRM1 | cyan | HSD17B2 | darkgrey | CASP7 | grey | LHX6 | grey | PPP1R3E | grey | ZBED5 | grey |
| GCHFR | blue | RP11-252E2.1 | blue | GRSF1 | cyan | HSH2D | darkgrey | CASQ1 | grey | LHX8 | grey | PPP1R9B | grey | ZBED6 | grey |
| GCK | blue | RP11-259G18.1 | blue | GRTP1 | cyan | HSP90B1 | darkgrey | CASQ2 | grey | LIAS | grey | PPP2R1A | grey | ZBED6CL | grey |
| GCKR | blue | RP11-25I15.3 | blue | GSE1 | cyan | HSPA12B | darkgrey | CATSPER1 | grey | LIF | grey | PPP2R1B | grey | ZBED8 | grey |
| GCLC | blue | RP11-272D12.1 | blue | GSK3B | cyan | HSPA5 | darkgrey | CBLL1 | grey | LIG1 | grey | PPP2R2D | grey | ZBED9 | grey |
| GCNT7 | blue | RP11-285G1.14 | blue | GSPT1 | cyan | HSPB2 | darkgrey | CBLN1 | grey | LILRA6 | grey | PPP2R3C | grey | ZBTB1 | grey |
| GDAP1L1 | blue | RP11-288L9.1 | blue | GSPT2 | cyan | HSPB6 | darkgrey | CBLN2 | grey | LILRB3 | grey | PPP5C | grey | ZBTB16 | grey |
| GDF15 | blue | RP11-304L19.4 | blue | GSTK1 | cyan | HSPB7 | darkgrey | CBLN3 | grey | LILRB4 | grey | PPP6R1 | grey | ZBTB26 | grey |
| GDF2 | blue | RP11-305L7.3 | blue | GSTO1 | cyan | HTN1 | darkgrey | CBR1 | grey | LIMD1-AS1 | grey | PPRC1 | grey | ZBTB39 | grey |
| GDNF | blue | RP11-320N7.2 | blue | GTF2A1 | cyan | HYOU1 | darkgrey | CBR3 | grey | LIMD2 | grey | PPWD1 | grey | ZBTB41 | grey |
| GEMIN4 | blue | RP11-332H18.4 | blue | GTF2A2 | cyan | IDH2 | darkgrey | CBX6 | grey | LIMS2 | grey | PQLC3 | grey | ZBTB42 | grey |
| GEMIN7 | blue | RP11-353N14.1 | blue | GTF2B | cyan | IFNLR1 | darkgrey | CBX7 | grey | LIN28A | grey | PRADC1 | grey | ZBTB47 | grey |
| GET4 | blue | RP11-357G3.1 | blue | GTF2E1 | cyan | IGHD | darkgrey | CBY3 | grey | LIN54 | grey | PRAF2 | grey | ZBTB6 | grey |
| GFER | blue | RP11-384L8.1 | blue | GTF2I | cyan | IGHM | darkgrey | CC2D2B | grey | LINC00052 | grey | PRAMEF10 | grey | ZBTB7C | grey |
| GFI1B | blue | RP11-396F22.1 | blue | GTF3A | cyan | IGHV3-54 | darkgrey | CCBE1 | grey | LINC00086 | grey | PRAMEF11 | grey | ZBTB8A | grey |
| GFM1 | blue | RP11-399E6.1 | blue | GTF3C2 | cyan | IGJ | darkgrey | CCDC102A | grey | LINC00094 | grey | PRAMEF12 | grey | ZBTB9 | grey |
| GFOD2 | blue | RP11-400N9.1 | blue | GTF3C2-AS1 | cyan | IGK | darkgrey | CCDC102B | grey | LINC00102 | grey | PRB1 | grey | ZC2HC1B | grey |
| GFRA2 | blue | RP11-402D21.2 | blue | GTF3C6 | cyan | IGKC | darkgrey | CCDC106 | grey | LINC00112 | grey | PRB3 | grey | ZC2HC1C | grey |
| GFRA3 | blue | RP11-403P17.3 | blue | GUCY1A3 | cyan | IGLC1 | darkgrey | CCDC107 | grey | LINC00113 | grey | PRB4 | grey | ZC3H12C | grey |
| GFRA4 | blue | RP11-408I18.9 | blue | GUSBP1 | cyan | IGLJ3 | darkgrey | CCDC109B | grey | LINC00115 | grey | PRC1 | grey | ZC3H12D | grey |
| GGACT | blue | RP11-414H17.5 | blue | GUSBP2 | cyan | IGLL3P | darkgrey | CCDC110 | grey | LINC00116 | grey | PRCC | grey | ZC3H18 | grey |
| GGN | blue | RP11-425D10.10 | blue | GVQW1 | cyan | IGLL5 | darkgrey | CCDC112 | grey | LINC00165 | grey | PRDM12 | grey | ZC4H2 | grey |
| GGT7 | blue | RP11-426C22.5 | blue | GYG1 | cyan | IGLV1-44 | darkgrey | CCDC113 | grey | LINC00174 | grey | PRDM4 | grey | ZCCHC10 | grey |
| GGTLC2 | blue | RP11-429B14.4 | blue | H2AFV | cyan | IGSF11 | darkgrey | CCDC115 | grey | LINC00184 | grey | PRDM6 | grey | ZCCHC12 | grey |
| GH2 | blue | RP11-432J9.6 | blue | H2AFY | cyan | IKZF3 | darkgrey | CCDC122 | grey | LINC00210 | grey | PRDM9 | grey | ZCCHC18 | grey |
| GHRH | blue | RP11-435O5.5 | blue | H2AFZ | cyan | IL2RG | darkgrey | CCDC124 | grey | LINC00216 | grey | PRDX5 | grey | ZCCHC5 | grey |
| GHRHR | blue | RP11-436D10.3 | blue | H2BFS | cyan | IMP4 | darkgrey | CCDC13-AS1 | grey | LINC00221 | grey | PRELID2 | grey | ZCWPW1 | grey |
| GHRL | blue | RP11-440L14.3 | blue | HADHA | cyan | INSIG1 | darkgrey | CCDC130 | grey | LINC00242 | grey | PREX1 | grey | ZDBF2 | grey |
| GHRLOS | blue | RP11-442O18.2 | blue | HADHB | cyan | INSR | darkgrey | CCDC137 | grey | LINC00244 | grey | PRF1 | grey | ZDHHC21 | grey |
| GHSR | blue | RP11-443C10.1 | blue | HAND2-AS1 | cyan | IRF6 | darkgrey | CCDC138 | grey | LINC00260 | grey | PRG4 | grey | ZDHHC23 | grey |
| GIPC3 | blue | RP11-445L13__B.3 | blue | HAP1 | cyan | IRX3 | darkgrey | CCDC14 | grey | LINC00261 | grey | PRICKLE2 | grey | ZDHHC4 | grey |
| GIPR | blue | RP11-457I16.2 | blue | HAPLN3 | cyan | ISG20 | darkgrey | CCDC140 | grey | LINC00263 | grey | PRICKLE2-AS3 | grey | ZDHHC9 | grey |
| GIT1 | blue | RP11-45M22.5 | blue | HAT1 | cyan | ISLR | darkgrey | CCDC146 | grey | LINC00266-1 | grey | PRIM1 | grey | ZEB1-AS1 | grey |
| GJA3 | blue | RP11-461A8.4 | blue | HAUS8 | cyan | ITGA2 | darkgrey | CCDC148 | grey | LINC00269 | grey | PRIM2 | grey | ZFAND2A | grey |
| GJA8 | blue | RP11-464F9.20 | blue | HBQ1 | cyan | ITGB1BP1 | darkgrey | CCDC152 | grey | LINC00280 | grey | PRIMA1 | grey | ZFAND2B | grey |
| GJB1 | blue | RP11-465B22.8 | blue | HCFC2 | cyan | ITGB8 | darkgrey | CCDC155 | grey | LINC00284 | grey | PRIMPOL | grey | ZFAND5 | grey |
| GJC2 | blue | RP11-473I1.9 | blue | HCRTR2 | cyan | ITIH5 | darkgrey | CCDC167 | grey | LINC00290 | grey | PRKAB1 | grey | ZFAT | grey |
| GJD3 | blue | RP11-480I12.10 | blue | HDGF | cyan | ITM2C | darkgrey | CCDC170 | grey | LINC00298 | grey | PRKAG1 | grey | ZFHX4-AS1 | grey |
| GJD4 | blue | RP11-488L18.10 | blue | HDHD1 | cyan | ITPR2 | darkgrey | CCDC172 | grey | LINC00305 | grey | PRKAG3 | grey | ZFP30 | grey |
| GKN1 | blue | RP11-489E7.4 | blue | HDHD2 | cyan | ITPRIPL1 | darkgrey | CCDC173 | grey | LINC00307 | grey | PRKCD | grey | ZFP36 | grey |
| GLI1 | blue | RP11-498E2.7 | blue | HEATR1 | cyan | IVNS1ABP | darkgrey | CCDC176 | grey | LINC00309 | grey | PRKG1 | grey | ZFP37 | grey |
| GLI4 | blue | RP11-508N22.12 | blue | HEATR5B | cyan | JTB | darkgrey | CCDC177 | grey | LINC00312 | grey | PRKG1-AS1 | grey | ZFP57 | grey |
| GLIS3-AS1 | blue | RP11-517C16.2 | blue | HEBP1 | cyan | JUP | darkgrey | CCDC181 | grey | LINC00317 | grey | PRKG2 | grey | ZFP92 | grey |
| GLP2R | blue | RP11-536G4.2 | blue | HECTD1 | cyan | KANK2 | darkgrey | CCDC184 | grey | LINC00319 | grey | PRKX | grey | ZFPM2 | grey |
| GLRA1 | blue | RP11-543C4.1 | blue | HECTD3 | cyan | KCNA3 | darkgrey | CCDC23 | grey | LINC00324 | grey | PRM1 | grey | ZFY | grey |
| GLRA2 | blue | RP11-552F3.10 | blue | HECW1 | cyan | KCNK6 | darkgrey | CCDC3 | grey | LINC00327 | grey | PRMT6 | grey | ZFYVE1 | grey |
| GLS | blue | RP11-554D14.1 | blue | HERC2 | cyan | KDELR1 | darkgrey | CCDC38 | grey | LINC00330 | grey | PRMT9 | grey | ZFYVE9 | grey |
| GLTPD2 | blue | RP11-560A15.4 | blue | HEXB | cyan | KIAA0040 | darkgrey | CCDC42 | grey | LINC00337 | grey | PRO1082 | grey | ZHX1-C8orf76 | grey |
| GLTSCR2 | blue | RP11-560G2.2 | blue | HGD | cyan | KIAA0125 | darkgrey | CCDC51 | grey | LINC00339 | grey | PRO1804 | grey | ZIC1 | grey |
| GLYCTK | blue | RP11-5N11.2 | blue | HHLA2 | cyan | KIAA1324 | darkgrey | CCDC54 | grey | LINC00347 | grey | PRO2012 | grey | ZIC2 | grey |
| GMEB2 | blue | RP11-613M5.1 | blue | HIAT1 | cyan | KIAA1522 | darkgrey | CCDC60 | grey | LINC00408 | grey | PRO2214 | grey | ZIC3 | grey |
| GMPPA | blue | RP11-619L12.4 | blue | HIBADH | cyan | KIF21A | darkgrey | CCDC66 | grey | LINC00410 | grey | PRO2852 | grey | ZIC5 | grey |
| GMPS | blue | RP11-629O1.2 | blue | HIF1A | cyan | KIT | darkgrey | CCDC68 | grey | LINC00423 | grey | PRO2958 | grey | ZKSCAN4 | grey |
| GNA15 | blue | RP11-635N19.1 | blue | HIGD1A | cyan | KLF5 | darkgrey | CCDC7 | grey | LINC00452 | grey | PRO2964 | grey | ZMAT1 | grey |
| GNAT1 | blue | RP11-63D14.1 | blue | HIPK1 | cyan | KLHDC8A | darkgrey | CCDC79 | grey | LINC00462 | grey | PROCR | grey | ZMAT4 | grey |
| GNB1L | blue | RP11-63K6.7 | blue | HIPK3 | cyan | KLHL6 | darkgrey | CCDC82 | grey | LINC00466 | grey | PROK1 | grey | ZMAT5 | grey |
| GNB3 | blue | RP11-654A16.3 | blue | HIST1H1B | cyan | KREMEN1 | darkgrey | CCDC83 | grey | LINC00472 | grey | PROKR2 | grey | ZMIZ1 | grey |
| GNG8 | blue | RP11-65L19.4 | blue | HIST1H2BD | cyan | KRT14 | darkgrey | CCDC84 | grey | LINC00477 | grey | PROSER2 | grey | ZMIZ1-AS1 | grey |
| GNGT1 | blue | RP11-661A12.9 | blue | HIST1H2BE | cyan | KRT18 | darkgrey | CCDC85A | grey | LINC00478 | grey | PROX1-AS1 | grey | ZNF10 | grey |
| GNGT2 | blue | RP11-663N22.1 | blue | HIST1H2BH | cyan | KRT23 | darkgrey | CCDC87 | grey | LINC00479 | grey | PROX2 | grey | ZNF100 | grey |
| GNL1 | blue | RP11-66N11.7 | blue | HIST1H2BK | cyan | KRT5 | darkgrey | CCDC88C | grey | LINC00483 | grey | PRPF18 | grey | ZNF132 | grey |
| GNRH2 | blue | RP11-66N11.8 | blue | HLA-C | cyan | KRT80 | darkgrey | CCDC89 | grey | LINC00520 | grey | PRPF19 | grey | ZNF133 | grey |
| GNRHR2 | blue | RP11-676J12.6 | blue | HLX | cyan | KRTCAP3 | darkgrey | CCDC90B | grey | LINC00525 | grey | PRPF3 | grey | ZNF134 | grey |
| GOLGA2 | blue | RP11-677O4.2 | blue | HMBOX1 | cyan | KSR1 | darkgrey | CCDC91 | grey | LINC00536 | grey | PRPF39 | grey | ZNF137P | grey |
| GOLGA7B | blue | RP11-67L3.4 | blue | HMGB1 | cyan | LACRT | darkgrey | CCDC96 | grey | LINC00540 | grey | PRPF40B | grey | ZNF138 | grey |
| GOLT1B | blue | RP11-686D22.8 | blue | HMGN1 | cyan | LAMA4 | darkgrey | CCDC97 | grey | LINC00558 | grey | PRPF8 | grey | ZNF140 | grey |
| GOPC | blue | RP11-690I21.2 | blue | HMGN2 | cyan | LARP1 | darkgrey | CCER1 | grey | LINC00559 | grey | PRPH | grey | ZNF154 | grey |
| GORAB | blue | RP11-700H6.1 | blue | HMGN3 | cyan | LCN1 | darkgrey | CCK | grey | LINC00560 | grey | PRPH2 | grey | ZNF157 | grey |
| GOT1 | blue | RP11-701I24.3 | blue | HMGN4 | cyan | LCN2 | darkgrey | CCKBR | grey | LINC00563 | grey | PRPS1L1 | grey | ZNF16 | grey |
| GP9 | blue | RP11-727F15.11 | blue | HNRNPA1 | cyan | LDLRAP1 | darkgrey | CCL11 | grey | LINC00572 | grey | PRPS2 | grey | ZNF175 | grey |
| GPALPP1 | blue | RP11-77H9.8 | blue | HNRNPA2B1 | cyan | LGALS1 | darkgrey | CCL13 | grey | LINC00574 | grey | PRPSAP1 | grey | ZNF18 | grey |
| GPATCH2 | blue | RP11-783K16.13 | blue | HNRNPA3 | cyan | LHFPL1 | darkgrey | CCL16 | grey | LINC00577 | grey | PRPSAP2 | grey | ZNF184 | grey |
| GPATCH3 | blue | RP11-792A8.4 | blue | HNRNPAB | cyan | LIMCH1 | darkgrey | CCL18 | grey | LINC00582 | grey | PRR11 | grey | ZNF185 | grey |
| GPBP1L1 | blue | RP11-793H13.11 | blue | HNRNPC | cyan | LINC00621 | darkgrey | CCL19 | grey | LINC00587 | grey | PRR12 | grey | ZNF197 | grey |
| GPC1 | blue | RP11-796E2.4 | blue | HNRNPD | cyan | LINC00948 | darkgrey | CCL2 | grey | LINC00589 | grey | PRR13 | grey | ZNF211 | grey |
| GPC2 | blue | RP11-79P5.2 | blue | HNRNPDL | cyan | LINC00960 | darkgrey | CCL20 | grey | LINC00592 | grey | PRR24 | grey | ZNF213 | grey |
| GPER1 | blue | RP11-805I24.3 | blue | HNRNPH1 | cyan | LMNA | darkgrey | CCL21 | grey | LINC00597 | grey | PRR3 | grey | ZNF213-AS1 | grey |
| GPHB5 | blue | RP11-83N9.5 | blue | HNRNPH3 | cyan | LMO3 | darkgrey | CCL23 | grey | LINC00605 | grey | PRR32 | grey | ZNF223 | grey |
| GPN3 | blue | RP11-843B15.2 | blue | HNRNPK | cyan | LMO4 | darkgrey | CCL25 | grey | LINC00607 | grey | PRR34-AS1 | grey | ZNF227 | grey |
| GPR123 | blue | RP11-845C23.3 | blue | HNRNPR | cyan | LNX2 | darkgrey | CCL4 | grey | LINC00612 | grey | PRR35 | grey | ZNF232 | grey |
| GPR135 | blue | RP11-847H18.2 | blue | HNRNPU | cyan | LOC100293211 | darkgrey | CCL7 | grey | LINC00615 | grey | PRR5 | grey | ZNF234 | grey |
| GPR144 | blue | RP11-85A1.3 | blue | HNRNPU-AS1 | cyan | LOC100506098 | darkgrey | CCL8 | grey | LINC00616 | grey | PRR7-AS1 | grey | ZNF239 | grey |
| GPR153 | blue | RP11-90P13.1 | blue | HNRNPUL1 | cyan | LOC100506100 | darkgrey | CCM2L | grey | LINC00619 | grey | PRR9 | grey | ZNF25 | grey |
| GPR161 | blue | RP11-930P14.2 | blue | HNRNPUL2 | cyan | LOC100507073 | darkgrey | CCNA1 | grey | LINC00622 | grey | PRRG1 | grey | ZNF250 | grey |
| GPR162 | blue | RP11-953B20.1 | blue | HOOK3 | cyan | LOC101927929 | darkgrey | CCNB3 | grey | LINC00623 | grey | PRRG3 | grey | ZNF251 | grey |
| GPR182 | blue | RP11-95D17.1 | blue | HOTTIP | cyan | LOC101928045 | darkgrey | CCND1 | grey | LINC00629 | grey | PRRT3 | grey | ZNF252P-AS1 | grey |
| GPR20 | blue | RP11-998D10.7 | blue | HOXB3 | cyan | LOC101929272 | darkgrey | CCNE2 | grey | LINC00632 | grey | PRRX2 | grey | ZNF256 | grey |
| GPR25 | blue | RP13-1032I1.7 | blue | HP1BP3 | cyan | LOC101929549 | darkgrey | CCNO | grey | LINC00639 | grey | PRSS21 | grey | ZNF266 | grey |
| GPR26 | blue | RP13-30A9.2 | blue | HPRT1 | cyan | LOC102723479 | darkgrey | CCNT2-AS1 | grey | LINC00645 | grey | PRSS33 | grey | ZNF267 | grey |
| GPR3 | blue | RP13-436F16.1 | blue | HR | cyan | LOC727916 | darkgrey | CCNYL2 | grey | LINC00648 | grey | PRSS35 | grey | ZNF280A | grey |
| GPR31 | blue | RP3-327A19.5 | blue | HRH3 | cyan | LOC93432 | darkgrey | CCR1 | grey | LINC00654 | grey | PRSS37 | grey | ZNF280C | grey |
| GPR32 | blue | RP3-329A5.8 | blue | HRK | cyan | LOXL2 | darkgrey | CCR3 | grey | LINC00656 | grey | PRSS3P2 | grey | ZNF283 | grey |
| GPR37L1 | blue | RP3-336K20__B.2 | blue | HS6ST3 | cyan | LPO | darkgrey | CCR4 | grey | LINC00665 | grey | PRSS55 | grey | ZNF284 | grey |
| GPR39 | blue | RP3-406C18.2 | blue | HSBP1 | cyan | LRBA | darkgrey | CCR5 | grey | LINC00668 | grey | PRTFDC1 | grey | ZNF295-AS1 | grey |
| GPR45 | blue | RP3-522P13.2 | blue | HSD17B11 | cyan | LRP1 | darkgrey | CCR6 | grey | LINC00673 | grey | PRUNE2 | grey | ZNF29P | grey |
| GPR61 | blue | RP4-555D20.3 | blue | HSDL2 | cyan | LRRC59 | darkgrey | CCR7 | grey | LINC00685 | grey | PSAP | grey | ZNF300 | grey |
| GPR62 | blue | RP4-560B9.4 | blue | HSP90AA1 | cyan | LSR | darkgrey | CCR8 | grey | LINC00698 | grey | PSAPL1 | grey | ZNF317 | grey |
| GPR68 | blue | RP4-593H12.1 | blue | HSP90AB1 | cyan | LTBP4 | darkgrey | CCRL2 | grey | LINC00705 | grey | PSCA | grey | ZNF318 | grey |
| GPR78 | blue | RP4-612B18.3 | blue | HSPA13 | cyan | LTF | darkgrey | CCRN4L | grey | LINC00824 | grey | PSD2 | grey | ZNF32 | grey |
| GPR97 | blue | RP4-676L2.1 | blue | HSPBAP1 | cyan | LY9 | darkgrey | CCSER1 | grey | LINC00838 | grey | PSENEN | grey | ZNF320 | grey |
| GPRC5C | blue | RP4-773N10.4 | blue | HSPE1 | cyan | LYZ | darkgrey | CCT4 | grey | LINC00842 | grey | PSG2 | grey | ZNF324 | grey |
| GPRIN1 | blue | RP4-781K5.2 | blue | HTATSF1 | cyan | LZTS3 | darkgrey | CCT5 | grey | LINC00845 | grey | PSG7 | grey | ZNF329 | grey |
| GPRIN2 | blue | RP4-794H19.1 | blue | HTR1A | cyan | MACC1 | darkgrey | CCT6B | grey | LINC00847 | grey | PSKH2 | grey | ZNF331 | grey |
| GPSM1 | blue | RP5-1031D4.2 | blue | HYDIN2 | cyan | MAL2 | darkgrey | CCT7 | grey | LINC00852 | grey | PSMA7 | grey | ZNF34 | grey |
| GPT | blue | RP5-1061H20.3 | blue | IARS | cyan | MANF | darkgrey | CCZ1 | grey | LINC00853 | grey | PSMB10 | grey | ZNF35 | grey |
| GPX5 | blue | RP5-1118D24.2 | blue | IARS2 | cyan | MANSC1 | darkgrey | CCZ1B | grey | LINC00856 | grey | PSMB3 | grey | ZNF354A | grey |
| GRAP | blue | RP5-1184F4.5 | blue | ICOSLG | cyan | MAPK13 | darkgrey | CD101 | grey | LINC00858 | grey | PSMB6 | grey | ZNF354B | grey |
| GRAP2 | blue | RP5-963E22.5 | blue | ID2 | cyan | MAPK6 | darkgrey | CD109 | grey | LINC00862 | grey | PSMB8 | grey | ZNF366 | grey |
| GRASP | blue | RP5-968J1.1 | blue | IDE | cyan | MCAM | darkgrey | CD14 | grey | LINC00865 | grey | PSMB8-AS1 | grey | ZNF367 | grey |
| GRIK1 | blue | RP6-91H8.2 | blue | IDH1 | cyan | MCFD2 | darkgrey | CD151 | grey | LINC00879 | grey | PSMB9 | grey | ZNF37BP | grey |
| GRIK1-AS1 | blue | RPA1 | blue | IER3IP1 | cyan | MCTP2 | darkgrey | CD160 | grey | LINC00886 | grey | PSMC1 | grey | ZNF385A | grey |
| GRIK4 | blue | RPE | blue | IFI16 | cyan | MEDAG | darkgrey | CD163L1 | grey | LINC00892 | grey | PSMC3 | grey | ZNF385B | grey |
| GRIK5 | blue | RPH3AL | blue | IFNGR1 | cyan | MEIS1 | darkgrey | CD177 | grey | LINC00896 | grey | PSMC3IP | grey | ZNF385C | grey |
| GRIN1 | blue | RPIA | blue | IFT22 | cyan | MEIS2 | darkgrey | CD180 | grey | LINC00905 | grey | PSMD11 | grey | ZNF394 | grey |
| GRIN2C | blue | RPL13AP17 | blue | IFT52 | cyan | MEN1 | darkgrey | CD19 | grey | LINC00909 | grey | PSMD2 | grey | ZNF415 | grey |
| GRIN3B | blue | RPL23AP53 | blue | IGDCC3 | cyan | MEOX1 | darkgrey | CD1A | grey | LINC00919 | grey | PSMD3 | grey | ZNF418 | grey |
| GRIP2 | blue | RPL34-AS1 | blue | IGF1R | cyan | METTL21B | darkgrey | CD1B | grey | LINC00920 | grey | PSMD7 | grey | ZNF430 | grey |
| GRIPAP1 | blue | RPL3L | blue | IGF2BP2 | cyan | MFSD4 | darkgrey | CD1C | grey | LINC00922 | grey | PSMG3 | grey | ZNF432 | grey |
| GRK1 | blue | RPLP1 | blue | IGF2R | cyan | MFSD6 | darkgrey | CD1E | grey | LINC00924 | grey | PSMG3-AS1 | grey | ZNF439 | grey |
| GRM2 | blue | RPP25 | blue | IGLL1 | cyan | MGAT4A | darkgrey | CD200R1 | grey | LINC00930 | grey | PSPH | grey | ZNF441 | grey |
| GRM4 | blue | RPPH1 | blue | IGSF10 | cyan | MICALL1 | darkgrey | CD207 | grey | LINC00935 | grey | PSTPIP1 | grey | ZNF442 | grey |
| GRM5 | blue | RPRML | blue | IGSF3 | cyan | MIEN1 | darkgrey | CD22 | grey | LINC00936 | grey | PSTPIP2 | grey | ZNF461 | grey |
| GRM8 | blue | RPS6KA3 | blue | IKBKB | cyan | MKNK2 | darkgrey | CD226 | grey | LINC00943 | grey | PTAFR | grey | ZNF462 | grey |
| GRPEL2 | blue | RPS6KA4 | blue | IKBKE | cyan | MLEC | darkgrey | CD244 | grey | LINC00951 | grey | PTCH2 | grey | ZNF468 | grey |
| GRWD1 | blue | RPS6KB2 | blue | IL13RA1 | cyan | MLPH | darkgrey | CD247 | grey | LINC00954 | grey | PTCHD4 | grey | ZNF470 | grey |
| GS1-124K5.9 | blue | RPS6KL1 | blue | IL1RN | cyan | MMP14 | darkgrey | CD28 | grey | LINC00957 | grey | PTCSC1 | grey | ZNF474 | grey |
| GS1-164F24.1 | blue | RPUSD1 | blue | IL21R-AS1 | cyan | MMP16 | darkgrey | CD33 | grey | LINC00959 | grey | PTGDS | grey | ZNF480 | grey |
| GSAP | blue | RPUSD2 | blue | IL6ST | cyan | MMP2 | darkgrey | CD37 | grey | LINC00961 | grey | PTGER2 | grey | ZNF483 | grey |
| GSC | blue | RRAD | blue | ILF3 | cyan | MMP28 | darkgrey | CD38 | grey | LINC00963 | grey | PTGER4 | grey | ZNF484 | grey |
| GSC2 | blue | RRN3 | blue | ILK | cyan | MOXD1 | darkgrey | CD3D | grey | LINC00965 | grey | PTGES | grey | ZNF486 | grey |
| GSDMC | blue | RRP1 | blue | IMPA1 | cyan | MPP5 | darkgrey | CD3E | grey | LINC00967 | grey | PTGFR | grey | ZNF488 | grey |
| GSDMD | blue | RRP15 | blue | IMPACT | cyan | MPP7 | darkgrey | CD3EAP | grey | LINC00968 | grey | PTGR1 | grey | ZNF490 | grey |
| GSG1 | blue | RRP7A | blue | IMPDH2 | cyan | MPZL2 | darkgrey | CD3G | grey | LINC00969 | grey | PTGR2 | grey | ZNF491 | grey |
| GSG1L | blue | RRP9 | blue | INPP5F | cyan | MRC2 | darkgrey | CD4 | grey | LINC00970 | grey | PTGS1 | grey | ZNF492 | grey |
| GSG2 | blue | RS1 | blue | INPP5J | cyan | MRGPRF | darkgrey | CD40LG | grey | LINC00993 | grey | PTGS2 | grey | ZNF501 | grey |
| GSK3A | blue | RSBN1 | blue | INSIG2 | cyan | MRPL40 | darkgrey | CD48 | grey | LINC01000 | grey | PTH1R | grey | ZNF503 | grey |
| GSX2 | blue | RSG1 | blue | INSM2 | cyan | MRPS33 | darkgrey | CD6 | grey | LINC01012 | grey | PTH2R | grey | ZNF503-AS1 | grey |
| GTF2H1 | blue | RSL24D1 | blue | INTS12 | cyan | MS4A1 | darkgrey | CD69 | grey | LINC01015 | grey | PTHLH | grey | ZNF513 | grey |
| GTF2H4 | blue | RSPH6A | blue | IPO5 | cyan | MSI2 | darkgrey | CD72 | grey | LINC01016 | grey | PTMS | grey | ZNF514 | grey |
| GTF2IRD2 | blue | RSPO1 | blue | IPO7 | cyan | MSMB | darkgrey | CD74 | grey | LINC01018 | grey | PTN | grey | ZNF518B | grey |
| GTF3C1 | blue | RSPO4 | blue | IPO8 | cyan | MUC15 | darkgrey | CD81-AS1 | grey | LINC01019 | grey | PTOV1 | grey | ZNF526 | grey |
| GTF3C4 | blue | RSPRY1 | blue | IQCB1 | cyan | MUC5B | darkgrey | CD83 | grey | LINC01049 | grey | PTPLA | grey | ZNF527 | grey |
| GTPBP10 | blue | RTCA | blue | IQCF5 | cyan | MUCL1 | darkgrey | CD84 | grey | LINC01057 | grey | PTPN13 | grey | ZNF528 | grey |
| GTPBP6 | blue | RTEL1 | blue | IQCK | cyan | MXRA8 | darkgrey | CD86 | grey | LINC01069 | grey | PTPN3 | grey | ZNF530 | grey |
| GTSE1 | blue | RTFDC1 | blue | IQGAP1 | cyan | MYO5B | darkgrey | CD8A | grey | LINC01085 | grey | PTPN6 | grey | ZNF541 | grey |
| GUCA1A | blue | RTKN | blue | IQGAP3 | cyan | MYO7A | darkgrey | CD9 | grey | LINC01091 | grey | PTPN7 | grey | ZNF542P | grey |
| GUCA1B | blue | RTN4R | blue | IRF2 | cyan | NANS | darkgrey | CD97 | grey | LINC01094 | grey | PTPRC | grey | ZNF549 | grey |
| GUCA2A | blue | RTN4RL1 | blue | IRF2BP2 | cyan | NAPA | darkgrey | CD99P1 | grey | LINC01098 | grey | PTPRCAP | grey | ZNF552 | grey |
| GUCA2B | blue | RTP5 | blue | IRF2BPL | cyan | NCALD | darkgrey | CDA | grey | LINC01102 | grey | PTPRD | grey | ZNF557 | grey |
| GUCY2C | blue | RUNDC3A | blue | IRX4 | cyan | NDRG2 | darkgrey | CDC25B | grey | LINC01114 | grey | PTPRF | grey | ZNF563 | grey |
| GUCY2D | blue | RUNX1-IT1 | blue | ISCA1 | cyan | NDUFB3 | darkgrey | CDC37 | grey | LINC01115 | grey | PTPRN | grey | ZNF564 | grey |
| GUCY2F | blue | RUSC2 | blue | ISCU | cyan | NEBL | darkgrey | CDC42-IT1 | grey | LINC01116 | grey | PTPRN2 | grey | ZNF565 | grey |
| GZMM | blue | RWDD1 | blue | ITFG1 | cyan | NEDD4L | darkgrey | CDC42BPA | grey | LINC01119 | grey | PTPRT | grey | ZNF566 | grey |
| H1FNT | blue | RYBP | blue | ITGA6 | cyan | NES | darkgrey | CDC42BPB | grey | LINC01122 | grey | PTPRU | grey | ZNF57 | grey |
| H1FOO | blue | S100A3 | blue | ITGAV | cyan | NEURL1B | darkgrey | CDC42EP1 | grey | LINC01125 | grey | PTS | grey | ZNF570 | grey |
| H2AFX | blue | S100A5 | blue | ITGB1 | cyan | NFATC1 | darkgrey | CDC42EP4 | grey | LINC01127 | grey | PTTG1 | grey | ZNF571-AS1 | grey |
| H2BFM | blue | S100A7A | blue | ITLN1 | cyan | NFKBIZ | darkgrey | CDC42EP5 | grey | LINC01133 | grey | PTTG2 | grey | ZNF572 | grey |
| HAB1 | blue | S100P | blue | ITM2A | cyan | NOSTRIN | darkgrey | CDCA4 | grey | LINC01136 | grey | PTTG3P | grey | ZNF574 | grey |
| HABP4 | blue | S1PR2 | blue | ITM2B | cyan | NOTCH4 | darkgrey | CDCA7L | grey | LINC01139 | grey | PTX3 | grey | ZNF575 | grey |
| HAGHL | blue | S1PR4 | blue | IWS1 | cyan | NPR2 | darkgrey | CDCA8 | grey | LINC01146 | grey | PUS3 | grey | ZNF577 | grey |
| HAGLROS | blue | S1PR5 | blue | JAGN1 | cyan | NRP2 | darkgrey | CDH10 | grey | LINC01153 | grey | PUS7 | grey | ZNF580 | grey |
| HAMP | blue | SAA3P | blue | JAK1 | cyan | NSUN7 | darkgrey | CDH11 | grey | LINC01158 | grey | PUSL1 | grey | ZNF581 | grey |
| HAND2 | blue | SACS | blue | JAK2 | cyan | NT5C2 | darkgrey | CDH12 | grey | LINC01165 | grey | PVALB | grey | ZNF587 | grey |
| HAO2 | blue | SAMD1 | blue | JAZF1 | cyan | NUCB2 | darkgrey | CDH13 | grey | LINC01186 | grey | PVRIG | grey | ZNF593 | grey |
| HAPLN2 | blue | SAMD10 | blue | JKAMP | cyan | NUS1 | darkgrey | CDH18 | grey | LINC01197 | grey | PVRL3 | grey | ZNF595 | grey |
| HAS1 | blue | SAMD11 | blue | JMJD1C | cyan | NUS1P3 | darkgrey | CDH19 | grey | LINC01207 | grey | PVRL3-AS1 | grey | ZNF606 | grey |
| HAUS2 | blue | SAMD14 | blue | KANK3 | cyan | NXPH3 | darkgrey | CDH20 | grey | LINC01214 | grey | PXMP2 | grey | ZNF607 | grey |
| HAUS6 | blue | SAMM50 | blue | KANSL2 | cyan | OAS2 | darkgrey | CDH24 | grey | LINC01220 | grey | PXN-AS1 | grey | ZNF610 | grey |
| HAVCR1P1 | blue | SAPCD2 | blue | KARS | cyan | OCIAD2 | darkgrey | CDH3 | grey | LINC01222 | grey | PXYLP1 | grey | ZNF611 | grey |
| HBM | blue | SARDH | blue | KAT6A | cyan | OCLN | darkgrey | CDIP1 | grey | LINC01225 | grey | PYCARD | grey | ZNF613 | grey |
| HBZ | blue | SARS | blue | KAT6B | cyan | ODAM | darkgrey | CDIPT-AS1 | grey | LINC01231 | grey | PYCARDOS | grey | ZNF618 | grey |
| HCFC1 | blue | SAT1 | blue | KAT7 | cyan | ORMDL2 | darkgrey | CDK5 | grey | LINC01234 | grey | PYCRL | grey | ZNF622 | grey |
| HCG9 | blue | SBF1 | blue | KBTBD2 | cyan | OSBP | darkgrey | CDK5RAP3 | grey | LINC01252 | grey | PYGB | grey | ZNF628 | grey |
| HCN2 | blue | SBK1 | blue | KCNC1 | cyan | P4HB | darkgrey | CDKL1 | grey | LINC01255 | grey | PYGM | grey | ZNF629 | grey |
| HCN3 | blue | SCAF4 | blue | KCNE1 | cyan | PADI2 | darkgrey | CDKN1A | grey | LINC01278 | grey | PZP | grey | ZNF630 | grey |
| HCN4 | blue | SCAF8 | blue | KCNJ10 | cyan | PAG1 | darkgrey | CDKN2C | grey | LINC01281 | grey | QDPR | grey | ZNF638 | grey |
| HCP5B | blue | SCAND1 | blue | KCNK10 | cyan | PAIP2B | darkgrey | CDKN3 | grey | LINC01289 | grey | QPCT | grey | ZNF645 | grey |
| HCRT | blue | SCAP | blue | KCNK12 | cyan | PALM | darkgrey | CDNF | grey | LINC01293 | grey | QRFPR | grey | ZNF649 | grey |
| HDAC10 | blue | SCARF2 | blue | KCNK5 | cyan | PAQR7 | darkgrey | CDPF1 | grey | LINC01300 | grey | QTRT1 | grey | ZNF658 | grey |
| HDAC11 | blue | SCGN | blue | KCNS2 | cyan | PARP6 | darkgrey | CDR1 | grey | LINC01305 | grey | R3HCC1 | grey | ZNF662 | grey |
| HDAC6 | blue | SCN2B | blue | KCTD12 | cyan | PARVB | darkgrey | CDR2L | grey | LINC01312 | grey | RAB11B-AS1 | grey | ZNF663P | grey |
| HDDC2 | blue | SCN3A | blue | KCTD13 | cyan | PAX6 | darkgrey | CDSN | grey | LINC01315 | grey | RAB11FIP3 | grey | ZNF665 | grey |
| HDGFRP2 | blue | SCNN1B | blue | KCTD6 | cyan | PCSK5 | darkgrey | CEACAM19 | grey | LINC01343 | grey | RAB11FIP5 | grey | ZNF669 | grey |
| HDHD3 | blue | SCNN1D | blue | KDELC2 | cyan | PDE4B | darkgrey | CEACAM21 | grey | LINC01346 | grey | RAB13 | grey | ZNF680 | grey |
| HEATR5A | blue | SCP2D1 | blue | KDELR2 | cyan | PDGFRB | darkgrey | CEBPB | grey | LINC01354 | grey | RAB1B | grey | ZNF684 | grey |
| HELZ2 | blue | SCRN2 | blue | KDELR3 | cyan | PDIA3 | darkgrey | CEBPD | grey | LINC01355 | grey | RAB33A | grey | ZNF700 | grey |
| HERC1 | blue | SCRT1 | blue | KDM2A | cyan | PDIA6 | darkgrey | CEBPG | grey | LINC01356 | grey | RAB36 | grey | ZNF701 | grey |
| HERC4 | blue | SCT | blue | KDM3B | cyan | PDK1 | darkgrey | CECR3 | grey | LINC01359 | grey | RAB37 | grey | ZNF703 | grey |
| HERPUD2 | blue | SCUBE1 | blue | KDSR | cyan | PDK3 | darkgrey | CECR5 | grey | LINC01362 | grey | RAB38 | grey | ZNF708 | grey |
| HES2 | blue | SCYL1 | blue | KHDRBS1 | cyan | PDZK1IP1 | darkgrey | CECR6 | grey | LINC01364 | grey | RAB3A | grey | ZNF711 | grey |
| HES4 | blue | SCYL2 | blue | KHNYN | cyan | PERP | darkgrey | CECR7 | grey | LINC01365 | grey | RAB3IL1 | grey | ZNF713 | grey |
| HES5 | blue | SDE2 | blue | KIAA0100 | cyan | PEX7 | darkgrey | CELF2-AS1 | grey | LINC01395 | grey | RAB43 | grey | ZNF717 | grey |
| HES6 | blue | SDHC | blue | KIAA0430 | cyan | PGAP2 | darkgrey | CELF6 | grey | LINC01398 | grey | RAB4B | grey | ZNF721 | grey |
| HES7 | blue | SDHD | blue | KIAA0825 | cyan | PGM3 | darkgrey | CELSR3-AS1 | grey | LINC01424 | grey | RAB6A | grey | ZNF732 | grey |
| HEXDC | blue | SDSL | blue | KIAA0922 | cyan | PGM5-AS1 | darkgrey | CENPB | grey | LINC01425 | grey | RAB7A | grey | ZNF749 | grey |
| HEXIM2 | blue | SEC14L2 | blue | KIAA1033 | cyan | PHLDA1 | darkgrey | CENPC | grey | LINC01426 | grey | RAB7B | grey | ZNF75A | grey |
| HGC6.3 | blue | SEC14L3 | blue | KIAA1045 | cyan | PHLPP1 | darkgrey | CENPH | grey | LINC01431 | grey | RAB9B | grey | ZNF76 | grey |
| HGFAC | blue | SEC14L4 | blue | KIAA1143 | cyan | PIGR | darkgrey | CENPL | grey | LINC01432 | grey | RAB9BP1 | grey | ZNF763 | grey |
| HHLA1 | blue | SEC1P | blue | KIAA1191 | cyan | PIGX | darkgrey | CENPQ | grey | LINC01442 | grey | RABAC1 | grey | ZNF766 | grey |
| HIATL1 | blue | SEC23IP | blue | KIAA1211L | cyan | PIK3AP1 | darkgrey | CENPV | grey | LINC01448 | grey | RABGEF1 | grey | ZNF767P | grey |
| HIC2 | blue | SEMA3B | blue | KIAA1432 | cyan | PIP | darkgrey | CENPW | grey | LINC01449 | grey | RABGGTB | grey | ZNF776 | grey |
| HIGD1B | blue | SEMA3F | blue | KIAA1551 | cyan | PIP5K1B | darkgrey | CEP164 | grey | LINGO2 | grey | RABIF | grey | ZNF778 | grey |
| HILS1 | blue | SEMA4C | blue | KIAA1598 | cyan | PIR | darkgrey | CEP19 | grey | LIPC | grey | RAC2 | grey | ZNF786 | grey |
| HIRIP3 | blue | SEMA6B | blue | KIAA2026 | cyan | PITPNA | darkgrey | CEP192 | grey | LIPF | grey | RACGAP1 | grey | ZNF791 | grey |
| HIST1H1T | blue | SEMA6C | blue | KIF18B | cyan | PKNOX2 | darkgrey | CEP290 | grey | LL0XNC01-116E7.2 | grey | RAD18 | grey | ZNF804A | grey |
| HIST1H2AB | blue | SEMA7A | blue | KIF26B | cyan | PKP2 | darkgrey | CEP72 | grey | LL0XNC01-7P3.1 | grey | RAD51-AS1 | grey | ZNF808 | grey |
| HIST1H2BB | blue | SENP6 | blue | KIF5B | cyan | PKP4 | darkgrey | CER1 | grey | LL22NC03-N14H11.1 | grey | RAD51C | grey | ZNF823 | grey |
| HIST1H2BJ | blue | SEPT5-GP1BB | blue | KISS1R | cyan | PLA2R1 | darkgrey | CERCAM | grey | LMAN1L | grey | RADIL | grey | ZNF84 | grey |
| HIST1H2BL | blue | SERPINA10 | blue | KLF12 | cyan | PLAC9 | darkgrey | CERK | grey | LMAN2L | grey | RALBP1 | grey | ZNF844 | grey |
| HIST1H4G | blue | SERPINA2 | blue | KLF3 | cyan | PLCB1 | darkgrey | CERS2 | grey | LMBR1L | grey | RALGDS | grey | ZNF85 | grey |
| HIST1H4I | blue | SERPINA4 | blue | KLHDC10 | cyan | PLEKHH1 | darkgrey | CERS3 | grey | LMBRD2 | grey | RALGPS2 | grey | ZNF850 | grey |
| HIST3H2A | blue | SERPINA6 | blue | KLHDC2 | cyan | PLEKHS1 | darkgrey | CES5A | grey | LMCD1 | grey | RALY | grey | ZNF862 | grey |
| HIST3H3 | blue | SERPINF2 | blue | KLHL7 | cyan | PLLP | darkgrey | CETN1 | grey | LMNB2 | grey | RALY-AS1 | grey | ZNF876P | grey |
| HK1 | blue | SERTAD2 | blue | KLHL9 | cyan | PLXNA2 | darkgrey | CFC1B | grey | LMNTD1 | grey | RALYL | grey | ZNF879 | grey |
| HK3 | blue | SETD1A | blue | KLK5 | cyan | PLXND1 | darkgrey | CFHR2 | grey | LMO7DN | grey | RAMP2-AS1 | grey | ZNF883 | grey |
| HKDC1 | blue | SETD5 | blue | KLK6 | cyan | PNOC | darkgrey | CFI | grey | LMOD1 | grey | RAMP3 | grey | ZNFX1 | grey |
| HLCS | blue | SEZ6 | blue | KMT2A | cyan | PODN | darkgrey | CFP | grey | LMX1B | grey | RANBP3L | grey | ZNHIT3 | grey |
| HLTF | blue | SFI1 | blue | KMT2C | cyan | POLR2C | darkgrey | CGRRF1 | grey | LNP1 | grey | RANBP6 | grey | ZNRF2P1 | grey |
| HMBS | blue | SFRP5 | blue | KMT2D | cyan | PON3 | darkgrey | CH25H | grey | LNX1 | grey | RAP2C-AS1 | grey | ZNRF3 | grey |
| HMCES | blue | SFTA1P | blue | KMT2E | cyan | PPAP2C | darkgrey | CHAC1 | grey | LOC100049716 | grey | RAPGEF3 | grey | ZP2 | grey |
| HMCN2 | blue | SFTA2 | blue | KNG1 | cyan | PPAPDC1B | darkgrey | CHAC2 | grey | LOC100127940 | grey | RAPGEF5 | grey | ZP4 | grey |
| HMGB4 | blue | SFTPC | blue | KPNA1 | cyan | PPAPDC2 | darkgrey | CHAD | grey | LOC100127972 | grey | RAPGEFL1 | grey | ZPBP | grey |
| HMX1 | blue | SFXN4 | blue | KPNA2 | cyan | PPIL1 | darkgrey | CHAMP1 | grey | LOC100128175 | grey | RARA | grey | ZPBP2 | grey |
| HNF1A | blue | SGCA | blue | KPNA3 | cyan | PPM1H | darkgrey | CHCHD1 | grey | LOC100128185 | grey | RARRES3 | grey | ZPR1 | grey |
| HNF4A | blue | SGMS1 | blue | KPNA4 | cyan | PPM1K | darkgrey | CHCHD4 | grey | LOC100128198 | grey | RARS | grey | ZSCAN16 | grey |
| HNF4A-AS1 | blue | SGPP1 | blue | KPNA6 | cyan | PPM1L | darkgrey | CHD3 | grey | LOC100128288 | grey | RASD2 | grey | ZSCAN21 | grey |
| HNRNPL | blue | SH2B1 | blue | KPNB1 | cyan | PPP1CA | darkgrey | CHD8 | grey | LOC100128554 | grey | RASGEF1A | grey | ZSCAN22 | grey |
| HNRNPLL | blue | SH2D1A | blue | KRT40 | cyan | PPP1R16A | darkgrey | CHDC2 | grey | LOC100128751 | grey | RASGEF1B | grey | ZSCAN26 | grey |
| HOMER3 | blue | SH2D3A | blue | KRT77 | cyan | PPP1R1B | darkgrey | CHEK2 | grey | LOC100128993 | grey | RASGRF2 | grey | ZSCAN31 | grey |
| HOXA-AS2 | blue | SH2D5 | blue | KTN1 | cyan | PPP1R3B | darkgrey | CHI3L1 | grey | LOC100129034 | grey | RASGRP1 | grey | ZSCAN32 | grey |
| HOXA-AS3 | blue | SH3BP1 | blue | KY | cyan | PPP2R3A | darkgrey | CHIAP2 | grey | LOC100129069 | grey | RASL10A | grey | ZSWIM5 | grey |
| HOXA11 | blue | SH3BP5L | blue | L1TD1 | cyan | PRDM1 | darkgrey | CHKB | grey | LOC100129098 | grey | RASL11A | grey | ZXDA | grey |
| HOXA11-AS | blue | SH3D21 | blue | LAGE3 | cyan | PRDM16 | darkgrey | CHL1 | grey | LOC100129198 | grey | RASL11B | grey | ZYX | grey |
| HOXA3 | blue | SH3GL1P2 | blue | LAMP2 | cyan | PREB | darkgrey | CHMP1B | grey | LOC100129380 | grey | RASL12 | grey | ZZZ3 | grey |
| HOXA4 | blue | SHANK1 | blue | LAMTOR3 | cyan | PRELID1 | darkgrey | CHMP2A | grey | LOC100129447 | grey | RASSF1 | grey | ACER3 | midnightblue |
| HOXA6 | blue | SHANK2-AS3 | blue | LAMTOR5 | cyan | PRKACB | darkgrey | CHMP7 | grey | LOC100129455 | grey | RASSF4 | grey | ACSL5 | midnightblue |
| HOXA7 | blue | SHBG | blue | LANCL1 | cyan | PRKCDBP | darkgrey | CHN1 | grey | LOC100129461 | grey | RASSF8-AS1 | grey | ADARB2 | midnightblue |
| HOXB-AS1 | blue | SHC2 | blue | LAP3 | cyan | PROL1 | darkgrey | CHODL | grey | LOC100129550 | grey | RASSF9 | grey | AGFG2 | midnightblue |
| HOXB-AS3 | blue | SHCBP1L | blue | LAPTM4A | cyan | PROM1 | darkgrey | CHPF | grey | LOC100129603 | grey | RBBP7 | grey | ALDH3B2 | midnightblue |
| HOXB1 | blue | SHD | blue | LAPTM4B | cyan | PRR15L | darkgrey | CHRM2 | grey | LOC100129620 | grey | RBM10 | grey | ALOX15B | midnightblue |
| HOXB13 | blue | SHF | blue | LARP4 | cyan | PRSS16 | darkgrey | CHRM3-AS2 | grey | LOC100129884 | grey | RBM12B | grey | ANTXR2 | midnightblue |
| HOXB4 | blue | SHFM1 | blue | LARS | cyan | PRSS8 | darkgrey | CHRM5 | grey | LOC100129917 | grey | RBM12B-AS1 | grey | AP1M2 | midnightblue |
| HOXB5 | blue | SHH | blue | LARS2 | cyan | PSD4 | darkgrey | CHRNA5 | grey | LOC100130078 | grey | RBM14 | grey | APCDD1L | midnightblue |
| HOXB8 | blue | SHISA4 | blue | LASP1 | cyan | PSME4 | darkgrey | CHST10 | grey | LOC100130111 | grey | RBM19 | grey | ARHGAP29 | midnightblue |
| HOXB9 | blue | SHQ1 | blue | LATS1 | cyan | PTDSS1 | darkgrey | CHST11 | grey | LOC100130219 | grey | RBM20 | grey | ARHGEF5 | midnightblue |
| HOXC-AS3 | blue | SHROOM1 | blue | LBR | cyan | PTK7 | darkgrey | CHST15 | grey | LOC100130264 | grey | RBM23 | grey | ATP2B2 | midnightblue |
| HOXC12 | blue | SIAH1 | blue | LDB3 | cyan | PTPN21 | darkgrey | CHST2 | grey | LOC100130278 | grey | RBM24 | grey | AZGP1P1 | midnightblue |
| HOXC5 | blue | SIGLEC11 | blue | LDHA | cyan | PTPRJ | darkgrey | CHST4 | grey | LOC100130331 | grey | RBM28 | grey | B3GALT5 | midnightblue |
| HOXD10 | blue | SIGLEC7 | blue | LDHB | cyan | PTPRS | darkgrey | CHST7 | grey | LOC100130357 | grey | RBM34 | grey | B4GALT3 | midnightblue |
| HOXD11 | blue | SIGLEC8 | blue | LDLRAD3 | cyan | PTRF | darkgrey | CHSY3 | grey | LOC100130429 | grey | RBM41 | grey | BAIAP2L1 | midnightblue |
| HOXD12 | blue | SIK3 | blue | LDOC1 | cyan | PTRHD1 | darkgrey | CHUK | grey | LOC100130456 | grey | RBM42 | grey | BCAP29 | midnightblue |
| HOXD13 | blue | SIM2 | blue | LDOC1L | cyan | PXDN | darkgrey | CIART | grey | LOC100130458 | grey | RBMX2 | grey | BDH1 | midnightblue |
| HOXD9 | blue | SIPA1 | blue | LECT1 | cyan | PXN | darkgrey | CIB4 | grey | LOC100130476 | grey | RBMXL1 | grey | C10orf128 | midnightblue |
| HP08942 | blue | SIRT1 | blue | LEFTY1 | cyan | R3HDM4 | darkgrey | CIDEB | grey | LOC100130642 | grey | RBP5 | grey | C19orf10 | midnightblue |
| HPCA | blue | SIRT2 | blue | LENG8 | cyan | RAB15 | darkgrey | CILP | grey | LOC100130691 | grey | RBP7 | grey | C5orf27 | midnightblue |
| HPCAL4 | blue | SIRT6 | blue | LEPROT | cyan | RAB27B | darkgrey | CILP2 | grey | LOC100130872 | grey | RBSG3 | grey | C6orf132 | midnightblue |
| HPDL | blue | SIT1 | blue | LETMD1 | cyan | RAP1GAP2 | darkgrey | CIPC | grey | LOC100130987 | grey | RBX1 | grey | CA5B | midnightblue |
| HPN | blue | SIVA1 | blue | LGALSL | cyan | RARRES2 | darkgrey | CIRBP | grey | LOC100131043 | grey | RCAN1 | grey | CALML5 | midnightblue |
| HPS3 | blue | SIX3 | blue | LHFPL4 | cyan | RASA3 | darkgrey | CISD1 | grey | LOC100131496 | grey | RCBTB2 | grey | CAMK2N1 | midnightblue |
| HPS6 | blue | SIX3-AS1 | blue | LIFR | cyan | RASEF | darkgrey | CISH | grey | LOC100131510 | grey | RCC2 | grey | CAPN12 | midnightblue |
| HPX | blue | SKIV2L | blue | LIMA1 | cyan | RASSF10 | darkgrey | CITED2 | grey | LOC100131541 | grey | RCCD1 | grey | CAPS | midnightblue |
| HRASLS2 | blue | SKIV2L2 | blue | LIN28B | cyan | RASSF5 | darkgrey | CKAP2L | grey | LOC100131564 | grey | RCSD1 | grey | CASZ1 | midnightblue |
| HRH2 | blue | SLAMF9 | blue | LIN7C | cyan | RASSF6 | darkgrey | CKB | grey | LOC100131581 | grey | RCVRN | grey | CBS | midnightblue |
| HS2ST1 | blue | SLC11A1 | blue | LINC-PINT | cyan | RBM47 | darkgrey | CKLF | grey | LOC100131655 | grey | RDH12 | grey | CCDC178 | midnightblue |
| HS3ST1 | blue | SLC12A3 | blue | LINC00301 | cyan | RBP1 | darkgrey | CKM | grey | LOC100131756 | grey | RDH8 | grey | CCDC50 | midnightblue |
| HS3ST5 | blue | SLC12A4 | blue | LINC00302 | cyan | RBPMS2 | darkgrey | CKMT2 | grey | LOC100131763 | grey | RDM1 | grey | CCDC85C | midnightblue |
| HS3ST6 | blue | SLC12A6 | blue | LINC00342 | cyan | RCAN3 | darkgrey | CKS1B | grey | LOC100132005 | grey | REC114 | grey | CD200 | midnightblue |
| HS6ST1 | blue | SLC12A9 | blue | LINC00354 | cyan | RCN1 | darkgrey | CKS2 | grey | LOC100132077 | grey | REEP1 | grey | CDC42BPG | midnightblue |
| HSD11B1L | blue | SLC13A4 | blue | LINC00454 | cyan | RCN3 | darkgrey | CLASP1 | grey | LOC100132078 | grey | REG1A | grey | CDCP1 | midnightblue |
| HSD17B1 | blue | SLC13A5 | blue | LINC00528 | cyan | REPS1 | darkgrey | CLC | grey | LOC100132167 | grey | REG1B | grey | CDK18 | midnightblue |
| HSD17B14 | blue | SLC16A11 | blue | LINC00529 | cyan | REPS2 | darkgrey | CLCA1 | grey | LOC100132319 | grey | REG1P | grey | CELF2 | midnightblue |
| HSD17B4 | blue | SLC16A5 | blue | LINC00535 | cyan | RGCC | darkgrey | CLCA4 | grey | LOC100132352 | grey | REG4 | grey | CGN | midnightblue |
| HSF2 | blue | SLC16A8 | blue | LINC00545 | cyan | RGMB | darkgrey | CLCN6 | grey | LOC100132354 | grey | RELA | grey | CHDH | midnightblue |
| HSF4 | blue | SLC18A3 | blue | LINC00555 | cyan | RHOB | darkgrey | CLDN10-AS1 | grey | LOC100132356 | grey | RELB | grey | CHKA | midnightblue |
| HSPA14 | blue | SLC19A1 | blue | LINC00561 | cyan | RMST | darkgrey | CLDN11 | grey | LOC100132661 | grey | RELL1 | grey | CHPF2 | midnightblue |
| HSPA9 | blue | SLC1A7 | blue | LINC00593 | cyan | RNF128 | darkgrey | CLDN12 | grey | LOC100132686 | grey | RELL2 | grey | CHST1 | midnightblue |
| HSPB11 | blue | SLC22A1 | blue | LINC00641 | cyan | RNF144B | darkgrey | CLDN16 | grey | LOC100132735 | grey | RELN | grey | CLDN3 | midnightblue |
| HSPB3 | blue | SLC22A12 | blue | LINC00657 | cyan | RNF152 | darkgrey | CLDN17 | grey | LOC100132891 | grey | RELT | grey | CLDN7 | midnightblue |
| HSPB9 | blue | SLC22A18AS | blue | LINC00674 | cyan | RNF39 | darkgrey | CLDN2 | grey | LOC100133039 | grey | REM2 | grey | CLDN8 | midnightblue |
| HSPBP1 | blue | SLC22A23 | blue | LINC00708 | cyan | ROBO4 | darkgrey | CLDN20 | grey | LOC100133089 | grey | REN | grey | COL15A1 | midnightblue |
| HSPC081 | blue | SLC22A31 | blue | LINC00841 | cyan | RP11-532F12.5 | darkgrey | CLEC2B | grey | LOC100133130 | grey | RENBP | grey | COL17A1 | midnightblue |
| HSPH1 | blue | SLC22A6 | blue | LINC00882 | cyan | RP11-736K20.5 | darkgrey | CLEC4G | grey | LOC100133315 | grey | REPIN1 | grey | CPNE8 | midnightblue |
| HTR1D | blue | SLC22A7 | blue | LINC00884 | cyan | RP11-747H7.3 | darkgrey | CLEC4GP1 | grey | LOC100133669 | grey | RERGL | grey | CTNNBIP1 | midnightblue |
| HTR4 | blue | SLC22A8 | blue | LINC00893 | cyan | RPN1 | darkgrey | CLEC5A | grey | LOC100133985 | grey | REST | grey | CTTNBP2NL | midnightblue |
| HTR5A | blue | SLC25A10 | blue | LINC00950 | cyan | RRAGD | darkgrey | CLEC7A | grey | LOC100134368 | grey | REV1 | grey | DBNDD1 | midnightblue |
| HTR6 | blue | SLC25A13 | blue | LINC00958 | cyan | RWDD2A | darkgrey | CLECL1 | grey | LOC100134937 | grey | REXO1L1P | grey | DCAF11 | midnightblue |
| HTR7 | blue | SLC25A19 | blue | LINC01007 | cyan | S100A1 | darkgrey | CLHC1 | grey | LOC100271832 | grey | RFC3 | grey | DCD | midnightblue |
| HTT | blue | SLC25A26 | blue | LINC01013 | cyan | SATB1 | darkgrey | CLIC1 | grey | LOC100271840 | grey | RFK | grey | DERL3 | midnightblue |
| HUS1 | blue | SLC25A32 | blue | LINC01206 | cyan | SCD5 | darkgrey | CLIC3 | grey | LOC100272216 | grey | RFNG | grey | DLK1 | midnightblue |
| HUS1B | blue | SLC25A34 | blue | LINC01233 | cyan | SCGB1D1 | darkgrey | CLIP2 | grey | LOC100286922 | grey | RFPL2 | grey | DOCK11 | midnightblue |
| HYAL2 | blue | SLC25A4 | blue | LINC01268 | cyan | SCGB1D2 | darkgrey | CLIP3 | grey | LOC100287290 | grey | RFWD3 | grey | DOCK4 | midnightblue |
| HYAL3 | blue | SLC25A41 | blue | LINC01272 | cyan | SCGB2A1 | darkgrey | CLK2 | grey | LOC100287590 | grey | RGAG1 | grey | DSEL | midnightblue |
| HYPM | blue | SLC25A42 | blue | LINC01282 | cyan | SCGB2A2 | darkgrey | CLN3 | grey | LOC100287896 | grey | RGL2 | grey | EDNRA | midnightblue |
| IAH1 | blue | SLC25A44 | blue | LINC01310 | cyan | SDC1 | darkgrey | CLPP | grey | LOC100288152 | grey | RGMA | grey | EIF4G3 | midnightblue |
| IBA57 | blue | SLC25A47 | blue | LINC01420 | cyan | SDC4 | darkgrey | CLPTM1 | grey | LOC100288181 | grey | RGN | grey | EPB41L3 | midnightblue |
| IBSP | blue | SLC25A48 | blue | LIPA | cyan | SEC11C | darkgrey | CLRN3 | grey | LOC100288310 | grey | RGPD4-AS1 | grey | EPHB3 | midnightblue |
| IBTK | blue | SLC25A5-AS1 | blue | LIPT1 | cyan | SEC16A | darkgrey | CLSTN1 | grey | LOC100288570 | grey | RGS1 | grey | EPS8L2 | midnightblue |
| ICAM5 | blue | SLC26A1 | blue | LITAF | cyan | SEC23B | darkgrey | CLSTN2 | grey | LOC100288675 | grey | RGS10 | grey | ERBB2 | midnightblue |
| ICE1 | blue | SLC26A10 | blue | LIX1L | cyan | SEC61A1 | darkgrey | CLSTN2-AS1 | grey | LOC100288721 | grey | RGS11 | grey | ERBB4 | midnightblue |
| ICE2 | blue | SLC27A4 | blue | LMAN1 | cyan | SEC61B | darkgrey | CLTB | grey | LOC100288860 | grey | RGS18 | grey | EVPL | midnightblue |
| ICK | blue | SLC27A5 | blue | LMAN2 | cyan | SEC61G | darkgrey | CLTC-IT1 | grey | LOC100288911 | grey | RGS19 | grey | F5 | midnightblue |
| IDH1-AS1 | blue | SLC28A1 | blue | LNPEP | cyan | SEL1L3 | darkgrey | CLTCL1 | grey | LOC100288966 | grey | RGS2 | grey | FAM195A | midnightblue |
| IDH3A | blue | SLC28A2 | blue | LOC100129129 | cyan | SELK | darkgrey | CLU | grey | LOC100289045 | grey | RGS22 | grey | FAM222A | midnightblue |
| IDUA | blue | SLC29A2 | blue | LOC100130548 | cyan | SEMA6A | darkgrey | CLUL1 | grey | LOC100289098 | grey | RGS3 | grey | FAM3D | midnightblue |
| IER5L | blue | SLC2A11 | blue | LOC100131053 | cyan | SERP1 | darkgrey | CLVS1 | grey | LOC100289333 | grey | RGS7BP | grey | FAM46B | midnightblue |
| IFI27L2 | blue | SLC2A5 | blue | LOC100131303 | cyan | SERPINA3 | darkgrey | CLYBL-AS1 | grey | LOC100289473 | grey | RGS8 | grey | FAM83A | midnightblue |
| IFI35 | blue | SLC2A6 | blue | LOC100134445 | cyan | SFT2D1 | darkgrey | CMA1 | grey | LOC100289495 | grey | RGS9 | grey | FAM83H | midnightblue |
| IFIH1 | blue | SLC2A8 | blue | LOC100190986 | cyan | SGCG | darkgrey | CMAS | grey | LOC100291323 | grey | RGSL1 | grey | FBP1 | midnightblue |
| IFITM10 | blue | SLC30A2 | blue | LOC100240728 | cyan | SH3BGRL2 | darkgrey | CMC1 | grey | LOC100310756 | grey | RHBDD2 | grey | FGF2 | midnightblue |
| IFNAR2 | blue | SLC30A9 | blue | LOC100287877 | cyan | SH3BP4 | darkgrey | CMC2 | grey | LOC100422212 | grey | RHBDF1 | grey | FGL2 | midnightblue |
| IFNL1 | blue | SLC32A1 | blue | LOC100289092 | cyan | SHANK2 | darkgrey | CMC4 | grey | LOC100422737 | grey | RHNO1 | grey | FILIP1L | midnightblue |
| IFNW1 | blue | SLC34A1 | blue | LOC100289094 | cyan | SHB | darkgrey | CMIP | grey | LOC100422781 | grey | RHOBTB1 | grey | FKBP2 | midnightblue |
| IFT57 | blue | SLC34A3 | blue | LOC100505534 | cyan | SHROOM3 | darkgrey | CMPK2 | grey | LOC100499194 | grey | RHOC | grey | FMO4 | midnightblue |
| IFT74 | blue | SLC35B3 | blue | LOC100505853 | cyan | SHROOM4 | darkgrey | CMTM3 | grey | LOC100505474 | grey | RHOG | grey | FURIN | midnightblue |
| IGF2-AS | blue | SLC35C1 | blue | LOC100506457 | cyan | SIGLEC15 | darkgrey | CMTM7 | grey | LOC100505478 | grey | RHOH | grey | FUT2 | midnightblue |
| IGF2BP1 | blue | SLC35C2 | blue | LOC100507477 | cyan | SIPA1L2 | darkgrey | CMTM8 | grey | LOC100505501 | grey | RHOXF1 | grey | FYN | midnightblue |
| IGFALS | blue | SLC35D1 | blue | LOC100996345 | cyan | SLAMF7 | darkgrey | CMTR1 | grey | LOC100505515 | grey | RIC3 | grey | GALNT6 | midnightblue |
| IGFLR1 | blue | SLC35F5 | blue | LOC100996583 | cyan | SLC11A2 | darkgrey | CNDP1 | grey | LOC100505564 | grey | RIC8B | grey | GCNT3 | midnightblue |
| IGFN1 | blue | SLC36A4 | blue | LOC100996724 | cyan | SLC12A2 | darkgrey | CNGA1 | grey | LOC100505570 | grey | RILP | grey | GGT6 | midnightblue |
| IGH | blue | SLC38A3 | blue | LOC100996740 | cyan | SLC13A2 | darkgrey | CNKSR2 | grey | LOC100505622 | grey | RILPL1 | grey | GIMAP8 | midnightblue |
| IGLON5 | blue | SLC38A5 | blue | LOC101926987 | cyan | SLC14A1 | darkgrey | CNN1 | grey | LOC100505635 | grey | RILPL2 | grey | GJB5 | midnightblue |
| IGSF21 | blue | SLC38A6 | blue | LOC101927018 | cyan | SLC16A10 | darkgrey | CNNM1 | grey | LOC100505658 | grey | RINT1 | grey | GLP1R | midnightblue |
| IGSF9 | blue | SLC39A3 | blue | LOC101927138 | cyan | SLC17A5 | darkgrey | CNNM3 | grey | LOC100505685 | grey | RIPK3 | grey | GLT8D2 | midnightblue |
| IGSF9B | blue | SLC39A5 | blue | LOC101927151 | cyan | SLC1A1 | darkgrey | CNPY1 | grey | LOC100505710 | grey | RITA1 | grey | GNAL | midnightblue |
| IHH | blue | SLC3A2 | blue | LOC101927206 | cyan | SLC1A2 | darkgrey | CNTD2 | grey | LOC100505711 | grey | RLF | grey | GNMT | midnightblue |
| IKBKG | blue | SLC43A2 | blue | LOC101927257 | cyan | SLC1A4 | darkgrey | CNTN1 | grey | LOC100505716 | grey | RMI1 | grey | GPR157 | midnightblue |
| IKZF4 | blue | SLC45A1 | blue | LOC101927406 | cyan | SLC25A5 | darkgrey | CNTN3 | grey | LOC100505718 | grey | RMI2 | grey | GPR56 | midnightblue |
| IKZF5 | blue | SLC45A2 | blue | LOC101927460 | cyan | SLC27A3 | darkgrey | CNTN4 | grey | LOC100505774 | grey | RNA45S5 | grey | GUCY1B3 | midnightblue |
| IL10RB-AS1 | blue | SLC45A3 | blue | LOC101927490 | cyan | SLC29A1 | darkgrey | CNTNAP1 | grey | LOC100505776 | grey | RNASE11 | grey | GYLTL1B | midnightblue |
| IL11 | blue | SLC46A2 | blue | LOC101927746 | cyan | SLC31A2 | darkgrey | CNTNAP2 | grey | LOC100505782 | grey | RNASE6 | grey | HDAC4 | midnightblue |
| IL12RB1 | blue | SLC4A7 | blue | LOC101928433 | cyan | SLC33A1 | darkgrey | CNTNAP3 | grey | LOC100505784 | grey | RNASEH1-AS1 | grey | HID1 | midnightblue |
| IL13 | blue | SLC4A9 | blue | LOC101928457 | cyan | SLC34A2 | darkgrey | CNTNAP3P2 | grey | LOC100505811 | grey | RNASEH2A | grey | HIP1 | midnightblue |
| IL17B | blue | SLC52A3 | blue | LOC101928557 | cyan | SLC35A3 | darkgrey | CNTRL | grey | LOC100505824 | grey | RNASEH2B | grey | HOOK1 | midnightblue |
| IL17C | blue | SLC5A11 | blue | LOC101928647 | cyan | SLC35B1 | darkgrey | COA4 | grey | LOC100505874 | grey | RNASEH2C | grey | IGLV6-57 | midnightblue |
| IL17F | blue | SLC5A6 | blue | LOC101929002 | cyan | SLC35F2 | darkgrey | COA5 | grey | LOC100505878 | grey | RNF112 | grey | IL20RA | midnightblue |
| IL17RE | blue | SLC6A11 | blue | LOC101929027 | cyan | SLC38A1 | darkgrey | COA6 | grey | LOC100505902 | grey | RNF113A | grey | ILDR1 | midnightblue |
| IL1F10 | blue | SLC6A17 | blue | LOC101929076 | cyan | SLC39A11 | darkgrey | COASY | grey | LOC100505912 | grey | RNF11B | grey | KATNAL1 | midnightblue |
| IL1RAPL1 | blue | SLC6A19 | blue | LOC101929154 | cyan | SLC39A6 | darkgrey | COCH | grey | LOC100505915 | grey | RNF122 | grey | KCNC4 | midnightblue |
| IL1RL2 | blue | SLC6A2 | blue | LOC101929177 | cyan | SLC39A9 | darkgrey | COG6 | grey | LOC100505918 | grey | RNF123 | grey | KCNN4 | midnightblue |
| IL20RB | blue | SLC6A3 | blue | LOC101929239 | cyan | SLC44A3 | darkgrey | COG7 | grey | LOC100506022 | grey | RNF133 | grey | KCNQ1 | midnightblue |
| IL27 | blue | SLC6A7 | blue | LOC101929372 | cyan | SLC50A1 | darkgrey | COL11A2 | grey | LOC100506047 | grey | RNF135 | grey | KCNS1 | midnightblue |
| IL3 | blue | SLC7A10 | blue | LOC101929478 | cyan | SLC5A1 | darkgrey | COL16A1 | grey | LOC100506113 | grey | RNF139-AS1 | grey | KIDINS220 | midnightblue |
| IL34 | blue | SLC7A3 | blue | LOC101930415 | cyan | SLC6A14 | darkgrey | COL18A1 | grey | LOC100506122 | grey | RNF144A-AS1 | grey | KLF7 | midnightblue |
| IL36A | blue | SLC7A4 | blue | LOC102723831 | cyan | SLC7A1 | darkgrey | COL21A1 | grey | LOC100506235 | grey | RNF148 | grey | KRT15 | midnightblue |
| IL3RA | blue | SLC8B1 | blue | LOC102724356 | cyan | SLC7A2 | darkgrey | COL27A1 | grey | LOC100506236 | grey | RNF157 | grey | KRT19 | midnightblue |
| IL4 | blue | SLC9A2 | blue | LOC145837 | cyan | SLC9A1 | darkgrey | COL28A1 | grey | LOC100506272 | grey | RNF157-AS1 | grey | KRT7 | midnightblue |
| IL4I1 | blue | SLC9A5 | blue | LOC150005 | cyan | SLC9A3R1 | darkgrey | COL4A5 | grey | LOC100506289 | grey | RNF166 | grey | KRT8 | midnightblue |
| IL5 | blue | SLC9A8 | blue | LOC151484 | cyan | SLC9A7 | darkgrey | COL6A6 | grey | LOC100506319 | grey | RNF175 | grey | LAD1 | midnightblue |
| IL9R | blue | SLCO4A1 | blue | LOC202181 | cyan | SLIT3 | darkgrey | COL8A1 | grey | LOC100506325 | grey | RNF182 | grey | LEPREL1 | midnightblue |
| ILKAP | blue | SLIT1 | blue | LOC255177 | cyan | SLITRK5 | darkgrey | COL8A2 | grey | LOC100506351 | grey | RNF183 | grey | LIPH | midnightblue |
| ILVBL | blue | SLMAP | blue | LOC283070 | cyan | SLPI | darkgrey | COLCA1 | grey | LOC100506368 | grey | RNF185 | grey | LOC100128079 | midnightblue |
| IMMT | blue | SLMO1 | blue | LOC283177 | cyan | SMCO4 | darkgrey | COLCA2 | grey | LOC100506371 | grey | RNF185-AS1 | grey | LOC101926907 | midnightblue |
| ING4 | blue | SLURP1 | blue | LOC283745 | cyan | SMCR8 | darkgrey | COLEC10 | grey | LOC100506388 | grey | RNF19B | grey | LOC101927703 | midnightblue |
| INGX | blue | SLX4 | blue | LOC284080 | cyan | SMDT1 | darkgrey | COLEC11 | grey | LOC100506405 | grey | RNF2 | grey | LOC101927811 | midnightblue |
| INHA | blue | SMAD4 | blue | LOC284240 | cyan | SMR3A | darkgrey | COLGALT1 | grey | LOC100506459 | grey | RNF208 | grey | LOC102724362 | midnightblue |
| INO80 | blue | SMARCD3 | blue | LOC284837 | cyan | SMR3B | darkgrey | COLGALT2 | grey | LOC100506470 | grey | RNF212B | grey | LRRC8E | midnightblue |
| INO80C | blue | SMC3 | blue | LOC285593 | cyan | SNTB2 | darkgrey | COMMD1 | grey | LOC100506476 | grey | RNF215 | grey | LUZP1 | midnightblue |
| INPP5K | blue | SMC4 | blue | LOC285692 | cyan | SNX21 | darkgrey | COMMD7 | grey | LOC100506489 | grey | RNF24 | grey | MALL | midnightblue |
| INPPL1 | blue | SMC5 | blue | LOC286052 | cyan | SOD3 | darkgrey | COMMD8 | grey | LOC100506526 | grey | RNF40 | grey | MAP4K4 | midnightblue |
| INS | blue | SMCHD1 | blue | LOC286068 | cyan | SORL1 | darkgrey | COMMD9 | grey | LOC100506538 | grey | RNF41 | grey | ME1 | midnightblue |
| INSL3 | blue | SMEK1 | blue | LOC286382 | cyan | SOX10 | darkgrey | COPRS | grey | LOC100506563 | grey | RNF5 | grey | MKL2 | midnightblue |
| INSL6 | blue | SMEK2 | blue | LOC340090 | cyan | SOX8 | darkgrey | COPS4 | grey | LOC100506603 | grey | RNF8 | grey | MSLN | midnightblue |
| INSRR | blue | SMG5 | blue | LOC400684 | cyan | SOX9 | darkgrey | COPS6 | grey | LOC100506606 | grey | RNFT2 | grey | MUC16 | midnightblue |
| INTS3 | blue | SMG7 | blue | LOC400940 | cyan | SPATS2 | darkgrey | COPS7A | grey | LOC100506667 | grey | RNMTL1 | grey | MZB1 | midnightblue |
| INTS6 | blue | SMG7-AS1 | blue | LOC401134 | cyan | SPCS3 | darkgrey | COPZ2 | grey | LOC100506691 | grey | RNPEP | grey | NBEAL1 | midnightblue |
| INTS8 | blue | SMIM1 | blue | LOC440117 | cyan | SPINT1 | darkgrey | COQ10A | grey | LOC100506699 | grey | ROBO3 | grey | NBEAL2 | midnightblue |
| INTS9 | blue | SMIM24 | blue | LOC440434 | cyan | SPINT2 | darkgrey | COQ5 | grey | LOC100506725 | grey | ROGDI | grey | NEURL3 | midnightblue |
| IP6K1 | blue | SMIM5 | blue | LOC441178 | cyan | SRGAP1 | darkgrey | COQ6 | grey | LOC100506731 | grey | ROMO1 | grey | NIPAL3 | midnightblue |
| IPPK | blue | SMNDC1 | blue | LOC643201 | cyan | SRP54 | darkgrey | CORO1A | grey | LOC100506790 | grey | ROR1 | grey | NPAS2 | midnightblue |
| IQCC | blue | SMOX | blue | LOC648691 | cyan | SRPK1 | darkgrey | CORO2A | grey | LOC100506797 | grey | ROR2 | grey | NRCAM | midnightblue |
| IQCD | blue | SMPD3 | blue | LOC727944 | cyan | SRPRB | darkgrey | CORO2B | grey | LOC100506851 | grey | RORB | grey | OLFML2A | midnightblue |
| IQCF2 | blue | SMTNL1 | blue | LOC728040 | cyan | SSR2 | darkgrey | CORO6 | grey | LOC100506858 | grey | RP1-100J12.1 | grey | OTX1 | midnightblue |
| IQCF3 | blue | SMTNL2 | blue | LOC728061 | cyan | SSR4 | darkgrey | COTL1 | grey | LOC100506860 | grey | RP1-130G2.1 | grey | PARP4 | midnightblue |
| IQCF6 | blue | SMYD1 | blue | LOC728690 | cyan | STAP2 | darkgrey | COX1 | grey | LOC100506929 | grey | RP1-135L22.1 | grey | PART1 | midnightblue |
| IQCH-AS1 | blue | SNAI1 | blue | LOC729732 | cyan | STARD7 | darkgrey | COX10-AS1 | grey | LOC100506990 | grey | RP1-142L7.8 | grey | PEBP4 | midnightblue |
| IQCJ-SCHIP1-AS1 | blue | SNAPC2 | blue | LOC780529 | cyan | STAT5A | darkgrey | COX14 | grey | LOC100507054 | grey | RP1-142L7.9 | grey | PID1 | midnightblue |
| IQSEC2 | blue | SNAPC3 | blue | LOH12CR2 | cyan | STATH | darkgrey | COX17 | grey | LOC100507065 | grey | RP1-149C7.1 | grey | PIK3CA | midnightblue |
| IQSEC3 | blue | SNCB | blue | LPCAT4 | cyan | STEAP1 | darkgrey | COX4I2 | grey | LOC100507156 | grey | RP1-151F17.2 | grey | PKP3 | midnightblue |
| IRF5 | blue | SNCG | blue | LPIN1 | cyan | STEAP3 | darkgrey | COX6A2 | grey | LOC100507165 | grey | RP1-187B23.1 | grey | PLEKHB1 | midnightblue |
| IRF9 | blue | SNHG10 | blue | LPP | cyan | STK39 | darkgrey | COX7A1 | grey | LOC100507221 | grey | RP1-190J20.2 | grey | PLXNB3 | midnightblue |
| IRGC | blue | SNORA71B | blue | LPXN | cyan | STON1 | darkgrey | COX8A | grey | LOC100507250 | grey | RP1-193H18.2 | grey | PLXNC1 | midnightblue |
| IRS4 | blue | SNORA78 | blue | LRG1 | cyan | STX2 | darkgrey | CPA2 | grey | LOC100507277 | grey | RP1-217P22.2 | grey | POSTN | midnightblue |
| ISG15 | blue | SNORD89 | blue | LRP11 | cyan | SULT1A2 | darkgrey | CPA4 | grey | LOC100507283 | grey | RP1-228H13.5 | grey | PREX2 | midnightblue |
| ISLR2 | blue | SNPH | blue | LRPPRC | cyan | SURF4 | darkgrey | CPAMD8 | grey | LOC100507311 | grey | RP1-30M3.5 | grey | PRKAB2 | midnightblue |
| IST1 | blue | SNRNP40 | blue | LRRFIP1 | cyan | SUSD4 | darkgrey | CPB2 | grey | LOC100507351 | grey | RP1-31B8.1 | grey | PRKCZ | midnightblue |
| ISYNA1 | blue | SNRPD3 | blue | LRRK2 | cyan | SVEP1 | darkgrey | CPEB1 | grey | LOC100507384 | grey | RP1-39J2.1 | grey | PROM2 | midnightblue |
| ITCH | blue | SNTA1 | blue | LRRN2 | cyan | SVIP | darkgrey | CPLX1 | grey | LOC100507388 | grey | RP1-58B11.1 | grey | PYCR1 | midnightblue |
| ITGA2B | blue | SNTG2 | blue | LRRN4 | cyan | SVOPL | darkgrey | CPN1 | grey | LOC100507395 | grey | RP1-68D18.4 | grey | QKI | midnightblue |
| ITGA3 | blue | SNW1 | blue | LRTM1 | cyan | SYDE1 | darkgrey | CPN2 | grey | LOC100507403 | grey | RP1-6P5.2 | grey | QPRT | midnightblue |
| ITGB2-AS1 | blue | SNX13 | blue | LSM1 | cyan | SYNE4 | darkgrey | CPNE1 | grey | LOC100507419 | grey | RP1-74M1.3 | grey | RAB17 | midnightblue |
| ITGB3BP | blue | SNX14 | blue | LSM12 | cyan | SYNPO | darkgrey | CPNE4 | grey | LOC100507431 | grey | RP1-86D1.3 | grey | RAB25 | midnightblue |
| ITGB7 | blue | SNX22 | blue | LSM14A | cyan | TADA2B | darkgrey | CPO | grey | LOC100507443 | grey | RP1-93H18.6 | grey | RAB26 | midnightblue |
| ITIH1 | blue | SNX32 | blue | LSM2 | cyan | TBC1D24 | darkgrey | CPSF1 | grey | LOC100507459 | grey | RP11-1007O24.2 | grey | RAP1GAP | midnightblue |
| ITIH4 | blue | SNX8 | blue | LTB4R | cyan | TBC1D30 | darkgrey | CPSF3 | grey | LOC100507468 | grey | RP11-1012E15.1 | grey | RASAL1 | midnightblue |
| ITIH6 | blue | SOAT1 | blue | LTN1 | cyan | TBC1D9 | darkgrey | CPSF4 | grey | LOC100507480 | grey | RP11-1017G21.4 | grey | RASSF8 | midnightblue |
| ITPKA | blue | SOCS1 | blue | LUC7L3 | cyan | TC2N | darkgrey | CPT1A | grey | LOC100507501 | grey | RP11-1024P17.1 | grey | RFTN2 | midnightblue |
| ITPKB-IT1 | blue | SOCS2-AS1 | blue | LXN | cyan | TCN1 | darkgrey | CPTP | grey | LOC100507506 | grey | RP11-1069G10.1 | grey | RHOJ | midnightblue |
| IZUMO1 | blue | SOCS4 | blue | LY6G6C | cyan | TECPR1 | darkgrey | CPVL | grey | LOC100507516 | grey | RP11-1081M5.2 | grey | RMDN1 | midnightblue |
| IZUMO4 | blue | SOCS5 | blue | LY96 | cyan | TFAP2A | darkgrey | CPXM1 | grey | LOC100507520 | grey | RP11-108K3.2 | grey | ROPN1 | midnightblue |
| JADE2 | blue | SOHLH1 | blue | LYPLA1 | cyan | TFAP2C | darkgrey | CR1L | grey | LOC100507535 | grey | RP11-108P20.4 | grey | ROPN1B | midnightblue |
| JAG2 | blue | SORCS2 | blue | LYPLAL1 | cyan | TFCP2L1 | darkgrey | CR936796 | grey | LOC100507540 | grey | RP11-109D24.1 | grey | RORC | midnightblue |
| JAKMIP1 | blue | SOX1 | blue | LYRM5 | cyan | TIE1 | darkgrey | CRABP2 | grey | LOC100507557 | grey | RP11-109D9.4 | grey | RP11-164P12.4 | midnightblue |
| JARID2-AS1 | blue | SOX14 | blue | LYST | cyan | TIFA | darkgrey | CRB1 | grey | LOC100507560 | grey | RP11-109G23.3 | grey | S1PR1 | midnightblue |
| JMJD4 | blue | SOX15 | blue | M6PR | cyan | TJP2 | darkgrey | CRCP | grey | LOC100507564 | grey | RP11-1109F11.3 | grey | SCARF1 | midnightblue |
| JMJD6 | blue | SOX18 | blue | MAB21L3 | cyan | TMC4 | darkgrey | CREB3 | grey | LOC100507642 | grey | RP11-1109F11.5 | grey | SCGB3A1 | midnightblue |
| JMY | blue | SOX2 | blue | MACF1 | cyan | TMEM125 | darkgrey | CREB3L1 | grey | LOC100508631 | grey | RP11-1114A5.4 | grey | SCNN1A | midnightblue |
| JPH2 | blue | SOX21 | blue | MAGEB3 | cyan | TMEM139 | darkgrey | CREB5 | grey | LOC100509303 | grey | RP11-111K18.2 | grey | SEZ6L2 | midnightblue |
| JPH3 | blue | SOX3 | blue | MAGED1 | cyan | TMEM213 | darkgrey | CRELD1 | grey | LOC100631378 | grey | RP11-111M22.4 | grey | SGSM3 | midnightblue |
| JRK | blue | SP2 | blue | MAGI3 | cyan | TMEM214 | darkgrey | CRHBP | grey | LOC100652824 | grey | RP11-112J3.16 | grey | SIPA1L3 | midnightblue |
| JSRP1 | blue | SP3 | blue | MAGIX | cyan | TMEM258 | darkgrey | CRIM1 | grey | LOC100652911 | grey | RP11-1152H14.1 | grey | SLC35F3 | midnightblue |
| KANSL1 | blue | SP3P | blue | MAGT1 | cyan | TMEM45A | darkgrey | CRIP1 | grey | LOC100652999 | grey | RP11-115A15.2 | grey | SLC44A4 | midnightblue |
| KANSL1L | blue | SP5 | blue | MAK16 | cyan | TMEM56 | darkgrey | CRIP2 | grey | LOC100653005 | grey | RP11-116D17.1 | grey | SLC4A11 | midnightblue |
| KAT2A | blue | SP6 | blue | MALAT1 | cyan | TMEM99 | darkgrey | CRISPLD1 | grey | LOC100996251 | grey | RP11-116O18.1 | grey | SLC7A5 | midnightblue |
| KATNAL2 | blue | SP8 | blue | MAML2 | cyan | TMX4 | darkgrey | CRISPLD2 | grey | LOC100996255 | grey | RP11-118G23.2 | grey | SLC7A6 | midnightblue |
| KATNB1 | blue | SPACA3 | blue | MAN2B2 | cyan | TNFRSF11A | darkgrey | CRLF2 | grey | LOC100996286 | grey | RP11-119F7.5 | grey | SLCO4A1-AS1 | midnightblue |
| KATNBL1 | blue | SPACA4 | blue | MANBAL | cyan | TNFRSF17 | darkgrey | CRMP1 | grey | LOC100996404 | grey | RP11-1217F2.1 | grey | SNRK | midnightblue |
| KAZALD1 | blue | SPAG11A | blue | MAP1LC3B | cyan | TNFRSF19 | darkgrey | CRTAM | grey | LOC100996412 | grey | RP11-121C2.2 | grey | SPDEF | midnightblue |
| KCMF1 | blue | SPAG5-AS1 | blue | MAP3K1 | cyan | TNFSF13 | darkgrey | CRTC3 | grey | LOC100996425 | grey | RP11-123K19.2 | grey | STARD10 | midnightblue |
| KCNA10 | blue | SPAG8 | blue | MAP3K19 | cyan | TNS1 | darkgrey | CRY1 | grey | LOC100996455 | grey | RP11-124L9.5 | grey | SYNC | midnightblue |
| KCNA7 | blue | SPANXB1 | blue | MAP3K4 | cyan | TNXB | darkgrey | CRY2 | grey | LOC100996457 | grey | RP11-1260E13.2 | grey | SYT7 | midnightblue |
| KCNAB2 | blue | SPAST | blue | MAPK1 | cyan | TOM1L1 | darkgrey | CRYGN | grey | LOC100996549 | grey | RP11-1275H24.2 | grey | TBCB | midnightblue |
| KCNAB3 | blue | SPATA19 | blue | MAPK14 | cyan | TPD52 | darkgrey | CRYGS | grey | LOC100996624 | grey | RP11-1277A3.1 | grey | TFPI | midnightblue |
| KCNC2 | blue | SPATA21 | blue | MAPK1IP1L | cyan | TPD52L1 | darkgrey | CRYL1 | grey | LOC100996681 | grey | RP11-127B20.2 | grey | TJP3 | midnightblue |
| KCNC3 | blue | SPATA24 | blue | MAPK9 | cyan | TRAK1 | darkgrey | CRYZL1 | grey | LOC100996756 | grey | RP11-131L23.2 | grey | TMED9 | midnightblue |
| KCND1 | blue | SPATA3 | blue | MAPKAPK5 | cyan | TRAPPC3 | darkgrey | CSAD | grey | LOC100996760 | grey | RP11-134G8.8 | grey | TMEM120B | midnightblue |
| KCNF1 | blue | SPATA3-AS1 | blue | MAPRE1 | cyan | TRIM2 | darkgrey | CSF2RB | grey | LOC100996902 | grey | RP11-134L10.1 | grey | TMPRSS2 | midnightblue |
| KCNG1 | blue | SPATA31E1 | blue | MARCKS | cyan | TRIM29 | darkgrey | CSF3R | grey | LOC101060019 | grey | RP11-138I17.1 | grey | TMPRSS3 | midnightblue |
| KCNH1 | blue | SPATA33 | blue | MARVELD3 | cyan | TRPT1 | darkgrey | CSH1 | grey | LOC101060391 | grey | RP11-138I18.2 | grey | TNS4 | midnightblue |
| KCNH2 | blue | SPATA42 | blue | MAX | cyan | TSPAN13 | darkgrey | CSK | grey | LOC101060424 | grey | RP11-13K12.5 | grey | TSHZ1 | midnightblue |
| KCNH3 | blue | SPATC1 | blue | MAZ | cyan | TSPAN33 | darkgrey | CSMD2-AS1 | grey | LOC101593348 | grey | RP11-140I16.3 | grey | TSTA3 | midnightblue |
| KCNH4 | blue | SPATS2L | blue | MBD3L1 | cyan | TSPAN4 | darkgrey | CSMD3 | grey | LOC101926906 | grey | RP11-141M1.1 | grey | TXNDC11 | midnightblue |
| KCNH5 | blue | SPC24 | blue | MBNL1 | cyan | TSTD1 | darkgrey | CSN1S2AP | grey | LOC101926912 | grey | RP11-143I21.1 | grey | U47924.27 | midnightblue |
| KCNH6 | blue | SPEF1 | blue | MBNL2 | cyan | TTC22 | darkgrey | CSN2 | grey | LOC101926915 | grey | RP11-143K11.1 | grey | UHRF1BP1L | midnightblue |
| KCNIP3 | blue | SPEM1 | blue | MBTD1 | cyan | TUB | darkgrey | CSNK1A1P1 | grey | LOC101926918 | grey | RP11-152L20.3 | grey | WFDC2 | midnightblue |
| KCNJ4 | blue | SPERT | blue | MCC | cyan | TUFT1 | darkgrey | CSNK1D | grey | LOC101926942 | grey | RP11-153K16.2 | grey | WNK1 | midnightblue |
| KCNJ9 | blue | SPHK2 | blue | MCHR2-AS1 | cyan | TWIST2 | darkgrey | CSNK1E | grey | LOC101926943 | grey | RP11-157B13.7 | grey | WNK2 | midnightblue |
| KCNK16 | blue | SPIN2A | blue | MCM10 | cyan | UBA5 | darkgrey | CSNK1G2 | grey | LOC101926960 | grey | RP11-157P1.4 | grey | WSB1 | midnightblue |
| KCNK4 | blue | SPN | blue | MCTP1 | cyan | UBE2J1 | darkgrey | CSPG4P5 | grey | LOC101926967 | grey | RP11-158G18.1 | grey | YIPF2 | midnightblue |
| KCNK7 | blue | SPNS1 | blue | MCUR1 | cyan | UGT8 | darkgrey | CSRNP1 | grey | LOC101926996 | grey | RP11-166P13.4 | grey | YWHAH | midnightblue |
| KCNK9 | blue | SPNS3 | blue | MDM4 | cyan | UTP20 | darkgrey | CSRP1 | grey | LOC101927020 | grey | RP11-171I2.4 | grey | ZBP1 | midnightblue |
| KCNN1 | blue | SPOCD1 | blue | MDS2 | cyan | VAMP8 | darkgrey | CSRP3 | grey | LOC101927021 | grey | RP11-173B14.4 | grey | ZDHHC17 | midnightblue |
| KCNQ1DN | blue | SPOPL | blue | ME2 | cyan | VIMP | darkgrey | CST6 | grey | LOC101927027 | grey | RP11-173M1.8 | grey | ZEB2 | midnightblue |
| KCNQ2 | blue | SPPL2A | blue | MEA1 | cyan | VPS51 | darkgrey | CST7 | grey | LOC101927038 | grey | RP11-174G6.5 | grey | ZNF385D | midnightblue |
| KCNQ4 | blue | SPPL2B | blue | MED1 | cyan | VSIG10L | darkgrey | CST8 | grey | LOC101927044 | grey | RP11-177N22.3 | grey | ACACB | salmon |
| KCNT1 | blue | SPPL2C | blue | MED13 | cyan | VTCN1 | darkgrey | CSTB | grey | LOC101927058 | grey | RP11-179B15.6 | grey | ACO1 | salmon |
| KCP | blue | SPR | blue | MED13L | cyan | WIPF3 | darkgrey | CSTL1 | grey | LOC101927067 | grey | RP11-17A4.3 | grey | ACOX1 | salmon |
| KCTD11 | blue | SPRED3 | blue | MED29 | cyan | WIPI1 | darkgrey | CT83 | grey | LOC101927071 | grey | RP11-180N14.1 | grey | ACSS2 | salmon |
| KCTD17 | blue | SPRN | blue | MED7 | cyan | WISP2 | darkgrey | CTA-250D10.23 | grey | LOC101927085 | grey | RP11-182J23.1 | grey | ADCY5 | salmon |
| KCTD19 | blue | SPRR2C | blue | MEF2A | cyan | WNT5A | darkgrey | CTA-268H5.14 | grey | LOC101927093 | grey | RP11-184E9.2 | grey | ADH1B | salmon |
| KCTD2 | blue | SPRYD3 | blue | MEF2C | cyan | WWC1 | darkgrey | CTA-280A3__B.2 | grey | LOC101927095 | grey | RP11-186F10.2 | grey | ADIPOQ | salmon |
| KCTD3 | blue | SPSB2 | blue | MEIS3P1 | cyan | XBP1 | darkgrey | CTA-29F11.1 | grey | LOC101927122 | grey | RP11-18F14.4 | grey | AGPAT2 | salmon |
| KCTD5 | blue | SPSB3 | blue | METAP1 | cyan | XK | darkgrey | CTAGE11P | grey | LOC101927133 | grey | RP11-18I14.11 | grey | AIFM2 | salmon |
| KDF1 | blue | SPSB4 | blue | METAP2 | cyan | XPNPEP2 | darkgrey | CTB-102L5.7 | grey | LOC101927143 | grey | RP11-194N12.2 | grey | AKR1C1 | salmon |
| KDM1B | blue | SPTB | blue | METTL23 | cyan | XPO6 | darkgrey | CTB-119C2.1 | grey | LOC101927157 | grey | RP11-199F11.2 | grey | AKT2 | salmon |
| KDM2B | blue | SPTBN2 | blue | METTL5 | cyan | YIPF3 | darkgrey | CTB-12A17.3 | grey | LOC101927159 | grey | RP11-1C8.6 | grey | ALDH2 | salmon |
| KDM5A | blue | SPTBN4 | blue | METTL7A | cyan | YKT6 | darkgrey | CTB-167B5.2 | grey | LOC101927210 | grey | RP11-209A2.1 | grey | AOC3 | salmon |
| KDM5C | blue | SPTBN5 | blue | MFF | cyan | ZBED5-AS1 | darkgrey | CTB-176F20.3 | grey | LOC101927228 | grey | RP11-210K20.4 | grey | APBB1IP | salmon |
| KDM6A | blue | SPTLC2 | blue | MFSD1 | cyan | ZCCHC24 | darkgrey | CTB-78F1.1 | grey | LOC101927253 | grey | RP11-214K3.19 | grey | APOE | salmon |
| KDM8 | blue | SPTSSB | blue | MGC12916 | cyan | ZDHHC16 | darkgrey | CTC-338M12.4 | grey | LOC101927256 | grey | RP11-214K3.20 | grey | APOL6 | salmon |
| KHDC3L | blue | SPTY2D1-AS1 | blue | MGEA5 | cyan | ZDHHC2 | darkgrey | CTC-428G20.3 | grey | LOC101927263 | grey | RP11-214N9.1 | grey | AQPEP | salmon |
| KHK | blue | SRCRB4D | blue | MGRN1 | cyan | ZDHHC5 | darkgrey | CTC-459F4.3 | grey | LOC101927273 | grey | RP11-215E13.2 | grey | ATP9A | salmon |
| KIAA0319 | blue | SRD5A2 | blue | MGST3 | cyan | ZG16B | darkgrey | CTC-471J1.2 | grey | LOC101927274 | grey | RP11-216L13.19 | grey | BLCAP | salmon |
| KIAA0907 | blue | SRD5A3 | blue | MIA3 | cyan | ZHX3 | darkgrey | CTC-527H23.4 | grey | LOC101927282 | grey | RP11-218F4.1 | grey | BOK | salmon |
| KIAA1109 | blue | SREBF1 | blue | MIB1 | cyan | ZNF221 | darkgrey | CTD-2021H9.3 | grey | LOC101927286 | grey | RP11-21L23.2 | grey | BTBD11 | salmon |
| KIAA1257 | blue | SREBF2 | blue | MICU1 | cyan | ZNF296 | darkgrey | CTD-2035E11.5 | grey | LOC101927287 | grey | RP11-220I1.5 | grey | BTD | salmon |
| KIAA1279 | blue | SREK1 | blue | MICU2 | cyan | ZNF33B | darkgrey | CTD-2076M15.1 | grey | LOC101927292 | grey | RP11-222K16.1 | grey | C14orf180 | salmon |
| KIAA1328 | blue | SREK1IP1 | blue | MID1IP1 | cyan | ZNF652 | darkgrey | CTD-2083E4.4 | grey | LOC101927313 | grey | RP11-226L15.5 | grey | C2CD2 | salmon |
| KIAA1456 | blue | SRL | blue | MIER1 | cyan | ZNF697 | darkgrey | CTD-2165H16.3 | grey | LOC101927340 | grey | RP11-227D13.1 | grey | C3orf55 | salmon |
| KIAA1614 | blue | SRMS | blue | MINA | cyan | ZNF750 | darkgrey | CTD-2196E14.6 | grey | LOC101927348 | grey | RP11-231C18.1 | grey | CACNA2D1 | salmon |
| KIAA1652 | blue | SRPK2 | blue | MIP | cyan | ZNHIT1 | darkgrey | CTD-2286N8.2 | grey | LOC101927358 | grey | RP11-231E19.1 | grey | CALB2 | salmon |
| KIAA1654 | blue | SRPK3 | blue | MIR3916 | cyan | A2M | darkorange | CTD-2292M16.8 | grey | LOC101927362 | grey | RP11-235E17.4 | grey | CAT | salmon |
| KIAA1656 | blue | SRRM3 | blue | MITD1 | cyan | AAED1 | darkorange | CTD-2293H3.1 | grey | LOC101927365 | grey | RP11-247L20.4 | grey | CD300LG | salmon |
| KIAA1683 | blue | SSBP1 | blue | MKKS | cyan | ABCA1 | darkorange | CTD-2297D10.2 | grey | LOC101927379 | grey | RP11-248J18.2 | grey | CD36 | salmon |
| KIAA1715 | blue | SSBP4 | blue | MKLN1 | cyan | ABCA6 | darkorange | CTD-2313J17.5 | grey | LOC101927380 | grey | RP11-250B2.3 | grey | CEBPA | salmon |
| KIAA1875 | blue | SSC5D | blue | MKRN1 | cyan | ABCA9 | darkorange | CTD-2325A15.5 | grey | LOC101927391 | grey | RP11-250B2.6 | grey | CIDEA | salmon |
| KIAA2018 | blue | SSH2 | blue | MLF2 | cyan | ABCC1 | darkorange | CTD-2366F13.2 | grey | LOC101927396 | grey | RP11-251G23.5 | grey | CIDEC | salmon |
| KIF14 | blue | SSNA1 | blue | MLKL | cyan | ABCC9 | darkorange | CTD-2520I13.1 | grey | LOC101927416 | grey | RP11-254F7.1 | grey | COL4A1 | salmon |
| KIF17 | blue | SSPO | blue | MMADHC | cyan | ABI3BP | darkorange | CTD-2537I9.5 | grey | LOC101927417 | grey | RP11-255C15.3 | grey | COL4A2 | salmon |
| KIF19 | blue | SSR4P1 | blue | MMD2 | cyan | ACTR3B | darkorange | CTD-2542L18.1 | grey | LOC101927420 | grey | RP11-258C19.7 | grey | COPG2IT1 | salmon |
| KIF1A | blue | SSTR1 | blue | MMGT1 | cyan | ADAM33 | darkorange | CTD-2553C6.1 | grey | LOC101927438 | grey | RP11-263K19.4 | grey | CPM | salmon |
| KIF22 | blue | SSTR2 | blue | MMP13 | cyan | ADAMTS2 | darkorange | CTD-2554C21.3 | grey | LOC101927451 | grey | RP11-266A24.1 | grey | CRYAB | salmon |
| KIF25 | blue | SSTR3 | blue | MOB1A | cyan | ADAMTS5 | darkorange | CTD-2561B21.11 | grey | LOC101927495 | grey | RP11-266L9.1 | grey | CSTA | salmon |
| KIF25-AS1 | blue | SSTR4 | blue | MOB1B | cyan | ADAMTSL4 | darkorange | CTD-2587M23.1 | grey | LOC101927502 | grey | RP11-274B18.4 | grey | DGAT2 | salmon |
| KIF26A | blue | SSTR5 | blue | MOB3A | cyan | ADAP2 | darkorange | CTD-2616J11.10 | grey | LOC101927513 | grey | RP11-278J20.2 | grey | DHFR | salmon |
| KIF2A | blue | SSUH2 | blue | MOCS2 | cyan | ADD1 | darkorange | CTD-2619J13.13 | grey | LOC101927523 | grey | RP11-279O17.1 | grey | ECE1 | salmon |
| KIF2C | blue | ST3GAL2 | blue | MORC3 | cyan | ADH1C | darkorange | CTD-2619J13.17 | grey | LOC101927526 | grey | RP11-284F21.8 | grey | EDN3 | salmon |
| KIF5C | blue | ST3GAL3 | blue | MORF4L1 | cyan | ADH5 | darkorange | CTD-3092A11.2 | grey | LOC101927531 | grey | RP11-285E9.5 | grey | EEPD1 | salmon |
| KIFC1 | blue | ST3GAL4 | blue | MORF4L2 | cyan | ADIPOR2 | darkorange | CTDP1 | grey | LOC101927534 | grey | RP11-285F7.2 | grey | ELMOD3 | salmon |
| KIFC3 | blue | ST6GALNAC6 | blue | MPHOSPH8 | cyan | ADRA2A | darkorange | CTDSP2 | grey | LOC101927537 | grey | RP11-28F1.2 | grey | FABP4 | salmon |
| KIR2DL1 | blue | ST8SIA2 | blue | MPLKIP | cyan | AEBP1 | darkorange | CTGF | grey | LOC101927543 | grey | RP11-292D4.3 | grey | FAH | salmon |
| KIR2DL3 | blue | ST8SIA5 | blue | MPV17 | cyan | AGMAT | darkorange | CTH | grey | LOC101927552 | grey | RP11-295M18.6 | grey | FGF10 | salmon |
| KIR2DS4 | blue | STAB2 | blue | MRFAP1 | cyan | AGTR1 | darkorange | CTHRC1 | grey | LOC101927553 | grey | RP11-295P9.12 | grey | FMO2 | salmon |
| KIRREL3 | blue | STAC3 | blue | MRI1 | cyan | AK021804 | darkorange | CTNNAL1 | grey | LOC101927588 | grey | RP11-297L17.2 | grey | FOXL2 | salmon |
| KISS1 | blue | STAG3 | blue | MROH2B | cyan | AKAP12 | darkorange | CTNNAP1 | grey | LOC101927599 | grey | RP11-298H24.1 | grey | G0S2 | salmon |
| KLC3 | blue | STAM | blue | MROH9 | cyan | AKR1B1 | darkorange | CTNS | grey | LOC101927609 | grey | RP11-300A12.2 | grey | GLDN | salmon |
| KLF1 | blue | STAR | blue | MRPL1 | cyan | AKR1C3 | darkorange | CTPS1 | grey | LOC101927623 | grey | RP11-301O19.1 | grey | GNAI1 | salmon |
| KLF10 | blue | STARD3 | blue | MRPL18 | cyan | AKT3 | darkorange | CTRB2 | grey | LOC101927640 | grey | RP11-303E16.7 | grey | GPAM | salmon |
| KLF16 | blue | STAT1 | blue | MRPL3 | cyan | ALX1 | darkorange | CTSD | grey | LOC101927641 | grey | RP11-304C12.3 | grey | GPC3 | salmon |
| KLHDC7A | blue | STC2 | blue | MRPL30 | cyan | AMOTL2 | darkorange | CTSE | grey | LOC101927648 | grey | RP11-305E6.4 | grey | GPC4 | salmon |
| KLHL10 | blue | STEAP1B | blue | MRPL33 | cyan | ANGPT1 | darkorange | CTSF | grey | LOC101927650 | grey | RP11-305O6.3 | grey | GPD1 | salmon |
| KLHL17 | blue | STH | blue | MRPL42 | cyan | ANGPTL2 | darkorange | CTSG | grey | LOC101927653 | grey | RP11-307O13.1 | grey | GPX4 | salmon |
| KLHL24 | blue | STK11 | blue | MRPL49 | cyan | ANK2 | darkorange | CTSH | grey | LOC101927660 | grey | RP11-309G3.3 | grey | GYG2 | salmon |
| KLHL25 | blue | STK11IP | blue | MRPL51 | cyan | ANKRD29 | darkorange | CTSL | grey | LOC101927667 | grey | RP11-310J24.3 | grey | HADH | salmon |
| KLHL31 | blue | STK17B | blue | MRPL9 | cyan | ANKRD40 | darkorange | CTSLP8 | grey | LOC101927690 | grey | RP11-314N13.3 | grey | HILPDA | salmon |
| KLHL35 | blue | STK19 | blue | MRPS10 | cyan | ANTXR1 | darkorange | CTSW | grey | LOC101927699 | grey | RP11-315F22.1 | grey | HK2 | salmon |
| KLHL40 | blue | STK3 | blue | MRPS15 | cyan | AOX1 | darkorange | CTXN3 | grey | LOC101927701 | grey | RP11-318A15.2 | grey | HNMT | salmon |
| KLHL8 | blue | STK32C | blue | MRPS21 | cyan | AP2A2 | darkorange | CUBN | grey | LOC101927710 | grey | RP11-319E16.2 | grey | HRASLS5 | salmon |
| KLK1 | blue | STK38L | blue | MRPS36 | cyan | APBB2 | darkorange | CUEDC2 | grey | LOC101927720 | grey | RP11-324J3.1 | grey | HSD11B1 | salmon |
| KLK12 | blue | STK4 | blue | MRPS6 | cyan | APCDD1 | darkorange | CUX2 | grey | LOC101927722 | grey | RP11-326I11.3 | grey | IL17RB | salmon |
| KLK13 | blue | STK40 | blue | MTCH1 | cyan | APMAP | darkorange | CUZD1 | grey | LOC101927735 | grey | RP11-329B9.3 | grey | INF2 | salmon |
| KLK14 | blue | STMN4 | blue | MTCH2 | cyan | APOD | darkorange | CX3CL1 | grey | LOC101927752 | grey | RP11-329B9.5 | grey | ITGA7 | salmon |
| KLK15 | blue | STPG1 | blue | MTDH | cyan | AR | darkorange | CX3CR1 | grey | LOC101927766 | grey | RP11-330O11.3 | grey | KCNIP2 | salmon |
| KLK2 | blue | STRA6 | blue | MTERF2 | cyan | ARHGEF6 | darkorange | CXCL1 | grey | LOC101927769 | grey | RP11-333O1.1 | grey | KLB | salmon |
| KLK3 | blue | STRN | blue | MTFMT | cyan | ARMCX1 | darkorange | CXCL13 | grey | LOC101927809 | grey | RP11-334C17.5 | grey | LARP6 | salmon |
| KLK4 | blue | STX1A | blue | MTFR1L | cyan | ARPIN | darkorange | CXCL14 | grey | LOC101927815 | grey | RP11-334J6.6 | grey | LEP | salmon |
| KLK8 | blue | STYX | blue | MTHFD1 | cyan | ASAP2 | darkorange | CXCL16 | grey | LOC101927820 | grey | RP11-338I21.1 | grey | LGALS12 | salmon |
| KLKB1 | blue | SUFU | blue | MTMR2 | cyan | ASPA | darkorange | CXCL6 | grey | LOC101927841 | grey | RP11-338N10.1 | grey | LINC00310 | salmon |
| KMT2B | blue | SUGP1 | blue | MTMR3 | cyan | ATP1A2 | darkorange | CXCL9 | grey | LOC101927849 | grey | RP11-33O4.1 | grey | LINC01140 | salmon |
| KMT2E-AS1 | blue | SULT2B1 | blue | MTO1 | cyan | ATP2B4 | darkorange | CXCR1 | grey | LOC101927876 | grey | RP11-341N2.1 | grey | LIPE | salmon |
| KNDC1 | blue | SULT4A1 | blue | MTPAP | cyan | AXL | darkorange | CXCR2 | grey | LOC101927877 | grey | RP11-342L8.2 | grey | LOC101929726 | salmon |
| KRAS | blue | SUN2 | blue | MTUS1 | cyan | BACE1 | darkorange | CXCR4 | grey | LOC101927886 | grey | RP11-343H5.6 | grey | LOC101930114 | salmon |
| KRBOX1 | blue | SUN3 | blue | MTUS2-AS1 | cyan | BEND7 | darkorange | CXXC1P1 | grey | LOC101927900 | grey | RP11-347D21.1 | grey | LOC102723493 | salmon |
| KRCC1 | blue | SUN5 | blue | MTX2 | cyan | BHMT2 | darkorange | CXorf21 | grey | LOC101927901 | grey | RP11-348B17.1 | grey | LOC284825 | salmon |
| KREMEN2 | blue | SURF2 | blue | MUC2 | cyan | BICC1 | darkorange | CXorf22 | grey | LOC101927934 | grey | RP11-348P10.2 | grey | LOC401052 | salmon |
| KRMP1 | blue | SURF6 | blue | MUSK | cyan | BNC2 | darkorange | CXorf23 | grey | LOC101927943 | grey | RP11-349E4.1 | grey | LPL | salmon |
| KRT16 | blue | SUSD3 | blue | MXD3 | cyan | C10orf90 | darkorange | CXorf24 | grey | LOC101927948 | grey | RP11-355B11.2 | grey | LSS | salmon |
| KRT19P2 | blue | SUV420H2 | blue | MXI1 | cyan | C14orf132 | darkorange | CXorf38 | grey | LOC101927950 | grey | RP11-356B19.11 | grey | MAOB | salmon |
| KRT24 | blue | SWSAP1 | blue | MYCBP2 | cyan | C1QA | darkorange | CXorf40A | grey | LOC101927974 | grey | RP11-359E8.5 | grey | MAP7D1 | salmon |
| KRT3 | blue | SYMPK | blue | MYCNOS | cyan | C1QB | darkorange | CXorf56 | grey | LOC101928000 | grey | RP11-360A18.2 | grey | MAPK10 | salmon |
| KRT32 | blue | SYN3 | blue | MYD88 | cyan | C1QC | darkorange | CXorf65 | grey | LOC101928002 | grey | RP11-362K14.7 | grey | ME3 | salmon |
| KRT33A | blue | SYNDIG1L | blue | MYL10 | cyan | C1R | darkorange | CYB561A3 | grey | LOC101928009 | grey | RP11-363E7.4 | grey | MEST | salmon |
| KRT35 | blue | SYNGAP1 | blue | MYL12A | cyan | C1S | darkorange | CYB561D2 | grey | LOC101928014 | grey | RP11-369C8.1 | grey | MGST1 | salmon |
| KRT4 | blue | SYP | blue | MYL12B | cyan | C1orf123 | darkorange | CYB5D2 | grey | LOC101928020 | grey | RP11-36B6.1 | grey | MMD | salmon |
| KRT72 | blue | SYT12 | blue | MYL2 | cyan | C1orf198 | darkorange | CYB5R2 | grey | LOC101928031 | grey | RP11-373D23.2 | grey | MRAP | salmon |
| KRT79 | blue | SYT5 | blue | MYL5 | cyan | C1orf54 | darkorange | CYBA | grey | LOC101928043 | grey | RP11-375I20.6 | grey | MRAS | salmon |
| KRT81 | blue | SYT8 | blue | MYLPF | cyan | C21orf88 | darkorange | CYC1 | grey | LOC101928054 | grey | RP11-378A13.1 | grey | MST4 | salmon |
| KRT83 | blue | SYT9 | blue | MYO3A | cyan | C2orf40 | darkorange | CYCS | grey | LOC101928099 | grey | RP11-378J18.8 | grey | NNAT | salmon |
| KRT85 | blue | TACC3 | blue | MYO5A | cyan | C5AR1 | darkorange | CYFIP2 | grey | LOC101928100 | grey | RP11-379F4.6 | grey | NPR1 | salmon |
| KRTAP1-1 | blue | TACO1 | blue | MYO5C | cyan | C6 | darkorange | CYLC2 | grey | LOC101928101 | grey | RP11-37C7.3 | grey | NPR3 | salmon |
| KSR2 | blue | TACR2 | blue | MYO6 | cyan | C8orf88 | darkorange | CYP11A1 | grey | LOC101928104 | grey | RP11-381P6.1 | grey | NQO1 | salmon |
| KXD1 | blue | TAF1B | blue | N4BP2L2 | cyan | CALD1 | darkorange | CYP1A2 | grey | LOC101928107 | grey | RP11-384P7.7 | grey | PCDH9 | salmon |
| L2HGDH | blue | TAF7 | blue | NAA20 | cyan | CALHM2 | darkorange | CYP1B1-AS1 | grey | LOC101928132 | grey | RP11-385F5.4 | grey | PCK1 | salmon |
| L3MBTL1 | blue | TAF9B | blue | NAAA | cyan | CAP2 | darkorange | CYP20A1 | grey | LOC101928134 | grey | RP11-388M20.1 | grey | PECR | salmon |
| LA16c-380H5.4 | blue | TAL1 | blue | NAB1 | cyan | CAV1 | darkorange | CYP24A1 | grey | LOC101928135 | grey | RP11-388M20.6 | grey | PFKFB1 | salmon |
| LA16c-381G6.1 | blue | TAL2 | blue | NACA | cyan | CAV2 | darkorange | CYP2A7 | grey | LOC101928139 | grey | RP11-389C8.2 | grey | PGM1 | salmon |
| LA16c-395F10.2 | blue | TANK | blue | NAE1 | cyan | CC2D2A | darkorange | CYP2B7P | grey | LOC101928152 | grey | RP11-389C8.3 | grey | PKDCC | salmon |
| LACC1 | blue | TAS2R14 | blue | NAP1L1 | cyan | CCDC69 | darkorange | CYP2C19 | grey | LOC101928157 | grey | RP11-38C18.2 | grey | PLA2G16 | salmon |
| LAMA1 | blue | TAS2R45 | blue | NAPG | cyan | CCDC80 | darkorange | CYP2J2 | grey | LOC101928161 | grey | RP11-38C18.3 | grey | PLIN1 | salmon |
| LAMA5-AS1 | blue | TBC1D19 | blue | NARS | cyan | CCND2 | darkorange | CYP2S1 | grey | LOC101928167 | grey | RP11-391M1.4 | grey | PLIN4 | salmon |
| LAMB2P1 | blue | TBC1D21 | blue | NAT8B | cyan | CD163 | darkorange | CYP3A7-CYP3AP1 | grey | LOC101928173 | grey | RP11-394I13.2 | grey | PLOD2 | salmon |
| LAMC2 | blue | TBC1D29 | blue | NBN | cyan | CD209 | darkorange | CYP4F11 | grey | LOC101928185 | grey | RP11-395N3.1 | grey | PLXNA4 | salmon |
| LAMC3 | blue | TBCD | blue | NBPF1 | cyan | CD34 | darkorange | CYP4F30P | grey | LOC101928190 | grey | RP11-399O19.9 | grey | PNPLA2 | salmon |
| LAT | blue | TBKBP1 | blue | NBPF20 | cyan | CD5 | darkorange | CYP4V2 | grey | LOC101928191 | grey | RP11-39H13.1 | grey | PPARG | salmon |
| LBX1 | blue | TBL2 | blue | NCAN | cyan | CD79A | darkorange | CYP4X1 | grey | LOC101928196 | grey | RP11-400N13.1 | grey | PPP1R1A | salmon |
| LBX1-AS1 | blue | TBPL1 | blue | NCK1 | cyan | CD81 | darkorange | CYP4Z1 | grey | LOC101928200 | grey | RP11-401P9.4 | grey | PRKAR2B | salmon |
| LCAT | blue | TBX1 | blue | NCK2 | cyan | CD93 | darkorange | CYP4Z2P | grey | LOC101928201 | grey | RP11-403P17.4 | grey | PRRG4 | salmon |
| LCN15 | blue | TBX10 | blue | NCOA1 | cyan | CD99 | darkorange | CYP7B1 | grey | LOC101928205 | grey | RP11-410L14.2 | grey | PSAT1 | salmon |
| LCN8 | blue | TBX2 | blue | NCOA2 | cyan | CD99L2 | darkorange | CYR61 | grey | LOC101928207 | grey | RP11-416I2.1 | grey | PYGL | salmon |
| LCNL1 | blue | TBX3 | blue | NCOA4 | cyan | CDH5 | darkorange | CYS1 | grey | LOC101928243 | grey | RP11-420K14.2 | grey | RARRES1 | salmon |
| LCOR | blue | TBX4 | blue | NCOA5 | cyan | CDO1 | darkorange | CYSLTR1 | grey | LOC101928245 | grey | RP11-421E14.2 | grey | RASD1 | salmon |
| LCP1 | blue | TBX5 | blue | NCOA7 | cyan | CDON | darkorange | CYSLTR2 | grey | LOC101928255 | grey | RP11-421F16.3 | grey | RBP4 | salmon |
| LDHC | blue | TBX6 | blue | NCSTN | cyan | CELSR2 | darkorange | CYSTM1 | grey | LOC101928269 | grey | RP11-422P24.11 | grey | RDH10 | salmon |
| LDHD | blue | TBXA2R | blue | NDFIP1 | cyan | CFH | darkorange | CYTH1 | grey | LOC101928288 | grey | RP11-432M24.4 | grey | RDH5 | salmon |
| LELP1 | blue | TCAP | blue | NDFIP2 | cyan | CHRDL1 | darkorange | CYTH4 | grey | LOC101928326 | grey | RP11-433A10.3 | grey | RETSAT | salmon |
| LEMD3 | blue | TCEA2 | blue | NDRG3 | cyan | CHST3 | darkorange | CYTL1 | grey | LOC101928335 | grey | RP11-439E19.10 | grey | RNF150 | salmon |
| LENG1 | blue | TCEB3 | blue | NDUFA12 | cyan | CLDN4 | darkorange | D21S2090E | grey | LOC101928343 | grey | RP11-440I14.2 | grey | RP11-2E11.9 | salmon |
| LEPREL2 | blue | TCEB3B | blue | NDUFA4 | cyan | CLIP4 | darkorange | DAAM1 | grey | LOC101928371 | grey | RP11-445H22.4 | grey | RP11-524D16__A.3 | salmon |
| LEPROTL1 | blue | TCERG1 | blue | NDUFA6 | cyan | CLMP | darkorange | DAB1 | grey | LOC101928403 | grey | RP11-44F14.8 | grey | RXRA | salmon |
| LETM1 | blue | TCF12 | blue | NDUFA8 | cyan | CMKLR1 | darkorange | DAB2 | grey | LOC101928409 | grey | RP11-44F21.5 | grey | S100B | salmon |
| LGALS2 | blue | TCF15 | blue | NDUFAB1 | cyan | CNKSR1 | darkorange | DACH1 | grey | LOC101928418 | grey | RP11-452L6.1 | grey | SELENBP1 | salmon |
| LGALS4 | blue | TCF21 | blue | NDUFB1 | cyan | CNN3 | darkorange | DACT2 | grey | LOC101928419 | grey | RP11-456P18.2 | grey | SERTAD4 | salmon |
| LGALS8 | blue | TCL6 | blue | NDUFB5 | cyan | CNRIP1 | darkorange | DACT3-AS1 | grey | LOC101928420 | grey | RP11-457K10.1 | grey | SLC19A3 | salmon |
| LGALS8-AS1 | blue | TCN2 | blue | NDUFB9 | cyan | CNTFR | darkorange | DAG1 | grey | LOC101928429 | grey | RP11-45M22.3 | grey | SLC24A3 | salmon |
| LGI2 | blue | TCOF1 | blue | NDUFS1 | cyan | COL12A1 | darkorange | DAPL1 | grey | LOC101928446 | grey | RP11-461L18.1 | grey | SMIM3 | salmon |
| LGI3 | blue | TCP10L | blue | NDUFV1 | cyan | COL14A1 | darkorange | DARS2 | grey | LOC101928461 | grey | RP11-466A19.8 | grey | SMOC1 | salmon |
| LGI4 | blue | TCRBV12S3 | blue | NDUFV2 | cyan | COL1A1 | darkorange | DAW1 | grey | LOC101928476 | grey | RP11-467D6.1 | grey | SORBS1 | salmon |
| LHFPL3 | blue | TDG | blue | NEDD8 | cyan | COL1A2 | darkorange | DBF4 | grey | LOC101928483 | grey | RP11-468E2.5 | grey | SOS1 | salmon |
| LHFPL3-AS2 | blue | TDRP | blue | NEK7 | cyan | COL3A1 | darkorange | DBIL5P | grey | LOC101928487 | grey | RP11-469M7.1 | grey | SOSTDC1 | salmon |
| LHX1 | blue | TECTA | blue | NELFCD | cyan | COL5A1 | darkorange | DBIL5P2 | grey | LOC101928491 | grey | RP11-470M17.2 | grey | SRPX2 | salmon |
| LHX4 | blue | TEKT1 | blue | NEMF | cyan | COL5A2 | darkorange | DCBLD1 | grey | LOC101928496 | grey | RP11-471G13.5 | grey | SYNE3 | salmon |
| LHX5 | blue | TEKT2 | blue | NET1 | cyan | COL6A3 | darkorange | DCHS1 | grey | LOC101928516 | grey | RP11-474P2.2 | grey | SYTL4 | salmon |
| LHX9 | blue | TEKT5 | blue | NFAT5 | cyan | COLEC12 | darkorange | DCHS2 | grey | LOC101928525 | grey | RP11-476D10.1 | grey | TBC1D2B | salmon |
| LILRA2 | blue | TELO2 | blue | NFIA | cyan | COPS8 | darkorange | DCK | grey | LOC101928537 | grey | RP11-477N3.1 | grey | TFAP2B | salmon |
| LILRA3 | blue | TET1 | blue | NFIB | cyan | CORO1C | darkorange | DCLRE1A | grey | LOC101928559 | grey | RP11-480A16.1 | grey | THRSP | salmon |
| LILRA4 | blue | TET2 | blue | NFIC | cyan | CPA3 | darkorange | DCPS | grey | LOC101928560 | grey | RP11-483C6.1 | grey | TIMP4 | salmon |
| LILRA5 | blue | TEX101 | blue | NFKB1 | cyan | CPE | darkorange | DCST1 | grey | LOC101928565 | grey | RP11-48B3.4 | grey | TKT | salmon |
| LILRP2 | blue | TEX11 | blue | NFU1 | cyan | CPEB3 | darkorange | DCST2 | grey | LOC101928626 | grey | RP11-490G2.2 | grey | TLN2 | salmon |
| LIM2 | blue | TEX13A | blue | NFYB | cyan | CPED1 | darkorange | DCSTAMP | grey | LOC101928635 | grey | RP11-493L12.3 | grey | TMEM132C | salmon |
| LIME1 | blue | TEX19 | blue | NGFRAP1 | cyan | CPNE2 | darkorange | DCTN1 | grey | LOC101928661 | grey | RP11-495K9.3 | grey | TMEM140 | salmon |
| LIN37 | blue | TEX264 | blue | NGRN | cyan | CPQ | darkorange | DCTN1-AS1 | grey | LOC101928663 | grey | RP11-495K9.5 | grey | TMEM37 | salmon |
| LIN7B | blue | TEX28 | blue | NHS | cyan | CPXM2 | darkorange | DCTN3 | grey | LOC101928666 | grey | RP11-495P10.9 | grey | TPRG1 | salmon |
| LIN9 | blue | TEX29 | blue | NIPA2 | cyan | CRTAP | darkorange | DCTPP1 | grey | LOC101928668 | grey | RP11-498C9.17 | grey | TRHDE-AS1 | salmon |
| LINC00028 | blue | TEX35 | blue | NIPAL2 | cyan | CSF1 | darkorange | DCXR | grey | LOC101928694 | grey | RP11-49K24.4 | grey | TRIM8 | salmon |
| LINC00029 | blue | TEX40 | blue | NIPAL4 | cyan | CSF1R | darkorange | DDAH2 | grey | LOC101928708 | grey | RP11-4M23.7 | grey | TUSC5 | salmon |
| LINC00092 | blue | TFAP2D | blue | NIPSNAP3A | cyan | CSGALNACT1 | darkorange | DDB1 | grey | LOC101928710 | grey | RP11-502N13.2 | grey | VKORC1L1 | salmon |
| LINC00162 | blue | TFAP4 | blue | NKIRAS1 | cyan | CSRP2 | darkorange | DDB2 | grey | LOC101928725 | grey | RP11-503C24.6 | grey |  |  |
| LINC00163 | blue | TFB2M | blue | NKTR | cyan | CTSC | darkorange | DDIAS | grey | LOC101928730 | grey | RP11-505K9.4 | grey |  |  |

**Supplementary Table S4.**  80 GO biological process.

| **ID** | **Description** | **GeneRatio** | **pvalue** | **p.adjust** | **qvalue** | **geneID** | **Count** |
| --- | --- | --- | --- | --- | --- | --- | --- |
| GO:0090287 | regulation of cellular response to growth factor stimulus | 8/115 | <0.001 | <0.001 | 0.409 | SNX6/SKI/ADAMTSL2/SDCBP/RBPJ/CYFIP1/SFRP5/NPTN | 8 |
| GO:0006879 | cellular iron ion homeostasis | 4/115 | 0.001 | 0.001 | 0.409 | SKP1/NDFIP1/ISCU/NCOA4 | 4 |
| GO:0060428 | lung epithelium development | 3/115 | 0.001 | 0.001 | 0.409 | ADAMTSL2/RBPJ/FOXJ1 | 3 |
| GO:0055072 | iron ion homeostasis | 4/115 | 0.001 | 0.001 | 0.409 | SKP1/NDFIP1/ISCU/NCOA4 | 4 |
| GO:0007163 | establishment or maintenance of cell polarity | 6/115 | 0.002 | 0.002 | 0.409 | SFRP5/ARF4/ACTL8/SPN/FOXJ1/RHOBTB3 | 6 |
| GO:0045088 | regulation of innate immune response | 9/115 | 0.002 | 0.002 | 0.409 | PIAS1/ADAR/LY96/SKP1/WDFY1/IFI16/CTSB/UBQLN1/JAK1 | 9 |
| GO:0048013 | ephrin receptor signaling pathway | 4/115 | 0.002 | 0.002 | 0.409 | NCK2/RASA1/SDCBP/RBPJ | 4 |
| GO:0061314 | Notch signaling involved in heart development | 2/115 | 0.002 | 0.002 | 0.409 | RBPJ/NOTCH2 | 2 |
| GO:0070106 | interleukin-27-mediated signaling pathway | 2/115 | 0.002 | 0.002 | 0.409 | CRLF1/JAK1 | 2 |
| GO:0014009 | glial cell proliferation | 3/115 | 0.004 | 0.004 | 0.416 | SKI/IL34/PPP1CC | 3 |
| GO:0002643 | regulation of tolerance induction | 2/115 | 0.004 | 0.004 | 0.416 | PDCD1/FOXJ1 | 2 |
| GO:0072659 | protein localization to plasma membrane | 6/115 | 0.005 | 0.005 | 0.416 | EFR3A/LYPLA1/NKD2/GORASP2/PRAM1/STAC3 | 6 |
| GO:0030521 | androgen receptor signaling pathway | 3/115 | 0.006 | 0.006 | 0.416 | PIAS1/DDX17/NCOA4 | 3 |
| GO:0032434 | regulation of proteasomal ubiquitin-dependent protein catabolic process | 4/115 | 0.007 | 0.007 | 0.416 | PIAS1/SDCBP/NKD2/UBQLN1 | 4 |
| GO:0006893 | Golgi to plasma membrane transport | 3/115 | 0.007 | 0.007 | 0.416 | LYPLA1/ARF4/ARF3 | 3 |
| GO:0031331 | positive regulation of cellular catabolic process | 7/115 | 0.007 | 0.007 | 0.416 | PIAS1/FTO/BNIP3L/NKD2/UBQLN1/DAPK1/PTTG1IP | 7 |
| GO:0051101 | regulation of DNA binding | 4/115 | 0.007 | 0.007 | 0.416 | SKI/IFI16/SUMO3/PINX1 | 4 |
| GO:0003177 | pulmonary valve development | 2/115 | 0.007 | 0.007 | 0.416 | RBPJ/NOTCH2 | 2 |
| GO:0006622 | protein targeting to lysosome | 2/115 | 0.007 | 0.007 | 0.416 | SCARB2/NCOA4 | 2 |
| GO:0016553 | base conversion or substitution editing | 2/115 | 0.007 | 0.007 | 0.416 | ADAR/ADAD2 | 2 |
| GO:0051348 | negative regulation of transferase activity | 6/115 | 0.008 | 0.008 | 0.416 | SNX6/ADAR/DUSP6/YWHAG/PINX1/SH2B3 | 6 |
| GO:0051349 | positive regulation of lyase activity | 2/115 | 0.009 | 0.009 | 0.416 | GUCA2A/ISCU | 2 |
| GO:1903322 | positive regulation of protein modification by small protein conjugation or removal | 4/115 | 0.010 | 0.010 | 0.416 | PIAS1/NDFIP1/UBQLN1/PTTG1IP | 4 |
| GO:0002507 | tolerance induction | 2/115 | 0.010 | 0.010 | 0.416 | PDCD1/FOXJ1 | 2 |
| GO:0043312 | neutrophil degranulation | 8/115 | 0.010 | 0.010 | 0.416 | DEGS1/SDCBP/CTSB/CYFIP1/TMEM30A/CTSA/PRAM1/S100A11 | 8 |
| GO:0002283 | neutrophil activation involved in immune response | 8/115 | 0.011 | 0.011 | 0.416 | DEGS1/SDCBP/CTSB/CYFIP1/TMEM30A/CTSA/PRAM1/S100A11 | 8 |
| GO:0015914 | phospholipid transport | 3/115 | 0.011 | 0.011 | 0.416 | PITPNB/SCARB2/TMEM30A | 3 |
| GO:0042119 | neutrophil activation | 8/115 | 0.012 | 0.012 | 0.416 | DEGS1/SDCBP/CTSB/CYFIP1/TMEM30A/CTSA/PRAM1/S100A11 | 8 |
| GO:0007176 | regulation of epidermal growth factor-activated receptor activity | 2/115 | 0.012 | 0.012 | 0.416 | SNX6/NCK2 | 2 |
| GO:0002446 | neutrophil mediated immunity | 8/115 | 0.012 | 0.012 | 0.416 | DEGS1/SDCBP/CTSB/CYFIP1/TMEM30A/CTSA/PRAM1/S100A11 | 8 |
| GO:1990778 | protein localization to cell periphery | 6/115 | 0.013 | 0.013 | 0.416 | EFR3A/LYPLA1/NKD2/GORASP2/PRAM1/STAC3 | 6 |
| GO:0010390 | histone monoubiquitination | 2/115 | 0.014 | 0.014 | 0.416 | SKP1/UBE2E1 | 2 |
| GO:0008360 | regulation of cell shape | 4/115 | 0.014 | 0.014 | 0.416 | RASA1/CYFIP1/ANXA7/RHOBTB3 | 4 |
| GO:0050777 | negative regulation of immune response | 4/115 | 0.014 | 0.014 | 0.416 | ADAR/IFI16/PDCD1/FOXJ1 | 4 |
| GO:0001890 | placenta development | 4/115 | 0.015 | 0.015 | 0.416 | ANG/DNAJB6/RBPJ/CTSB | 4 |
| GO:1903829 | positive regulation of cellular protein localization | 6/115 | 0.015 | 0.015 | 0.416 | YWHAG/TMEM30A/NPTN/NKD2/PINX1/STAC3 | 6 |
| GO:0006469 | negative regulation of protein kinase activity | 5/115 | 0.015 | 0.015 | 0.416 | SNX6/ADAR/DUSP6/YWHAG/SH2B3 | 5 |
| GO:0010506 | regulation of autophagy | 6/115 | 0.016 | 0.016 | 0.416 | SNX6/IFI16/BNIP3L/UBQLN1/DAPK1/CTSA | 6 |
| GO:0009896 | positive regulation of catabolic process | 7/115 | 0.016 | 0.016 | 0.416 | PIAS1/FTO/BNIP3L/NKD2/UBQLN1/DAPK1/PTTG1IP | 7 |
| GO:0007259 | JAK-STAT cascade | 4/115 | 0.016 | 0.016 | 0.416 | CRLF3/PIAS1/CRLF1/SH2B3 | 4 |
| GO:0048741 | skeletal muscle fiber development | 2/115 | 0.017 | 0.017 | 0.416 | SKI/STAC3 | 2 |
| GO:0030510 | regulation of BMP signaling pathway | 3/115 | 0.019 | 0.019 | 0.416 | SKI/RBPJ/SFRP5 | 3 |
| GO:0060251 | regulation of glial cell proliferation | 2/115 | 0.020 | 0.020 | 0.416 | SKI/PPP1CC | 2 |
| GO:0030224 | monocyte differentiation | 2/115 | 0.021 | 0.021 | 0.416 | IFI16/IL34 | 2 |
| GO:1903131 | mononuclear cell differentiation | 2/115 | 0.021 | 0.021 | 0.416 | IFI16/IL34 | 2 |
| GO:0098876 | vesicle-mediated transport to the plasma membrane | 3/115 | 0.021 | 0.021 | 0.416 | LYPLA1/ARF4/ARF3 | 3 |
| GO:0060323 | head morphogenesis | 2/115 | 0.022 | 0.022 | 0.416 | SKI/SSBP3 | 2 |
| GO:1905314 | semi-lunar valve development | 2/115 | 0.022 | 0.022 | 0.416 | RBPJ/NOTCH2 | 2 |
| GO:0001659 | temperature homeostasis | 4/115 | 0.022 | 0.022 | 0.416 | RBPJ/FTO/LPIN1/PDGFC | 4 |
| GO:1905898 | positive regulation of response to endoplasmic reticulum stress | 2/115 | 0.023 | 0.023 | 0.416 | NCK2/UBQLN1 | 2 |
| GO:0001666 | response to hypoxia | 6/115 | 0.024 | 0.024 | 0.416 | ANG/RBPJ/BNIP3L/ALDH3A1/UBQLN1/HIGD1A | 6 |
| GO:0002683 | negative regulation of immune system process | 7/115 | 0.024 | 0.024 | 0.416 | ADAR/LY96/IFI16/PDCD1/UBQLN1/SPN/FOXJ1 | 7 |
| GO:0018108 | peptidyl-tyrosine phosphorylation | 6/115 | 0.025 | 0.025 | 0.416 | SNX6/NCK2/CRLF1/JAK1/SH2B3/PDGFC | 6 |
| GO:0030041 | actin filament polymerization | 4/115 | 0.026 | 0.026 | 0.416 | NCK2/RASA1/ANG/CYFIP1 | 4 |
| GO:0015748 | organophosphate ester transport | 3/115 | 0.027 | 0.027 | 0.416 | PITPNB/SCARB2/TMEM30A | 3 |
| GO:0042149 | cellular response to glucose starvation | 2/115 | 0.028 | 0.028 | 0.416 | IFI16/HIGD1A | 2 |
| GO:1905475 | regulation of protein localization to membrane | 4/115 | 0.029 | 0.029 | 0.416 | LYPLA1/YWHAG/NKD2/STAC3 | 4 |
| GO:0046460 | neutral lipid biosynthetic process | 2/115 | 0.029 | 0.029 | 0.416 | ANG/LPIN1 | 2 |
| GO:0046463 | acylglycerol biosynthetic process | 2/115 | 0.029 | 0.029 | 0.416 | ANG/LPIN1 | 2 |
| GO:0060759 | regulation of response to cytokine stimulus | 4/115 | 0.030 | 0.030 | 0.416 | PIAS1/ADAR/JAK1/SH2B3 | 4 |
| GO:0061756 | leukocyte adhesion to vascular endothelial cell | 2/115 | 0.030 | 0.030 | 0.416 | NFAT5/SPN | 2 |
| GO:0032970 | regulation of actin filament-based process | 6/115 | 0.033 | 0.033 | 0.416 | NCK2/RASA1/CYFIP1/NOTCH2/FRMD6/RHOBTB3 | 6 |
| GO:0006914 | autophagy | 7/115 | 0.034 | 0.034 | 0.416 | SNX6/IFI16/BNIP3L/UBQLN1/DAPK1/CTSA/ANXA7 | 7 |
| GO:0061919 | process utilizing autophagic mechanism | 7/115 | 0.034 | 0.034 | 0.416 | SNX6/IFI16/BNIP3L/UBQLN1/DAPK1/CTSA/ANXA7 | 7 |
| GO:0008542 | visual learning | 2/115 | 0.035 | 0.035 | 0.416 | PIAS1/NPTN | 2 |
| GO:0072666 | establishment of protein localization to vacuole | 2/115 | 0.035 | 0.035 | 0.416 | SCARB2/NCOA4 | 2 |
| GO:0022407 | regulation of cell-cell adhesion | 6/115 | 0.039 | 0.039 | 0.416 | NCK2/NFAT5/PDCD1/SPN/FOXJ1/SH2B3 | 6 |
| GO:0043618 | regulation of transcription from RNA polymerase II promoter in response to stress | 3/115 | 0.039 | 0.039 | 0.416 | NCK2/RBPJ/HIGD1A | 3 |
| GO:0071456 | cellular response to hypoxia | 4/115 | 0.039 | 0.039 | 0.416 | RBPJ/BNIP3L/UBQLN1/HIGD1A | 4 |
| GO:1905477 | positive regulation of protein localization to membrane | 3/115 | 0.040 | 0.040 | 0.416 | YWHAG/NKD2/STAC3 | 3 |
| GO:0008637 | apoptotic mitochondrial changes | 3/115 | 0.041 | 0.041 | 0.416 | YWHAG/BNIP3L/HIGD1A | 3 |
| GO:0006968 | cellular defense response | 2/115 | 0.042 | 0.042 | 0.416 | LY96/SPN | 2 |
| GO:0046486 | glycerolipid metabolic process | 6/115 | 0.043 | 0.043 | 0.416 | EFR3A/ANG/GDE1/LPIN1/PLCH2/ARF3 | 6 |
| GO:0097345 | mitochondrial outer membrane permeabilization | 2/115 | 0.044 | 0.044 | 0.416 | YWHAG/BNIP3L | 2 |
| GO:0006120 | mitochondrial electron transport, NADH to ubiquinone | 2/115 | 0.045 | 0.045 | 0.416 | NDUFA12/ISCU | 2 |
| GO:0009952 | anterior/posterior pattern specification | 4/115 | 0.047 | 0.047 | 0.416 | SKI/BARX1/RBPJ/SSBP3 | 4 |
| GO:0048762 | mesenchymal cell differentiation | 4/115 | 0.047 | 0.047 | 0.416 | SDCBP/RBPJ/SEMA3B/DDX17 | 4 |
| GO:0045981 | positive regulation of nucleotide metabolic process | 2/115 | 0.048 | 0.048 | 0.416 | GUCA2A/ISCU | 2 |
| GO:1900544 | positive regulation of purine nucleotide metabolic process | 2/115 | 0.048 | 0.048 | 0.416 | GUCA2A/ISCU | 2 |
| GO:0061951 | establishment of protein localization to plasma membrane | 2/115 | 0.050 | 0.050 | 0.416 | LYPLA1/GORASP2 | 2 |

**Supplementary Table S5.** 9 GO cellular component.

| ID | Description | GeneRatio | pvalue | p.adjust | qvalue | geneID | Count |
| --- | --- | --- | --- | --- | --- | --- | --- |
| GO:0043202 | lysosomal lumen | 4/115 | 0.002 | 0.002 | 0.273 | CTSB/SCARB2/CTSA/LIPA | 4 |
| GO:0016605 | PML body | 4/115 | 0.003 | 0.003 | 0.273 | PIAS1/SKI/SUMO3/SPN | 4 |
| GO:0010008 | endosome membrane | 8/115 | 0.007 | 0.007 | 0.305 | SNX6/LY96/PLEKHF2/NDFIP1/SCARB2/ITM2B/TMEM9B/KIAA0319 | 8 |
| GO:0005925 | focal adhesion | 7/115 | 0.010 | 0.010 | 0.305 | SDCBP/YWHAG/CYFIP1/SCARB2/LASP1/JAK1/PPP1CC | 7 |
| GO:0030055 | cell-substrate junction | 7/115 | 0.011 | 0.011 | 0.305 | SDCBP/YWHAG/CYFIP1/SCARB2/LASP1/JAK1/PPP1CC | 7 |
| GO:0001726 | ruffle | 4/115 | 0.018 | 0.018 | 0.431 | RASA1/CYFIP1/ARF4/S100A11 | 4 |
| GO:0099738 | cell cortex region | 2/115 | 0.025 | 0.025 | 0.431 | ACTL8/MYL12B | 2 |
| GO:0000775 | chromosome, centromeric region | 4/115 | 0.027 | 0.027 | 0.431 | CBX1/SUMO3/PINX1/PPP1CC | 4 |
| GO:0000784 | nuclear chromosome, telomeric region | 3/115 | 0.037 | 0.037 | 0.431 | CBX1/PINX1/PPP1CC | 3 |

**Supplementary Table S6.** 21 GO molecular function.

| ID | Description | GeneRatio | pvalue | p.adjust | qvalue | geneID | Count |
| --- | --- | --- | --- | --- | --- | --- | --- |
| GO:0008093 | cytoskeletal adaptor activity | 3/109 | <0.001 | <0.001 | 0.032 | NCK2/SDCBP/ACTL8 | 3 |
| GO:0004000 | adenosine deaminase activity | 2/109 | 0.002 | 0.002 | 0.152 | ADAR/ADAD2 | 2 |
| GO:0005548 | phospholipid transporter activity | 3/109 | 0.004 | 0.004 | 0.152 | PITPNB/SCARB2/TMEM30A | 3 |
| GO:0005521 | lamin binding | 2/109 | 0.004 | 0.004 | 0.152 | BNIP3L/PPP1CC | 2 |
| GO:0047485 | protein N-terminus binding | 4/109 | 0.005 | 0.005 | 0.152 | SDCBP/RBPJ/PPP1CC/PEX19 | 4 |
| GO:0004622 | lysophospholipase activity | 2/109 | 0.006 | 0.006 | 0.176 | LYPLA1/GDE1 | 2 |
| GO:0070851 | growth factor receptor binding | 4/109 | 0.009 | 0.009 | 0.208 | SDCBP/NPTN/ARF4/PDGFC | 4 |
| GO:0008198 | ferrous iron binding | 2/109 | 0.009 | 0.009 | 0.208 | FTO/ISCU | 2 |
| GO:0003725 | double-stranded RNA binding | 3/109 | 0.011 | 0.011 | 0.209 | ADAR/ADAD2/ZFR | 3 |
| GO:0031210 | phosphatidylcholine binding | 2/109 | 0.012 | 0.012 | 0.209 | PITPNB/SCARB2 | 2 |
| GO:0050997 | quaternary ammonium group binding | 2/109 | 0.012 | 0.012 | 0.209 | PITPNB/SCARB2 | 2 |
| GO:0005543 | phospholipid binding | 7/109 | 0.016 | 0.016 | 0.242 | SNX6/PLEKHF2/WDFY1/SDCBP/PITPNB/SCARB2/ANXA7 | 7 |
| GO:0019239 | deaminase activity | 2/109 | 0.017 | 0.017 | 0.242 | ADAR/ADAD2 | 2 |
| GO:0043394 | proteoglycan binding | 2/109 | 0.021 | 0.021 | 0.259 | SDCBP/CTSB | 2 |
| GO:0001784 | phosphotyrosine residue binding | 2/109 | 0.025 | 0.025 | 0.288 | NCK2/RASA1 | 2 |
| GO:0005525 | GTP binding | 6/109 | 0.028 | 0.028 | 0.288 | SUCLG2/ARF4/DAPK1/RHOBTB3/ARF3/ARL15 | 6 |
| GO:0032550 | purine ribonucleoside binding | 6/109 | 0.029 | 0.029 | 0.288 | SUCLG2/ARF4/DAPK1/RHOBTB3/ARF3/ARL15 | 6 |
| GO:0050681 | androgen receptor binding | 2/109 | 0.030 | 0.030 | 0.288 | PIAS1/NCOA4 | 2 |
| GO:0098632 | cell-cell adhesion mediator activity | 2/109 | 0.038 | 0.038 | 0.303 | NPTN/S100A11 | 2 |
| GO:0044389 | ubiquitin-like protein ligase binding | 5/109 | 0.042 | 0.042 | 0.315 | PIAS1/SKI/SUMO3/NKD2/JAK1 | 5 |
| GO:0003713 | transcription coactivator activity | 5/109 | 0.047 | 0.047 | 0.336 | PIAS1/DDX17/NR2C2/LPIN1/NCOA4 | 5 |

**Supplementary Table S7.** 7 KEGG pathway

| ID | Description | GeneRatio | pvalue | p.adjust | qvalue | geneID | Count |
| --- | --- | --- | --- | --- | --- | --- | --- |
| hsa04360 | Axon guidance | 5/58 | 0.009 | 0.009 | 0.695 | NTN4/NCK2/RASA1/SEMA3B/MYL12B | 5 |
| hsa04142 | Lysosome | 4/58 | 0.014 | 0.014 | 0.695 | CTSB/SCARB2/CTSA/LIPA | 4 |
| hsa04810 | Regulation of actin cytoskeleton | 5/58 | 0.019 | 0.019 | 0.695 | GNG12/CYFIP1/MYL12B/PPP1CC/PDGFC | 5 |
| hsa04390 | Hippo signaling pathway | 4/58 | 0.026 | 0.026 | 0.695 | YWHAG/FRMD6/NKD2/PPP1CC | 4 |
| hsa05160 | Hepatitis C | 4/58 | 0.026 | 0.026 | 0.695 | PIAS1/CLDN14/YWHAG/JAK1 | 4 |
| hsa04658 | Th1 and Th2 cell differentiation | 3/58 | 0.028 | 0.028 | 0.695 | RBPJ/NOTCH2/JAK1 | 3 |
| hsa00600 | Sphingolipid metabolism | 2/58 | 0.048 | 0.048 | 0.778 | DEGS1/SGMS2 | 2 |

**Supplementary Table S****8.** The 64 protein-interaction pairs contaomomg 64 protein nodes of the PPI network.

| **node1** | **node2** | **node1_string_internal_id** | **node2_string_internal_id** | **node1_external_id** | **node2_external_id** | **neighborhood_on_chromosome** | **gene_fusion** | **phylogenetic_cooccurrence** | **homology** | **coexpression** | **experimentally_determined_interaction** | **database_annotated** | **automated_textmining** | **combined_score** |
| --- | --- | --- | --- | --- | --- | --- | --- | --- | --- | --- | --- | --- | --- | --- |
| RBPJ | NOTCH2 | 4441499 | 4434503 | 9606.ENSP00000345206 | 9606.ENSP00000256646 | 0 | 0 | 0 | 0 | 0.063 | 0.814 | 0.9 | 0.905 | 0.998 |
| ATP5C1 | ATP5J2 | 4441891 | 4436969 | 9606.ENSP00000349142 | 9606.ENSP00000292475 | 0 | 0 | 0 | 0 | 0.889 | 0.084 | 0.9 | 0.541 | 0.994 |
| SUMO3 | PIAS1 | 4448523 | 4434102 | 9606.ENSP00000409666 | 9606.ENSP00000249636 | 0 | 0 | 0 | 0 | 0 | 0.494 | 0.9 | 0.633 | 0.979 |
| ATP5C1 | NDUFA12 | 4441891 | 4440206 | 9606.ENSP00000349142 | 9606.ENSP00000330737 | 0 | 0 | 0 | 0 | 0.868 | 0.818 | 0 | 0 | 0.975 |
| SGMS2 | DEGS1 | 4446303 | 4439057 | 9606.ENSP00000378176 | 9606.ENSP00000316476 | 0 | 0 | 0 | 0 | 0.048 | 0 | 0.9 | 0.657 | 0.964 |
| JAK1 | PIAS1 | 4441317 | 4434102 | 9606.ENSP00000343204 | 9606.ENSP00000249636 | 0 | 0 | 0 | 0 | 0.061 | 0.183 | 0.9 | 0.414 | 0.949 |
| UBE2E1 | SKP1 | 4437986 | 4433537 | 9606.ENSP00000303709 | 9606.ENSP00000231487 | 0 | 0 | 0 | 0 | 0.053 | 0.261 | 0.9 | 0.246 | 0.94 |
| CRLF1 | JAK1 | 4446015 | 4441317 | 9606.ENSP00000376188 | 9606.ENSP00000343204 | 0 | 0 | 0 | 0 | 0 | 0.072 | 0.9 | 0.292 | 0.928 |
| JAK1 | UBE2E1 | 4441317 | 4437986 | 9606.ENSP00000343204 | 9606.ENSP00000303709 | 0 | 0 | 0 | 0 | 0 | 0.183 | 0.9 | 0.174 | 0.926 |
| NDUFA12 | ATP5J2 | 4440206 | 4436969 | 9606.ENSP00000330737 | 9606.ENSP00000292475 | 0 | 0 | 0 | 0 | 0.876 | 0 | 0 | 0.384 | 0.92 |
| RBPJ | SKP1 | 4441499 | 4433537 | 9606.ENSP00000345206 | 9606.ENSP00000231487 | 0 | 0 | 0 | 0 | 0.045 | 0 | 0.9 | 0.169 | 0.913 |
| ARF4 | ARF3 | 4438185 | 4434505 | 9606.ENSP00000306010 | 9606.ENSP00000256682 | 0 | 0 | 0.448 | 0.978 | 0.063 | 0 | 0.9 | 0.714 | 0.904 |
| DEGS1 | TMEM30A | 4439057 | 4433504 | 9606.ENSP00000316476 | 9606.ENSP00000230461 | 0 | 0 | 0 | 0 | 0.061 | 0 | 0.9 | 0 | 0.902 |
| KIAA0319 | SCARB2 | 4445026 | 4435511 | 9606.ENSP00000367459 | 9606.ENSP00000264896 | 0 | 0 | 0 | 0 | 0 | 0 | 0.9 | 0 | 0.9 |
| CTSA | SDCBP | 4443947 | 4434752 | 9606.ENSP00000361562 | 9606.ENSP00000260130 | 0 | 0 | 0 | 0 | 0 | 0 | 0.9 | 0 | 0.9 |
| NDUFA12 | MTCH2 | 4440206 | 4437945 | 9606.ENSP00000330737 | 9606.ENSP00000303222 | 0 | 0 | 0 | 0 | 0.185 | 0.866 | 0 | 0.116 | 0.895 |
| MYL12B | PPP1CC | 4450713 | 4441182 | 9606.ENSP00000463559 | 9606.ENSP00000341779 | 0 | 0 | 0 | 0 | 0.055 | 0.187 | 0.8 | 0.113 | 0.845 |
| ATP5C1 | MTCH2 | 4441891 | 4437945 | 9606.ENSP00000349142 | 9606.ENSP00000303222 | 0 | 0 | 0 | 0 | 0.123 | 0.821 | 0 | 0.052 | 0.838 |
| YWHAG | SKP1 | 4438211 | 4433537 | 9606.ENSP00000306330 | 9606.ENSP00000231487 | 0 | 0 | 0 | 0 | 0.07 | 0.745 | 0 | 0.108 | 0.77 |
| FTO | MTCH2 | 4448992 | 4437945 | 9606.ENSP00000418823 | 9606.ENSP00000303222 | 0 | 0 | 0 | 0 | 0 | 0 | 0 | 0.732 | 0.732 |
| ANXA7 | SNX6 | 4444056 | 4442632 | 9606.ENSP00000362010 | 9606.ENSP00000355217 | 0 | 0 | 0 | 0 | 0.072 | 0 | 0 | 0.67 | 0.68 |
| DDX17 | CIRH1A | 4446598 | 4439903 | 9606.ENSP00000380033 | 9606.ENSP00000327179 | 0 | 0 | 0 | 0 | 0.652 | 0 | 0 | 0.044 | 0.653 |
| DAPK1 | PRR15 | 4447310 | 4439154 | 9606.ENSP00000386135 | 9606.ENSP00000317836 | 0 | 0 | 0 | 0 | 0 | 0 | 0 | 0.644 | 0.644 |
| CTSB | ZFR | 4441541 | 4435547 | 9606.ENSP00000345672 | 9606.ENSP00000265069 | 0 | 0 | 0 | 0 | 0 | 0 | 0 | 0.643 | 0.643 |
| SUCLG2 | CIRH1A | 4449031 | 4439903 | 9606.ENSP00000419325 | 9606.ENSP00000327179 | 0 | 0 | 0 | 0 | 0 | 0 | 0 | 0.641 | 0.641 |
| CTSA | CTSB | 4443947 | 4441541 | 9606.ENSP00000361562 | 9606.ENSP00000345672 | 0 | 0 | 0 | 0 | 0.242 | 0 | 0 | 0.529 | 0.627 |
| DDX17 | ADAR | 4446598 | 4443107 | 9606.ENSP00000380033 | 9606.ENSP00000357459 | 0 | 0 | 0 | 0 | 0.089 | 0.392 | 0 | 0.336 | 0.6 |
| CYFIP1 | NCK2 | 4451554 | 4433581 | 9606.ENSP00000481038 | 9606.ENSP00000233154 | 0 | 0 | 0 | 0 | 0 | 0.511 | 0 | 0.173 | 0.578 |
| MRPS6 | SON | 4446909 | 4441880 | 9606.ENSP00000382250 | 9606.ENSP00000348984 | 0 | 0 | 0 | 0 | 0 | 0 | 0 | 0.566 | 0.566 |
| LASP1 | S100A11 | 4439747 | 4435987 | 9606.ENSP00000325240 | 9606.ENSP00000271638 | 0 | 0 | 0 | 0 | 0.063 | 0 | 0 | 0.526 | 0.537 |
| SSBP3 | CRLF3 | 4443722 | 4439226 | 9606.ENSP00000360371 | 9606.ENSP00000318804 | 0 | 0 | 0 | 0 | 0 | 0 | 0 | 0.535 | 0.535 |
| ARF4 | NCK2 | 4438185 | 4433581 | 9606.ENSP00000306010 | 9606.ENSP00000233154 | 0 | 0 | 0 | 0 | 0 | 0.064 | 0 | 0.505 | 0.516 |
| FTO | ZMAT3 | 4448992 | 4438666 | 9606.ENSP00000418823 | 9606.ENSP00000311221 | 0 | 0 | 0 | 0 | 0 | 0.513 | 0 | 0 | 0.513 |
| TBC1D23 | RHOBTB3 | 4446233 | 4445299 | 9606.ENSP00000377700 | 9606.ENSP00000369318 | 0 | 0 | 0 | 0 | 0.055 | 0 | 0 | 0.504 | 0.512 |
| CTSB | APLP2 | 4441541 | 4435272 | 9606.ENSP00000345672 | 9606.ENSP00000263574 | 0 | 0 | 0 | 0 | 0.097 | 0 | 0 | 0.479 | 0.51 |
| NPTN | EFR3A | 4436864 | 4434368 | 9606.ENSP00000290401 | 9606.ENSP00000254624 | 0 | 0 | 0 | 0 | 0.082 | 0 | 0 | 0.481 | 0.504 |
| MYL12B | DAPK1 | 4450713 | 4447310 | 9606.ENSP00000463559 | 9606.ENSP00000386135 | 0 | 0 | 0 | 0 | 0.05 | 0.472 | 0 | 0.085 | 0.501 |
| LASP1 | RASA1 | 4439747 | 4436131 | 9606.ENSP00000325240 | 9606.ENSP00000274376 | 0 | 0 | 0 | 0 | 0 | 0 | 0 | 0.5 | 0.5 |
| ITM2B | APLP2 | 4445077 | 4435272 | 9606.ENSP00000367828 | 9606.ENSP00000263574 | 0 | 0 | 0 | 0 | 0.09 | 0.157 | 0 | 0.39 | 0.492 |
| SUMO3 | SKP1 | 4448523 | 4433537 | 9606.ENSP00000409666 | 9606.ENSP00000231487 | 0 | 0 | 0 | 0 | 0.122 | 0 | 0 | 0.442 | 0.489 |
| SON | RBPJ | 4441880 | 4441499 | 9606.ENSP00000348984 | 9606.ENSP00000345206 | 0 | 0 | 0 | 0 | 0.101 | 0 | 0 | 0.45 | 0.484 |
| SON | PPP1CC | 4441880 | 4441182 | 9606.ENSP00000348984 | 9606.ENSP00000341779 | 0 | 0 | 0 | 0 | 0.064 | 0 | 0 | 0.469 | 0.481 |
| FTO | NOTCH2 | 4448992 | 4434503 | 9606.ENSP00000418823 | 9606.ENSP00000256646 | 0 | 0 | 0 | 0 | 0 | 0 | 0 | 0.479 | 0.479 |
| CTSB | JAK1 | 4441541 | 4441317 | 9606.ENSP00000345672 | 9606.ENSP00000343204 | 0 | 0 | 0 | 0 | 0.061 | 0 | 0 | 0.463 | 0.475 |
| PPP1CC | ARF4 | 4441182 | 4438185 | 9606.ENSP00000341779 | 9606.ENSP00000306010 | 0 | 0 | 0.364 | 0 | 0.063 | 0.157 | 0 | 0.066 | 0.468 |
| SUCLG2 | ATP5C1 | 4449031 | 4441891 | 9606.ENSP00000419325 | 9606.ENSP00000349142 | 0.137 | 0 | 0 | 0 | 0.282 | 0.063 | 0 | 0.182 | 0.462 |
| UBQLN1 | WDFY1 | 4444723 | 4433574 | 9606.ENSP00000365576 | 9606.ENSP00000233055 | 0 | 0 | 0 | 0 | 0.064 | 0.442 | 0 | 0.049 | 0.46 |
| HIGD1A | ATP5J2 | 4448007 | 4436969 | 9606.ENSP00000398064 | 9606.ENSP00000292475 | 0 | 0 | 0 | 0 | 0.457 | 0 | 0 | 0 | 0.457 |
| NFAT5 | DDX17 | 4450573 | 4446598 | 9606.ENSP00000457593 | 9606.ENSP00000380033 | 0 | 0 | 0 | 0 | 0.082 | 0.328 | 0 | 0.187 | 0.454 |
| SH2B3 | JAK1 | 4441528 | 4441317 | 9606.ENSP00000345492 | 9606.ENSP00000343204 | 0 | 0 | 0 | 0 | 0 | 0.054 | 0 | 0.444 | 0.451 |
| SGMS2 | FRMD6 | 4446303 | 4441376 | 9606.ENSP00000378176 | 9606.ENSP00000343899 | 0 | 0 | 0 | 0 | 0.07 | 0 | 0 | 0.431 | 0.448 |
| LPIN1 | PEX19 | 4447998 | 4443022 | 9606.ENSP00000397908 | 9606.ENSP00000357051 | 0 | 0 | 0 | 0 | 0.049 | 0 | 0 | 0.442 | 0.447 |
| CYFIP1 | FTO | 4451554 | 4448992 | 9606.ENSP00000481038 | 9606.ENSP00000418823 | 0 | 0 | 0 | 0 | 0 | 0 | 0 | 0.446 | 0.446 |
| LYPLA1 | EFR3A | 4439327 | 4434368 | 9606.ENSP00000320043 | 9606.ENSP00000254624 | 0 | 0 | 0 | 0 | 0.065 | 0 | 0 | 0.43 | 0.445 |
| PPP1CC | ARF3 | 4441182 | 4434505 | 9606.ENSP00000341779 | 9606.ENSP00000256682 | 0 | 0 | 0.336 | 0 | 0.063 | 0.157 | 0 | 0.066 | 0.444 |
| LYPLA1 | GORASP2 | 4439327 | 4433617 | 9606.ENSP00000320043 | 9606.ENSP00000234160 | 0 | 0 | 0 | 0 | 0 | 0 | 0 | 0.444 | 0.444 |
| PPP1CC | YWHAG | 4441182 | 4438211 | 9606.ENSP00000341779 | 9606.ENSP00000306330 | 0 | 0 | 0 | 0 | 0.064 | 0.385 | 0 | 0.104 | 0.439 |
| DUSP6 | NCK2 | 4436348 | 4433581 | 9606.ENSP00000279488 | 9606.ENSP00000233154 | 0 | 0 | 0 | 0 | 0 | 0 | 0 | 0.437 | 0.437 |
| MRPS6 | PINX1 | 4446909 | 4439243 | 9606.ENSP00000382250 | 9606.ENSP00000318966 | 0 | 0 | 0 | 0 | 0 | 0 | 0 | 0.429 | 0.429 |
| AFF1 | LASP1 | 4446378 | 4439747 | 9606.ENSP00000378578 | 9606.ENSP00000325240 | 0 | 0 | 0 | 0 | 0 | 0 | 0 | 0.426 | 0.426 |
| DAPK1 | UBQLN1 | 4447310 | 4444723 | 9606.ENSP00000386135 | 9606.ENSP00000365576 | 0 | 0 | 0 | 0 | 0 | 0.087 | 0 | 0.393 | 0.422 |
| UBQLN1 | SKP1 | 4444723 | 4433537 | 9606.ENSP00000365576 | 9606.ENSP00000231487 | 0 | 0 | 0 | 0 | 0.179 | 0.134 | 0 | 0.252 | 0.422 |
| ANXA7 | S100A11 | 4444056 | 4435987 | 9606.ENSP00000362010 | 9606.ENSP00000271638 | 0 | 0 | 0 | 0 | 0.062 | 0.061 | 0 | 0.389 | 0.414 |
| SGMS2 | CTSA | 4446303 | 4443947 | 9606.ENSP00000378176 | 9606.ENSP00000361562 | 0 | 0 | 0 | 0 | 0 | 0.108 | 0 | 0.369 | 0.413 |

**Supplementary Table S9.** The results of the infiltration abundance of 28 immune cell types in all samples in the GSE58331 dataset analyzed by ssGSEA algorithm.

|  | **Activated B cell** | **Activated CD4 T cell** | **Activated CD8 T cell** | **Activated dendritic cell** | **CD56bright natural killer cell** | **CD56dim natural killer cell** | **Central memory CD4 T cell** | **Central memory CD8 T cell** | **Effector memeory CD4 T cell** | **Effector memeory CD8 T cell** | **Eosinophil** | **Gamma delta T cell** | **Immature B cell** | **Immature dendritic cell** | **Macrophage** | **Mast cell** | **MDSC** | **Memory B cell** | **Monocyte** | **Natural killer cell** | **Natural killer T cell** | **Neutrophil** | **Plasmacytoid dendritic cell** | **Regulatory T cell** | **T follicular helper cell** | **Type 1 T helper cell** | **Type 17 T helper cell** | **Type 2 T helper cell** |
| --- | --- | --- | --- | --- | --- | --- | --- | --- | --- | --- | --- | --- | --- | --- | --- | --- | --- | --- | --- | --- | --- | --- | --- | --- | --- | --- | --- | --- |
| GSM1407182 | 0.028 | -0.293 | 0.083 | 0.354 | 0.242 | 0.303 | 0.470 | 0.314 | 0.157 | 0.463 | -0.086 | 0.280 | 0.045 | 0.242 | 0.017 | 0.171 | 0.306 | -0.022 | 0.439 | 0.329 | 0.129 | -0.131 | 0.449 | 0.210 | 0.288 | 0.141 | 0.007 | -0.057 |
| GSM1407183 | -0.092 | -0.247 | 0.026 | 0.331 | 0.294 | 0.261 | 0.504 | 0.306 | 0.254 | 0.450 | -0.082 | 0.330 | -0.008 | 0.328 | 0.003 | 0.137 | 0.341 | 0.047 | 0.378 | 0.324 | 0.129 | -0.114 | 0.454 | 0.224 | 0.298 | 0.129 | -0.076 | 0.041 |
| GSM1407184 | 0.003 | -0.195 | 0.135 | 0.344 | 0.242 | 0.229 | 0.486 | 0.285 | 0.264 | 0.486 | -0.023 | 0.319 | 0.016 | 0.331 | -0.007 | 0.256 | 0.427 | -0.020 | 0.375 | 0.313 | 0.155 | -0.098 | 0.452 | 0.259 | 0.299 | 0.145 | -0.033 | 0.061 |
| GSM1407185 | 0.145 | -0.295 | 0.182 | 0.315 | 0.275 | 0.340 | 0.459 | 0.246 | 0.064 | 0.432 | 0.021 | 0.279 | 0.078 | 0.206 | 0.048 | 0.179 | 0.381 | 0.036 | 0.484 | 0.284 | 0.145 | -0.188 | 0.405 | 0.190 | 0.308 | 0.144 | 0.068 | -0.151 |
| GSM1407186 | -0.076 | -0.266 | 0.049 | 0.349 | 0.271 | 0.259 | 0.494 | 0.319 | 0.244 | 0.465 | -0.079 | 0.358 | -0.011 | 0.329 | -0.007 | 0.208 | 0.376 | 0.025 | 0.404 | 0.343 | 0.157 | -0.151 | 0.477 | 0.287 | 0.319 | 0.126 | -0.055 | 0.009 |
| GSM1407187 | -0.011 | -0.254 | 0.092 | 0.342 | 0.248 | 0.278 | 0.504 | 0.290 | 0.164 | 0.472 | -0.032 | 0.334 | 0.024 | 0.280 | 0.046 | 0.226 | 0.389 | 0.015 | 0.443 | 0.334 | 0.133 | -0.024 | 0.459 | 0.232 | 0.309 | 0.155 | -0.019 | -0.044 |
| GSM1407188 | 0.023 | -0.191 | 0.089 | 0.368 | 0.249 | 0.339 | 0.499 | 0.375 | 0.134 | 0.470 | 0.068 | 0.353 | 0.056 | 0.307 | 0.098 | 0.339 | 0.456 | 0.048 | 0.491 | 0.365 | 0.156 | 0.102 | 0.423 | 0.293 | 0.337 | 0.186 | 0.027 | -0.069 |
| GSM1407189 | 0.040 | -0.260 | 0.134 | 0.349 | 0.236 | 0.370 | 0.481 | 0.341 | 0.115 | 0.441 | 0.049 | 0.312 | 0.010 | 0.269 | 0.037 | 0.220 | 0.379 | 0.039 | 0.481 | 0.350 | 0.152 | -0.121 | 0.410 | 0.210 | 0.285 | 0.172 | 0.024 | -0.059 |
| GSM1407190 | 0.004 | -0.227 | 0.098 | 0.348 | 0.224 | 0.366 | 0.508 | 0.303 | 0.116 | 0.447 | 0.052 | 0.296 | 0.020 | 0.309 | 0.021 | 0.336 | 0.390 | 0.012 | 0.469 | 0.344 | 0.151 | -0.040 | 0.431 | 0.188 | 0.287 | 0.189 | 0.037 | -0.024 |
| GSM1407191 | -0.105 | -0.227 | 0.046 | 0.387 | 0.300 | 0.216 | 0.507 | 0.320 | 0.262 | 0.454 | 0.080 | 0.321 | 0.023 | 0.342 | -0.004 | 0.298 | 0.399 | 0.061 | 0.353 | 0.334 | 0.150 | 0.035 | 0.459 | 0.235 | 0.292 | 0.141 | -0.102 | 0.072 |
| GSM1407192 | 0.100 | -0.296 | 0.138 | 0.313 | 0.220 | 0.382 | 0.491 | 0.310 | 0.085 | 0.454 | 0.038 | 0.300 | 0.030 | 0.264 | 0.047 | 0.173 | 0.348 | 0.004 | 0.473 | 0.347 | 0.140 | -0.116 | 0.405 | 0.203 | 0.303 | 0.171 | 0.007 | -0.052 |
| GSM1407193 | 0.125 | -0.305 | 0.141 | 0.295 | 0.230 | 0.421 | 0.437 | 0.297 | 0.026 | 0.405 | -0.014 | 0.286 | 0.034 | 0.202 | 0.064 | 0.174 | 0.335 | 0.016 | 0.471 | 0.312 | 0.130 | -0.092 | 0.365 | 0.213 | 0.271 | 0.175 | 0.060 | -0.134 |
| GSM1407194 | 0.081 | -0.294 | 0.122 | 0.328 | 0.235 | 0.394 | 0.459 | 0.298 | 0.074 | 0.450 | 0.046 | 0.295 | 0.047 | 0.254 | 0.090 | 0.205 | 0.369 | 0.010 | 0.482 | 0.341 | 0.152 | -0.023 | 0.394 | 0.215 | 0.295 | 0.160 | 0.039 | -0.083 |
| GSM1407195 | 0.352 | -0.121 | 0.301 | 0.355 | 0.264 | 0.387 | 0.494 | 0.321 | 0.063 | 0.476 | -0.098 | 0.226 | 0.128 | 0.254 | 0.017 | 0.086 | 0.434 | 0.037 | 0.485 | 0.317 | 0.129 | -0.181 | 0.449 | 0.188 | 0.268 | 0.156 | 0.020 | -0.083 |
| GSM1407196 | 0.340 | -0.092 | 0.264 | 0.390 | 0.268 | 0.324 | 0.536 | 0.324 | 0.108 | 0.500 | -0.078 | 0.243 | 0.173 | 0.303 | 0.059 | 0.089 | 0.523 | 0.030 | 0.451 | 0.286 | 0.119 | -0.260 | 0.456 | 0.163 | 0.297 | 0.171 | -0.054 | -0.012 |
| GSM1407197 | 0.045 | -0.300 | 0.143 | 0.299 | 0.259 | 0.390 | 0.456 | 0.337 | 0.108 | 0.454 | -0.136 | 0.356 | 0.031 | 0.226 | 0.078 | 0.213 | 0.344 | -0.005 | 0.485 | 0.337 | 0.138 | -0.080 | 0.409 | 0.245 | 0.292 | 0.164 | 0.041 | -0.125 |
| GSM1407198 | 0.142 | -0.224 | 0.167 | 0.282 | 0.234 | 0.397 | 0.458 | 0.309 | 0.057 | 0.434 | -0.094 | 0.307 | 0.030 | 0.232 | 0.093 | 0.164 | 0.348 | -0.022 | 0.467 | 0.331 | 0.138 | -0.130 | 0.379 | 0.208 | 0.281 | 0.173 | 0.056 | -0.097 |
| GSM1407199 | 0.249 | -0.173 | 0.180 | 0.315 | 0.231 | 0.403 | 0.454 | 0.264 | 0.072 | 0.436 | -0.092 | 0.210 | 0.058 | 0.234 | 0.041 | 0.144 | 0.343 | -0.006 | 0.501 | 0.284 | 0.114 | -0.135 | 0.422 | 0.157 | 0.257 | 0.132 | 0.087 | -0.094 |
| GSM1407200 | 0.176 | -0.151 | 0.157 | 0.304 | 0.241 | 0.462 | 0.368 | 0.237 | 0.079 | 0.451 | 0.056 | 0.166 | 0.152 | 0.248 | 0.005 | 0.177 | 0.406 | 0.023 | 0.447 | 0.287 | 0.122 | -0.123 | 0.415 | 0.163 | 0.193 | 0.074 | 0.032 | -0.151 |
| GSM1407201 | 0.029 | -0.286 | 0.136 | 0.328 | 0.256 | 0.365 | 0.490 | 0.333 | 0.111 | 0.434 | -0.036 | 0.319 | 0.021 | 0.276 | 0.072 | 0.114 | 0.379 | -0.011 | 0.462 | 0.349 | 0.107 | -0.144 | 0.403 | 0.256 | 0.307 | 0.149 | -0.018 | -0.072 |
| GSM1407202 | 0.174 | -0.207 | 0.189 | 0.333 | 0.293 | 0.369 | 0.479 | 0.367 | 0.153 | 0.492 | -0.089 | 0.420 | 0.087 | 0.261 | 0.116 | 0.256 | 0.441 | 0.004 | 0.489 | 0.364 | 0.129 | -0.142 | 0.446 | 0.322 | 0.313 | 0.191 | 0.004 | -0.082 |
| GSM1407203 | 0.257 | -0.172 | 0.248 | 0.324 | 0.273 | 0.381 | 0.444 | 0.264 | 0.050 | 0.475 | -0.129 | 0.246 | 0.085 | 0.255 | 0.018 | 0.058 | 0.330 | -0.022 | 0.483 | 0.282 | 0.108 | -0.163 | 0.451 | 0.122 | 0.254 | 0.140 | 0.078 | -0.099 |
| GSM1407204 | 0.252 | -0.105 | 0.230 | 0.328 | 0.272 | 0.261 | 0.514 | 0.356 | 0.183 | 0.467 | -0.055 | 0.320 | 0.125 | 0.312 | -0.120 | -0.065 | 0.377 | 0.103 | 0.410 | 0.281 | 0.109 | -0.250 | 0.494 | 0.121 | 0.224 | 0.099 | -0.060 | 0.059 |
| GSM1407205 | 0.084 | -0.275 | 0.124 | 0.294 | 0.241 | 0.404 | 0.441 | 0.319 | 0.102 | 0.453 | -0.064 | 0.328 | -0.040 | 0.247 | 0.061 | 0.146 | 0.339 | 0.016 | 0.482 | 0.312 | 0.137 | -0.115 | 0.402 | 0.221 | 0.297 | 0.145 | 0.035 | -0.091 |
| GSM1407206 | 0.397 | 0.003 | 0.351 | 0.386 | 0.281 | 0.319 | 0.524 | 0.329 | 0.117 | 0.532 | -0.025 | 0.314 | 0.212 | 0.321 | -0.024 | 0.037 | 0.479 | 0.043 | 0.482 | 0.297 | 0.145 | -0.131 | 0.483 | 0.194 | 0.315 | 0.167 | -0.009 | -0.059 |
| GSM1407207 | -0.024 | -0.127 | 0.180 | 0.365 | 0.304 | 0.260 | 0.515 | 0.354 | 0.250 | 0.510 | 0.055 | 0.433 | 0.074 | 0.348 | 0.059 | 0.223 | 0.528 | 0.066 | 0.407 | 0.365 | 0.178 | 0.087 | 0.468 | 0.321 | 0.288 | 0.193 | -0.062 | 0.017 |
| GSM1407208 | -0.066 | -0.155 | 0.143 | 0.343 | 0.292 | 0.241 | 0.512 | 0.310 | 0.234 | 0.480 | -0.041 | 0.360 | 0.052 | 0.364 | 0.028 | 0.164 | 0.444 | -0.009 | 0.403 | 0.350 | 0.174 | -0.071 | 0.447 | 0.303 | 0.288 | 0.176 | -0.085 | 0.010 |
| GSM1407209 | 0.030 | -0.188 | 0.199 | 0.420 | 0.279 | 0.262 | 0.483 | 0.353 | 0.219 | 0.479 | -0.069 | 0.349 | 0.095 | 0.358 | 0.075 | 0.143 | 0.549 | 0.012 | 0.466 | 0.370 | 0.146 | 0.199 | 0.459 | 0.302 | 0.289 | 0.188 | -0.015 | -0.046 |
| GSM1407210 | 0.056 | -0.244 | 0.132 | 0.328 | 0.246 | 0.382 | 0.442 | 0.306 | 0.092 | 0.425 | -0.096 | 0.282 | 0.041 | 0.246 | 0.040 | 0.099 | 0.361 | -0.034 | 0.474 | 0.338 | 0.124 | -0.087 | 0.416 | 0.158 | 0.238 | 0.145 | 0.004 | -0.161 |
| GSM1407211 | -0.074 | -0.210 | 0.203 | 0.359 | 0.328 | 0.278 | 0.521 | 0.372 | 0.222 | 0.466 | -0.053 | 0.457 | -0.034 | 0.382 | 0.087 | 0.218 | 0.490 | 0.025 | 0.402 | 0.334 | 0.167 | -0.215 | 0.469 | 0.338 | 0.363 | 0.168 | -0.065 | 0.079 |
| GSM1407212 | 0.071 | -0.284 | 0.134 | 0.315 | 0.287 | 0.329 | 0.498 | 0.334 | 0.145 | 0.452 | -0.020 | 0.377 | -0.004 | 0.274 | 0.061 | 0.173 | 0.366 | -0.030 | 0.465 | 0.353 | 0.128 | -0.142 | 0.428 | 0.318 | 0.299 | 0.165 | -0.034 | -0.038 |
| GSM1407213 | 0.097 | -0.279 | 0.058 | 0.282 | 0.254 | 0.368 | 0.458 | 0.324 | 0.065 | 0.444 | 0.006 | 0.343 | 0.021 | 0.231 | 0.073 | 0.111 | 0.361 | -0.035 | 0.491 | 0.344 | 0.149 | -0.134 | 0.417 | 0.270 | 0.283 | 0.141 | 0.024 | -0.092 |
| GSM1407214 | 0.075 | -0.259 | 0.117 | 0.309 | 0.226 | 0.358 | 0.474 | 0.318 | 0.109 | 0.425 | -0.043 | 0.291 | 0.029 | 0.232 | 0.078 | 0.141 | 0.366 | -0.003 | 0.496 | 0.351 | 0.149 | -0.081 | 0.389 | 0.293 | 0.277 | 0.125 | -0.007 | -0.080 |
| GSM1407215 | 0.118 | -0.253 | 0.170 | 0.341 | 0.263 | 0.377 | 0.500 | 0.368 | 0.140 | 0.487 | -0.123 | 0.399 | 0.036 | 0.287 | 0.105 | 0.260 | 0.451 | 0.005 | 0.483 | 0.367 | 0.139 | -0.163 | 0.434 | 0.325 | 0.346 | 0.175 | 0.019 | -0.069 |
| GSM1407216 | 0.108 | -0.298 | 0.146 | 0.284 | 0.234 | 0.383 | 0.436 | 0.239 | 0.098 | 0.431 | -0.066 | 0.296 | 0.051 | 0.149 | 0.055 | 0.123 | 0.348 | -0.002 | 0.469 | 0.325 | 0.142 | -0.010 | 0.404 | 0.174 | 0.221 | 0.140 | 0.007 | -0.150 |
| GSM1407218 | -0.002 | -0.246 | 0.216 | 0.329 | 0.256 | 0.358 | 0.510 | 0.364 | 0.158 | 0.440 | -0.088 | 0.352 | 0.040 | 0.337 | 0.070 | 0.185 | 0.423 | 0.010 | 0.443 | 0.345 | 0.132 | -0.078 | 0.448 | 0.286 | 0.308 | 0.181 | -0.016 | -0.031 |
| GSM1407219 | -0.018 | -0.290 | 0.096 | 0.326 | 0.269 | 0.291 | 0.488 | 0.339 | 0.187 | 0.463 | -0.052 | 0.368 | 0.036 | 0.315 | -0.013 | 0.123 | 0.307 | 0.040 | 0.399 | 0.295 | 0.106 | -0.181 | 0.467 | 0.258 | 0.324 | 0.145 | -0.045 | 0.036 |
| GSM1407220 | -0.108 | -0.260 | 0.067 | 0.351 | 0.252 | 0.265 | 0.544 | 0.336 | 0.276 | 0.477 | -0.038 | 0.357 | -0.032 | 0.359 | -0.011 | 0.208 | 0.380 | 0.047 | 0.391 | 0.331 | 0.122 | -0.184 | 0.459 | 0.283 | 0.311 | 0.146 | -0.071 | 0.010 |
| GSM1407221 | 0.001 | -0.268 | 0.108 | 0.340 | 0.251 | 0.284 | 0.520 | 0.344 | 0.195 | 0.474 | 0.086 | 0.382 | 0.046 | 0.365 | 0.037 | 0.186 | 0.421 | 0.000 | 0.401 | 0.336 | 0.156 | -0.158 | 0.481 | 0.277 | 0.339 | 0.168 | -0.043 | 0.063 |
| GSM1407222 | -0.113 | -0.272 | 0.026 | 0.316 | 0.271 | 0.229 | 0.532 | 0.336 | 0.252 | 0.453 | 0.094 | 0.394 | -0.033 | 0.392 | 0.019 | 0.184 | 0.389 | 0.029 | 0.405 | 0.318 | 0.168 | -0.227 | 0.459 | 0.240 | 0.361 | 0.156 | -0.077 | 0.124 |
| GSM1407223 | -0.052 | -0.264 | 0.144 | 0.335 | 0.259 | 0.296 | 0.546 | 0.318 | 0.214 | 0.463 | -0.103 | 0.334 | 0.007 | 0.336 | 0.022 | 0.135 | 0.388 | 0.008 | 0.390 | 0.311 | 0.112 | -0.171 | 0.450 | 0.240 | 0.317 | 0.155 | -0.027 | 0.036 |
| GSM1407224 | 0.038 | -0.215 | 0.116 | 0.345 | 0.278 | 0.260 | 0.523 | 0.338 | 0.210 | 0.422 | -0.058 | 0.363 | 0.005 | 0.299 | 0.048 | 0.220 | 0.384 | 0.009 | 0.416 | 0.298 | 0.117 | -0.182 | 0.476 | 0.269 | 0.329 | 0.164 | -0.049 | 0.033 |
| GSM1407225 | 0.066 | -0.295 | 0.126 | 0.305 | 0.260 | 0.389 | 0.476 | 0.374 | 0.081 | 0.433 | -0.085 | 0.370 | 0.062 | 0.247 | 0.080 | 0.255 | 0.374 | -0.038 | 0.474 | 0.342 | 0.144 | -0.144 | 0.410 | 0.282 | 0.306 | 0.166 | 0.027 | -0.111 |
| GSM1407226 | -0.046 | -0.296 | 0.141 | 0.336 | 0.292 | 0.318 | 0.503 | 0.370 | 0.206 | 0.471 | -0.126 | 0.393 | 0.043 | 0.308 | 0.071 | 0.203 | 0.347 | 0.037 | 0.437 | 0.353 | 0.140 | -0.193 | 0.457 | 0.297 | 0.309 | 0.170 | -0.018 | -0.039 |
| GSM1407227 | -0.064 | -0.308 | 0.142 | 0.330 | 0.277 | 0.334 | 0.500 | 0.360 | 0.183 | 0.459 | -0.116 | 0.393 | -0.045 | 0.302 | 0.084 | 0.252 | 0.372 | 0.024 | 0.464 | 0.341 | 0.126 | -0.193 | 0.464 | 0.298 | 0.327 | 0.184 | -0.018 | -0.049 |
| GSM1407228 | 0.074 | -0.305 | 0.177 | 0.319 | 0.234 | 0.392 | 0.456 | 0.338 | 0.105 | 0.452 | -0.103 | 0.349 | -0.016 | 0.237 | 0.091 | 0.205 | 0.348 | -0.015 | 0.487 | 0.339 | 0.131 | -0.121 | 0.397 | 0.253 | 0.289 | 0.170 | 0.037 | -0.102 |
| GSM1407229 | 0.015 | -0.337 | 0.134 | 0.312 | 0.276 | 0.352 | 0.499 | 0.363 | 0.117 | 0.475 | -0.105 | 0.399 | -0.018 | 0.275 | 0.083 | 0.195 | 0.363 | 0.000 | 0.475 | 0.345 | 0.128 | -0.155 | 0.437 | 0.294 | 0.317 | 0.162 | 0.020 | -0.102 |
| GSM1407230 | -0.032 | -0.251 | 0.104 | 0.357 | 0.270 | 0.260 | 0.522 | 0.334 | 0.236 | 0.494 | -0.116 | 0.420 | 0.037 | 0.358 | 0.056 | 0.268 | 0.489 | 0.042 | 0.436 | 0.359 | 0.146 | -0.115 | 0.482 | 0.349 | 0.346 | 0.177 | -0.039 | 0.060 |
| GSM1407231 | 0.036 | -0.264 | 0.161 | 0.323 | 0.284 | 0.306 | 0.513 | 0.357 | 0.122 | 0.481 | -0.086 | 0.419 | 0.058 | 0.318 | 0.122 | 0.233 | 0.449 | 0.020 | 0.465 | 0.357 | 0.157 | -0.190 | 0.445 | 0.316 | 0.317 | 0.182 | 0.021 | -0.081 |
| GSM1407232 | 0.111 | -0.254 | 0.176 | 0.327 | 0.299 | 0.365 | 0.472 | 0.335 | 0.112 | 0.447 | 0.022 | 0.371 | 0.063 | 0.256 | 0.118 | 0.150 | 0.396 | -0.046 | 0.476 | 0.366 | 0.142 | -0.140 | 0.448 | 0.317 | 0.303 | 0.151 | 0.043 | -0.136 |
| GSM1407233 | 0.245 | -0.125 | 0.204 | 0.344 | 0.294 | 0.277 | 0.511 | 0.353 | 0.153 | 0.435 | -0.078 | 0.265 | 0.022 | 0.312 | -0.064 | -0.041 | 0.399 | 0.028 | 0.466 | 0.262 | 0.125 | -0.227 | 0.498 | 0.126 | 0.254 | 0.132 | 0.006 | -0.005 |
| GSM1407234 | -0.103 | -0.290 | 0.109 | 0.342 | 0.298 | 0.275 | 0.502 | 0.348 | 0.220 | 0.480 | -0.114 | 0.409 | 0.074 | 0.340 | 0.095 | 0.218 | 0.427 | -0.029 | 0.436 | 0.333 | 0.150 | -0.218 | 0.475 | 0.313 | 0.357 | 0.171 | -0.075 | -0.001 |
| GSM1407235 | 0.298 | -0.006 | 0.358 | 0.365 | 0.325 | 0.259 | 0.542 | 0.336 | 0.131 | 0.509 | -0.019 | 0.316 | 0.184 | 0.381 | -0.029 | -0.028 | 0.503 | 0.064 | 0.484 | 0.287 | 0.133 | -0.290 | 0.509 | 0.190 | 0.288 | 0.165 | -0.044 | 0.063 |
| GSM1407236 | 0.626 | 0.222 | 0.477 | 0.388 | 0.323 | 0.309 | 0.572 | 0.325 | 0.252 | 0.529 | 0.105 | 0.378 | 0.649 | 0.302 | -0.009 | 0.091 | 0.608 | 0.166 | 0.460 | 0.323 | 0.190 | -0.218 | 0.483 | 0.383 | 0.358 | 0.246 | -0.113 | 0.066 |
| GSM1407237 | 0.343 | -0.048 | 0.212 | 0.342 | 0.295 | 0.248 | 0.501 | 0.333 | 0.138 | 0.436 | -0.124 | 0.256 | 0.200 | 0.361 | -0.090 | -0.075 | 0.377 | 0.046 | 0.442 | 0.255 | 0.058 | -0.235 | 0.513 | 0.093 | 0.248 | 0.153 | -0.017 | 0.017 |
| GSM1407238 | 0.161 | -0.093 | 0.280 | 0.374 | 0.316 | 0.275 | 0.502 | 0.377 | 0.156 | 0.484 | -0.123 | 0.302 | 0.082 | 0.364 | -0.051 | -0.024 | 0.419 | 0.033 | 0.451 | 0.258 | 0.081 | -0.234 | 0.508 | 0.160 | 0.235 | 0.139 | -0.017 | 0.061 |
| GSM1407239 | -0.052 | -0.240 | 0.128 | 0.324 | 0.310 | 0.253 | 0.499 | 0.346 | 0.254 | 0.446 | -0.054 | 0.416 | 0.009 | 0.316 | 0.059 | 0.140 | 0.377 | 0.031 | 0.380 | 0.299 | 0.158 | -0.179 | 0.480 | 0.297 | 0.314 | 0.152 | -0.059 | 0.064 |
| GSM1407240 | 0.168 | -0.189 | 0.134 | 0.329 | 0.306 | 0.224 | 0.477 | 0.265 | 0.128 | 0.403 | -0.119 | 0.213 | -0.061 | 0.349 | -0.098 | -0.057 | 0.255 | 0.095 | 0.480 | 0.204 | 0.098 | -0.224 | 0.483 | 0.022 | 0.193 | 0.091 | 0.000 | 0.051 |
| GSM1407241 | 0.047 | -0.351 | 0.130 | 0.318 | 0.228 | 0.360 | 0.474 | 0.330 | 0.139 | 0.455 | -0.070 | 0.350 | 0.078 | 0.247 | 0.079 | 0.213 | 0.386 | -0.034 | 0.498 | 0.346 | 0.136 | -0.137 | 0.436 | 0.315 | 0.319 | 0.176 | -0.026 | -0.093 |
| GSM1407242 | 0.055 | -0.321 | 0.186 | 0.336 | 0.212 | 0.355 | 0.517 | 0.354 | 0.114 | 0.477 | -0.009 | 0.323 | 0.074 | 0.261 | 0.076 | 0.182 | 0.366 | 0.016 | 0.472 | 0.347 | 0.144 | -0.179 | 0.461 | 0.328 | 0.337 | 0.176 | -0.020 | -0.117 |
| GSM1407243 | 0.244 | -0.220 | 0.134 | 0.349 | 0.292 | 0.275 | 0.496 | 0.316 | 0.110 | 0.391 | -0.106 | 0.257 | 0.086 | 0.324 | -0.075 | 0.076 | 0.335 | 0.006 | 0.448 | 0.236 | 0.065 | -0.217 | 0.492 | 0.136 | 0.257 | 0.121 | 0.006 | 0.004 |
| GSM1407244 | 0.011 | -0.262 | 0.101 | 0.344 | 0.263 | 0.326 | 0.508 | 0.352 | 0.189 | 0.415 | -0.046 | 0.401 | 0.010 | 0.361 | 0.107 | 0.198 | 0.452 | -0.022 | 0.488 | 0.338 | 0.136 | -0.090 | 0.467 | 0.331 | 0.312 | 0.172 | -0.011 | -0.032 |
| GSM1407245 | 0.050 | -0.233 | 0.170 | 0.336 | 0.304 | 0.315 | 0.516 | 0.341 | 0.159 | 0.458 | -0.040 | 0.409 | 0.036 | 0.301 | 0.088 | 0.220 | 0.456 | -0.010 | 0.457 | 0.352 | 0.140 | -0.132 | 0.439 | 0.305 | 0.333 | 0.193 | -0.005 | -0.062 |
| GSM1407246 | 0.082 | -0.319 | 0.059 | 0.323 | 0.273 | 0.344 | 0.481 | 0.293 | 0.117 | 0.404 | -0.119 | 0.344 | -0.016 | 0.234 | 0.031 | 0.135 | 0.290 | 0.003 | 0.482 | 0.356 | 0.116 | -0.154 | 0.463 | 0.285 | 0.308 | 0.143 | 0.005 | -0.148 |

**Supplementary Table S10.** Immune cells in disease and normal samples were compared by the Wilcox test method

| **gene** | **immune_cells** | **cor** | **p.value** | **gene** | **immune_cells** | **cor** | **p.value** |
| --- | --- | --- | --- | --- | --- | --- | --- |
| NKD2 | CD56bright natural killer cell | -0.475 | <0.001 | S100A11 | CD56bright natural killer cell | 0.678 | <0.001 |
| NKD2 | CD56dim natural killer cell | 0.783 | <0.001 | S100A11 | CD56dim natural killer cell | -0.641 | <0.001 |
| NKD2 | Central memory CD4 T cell | -0.616 | <0.001 | S100A11 | Central memory CD4 T cell | 0.502 | <0.001 |
| NKD2 | Central memory CD8 T cell | -0.152 | 0.230 | S100A11 | Central memory CD8 T cell | 0.470 | <0.001 |
| NKD2 | Effector memeory CD4 T cell | -0.742 | <0.001 | S100A11 | Effector memeory CD4 T cell | 0.585 | <0.001 |
| NKD2 | Gamma delta T cell | -0.227 | 0.072 | S100A11 | Gamma delta T cell | 0.358 | 0.004 |
| NKD2 | Immature dendritic cell | -0.686 | <0.001 | S100A11 | Immature dendritic cell | 0.727 | <0.001 |
| NKD2 | Neutrophil | 0.465 | <0.001 | S100A11 | Neutrophil | -0.358 | 0.004 |
| NKD2 | Plasmacytoid dendritic cell | -0.742 | <0.001 | S100A11 | Plasmacytoid dendritic cell | 0.674 | <0.001 |
| NKD2 | Regulatory T cell | -0.104 | 0.412 | S100A11 | Regulatory T cell | 0.194 | 0.125 |
| NKD2 | T follicular helper cell | -0.289 | 0.021 | S100A11 | T follicular helper cell | 0.192 | 0.128 |
| NKD2 | Type 2 T helper cell | -0.801 | <0.001 | S100A11 | Type 2 T helper cell | 0.595 | <0.001 |

- **Supplementary Table S11.** Clinical features of the TAO group

|  | **N**  **Sample/people** | **Duration** | **Thyroid status** | **Clinical stage** | **Clinical activity scores** | **Thyroid antibody status** |
| --- | --- | --- | --- | --- | --- | --- |
| GSE58331 | 35/25 | unknown | hyperthyroid(19);  hypothyroid(1);  euthyroid(3);  unknown  (2). | Twenty of the patients had orbital surgery for symptomatic relief. | unknown | positive  (3);  negative(2);  unknown(20). |
| GSE105149 | 7/4 | unknown | unknown | unknown | unknown | unknown |
| GSE175399 | 4 | 46.50 ± 7.89 months | unknown | unknown | less  than 3 | unknown |
| qRT-PCR | 5 | 11.75± 5.40months | hyperthyroid | moderate to severe | 3-4 | positive  (5) |
