# Supplementary figures and images for: Machine learning-based prediction of diagnostic markers for Graves’ orbitopathy
Source: Endocrine. 2023 Apr 15;81(2):277–89. doi: 10.1007/s12020-023-03349-z (PMC10293385; doi:10.1007/s12020-023-03349-z)

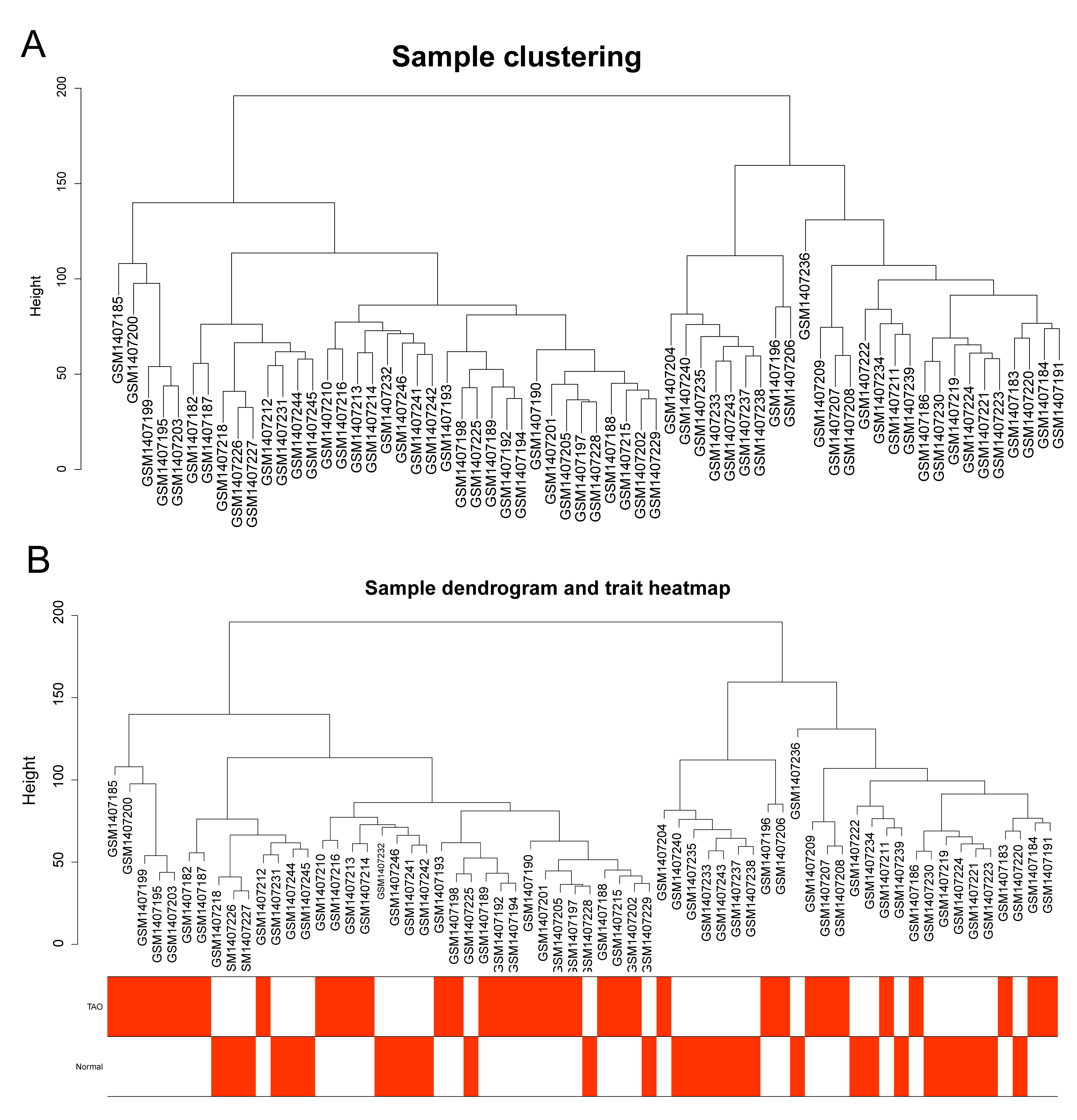

Supplement: Supplementary file 2 — Supplementary material figure 1 [file 12020_2023_3349_MOESM2_ESM.tif]
