# Supplementary material for: Machine learning-based prediction of diagnostic markers for Graves’ orbitopathy
Source: Endocrine. 2023 Apr 15;81(2):277–89. doi: 10.1007/s12020-023-03349-z (PMC10293385; doi:10.1007/s12020-023-03349-z)
Supplement: Supplementary file 3 — Supplementary Figure 1 legend [file 12020_2023_3349_MOESM3_ESM.docx]

**Supplementary materials**

**Supplementary Figure 1** Clustering plot and clustering dendrogram of samples. (A) The sample clustering plot showed that the samples clustered well. (B) Clustering dendrogram of samples based on their Euclidean distance.
